# Enantioselective synthesis of 1,2-disubstituted thiocyclobutanes *via* Michael addition†

**DOI:** 10.1039/d5sc01727k

**Published:** 2025-05-23

**Authors:** Emma G. L. Robert, Jerome Waser

**Affiliations:** a Laboratory of Catalysis and Organic Synthesis, Institute of Chemical Sciences and Engineering, Ecole Polytechnique Fédérale de Lausanne 1015 Lausanne Switzerland jerome.waser@epfl.ch

## Abstract

We report the diastereoselective and enantioselective synthesis of thio-substituted cyclobutanes *via* a sulfa-Michael addition using cyclobutenes. In the presence of DBU, various thio-cyclobutane esters and amides were obtained in up to quantitative yield and >95 : 5 dr. Using a chiral chinchona-based squaramide bifunctional acid–base catalyst and an *N*-acyl-oxazolidinone-substituted cyclobutene, thio-cyclobutanes were obtained with high yield and enantioselectivity (er up to 99.7 : 0.3).

## Abstract 2

We report a diastereoselective and enantioselective thio-Michael addition using cyclobutenes catalyzed by a bifunctional cinchona-squaramide catalyst.

## Graphical abstract

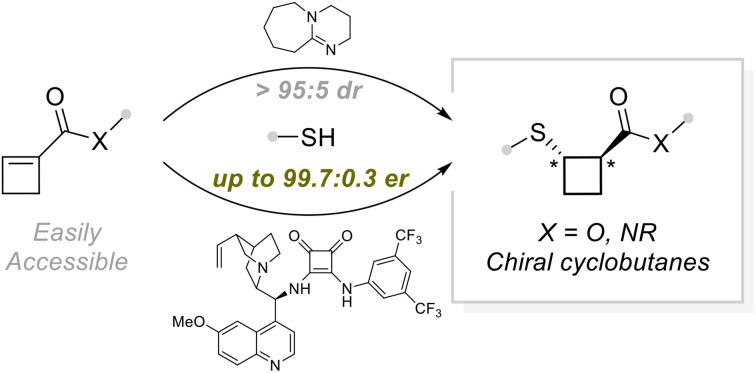

## Introduction

**Scheme 1:**
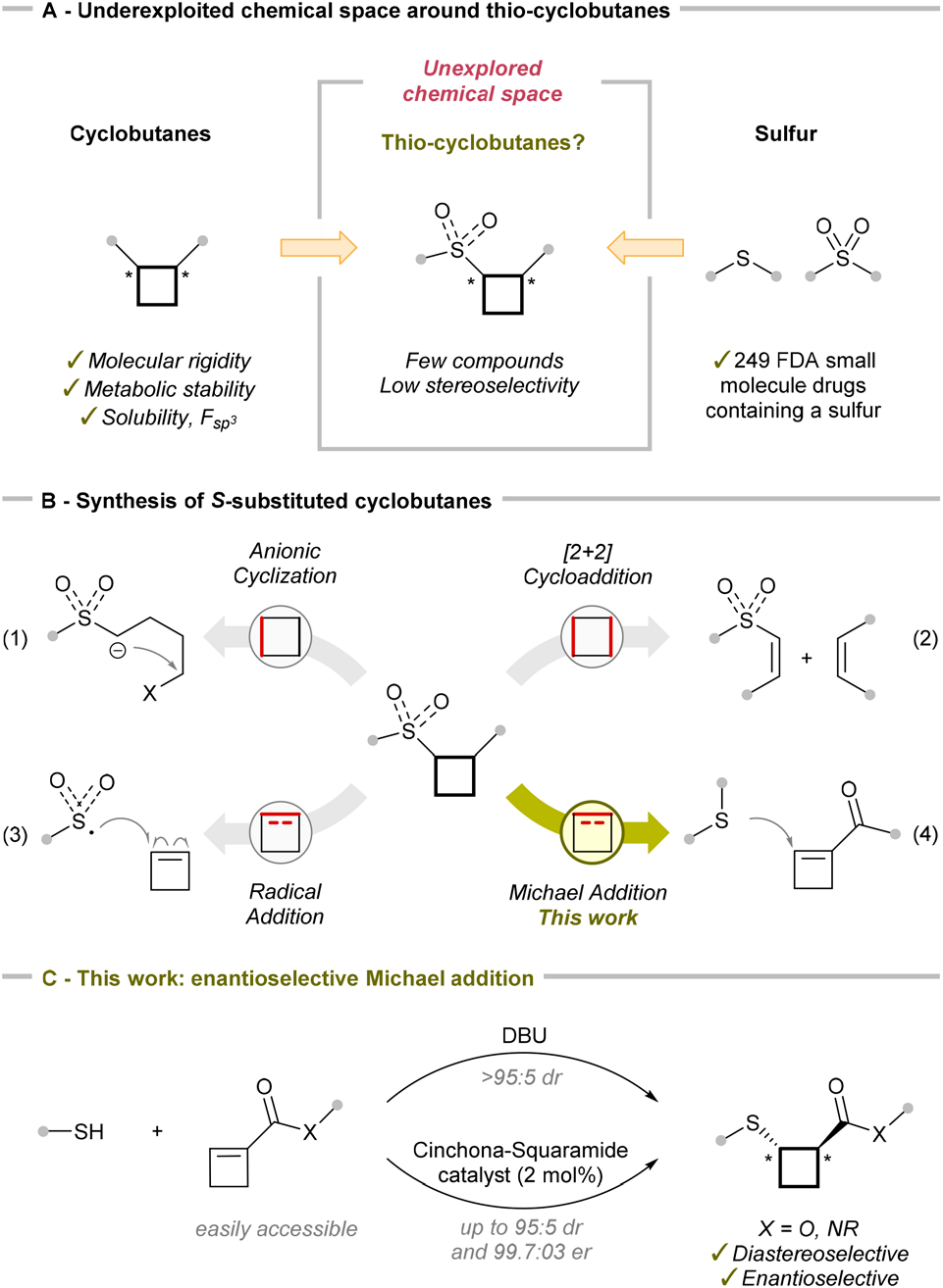
*S*-Substituted cyclobutanes in medicinal chemistry (A), synthetic strategies (B), and this work (C).

The incorporation of strained saturated rings into drug candidates has been widely adopted in medicinal chemistry.^1^ This approach gives access to compounds with enhanced molecular rigidity and often improved metabolic stability and solubility due to an increased fraction of sp^3^-hybridized carbons (*F*_sp^3^_). Among strained rings, cyclobutanes have established themselves as important structural motifs in drug development,^2^ and accessing di-substituted derivatives with high diastereoselectivity and enantioselectivity has become an area of intense research in synthetic chemistry (Scheme 1A).^3^ In addition, sulfur atoms are widely utilized in pharmaceuticals due to their unique electronic and structural properties, appearing in various functional groups such as thioethers and sulfones.^4^ In this regard, sulfur-substituted cyclobutanes have been investigated for their conformational rigidity, particularly in comparison to non-cyclic derivatives such as the drug captopril.^5^ The configuration of thio-containing stereocenters in 1,2-disubstituted cyclobutanes has been shown to significantly influence their bioactivity. Nevertheless, the thio-substituted cyclobutane motif remains largely underexplored in medicinal chemistry and drug development, with only a few examples reported in the patent literature.^6^ This can be attributed to the limited synthetic strategies available to synthesize this motif, especially in an enantiopure form, reinforcing the need for further exploration and development in this area.

There are currently four main strategies for accessing sulfur-substituted cyclobutanes: cyclization of acyclic precursors by deprotonation in the α-position to a sulfone, sulfoximine or sulfoxide and the subsequent substitution reaction (Scheme 1 and eqn (1));^7^ thermal-catalyzed,^8^ Lewis acid-catalyzed^9^ or photo-catalyzed^10^ [2 + 2] cycloaddition (Scheme 1 and eqn (2)); incorporating a sulfur group into an existing cyclobutene, either *via* radical intermediates (Scheme 1 and eqn (3))^11–14^ or through Michael addition of a thio nucleophile (Scheme 1 and eqn (4)).^5*b*,6,8,15,16^ The ring expansion of cyclopropanes to sulfur-substituted cyclobutanes was also explored, but it occurred with low efficiency.^17^

Concerning the radical sulfur addition pathway, Witiak and co-workers reported in 1972 the first two examples of Giese addition of thioacetic acid and benzyl thiol radicals generated by light irradiation of cyclobut-1-ene-1-carboxylic acid.^11^ These conditions led to a mixture of isomers in the case of benzyl thiol. In 2020, Gouverneur and co-workers used sulfonyl^12^ and sulfamoyl^13^ chlorides for the hydrosulfonylation and hydrosulfamoylation of activated alkenes under visible light catalysis. They described five examples using cyclobutene esters and amides as alkenes to obtain sulfoxide-substituted cyclobutanes majorly with a *cis*-configuration. In the same year, Landais and co-workers reported the sulfonylcyanation of chiral cyclobutenes under light irradiation.^14^ In this case, a *trans*-relationship between the *N*-acyl-oxazolidinone and the sulfonyl group was obtained.

Surprisingly, although the Michael addition of nitrogen nucleophiles to cyclobutenes has been exploited,^18^ its application to sulfur-based nucleophiles has remained largely unexplored. Ciabatti and co-workers reported that the addition of thioacetic acid to cyclobut-1-ene-1-carboxylic acid was also possible without generation of the radical but proceeded with only 7 : 3 dr.^5*b*^ Probably, due to the low selectivity, this method found only occasional use for the generation of simple building blocks for medicinal chemistry projects.^6^ Besides addition to cyclobut-1-ene-1-carboxylic acid, the only other cases of Michael addition involve a non-selective addition of glutathione to a conjugated ketone in the context of metabolic intermediate studies,^15*a*^ as well as additions to cyclobutene esters on a biased bicyclic system^15*b*^ and on a polyhalogenated derivative.^8^ In 2019, Aitken and co-workers reported access to β-sulfinyl cyclobutane amides *via* a rearrangement of α-sulfinyl precursors.^16^ The scope was limited to aryl sulphoxides, and the products were obtained in a diastereomeric mixture due to the stereogenic sulphoxide. Overall, highly diastereoselective Michael additions of thio-nucleophiles to cyclobutene esters remain extremely rare, and no enantioselective method has been reported yet. This is surprising considering that numerous examples of enantioselective sulfa-Michael additions using less strained substrates have been reported over the past two decades, primarily relying on the use of chiral metal complexes or organocatalysts.^19^

Herein, we describe the first highly diastereoselective (>95 : 5 dr) and enantioselective (up to 99.7 : 0.3 er) synthesis of thio-cyclobutanes. Starting from commercially available thiols and readily available cyclobutenes, our method enables the efficient formation of a wide range of thio-substituted cyclobutane esters and amides. High diastereoselectivities were first achieved just by using DBU as a base. With a chiral chinchona squaramide catalyst, thio-cyclobutanes were then accessed with high enantioselectivity. This novel method therefore offers for the first time control over both diastereoselectivity and enantioselectivity for the synthesis of 1,2-substituted thio-cyclobutane esters, offering efficient access to a currently underexploited chemical space for applications in medicinal chemistry.

## Results and discussion

### Reaction design and optimization

We began our investigation with the reaction of 2-bromothiophenol (1a) and readily accessible benzyl ester cyclobutene 2a,^20^ using K_2_CO_3_ as a base in MeCN at room temperature for 18 hours (Table 1, entry 1). To our satisfaction, the corresponding thio-cyclobutane ester 3a was obtained in 72% yield, but no diastereoselectivity was observed (50 : 50 dr). Switching K_2_CO_3_ for NEt_3_ (entry 2) or TMG (entry 3) increased the yield but resulted in a low dr (95% yield, 51 : 49 dr and 90% yield, 64 : 36 dr, respectively). TBD offered the desired product in 69% yield and 72 : 28 dr (entry 4). In contrast, DBU provided the *trans*-product in quantitative yield and >95 : 5 dr (entry 5). Changing the solvent from MeCN to EtOAc resulted in the same yield but lower dr (82 : 18) (entry 6). Lowering the reaction time to 1 or 2 hours did not affect the yield but lowered the diastereoselectivity to 64 : 36 after 1 hour (entry 7) and to 71 : 29 (entry 8) after 2 hours, suggesting thermodynamic control of the dr. Finally, conducting the reaction under an air atmosphere (entry 9) or with HPLC-grade MeCN (entry 10) resulted in a lower diastereoselectivity (86 : 14 dr and 89 : 11 dr, respectively), probably due to the presence of water, which can slow down the epimerization.

**Table 1:** Optimization of the Michael addition of 2-bromothiophenol (1a) to cyclobutene 2a^a^

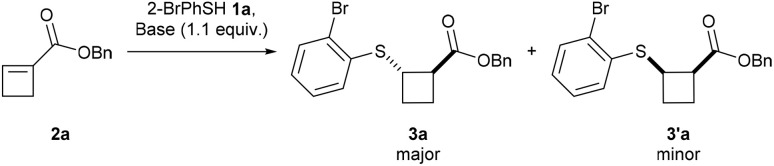					
Entry	Base	Solvent	Time	Yield^b^	dr^c^
1	K_2_CO_3_	MeCN	18 h	72%	50 : 50
2	NEt_3_	MeCN	18 h	95%	51 : 49
3	TMG	MeCN	18 h	90%	64 : 36
4	TBD	MeCN	18 h	69%	72 : 28
**5**	**DBU**	**MeCN**	**18 h**	**Quant.**	**>95 : 5**
6	DBU	EtOAc	18 h	Quant.	82 : 18
7	DBU	MeCN	1 h	Quant.	64 : 36
8	DBU	MeCN	2 h	98%	71 : 29
9	DBU	MeCN + air	18 h	81%	86 : 14
10	DBU	HPLC-MeCN	18 h	97%	89 : 11

a 1.0 equiv. thiol 1a (0.1 mmol), 1.1 equiv. cyclobutene 2a, 1.1 equiv. base, rt.

b ^1^H NMR of the crude mixture with dibromomethane as an internal standard.

c Measured from the crude ^1^H NMR.

### Scope of the diastereoselective Michael addition

**Scheme 2:**
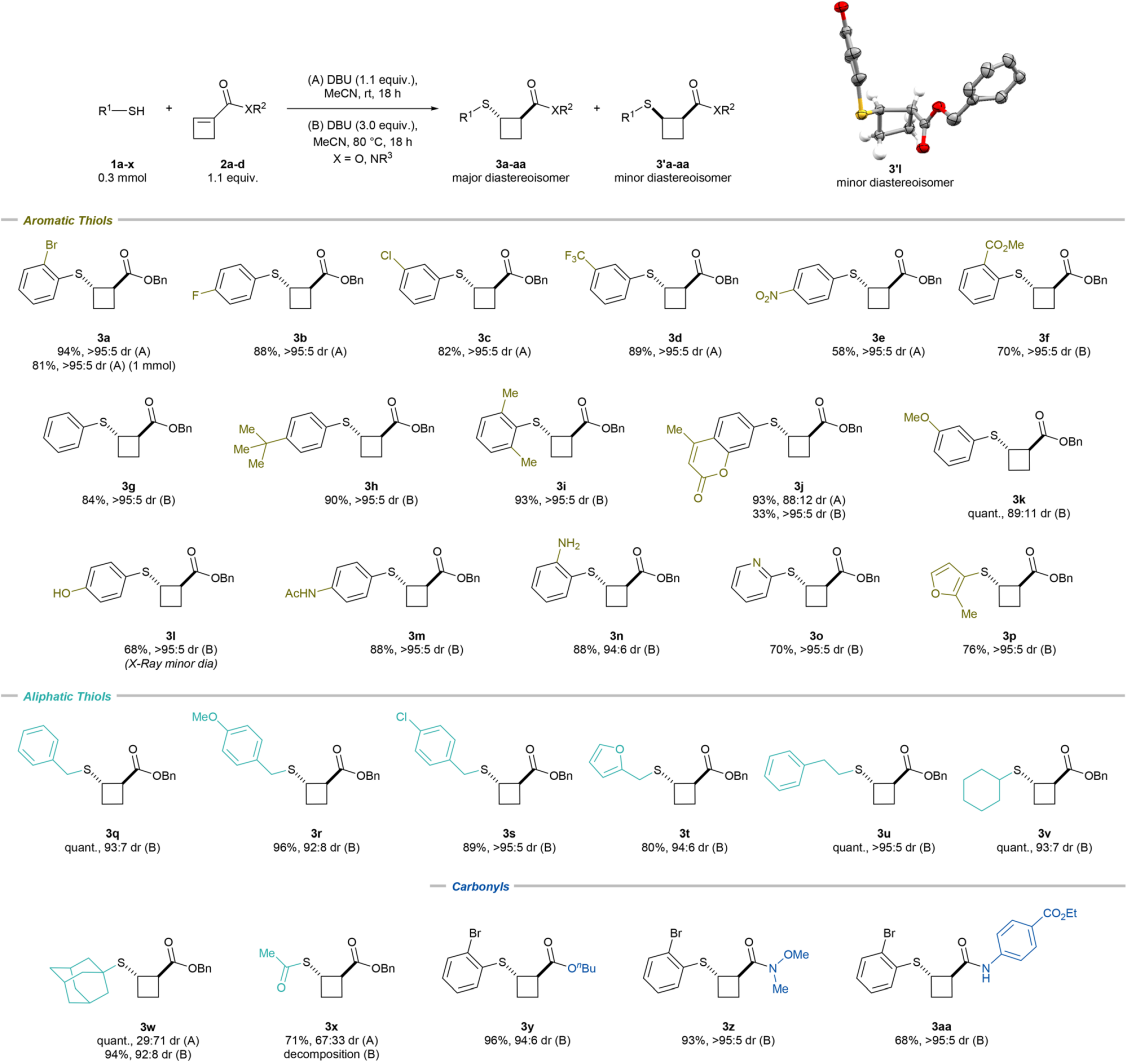
Scope of thiols and cyclobutenes in the racemic sulfa-Michael addition. Reaction conditions: (A) 0.3 mmol *S*-nucleophile 1, 1.1 equiv. cyclobutene 2, 1.1 equiv. DBU, MeCN [0.1 M], rt, 18 h. (B) 0.3 mmol *S*-nucleophile 1, 1.1 equiv. cyclobutene 2, 3.0 equiv. DBU, MeCN [0.1 M], 80 °C, 18 h. For the X-ray structure of 3′l, the H atoms not involved in the cyclobutane ring are omitted for clarity; thermal ellipsoids are given at 50% probability.

With the optimized conditions in hand, we began investigating the scope of aromatic thiols (Scheme 2). The model substrate 3a was obtained in 94% isolated yield and >95 : 5 dr on a 0.3 mmol scale. Scaling up the reaction to 1 mmol offered the desired product in 81% yield and an identical dr. A 4-fluorine or 3-chlorine substituent on the benzene moiety was well tolerated and led to 3b (88% yield and >95 : 5 dr) and 3c (82% yield and >95 : 5 dr), respectively. Other electron-withdrawing groups could be introduced, such as a CF_3_ (3d; 89% yield and >95 : 5 dr), a NO_2_ (3e; 58% yield and >95 : 5 dr) or a methyl ester (3f; 70% yield and >95 : 5 dr) group. For the latter, 3.0 equivalents of DBU and heating to 80 °C were necessary to obtain the desired product with high diastereoselectivity (method (B)). These conditions showed improved diastereoselectivity for all the thiols not bearing an electron-withdrawing group. Simple thiophenol provided 3g in 84% yield and >95 : 5 dr. *tert*-Butylthiophenol led to 3h in 90% yield and >95 : 5 dr, while 2,6-dimethylthiophenol offered 3i in 93% yield and >95 : 5 dr. A chromenone-substituted thiol was tolerated, and 3j was obtained in 93% yield and 88 : 12 dr with method (A) and in 33% yield and >95 : 5 dr with method (B). Methoxy-substituted thiophenol provided 3k in quantitative yield and 89 : 11 dr. A free alcohol, an acetamide and a free amine led to 3l (68% yield and >95 : 5 dr), 3m (88% yield and >95 : 5 dr) and 3n (88% yield and 94 : 6 dr), respectively. The X-ray structure of 3'l confirmed the molecular structure and relative configuration of the minor *cis*-diastereoisomer.^21^

Heteroaromatic-substituted thiols could also be introduced; 2-pyridine as well as furan-substituted thiol provided 3o (70% yield and >95 : 5 dr) and 3p (76% yield and >95 : 5 dr), respectively. Moreover, benzyl thiol provided 3q in quantitative yield and 93 : 7 dr. Similarly, methoxy- and chloro-substituted benzyl thiols could be introduced, giving 3r (96% yield and 92 : 8 dr) and 3s (89% yield and >95 : 5 dr), respectively. Furan-2-ylmethanethiol provided 3t (80% yield and 94 : 6 dr). A primary alkyl-substituted thiol led to 3u (quant. yield and >95 : 5 dr), while a secondary thiol gave 3v (quant. yield, 93 : 7 dr). The sterically hindered adamantyl thiol led to 3w in quantitative yield and 29 : 71 dr with method (A) and 94% yield and 92 : 8 dr with method (B). Thioacetic acid provided 3x in 71% yield and 67 : 33 dr with method (A). In this case, the use of method (B) could not improve the diastereoselectivity as decomposition was observed. Finally, replacement of the benzyl ester was implemented with an *n*-butyl ester (3y; 96% yield and 94 : 6 dr), a Weinreb amide (3z; 93% yield and >95 : 5 dr) or a benzocaine-substituted amide (3aa; 68% yield and >95 : 5 dr).

### Development of the enantioselective reaction

**Scheme 3:**
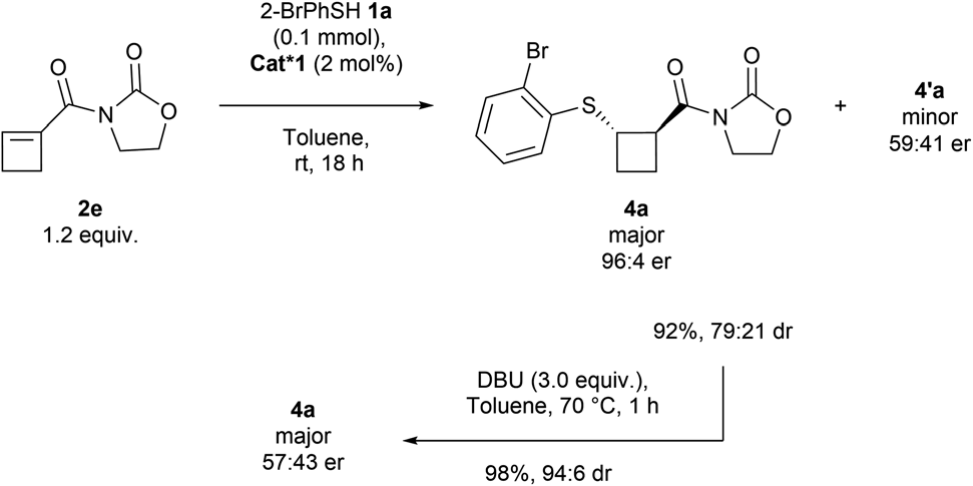
Enantioselective reaction with Cat*1 and an attempt at epimerization.

**Fig. 1:**
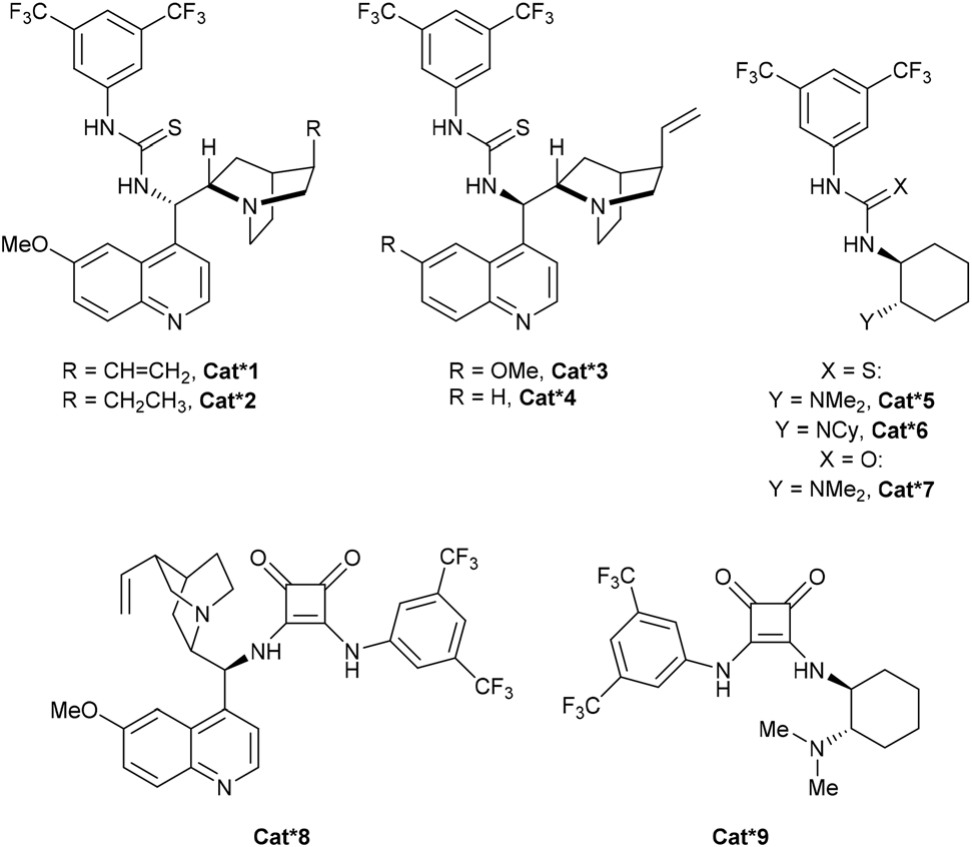
Structures of chiral bifunctional organocatalysts used in the optimization of the enantioselective sulfa-Michael addition.

For our investigation of the enantioselective sulfa-Michael addition of thiols onto cyclobutenes, we turned our attention to cyclobutene 2e substituted with a simple oxazolidinone auxiliary, which has demonstrated its versatility in asymmetric reactions (Scheme 3).^19^ Based on previous reports,^19*j*–*l*^ chiral bifunctional acid–base catalysts Cat*1–9 containing thiourea, urea and squaramides as well as chinchona alkaloids or chiral diamines were selected (Fig. 1).

Using 2 mol% of the commercially available thiourea cinchona catalyst Cat*1 in toluene at room temperature overnight, we were pleased to obtain the desired product in 92% yield, modest diastereoselectivity of 79 : 21 and good enantiomeric ratio of 96 : 4 for the major diastereoisomer (Scheme 3 and Table 2, entry 1), while the er of the minor diastereoisomer was lower (59 : 41 er). To improve the dr, 3.0 equivalents of DBU were added and the reaction mixture was heated to 70 °C for one hour. An improvement of dr was observed (94 : 6 dr compared to 79 : 21 dr), but the er dropped to 57 : 43, suggesting that the epimerization occurred *via* a retro-Michael/Michael-addition process rather than a simple deprotonation. This hypothesis was confirmed by adding a second thiol to the crude reaction mixture after full conversion, resulting in a mixture of the two thiol-substituted products (see Section 4.1 in the ESI†).

**Table 2:** Optimization of the enantioselective sulfa-Michael addition of 2-bromothiophenol (1a) to cyclobutene 2e^a^

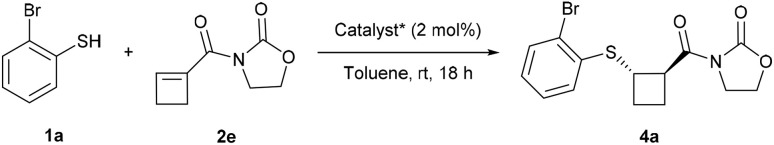				
Entry	Catalyst	Yield^b^	dr^c^	er^d^
1	Cat*1	92%	79 : 21	96 : 4
2	Cat*2	Quant.	85 : 15	96 : 4
3	Cat*3	Quant.	71 : 29	6 : 94
4	Cat*4	86%	48 : 52	5 : 95
5	Cat*5	98%	82 : 18	92 : 8
6	Cat*6	Quant.	55 : 45	38 : 62
7	Cat*7	84%	66 : 34	89 : 11
**8**	Cat*8	**99%**	**89 : 11**	**98 : 2**
9	Cat*9	98%	91 : 9	90 : 10

a 1.0 equiv. thiol 1a (0.1 mmol), 1.2 equiv. cyclobutene 2e, 2 mol% Cat*, toluene [0.1 M], rt, 18 h.

b ^1^H NMR of the crude mixture with dibromomethane as an internal standard.

c Measured from the crude ^1^H NMR.

d Measured from the SFC chromatogram.

We then turned our attention to other chiral organocatalysts in order to further improve the diastereoselectivity under kinetic control. Cat*2 with a hydrogenated quinuclidine substituent led to an improvement in dr (85 : 15) and the same er (Table 2, entry 2). Cat*3 with an inverted urea stereocenter gave a quantitative yield, but lower dr (71 : 29) and er (6 : 94) than Cat*1. Removing the methoxy group of the quinoline backbone (Cat*4) led to a low dr (entry 4). Takemoto's thiourea (Cat*5–6) and urea (Cat*7) type of catalysts led to a decrease in diastereoselectivity and enantioselectivity (entries 5–7). Squaramide-based cinchona Cat*8 gave the best results with 99% yield, 89 : 11 dr and 98 : 2 er (entry 8). Replacement of the chinchona by a chiral diamine moiety (Cat*9) gave a similar yield (98%), a higher dr (91 : 9), but a lower enantioselectivity (90 : 10 er) (entry 9). Cat*8 was then selected as the best catalyst, and further fine-tuning of the conditions was performed, but no significant improvement of neither the dr nor the er could be obtained (see the ESI†). In addition, performing the reaction with ester-substituted cyclobutene 2a led to the formation of thiocyclobutane 3a in a racemic form, confirming the essential role of the oxazolidinone auxiliary.

### Scope of the enantioselective reaction

**Scheme 4:**
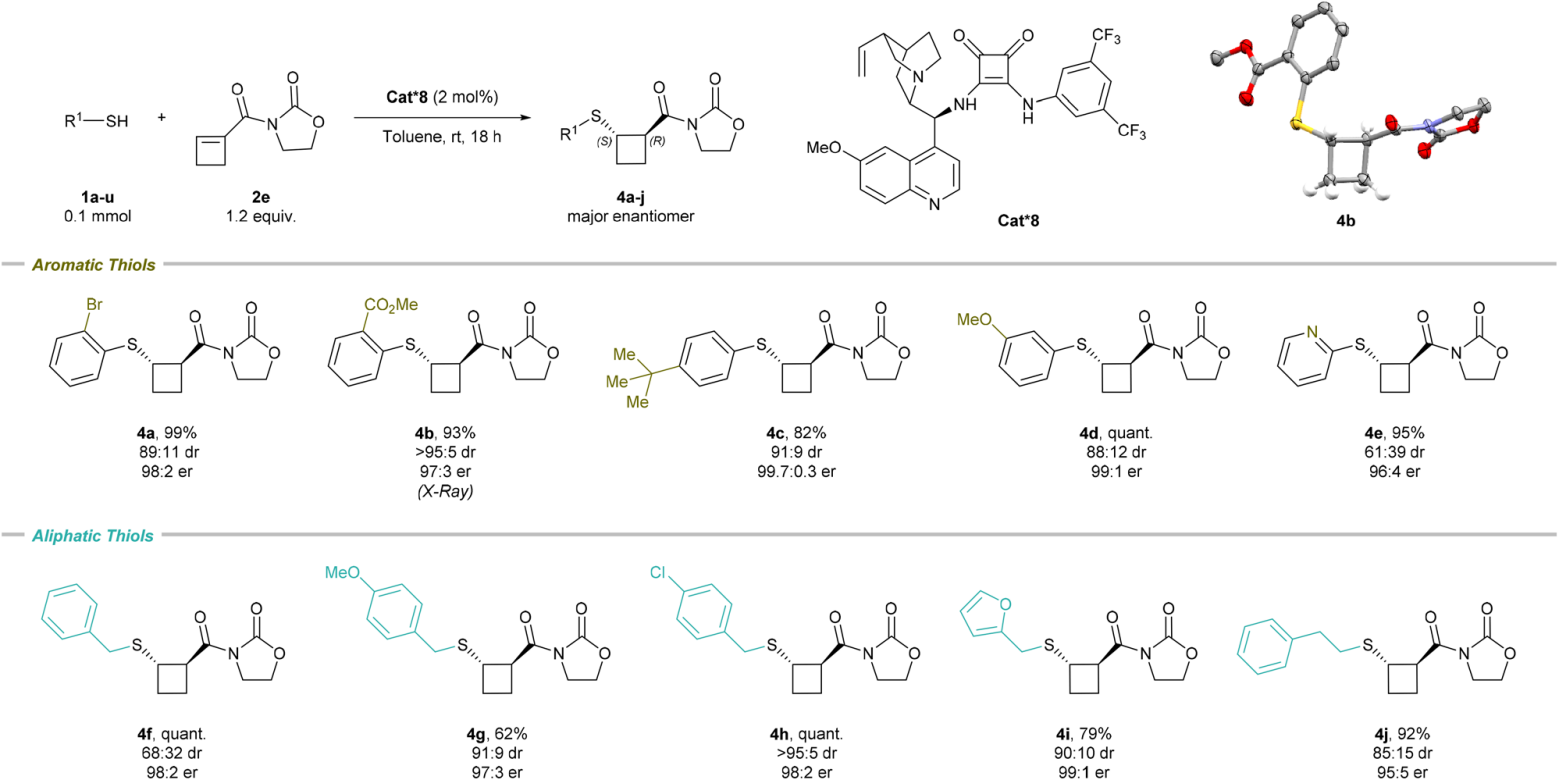
Scope of the enantioselective sulfa-Michael addition on cyclobutene. Reaction conditions: 0.1 mmol *S*-nucleophile 1, 1.2 equiv. cyclobutene 2e, 2 mol% Cat*8, toluene [0.1 M], rt, 18 h. The er of the major diastereoisomer is given. For the X-ray structure of 4b, the H atoms not involved in the cyclobutane ring are omitted for clarity; thermal ellipsoids are given at 50% probability.

The scope of the thiols was then examined for the enantioselective transformation (Scheme 4). Scaling up the reaction to 0.8 mmol led to the formation of 4a in similar yield, increased dr, and identical er (95% yield, 91 : 9 dr, and 98 : 2 er). Among aromatic thiols, an ester electron-withdrawing group, a neutral bulky *tert*-butyl group, and a methoxy electron-donating group all smoothly underwent the transformation with high diastereo- and enantioselectivity; 4b (93% yield, >95 : 5 dr, and 97 : 3 er), 4c (82% yield, 91 : 9 dr, and 99.7 : 0.3 er), and 4d (quant. yield, 88 : 12 dr, and 99 : 1 er) were obtained, respectively. The X-ray structure of 4b confirmed the molecular structure and absolute configuration of the major *trans*-enantiomer as (*S*-,*R*-), in accordance with Houk's Brønsted acid–hydrogen bonding stereoinduction model (see the ESI, Fig. S1†).^22^ 2-Pyridine-substituted thiol produced 4e (95% yield, 61 : 39 dr, and 96 : 4 er). Aliphatic thiols were also suitable in the enantioselective transformation. Benzyl thiol 4f (quant. yield, 68 : 32 dr, and 98 : 2 er) and substituted benzyl thiol 4g (62% yield, 91 : 9 dr, and 97 : 3 er) and 4h (quant. yield, >95 : 5 dr, and 98 : 2 er) were tolerated. Furan-2-ylmethanethiol produced 4i (79% yield, 90 : 10 dr, and 99 : 1 er), while 2-phenylethane-1-thiol gave 4j (92% yield, 85 : 15 dr, and 95 : 5 er). However, bulkier thiols such as cyclohexyl thiol and adamantyl thiol were not compatible with the reaction and provided the desired product in low yields (14% and <5%, respectively).

### Product modifications

**Scheme 5:**
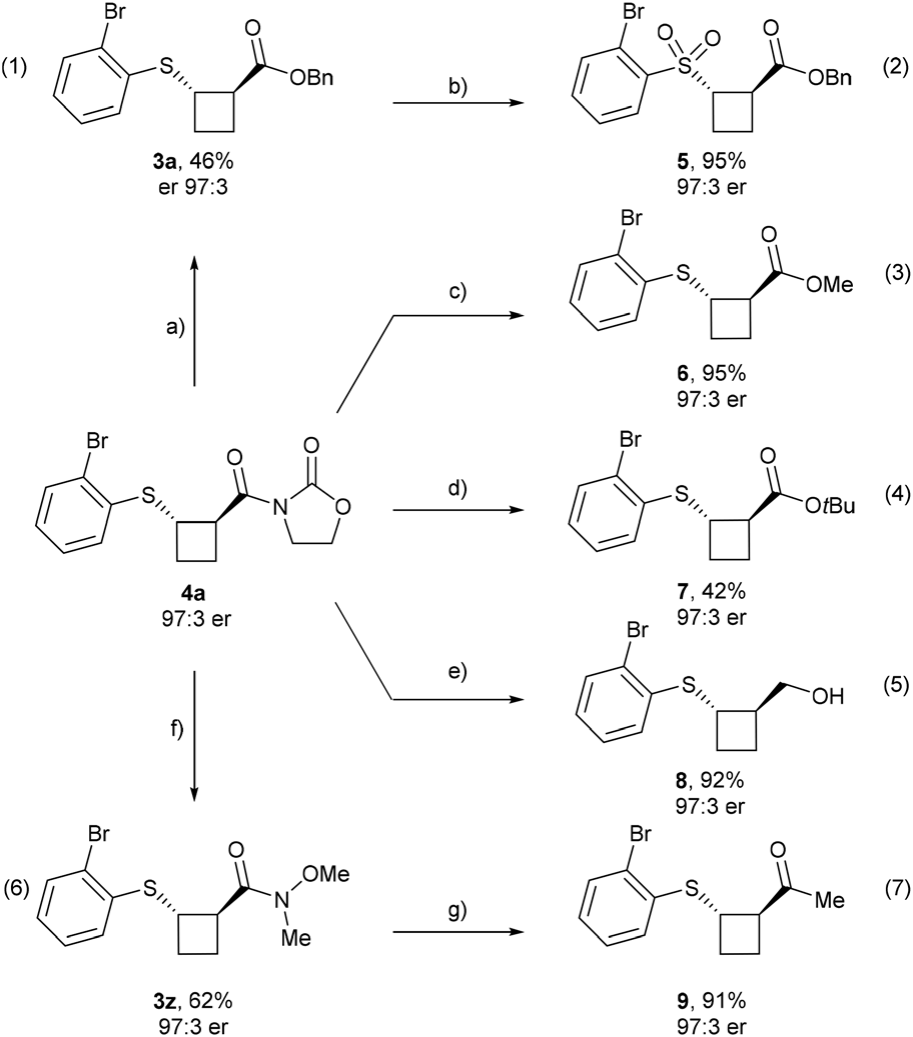
Transformations of enantioenriched 4a. Reaction conditions: (a) DMAP (0.3 equiv.), BnOH, rt, 18 h. (b) *m*CPBA (2.5 equiv.), DCM, rt, 3 h. (c) DMAP (0.3 equiv.), MeOH, rt, 2 h. (d) (1) LiOH·H_2_O (2.0 equiv.), H_2_O_2_ (6.0 equiv.), THF : H_2_O, rt, 18 h; (2) DMAP (0.1 equiv.), Boc_2_O (1.5 equiv.), *t*BuOH (2.0 equiv.), NEt_3_ (2.0 equiv.), neat, rt, 18 h. (e) NaBH_4_ (4.0 equiv.), THF : H_2_O, rt, 2 h. f) Weinreb amine·HCl (3.0 equiv.), DIPEA (3.0 equiv.), Yb(OTf)_3_ (10 mol%), MeCN, 90 °C, 48 h. (g) MeMgBr (1.2 equiv.), THF, −78–0 °C, 1 h.

Finally, we explored the functionalization of the obtained products (Scheme 5). The oxazolidinone auxiliary was successfully replaced by a benzyl ester to afford enantioenriched 3a in 46% yield with preservation of the enantiomeric ratio (eqn (1)). Treatment of 3a with a slight excess of *m*CPBA furnished sulfone 5, a motif frequently encountered in medicinal chemistry, in 95% yield and without erosion of enantiopurity (eqn (2)).

Moreover, other ester derivatives were synthesized from enantioenriched 4a, including methyl ester 6 (95% yield and 97 : 3 er) (eqn (3)) and the *tert*-butyl ester 7, obtained *via* saponification followed by esterification in 42% yield and 97 : 3 er (eqn (4)). Enantioenriched alcohol 8 can be obtained in 92% yield and with unchanged er by reduction of the oxazolidinone auxiliary using NaBH_4_ (eqn (5)). Furthermore, the Weinreb amide analog 3z was obtained by reaction with the amine chloride salt in the presence of base and a catalytic amount of Yb(OTf)_3_ (eqn (6)) and was subsequently converted into the corresponding methyl ketone 9 in 91% yield without loss of er (eqn (7)).

## Conclusions

In summary, we have developed a diastereoselective sulfa-Michael addition using cyclobutene derivatives, enabling the synthesis of thio-substituted cyclobutane esters and amides. Furthermore, the enantioselective version of the reaction was achieved, delivering thio-cyclobutanes with high enantioselectivity using a chiral cinchona squaramide organocatalyst. The synthetic versatility of the obtained compounds was demonstrated through oxidation of the sulfur atom to sulfone and transformation of the ester functionality into an alcohol or a carboxylic acid. Finally, the oxazolidinone auxiliary was successfully converted into various esters, a Weinreb amide, and a ketone, all whilemaintaining enantiopurity.

## Author contributions

E. G. L. R. conceived the project, optimized the reaction, performed the investigation on the scope of the reaction, the modification of the products and prepared the experimental parts and first draft of the manuscript. J. W. supervised the project, edited the manuscript, and proofread the experimental part.

## Conflicts of interest

There are no conflicts to declare.

## Supplementary Material

SC-016-D5SC01727K-s001

SC-016-D5SC01727K-s002

## Data Availability

ESI† is available as a pdf file, including general methods, experimental procedures, compound characterization data and copies of NMR spectra for new compounds. Raw data for compound characterization will be available with free access at https://doi.org/10.5281/zenodo.15490161 after final publication of the work. The authors have cited additional references within the ESI.†^23–25^
