# Supplementary material for: Enantioselective synthesis of 1,2-disubstituted thiocyclobutanes via Michael addition
Source: Chem Sci. 2025 May 23;16(26):12115–21. doi: 10.1039/d5sc01727k (PMC12142855; doi:10.1039/d5sc01727k)
Supplement: SC-016-D5SC01727K-s001 [file SC-016-D5SC01727K-s001.pdf]

Supplementary Information (SI) for Chemical Science.
This journal is © The Royal Society of Chemistry 2025

Supporting Information for

Enantioselective Synthesis of 1,2-Disubstituted Thiocyclobutanes *via* Michael Addition

Emma G. L. Robert and Jérôme Waser*†

†Laboratory of Catalysis and Organic Synthesis, Institute of Chemical Sciences and Engineering, Ecole Polytechnique Fédérale de Lausanne, EPFL SB ISIC LCSO, BCH 4306, 1015 Lausanne, Switzerland. jerome.waser@epfl.ch

(125 pages)

Contents

1. Experimental part 3
   1.1. Synthesis of cyclobutenes 4
   1.2. Optimization of the racemic sulfa-Michael addition 10
   1.3. Racemic sulfa-Michael addition onto cyclobutenes 11
   1.4. Crossover experiment 27
   1.5. Optimization of the enantioselective sulfa-Michael addition 28
   1.6. Enantioselective sulfa-Michael addition onto cyclobutenes 31
   1.7. Product modifications 38
   1.8. Proposed model for stereoinduction 44
2. References 45
3. X-rays crystallographic data 46
   3.1 Compound **3'l** (CCDC number 2383043) 46
   3.2 Compound **4b** (CCDC number 2415842) 52
4. NMR Spectra 56
5. SFC traces 109

1. Experimental part

All reactions were carried out in oven dried glassware under an atmosphere of nitrogen, unless stated otherwise.

All chemicals were purchased from Acros, Aldrich, TCI, Merck, Fluorochem or Combi-Blocks and used as such.

Chromatographic purification was performed as flash chromatography using Macherey-Nagel silica 40-63, 60 Å, using the solvents indicated as eluent with 0.1-0.5 bar pressure. For flash chromatography, distilled technical grade solvents were used. TLC was performed on Merck silica gel 60 F254 TLC aluminum or glass plates and visualized with UV light and para-anysaldehyde stain.

^{1}H-NMR spectra were recorded on a Brucker DPX-400 400 MHz spectrometer in chloroform-*d* or DMSO-d_6, all signals are reported in ppm with the internal chloroform signal at 7.26 ppm or the internal DMSO signal at 2.50 ppm as standard. The data is being reported as (s = singlet, d = doublet, t= triplet, q = quadruplet, qi = quintet, m = multiplet or unresolved, br = broad signal, app = apparent, coupling constant(s) in Hz, integration, interpretation).^{13}C-NMR spectra were recorded with ^{1}H-decoupling on a Brucker DPX-400 100 MHz spectrometer in chloroform-*d* or DMSO-d_6 all signals are reported in ppm with the internal chloroform signal at 77.0 ppm or the internal DMSO signal at 39.52 ppm as standard.

Melting points were measured on a Büchi B-540 melting point apparatus using open glass capillaries and are uncorrected.

Infrared spectra were recorded on a JASCO FT-IR B4100 spectrophotometer with an ATR PRO410-S and a ZnSe prisma and are reported as cm^{-1} (w = weak, m = medium, s = strong, br = broad).

High resolution mass spectrometric measurements were performed by the mass spectrometry service of ISIC at the EPFL on a MICROMASS (ESI) Q-TOF Ultima API. A standard data acquisition and instrument control system was utilized (Thermo Scientific) whereas the ion source was controlled by Chipsoft 8.3.1 software (Advion BioScience). Samples were loaded onto a 96-well plate (Eppendorf, Hamburg, Germany) within an injection volume of 5µl. The experimental condition for the ionization voltage was +1.4kV and the gas pressure was set at 0.30 psi. The temperature of ion transfer capillary was 275 °C, tube voltages. FTMS spectra were obtained in the 80-1000 m/z range in the reduce profile mode with a resolution set to 120,000. In all spectra one microscan was acquired with a maximum injection time value of 1000ms. Typical CID experiments were carried out using Normalized collision energy values of 26-28 and 5 Da of isolation width.

SFC analysis on the chiral stationary phase were performed on an Agilent 1260 Infinity II instrument using columns CHIRALPAK IA-3 and IC-3. HPLC analysis on chiral stationary phase was performed on an Agilent Acquity instrument using a Daicel CHIRALPAK IA chiral column. The exact conditions for the analyses are specified within the characterization section. SFC traces were compared to racemic samples.

1.1. Synthesis of cyclobutenes

General procedure (A) for esterification or amidation of bromocyclobutane

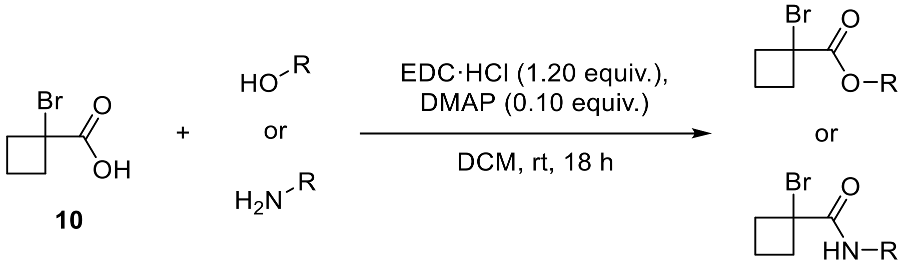

A solution of 1-bromocyclobutane-1-carboxylic acid **10** (1.00 equiv.), alcohol or amine (1.20 equiv.), EDC·HCl (1.20 equiv.) and DMAP (0.10 equiv.) in DCM was stirred at rt for 18 h. The mixture was then washed with water (1 x 100 mL), and the organic layer was dried over $MgSO_4$, filtered, and concentrated under reduced pressure. The crude bromocyclobutane was purified by flash chromatography.

1-Bromocyclobutanecarboxylic acid benzyl ester (11a)

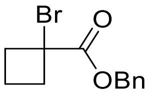

Prepared according to the general procedure A from 1-bromocyclobutane-1-carboxylic acid (150 mg, 838 µmol, 1.00 equiv.), benzylalcohol (109 mg, 1.01 mmol, 1.20 equiv.), EDC·HCl (193 mg, 1.01 mmol, 1.20 equiv.), DMAP (10.2 mg, 83.8 µmol, 0.100 equiv.), and DCM (4.0 mL). The crude product was purified by flash chromatography using PE/EtOAc 98:2 to afford **11a** as an oil (239 mg, 886 µmol, 79% yield).

Rf(PE/EtOAc 96:4): 0.71.
^{1}H NMR (400 MHz, chloroform-*d*) δ 7.50 – 7.30 (m, 5H, Ar*H*), 5.24 (s, 2H, OC*H*$_2$Ph), 3.05 – 2.84 (m, 2H, CC*H*$_2$), 2.72 – 2.52 (m, 2H, CC*H*$_2$), 2.33 – 2.10 (m, 1H, CH_2C*H*$_2$), 2.00 – 1.78 (m, 1H, CCH_2C*H*$_2$).
^{13}C NMR (101 MHz, chloroform-*d*) δ 171.5, 135.6, 128.7, 128.5, 128.1, 67.7, 54.3, 37.4, 16.9.
NMR spectra are in agreement with the reported data.[1]

1-Bromocyclobutanecarboxylic acid butyl ester (11b)

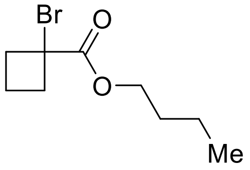

Prepared according to the general procedure A from 1-bromocyclobutane-1-carboxylic acid (895 mg, 5.00 mmol, 1.00 equiv.), 1-butanol (445 mg, 549 µL, 6.00 mmol, 1.20 equiv.), EDC·HCl (1.15 g, 6.00 mmol, 1.20 equiv.), DMAP (61.1 mg, 500 µmol, 0.100 equiv.), and DCM (12.1 mL). The crude product was purified by flash chromatography using PE/Et_2O 90:10 to afford **11b** as an oil (942 mg, 4.01 mmol, 80% yield).

Rf(PE/Et_2O 95:5): 0.50.

---

[1] E. G. L. Robert, V. Pirenne, M. D. Wodrich, J. Waser, *Angew. Chem. Int. Ed.* **2023**, *62*, e202302420.

^{1}H NMR (400 MHz, chloroform-*d*) δ 4.20 (t, *J* = 6.6 Hz, 2H, OC*H₂*), 2.99 – 2.79 (m, 2H, C(O)CC*H₂*), 2.62 (dddd, *J* = 13.9, 7.0, 5.0, 2.5 Hz, 2H, C(O)CC*H₂*), 2.22 (dtt, *J* = 11.5, 9.7, 5.9 Hz, 1H, C(O)CCH₂C*H₂*), 1.95 – 1.80 (m, 1H, C(O)CCH₂C*H₂*), 1.73 – 1.62 (m, 2H, OCH₂C*H₂*), 1.49 – 1.35 (m, 2H, OCH₂CH₂C*H₂*), 0.95 (t, *J* = 7.4 Hz, 3H, C*H₃*).
^{13}C NMR (101 MHz, chloroform-*d*) δ 171.8, 66.0, 54.6, 37.4, 30.6, 19.2, 16.9, 13.8.
NMR spectra are in agreement with the reported data.[2]

1-Bromanyl-N-methoxy-N-methyl-cyclobutane-1-carboxamide (11c)

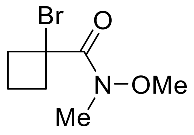

Prepared according to the general procedure A from 1-bromocyclobutane-1-carboxylic acid (300 mg, 1.68 mmol, 1.00 equiv.), methoxy(methyl)amine hydrochloride (196 mg, 2.01 mmol, 1.20 equiv.), EDC·HCl (386 mg, 2.01 mmol, 1.20 equiv.), DMAP (20.5 mg, 168 µmol, 0.100 equiv.), and DCM (8.0 mL). The crude product was purified by flash chromatography using PE/EtOAc 80:20 to afford **11c** as an oil (267 mg, 1.20 mmol, 72% yield).

Rf(PE/EtOAc 9:1): 0.40.
^{1}H NMR (400 MHz, chloroform-d) δ 3.77 (s, 3H, OC*H₃*), 3.23 (s, 3H, NC*H₃*), 2.96 – 2.85 (m, 2H, CC*H₂*), 2.57 – 2.45 (m, 2H, CC*H₂*), 2.43 – 2.28 (m, 1H, CCH₂C*H₂*), 1.86 – 1.69 (m, 1H, CCH₂C*H₂*).
^{13}C NMR (101 MHz, chloroform-d) δ 174.7, 60.6, 57.0, 37.3, 33.3, 16.2.
NMR spectra are in agreement with the reported data.[1]

Ethyl 4-(1-bromocyclobutane-1-carboxamido)benzoate (11d)

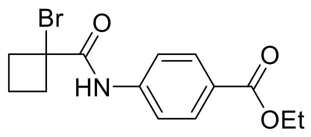

Prepared according to the general procedure A from 1-bromocyclobutane-1-carboxylic acid (716 mg, 4.00 mmol, 1.00 equiv.), ethyl 4-azanylbenzoate (793 mg, 4.80 mmol, 1.20 equiv.), EDC·HCl (920 mg, 4.80 mmol, 1.20 equiv.), DMAP (48.9 mg, 400 µmol, 0.100 equiv.), and DCM (9.71mL). The crude product was purified by flash chromatography using PE/EtOAc 90:10 to afford **11d** as a white solid (982 mg, 3.01 mmol, 75% yield).

Rf(PE/EtOAc 9:1): 0.29.
^{1}H NMR (400 MHz, chloroform-*d*) δ 8.17 (s, 1H, N*H*), 8.08 – 7.96 (m, 2H, Ar*H*), 7.70 – 7.59 (m, 2H, Ar*H*), 4.36 (q, *J* = 7.1 Hz, 2H, OC*H₂*), 3.10 (dtd, *J* = 8.8, 7.7, 5.0 Hz, 2H, CC*H₂*), 2.66 (dddd, *J* = 9.4, 7.3, 6.1, 3.3 Hz, 2H, CC*H₂*), 2.41 – 2.25 (m, 1H, CCH₂C*H₂*), 2.11 – 1.96 (m, 1H, CCH₂C*H₂*), 1.39 (t, *J* = 7.1 Hz, 3H, OCH₂C*H₃*).
^{13}C NMR (101 MHz, chloroform-*d*) δ 169.2, 166.2, 141.6, 131.0, 126.7, 119.0, 61.1, 60.5, 38.0, 17.2, 14.5.
NMR spectra are in agreement with the reported data.[3]

General procedure (B) for dehydrobromination

[2] A. A. Homon, O. V. Hryshchuk, S. Trofymchuk, O. Michurin, Y. Kuchkovska, D. S. Radchenko, O. O. Grygorenko, *Eur. J. Org. Chem.* **2018**, *2018*, 5596–5604.
[3] E. G. L. Robert, J. Waser, *Chem. Eur. J.* **2025**, *31*, e202403986.

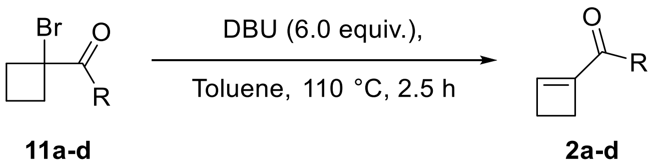

According to a reported procedure,[2] DBU (6.0 equiv.) in toluene was heated to 110 °C and the bromocyclobutane **11a-d** (1.00 equiv.) was added. The reaction was stirred at 110 °C for 2.5 h. White salts usually appear in suspension at the end of the reaction. The mixture was diluted in ether (100 mL) and then washed with water (1 x 100 mL), and the organic layer was dried over $MgSO_4$, filtered, and concentrated under reduced pressure. The crude cyclobutene was purified by flash chromatography.

Benzyl cyclobutene-1-carboxylate (2a)

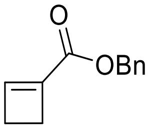

Prepared according to the general procedure B from **11a** (4.31 g, 3.20 mL, 16.0 mmol, 1.00 equiv.), DBU (14.6 g, 14.3 mL, 96.0 mmol, 6.00 equiv.) and toluene (50.3 mL). The crude product was purified by flash chromatography using PE/EtOAc 95:5 to afford **2a** as an oil (1.22 g, 6.47 mmol, 40% yield).

Rf(PE/EtOAc 95:5): 0.34.
^{1}H NMR (400 MHz, chloroform-*d*) δ 7.35 (dq, *J* = 16.7, 4.7 Hz, 5H, Ar*H*), 6.81 (s, 1H, C=C*H*), 5.18 (s, 2H, OCH_2Ph), 2.78 – 2.72 (m, 2H, CH_2CC(O)), 2.48 (t, *J* = 2.9 Hz, 2H, CH_2CH).
^{13}C NMR (101 MHz, chloroform-*d*, signals not fully resolved) δ 162.2, 147.2, 138.6, 136.2, 128.7, 128.3, 65.9, 29.3, 27.3.
NMR spectra are in agreement with the reported data.[4]

Cyclobutene-1-carboxylic acid butyl ester (2b)

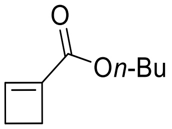

Prepared according to the general procedure B from **11b** (900 mg, 3.83 mmol, 1.00 equiv.), DBU (3.50 g, 3.43 mL, 23.0 mmol, 6.00 equiv.) and toluene (10.5 mL). The crude product was purified by flash chromatography using PE/EtOAc 95:5 to afford **2b** as an oil (331 mg, 2.14 mmol, 56% yield).

Rf(PE/Et_2O 95:5): 0.37.
^{1}H NMR (400 MHz, chloroform-*d*) δ 6.76 (s, 1H, *H*C=C), 4.13 (t, *J* = 6.7 Hz, 2H, OCH_2), 2.78 – 2.66 (m, 2H, CH_2CC(O)), 2.46 (dd, *J* = 4.4, 2.0 Hz, 2H, CH_2CH), 1.73 – 1.62 (m, 2H, OCH_2CH_2), 1.40 (h, *J* = 7.4 Hz, 2H, $OCH_2CH_2CH_2$), 0.94 (t, *J* = 7.4 Hz, 3H, CH_3).
^{13}C NMR (101 MHz, chloroform-*d*) δ 162.6, 146.3, 139.0, 64.1, 30.9, 29.3, 27.2, 19.3, 13.9.
NMR spectra are in agreement with the reported data.[2]

[4] H. Xu, W. Zhang, D. Shu, J. B. Werness, W. Tang, *Angew. Chem. Int. Ed.* **2008**, *47*, 8933–8936.

N-Methoxy-N-methylcyclobutene-1-carboxamide (2c)

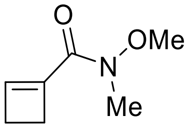

Prepared according to the general procedure B from **11c** (1.33 g, 6.00 mmol, 1.00 equiv.), DBU (5.48 g, 5.37 mL, 36.0 mmol, 6.00 equiv.) and toluene (15.6 mL). The crude product was purified by flash chromatography using PE/EtOAc 80:20 to afford **2c** as an oil (393 mg, 2.78 mmol, 46% yield).

Rf(PE/EtOAc 80:20): 0.53.
^{1}H NMR (400 MHz, chloroform-*d*) δ 6.66 (s, 1H, *H*C=C), 3.70 (s, 3H, OCH_3), 3.23 (s, 3H, NCH_3), 2.90 – 2.71 (m, 2H, CH_2CC(O)), 2.48 (dd, *J* = 4.6, 1.9 Hz, 2H, CH_2CH).
^{13}C NMR (101 MHz, chloroform-*d*) δ 162.9, 144.8, 139.5, 61.5, 32.7, 30.6, 27.7.
NMR spectra are in agreement with the reported data.[5]

Ethyl 4-(cyclobut-1-ene-1-carboxamido)benzoate (2d)

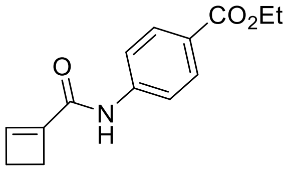

Prepared according to the general procedure B from **11d** (571 mg, 1.75 mmol, 1.00 equiv.), DBU (1.60 g, 1.57 mL, 10.5 mmol, 6.00 equiv.) and toluene (4.54 mL). The crude product was purified by flash chromatography using PE/EtOAc 70:30 to afford **2d** as a white solid (128 mg, 522 μmol, 30% yield).

m.p.: 154-155 °C.
Rf(PE/EtOAc 7:3): 0.61.
^{1}H NMR (400 MHz, chloroform-*d*) δ 8.02 (d, *J* = 8.8 Hz, 2H, Ar*H*), 7.72 – 7.51 (m, 2H, Ar*H*), 7.36 (s, 1H, N*H*), 6.79 (t, *J* = 1.2 Hz, 1H, *H*C=C), 4.36 (q, *J* = 7.1 Hz, 2H, OCH_2), 2.88 – 2.68 (m, 2H, C(O)CCH_2), 2.59 – 2.46 (m, 2H, CHCH_2), 1.39 (t, *J* = 7.1 Hz, 3H, OCH$_2$CH_3).
^{13}C NMR (101 MHz, chloroform-*d*) δ 166.2, 160.5, 142.6, 141.8, 141.6, 131.0, 126.2, 118.9, 61.0, 28.8, 26.4, 14.5.
IR (ν_{max}, cm^{-1}) 3354 (w), 2982 (w), 2932 (m), 2853 (w), 1719 (s), 1602 (m), 1526 (m), 1328 (m), 1274 (s), 1249 (s), 1166 (s), 1105 (m), 1024 (m), 770 (w), 739 (m).
HRMS (nanochip-ESI/LTQ-Orbitrap) m/z: $[M + Na]^+$ Calcd for $C_{14}H_{15}NNaO_3^+$ 268.0944; Found 268.0951.

3-(1-(Phenylthio)cyclobutane-1-carbonyl)oxazolidin-2-one (12)

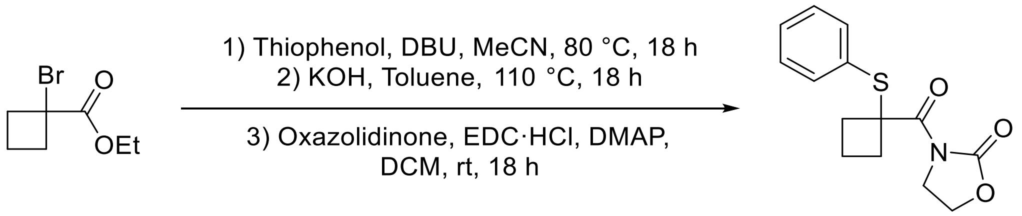

11f **12**

[5] J. M. Robinson, S. F. Tlais, J. Fong, R. L. Danheiser, *Tetrahedron* **2011**, *67*, 9890–9898.

According to a reported procedure,[6] commercially available 1-bromocyclobutanecarboxylic acid ethyl ester **11f** (12.4 g, 9.71 mL, 60.0 mmol, 1.00 equiv.) was dissolved in MeCN (60.0 mL). Thiophenol (6.61 g, 6.12 mL, 60.0 mmol, 1.00 equiv.) and DBU (13.7 g, 13.4 mL, 90.0 mmol, 1.50 equiv.) were then added. The mixture was stirred at 80 °C for 18 h. The crude mixture was diluted in EtOAc (200 mL) and washed with brine (200 mL), and the organic layer was dried over $MgSO_4$, filtered, and concentrated under reduced pressure. The resulting crude product was pure enough and taken to the next step without further purification.

KOH (13.5 g, 240 mmol, 4.00 equiv.) was heated in toluene (240 mL) at 110 °C and ethyl 1-(phenylthio)cyclobutane-1-carboxylate (14.2 g, 60.0 mmol, 1.00 equiv.) was added. The reaction was stirred at 110 °C for 18 h. The mixture was diluted in water (300 mL) and extracted with EtOAc (1 x 200 mL). The pH value of the aqueous layer was adjusted to 1 using HCl (1 M). The mixture was extracted with ethyl acetate (3 x 200 mL), dried over $MgSO_4$, filtered, and concentrated under reduced pressure. The crude white solid was pure enough and taken to the next step without further purification.

A solution of 1-(phenylthio)cyclobutanecarboxylic acid (12.5 g, 60.0 mmol, 1.00 equiv.), 2-oxazolidinone (6.27 g, 72.0 mmol, 1.20 equiv.), EDC·HCl (13.8 g, 72.0 mmol, 1.20 equiv.) and DMAP (733 mg, 6.00 mmol, 0.100 equiv.) in DCM (150 mL) was stirred at rt for 18 h. The mixture was then washed with water (1 x 200 mL), and the organic layer was dried over $MgSO_4$, filtered, and concentrated under reduced pressure. The crude product was purified by flash chromatography using PE/EtOAc 70:30 to afford **12** as a white solid (6.88 g, 24.8 mmol, 41% yield over 3 steps).

Rf(PE/EtOAc 7:3): 0.51.
^{1}H NMR (400 MHz, chloroform-*d*) δ 7.35 – 7.24 (m, 5H, Ar*H*), 4.47 (t, *J* = 8.1 Hz, 2H, OC*H$_2$*), 4.08 (t, *J* = 8.1 Hz, 2H, NC*H$_2$*), 2.79 – 2.63 (m, 2H, CC*H$_2$*), 2.45 – 2.30 (m, 2H, CC*H$_2$*), 2.30 – 2.13 (m, 1H, CCH$_2$C*H$_2$*), 1.81 (dtd, *J* = 15.9, 9.3, 4.7 Hz, 1H, CCH$_2$C*H$_2$*).
^{13}C NMR (101 MHz, chloroform-*d*) δ 173.5, 152.2, 133.5, 133.3, 128.9, 128.1, 62.5, 53.6, 43.7, 31.7, 15.4.
NMR spectra are in agreement with the reported data.[6]

3-(Cyclobut-1-ene-1-carbonyl)oxazolidin-2-one (2e)

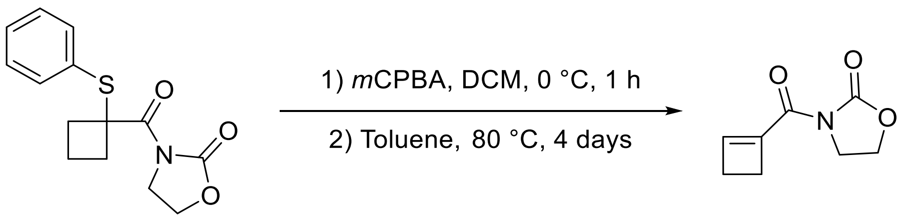

12 (6.88 g, 24.8 mmol, 1.00 equiv.) was diluted in DCM (163 mL). *m*CPBA (5.28 g, 23.6 mmol, 0.950 equiv.) dissolved in DCM (85.0 mL) was then added dropwise for 15 min at 0 °C. The reaction was stirred at 0 °C for 45 min. The reaction mixture was then diluted in DCM (100 mL) and washed with a sat. $NaHCO_3$ solution (200 mL). The combined organic layers were dried over $MgSO_4$, filtered, and concentrated under reduced pressure. The crude product was pure enough and taken to the next step without further purification.

The crude mixture was diluted in dry toluene (85.0 mL) under argon. The reaction was stirred at 80 °C for 4 days. The crude product was concentrated under reduced pressure and purified

[6] L. Ghisu, N. Melis, L. Serusi, A. Luridiana, F. Soddu, F. Secci, P. Caboni, R. Guillot, D. J. Aitken, A. Frongia, *Org. Biomol. Chem.* **2019**, *17*, 6143–6147.

by flash chromatography using PE/EtOAc 70:30 to afford **2e** as an oil (1.49 g, 8.92 mmol, 36% yield).

Rf(PE/EtOAc 8:2): 0.25.
^{1}H NMR (400 MHz, chloroform-*d*) δ 7.06 (d, *J* = 1.2 Hz, 1H, *H*C=C), 4.44 (t, *J* = 7.9 Hz, 2H, OCH_2), 4.05 (t, *J* = 8.0 Hz, 2H, NCH_2), 3.00 – 2.81 (m, 2H, CH_2C), 2.59 – 2.43 (m, 2H, $CHCH_2$).
^{13}C NMR (101 MHz, chloroform-*d*) δ 161.8, 153.0, 150.4, 138.0, 62.7, 43.3, 30.7, 27.9.
NMR spectra are in agreement with the reported data.[6]

1.2. Optimization of the racemic sulfa-Michael addition

General procedure for the optimization of the reaction

In a sealed vial under nitrogen, thiol **1a** and cyclobutene **2a** were diluted in 1 mL of solvent. DBU was then added. The reaction was stirred at the indicated temperature for the indicated time. The reaction mixture was concentrated under reduced pressure. The reaction mixture was analyzed by ^{1}H NMR (400 MHz, chloroform-*d*) using dibromomethane as an internal standard and by integration of the SC*H* proton at 4.19 (q, *J* = 8.3 Hz, 1H) ppm.

Optimization table

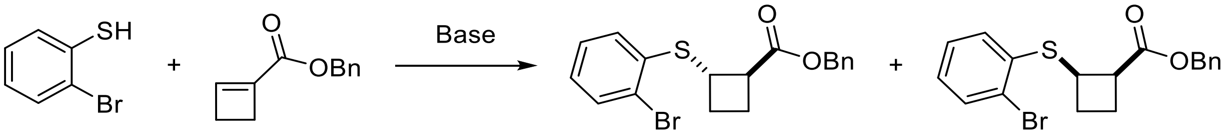

1a **2a** **3a** major **3'a** minor

Entry	Base	Solvent	T °C	Time	NMR Yield (%)[b]	dr [c]
1	DBU	MeCN	80 °C	18 h	82	>95:5
2	DBU	MeCN	rt	18 h	quant.	>95:5
3	NEt_3	MeCN	rt	18 h	95	51:49
4	TMG	MeCN	rt	18 h	90	64:36
5	TBD	MeCN	rt	18 h	69	72:28
6	K_2CO_3	MeCN	rt	18 h	72	50:50
7	DBU	EtOAc	rt	18 h	quant.	82:18
8	DBU	MeCN	rt	1 h	quant.	64:36
9	DBU	MeCN	rt	2 h	98	71:29
10	DBU	MeCN + air	rt	18 h	81	86:14
11	DBU	HPLC-MeCN	rt	18 h	97	89:11

[a]Reaction conditions: 1.0 equiv. thiol **1a** (0.1 mmol), 1.1 equiv. cyclobutene **2a**, 1.1 equiv. base. [b]1H NMR of the crude mixture with dibromomethane as an internal standard. [C]Measured on the crude ^{1}H NMR.

1.3. Racemic sulfa-Michael addition onto cyclobutenes

General procedure (C) for the racemic sulfa-Michael addition

In a sealed vial under nitrogen, thiol **1a-x** (0.3 mmol) and cyclobutene **2a-d** (1.1 equiv.) were diluted in 3 mL of MeCN. DBU (50.2 mg, 49.3 µL, 330 µmol, 1.10 equiv.) was then added. The reaction was stirred at rt for 18 h. The reaction mixture concentrated under reduced pressure and purified by flash chromatography. The mixture of diastereoisomers was isolated and the yield measured. For characterization purposes, a prep-TLC was then performed and only the major diastereoisomer was isolated and characterized, unless otherwise stated.

The dr was measured from the crude ^{1}H NMR spectra by integration of the SC*H* proton.

General procedure (D) for the racemic sulfa-Michael addition

In a sealed vial under nitrogen, thiol **1a-x** (0.3 mmol) and cyclobutene **2a-d** (1.1 equiv.) were diluted in 3 mL of MeCN. DBU (137 mg, 134 µL, 900 µmol, 3.00 equiv.) was then added. The reaction was stirred at 80 °C for 18 h. The reaction mixture concentrated under reduced pressure and purified by flash chromatography. The mixture of diastereoisomers was isolated and the yield measured. For characterization purposes, a prep-TLC was then performed and only the major diastereoisomer was isolated and characterized, unless otherwise stated.

The dr was measured from the crude ^{1}H NMR spectra by integration of the SC*H* proton.

Benzyl 2-((2-bromophenyl)thio)cyclobutane-1-carboxylate (3a)

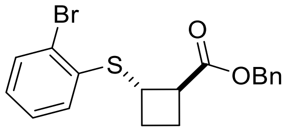

Prepared according to the general procedure C from 2-bromothiophenol (56.7 mg, 36.1 µL, 300 µmol, 1.00 equiv.) and cyclobutene **2a** (62.1 mg, 330 µmol, 1.10 equiv.). The crude product (dr >95:5) was purified by flash chromatography using PE/Et_2O 95:5 to afford **3a** and **3'a** as an oil (111 mg, 294 µmol, 94% yield).

A scale up experiment with 2-bromothiophenol (189 mg, 120 µL, 1.00 mmol, 1.00 equiv.), cyclobutene **2a** (207 mg, 1.10 mmol, 1.10 equiv.) and DBU (167 mg, 164 µL, 1.10 mmol, 1.10 equiv.) was also accomplished using the same procedure and led to **3a** (306 mg, 811 µmol, 81% yield, dr >95:5).

The configuration of the two diastereoisomers obtained was determined by 2D-NOESY.

Data for the trans- diastereoisomer:

Rf(PE/EtOAc 98:2): 0.58.
^{1}H NMR (400 MHz, chloroform-*d*) δ 7.52 (dd, *J* = 7.9, 1.3 Hz, 1H, Ar*H*), 7.38 – 7.28 (m, 5H, Ar*H*), 7.23 (dd, *J* = 7.9, 1.6 Hz, 1H, Ar*H*), 7.14 (td, *J* = 7.6, 1.3 Hz, 1H, Ar*H*), 7.00 (td, *J* = 7.6, 1.6 Hz, 1H, Ar*H*), 5.11 (s, 2H, OCH_2Ph), 4.19 (q, *J* = 8.3 Hz, 1H, SC*H*), 3.23 (q, *J* = 8.6 Hz, 1H, C*H*C(O)), 2.56 – 2.44 (m, 1H, SCHCH_2), 2.42 – 2.30 (m, 1H, CH_2CHC(O)), 2.23 (dq, *J* = 11.1, 8.9 Hz, 1H, CH_2CHC(O)), 2.08 (dq, *J* = 11.4, 9.2 Hz, 1H, SCHCH_2).
^{13}C NMR (101 MHz, chloroform-*d*) δ 173.3, 137.2, 135.9, 133.1, 129.5, 128.7, 128.4, 128.3, 127.9, 127.2, 123.8, 66.7, 46.1, 41.7, 27.0, 22.6.
IR ($ν_{max}$, cm^{-1}) 3036 (w), 2946 (m), 2863 (w), 1732 (s), 1705 (m), 1658 (m), 1577 (w), 1450 (m), 1386 (m), 1348 (m), 1253 (m), 1197 (s), 1160 (s), 1023 (m), 748 (s).
HRMS (Nanochip-based ESI/LTQ-Orbitrap) m/z: $[M + H]^+$ Calcd for $C_{18}H_{18}{}^{79}BrO_2S^+$ 377.0205; Found 377.0211.

Data for the cis- diastereoisomer:

Rf(PE/EtOAc 98:2): 0.41.
^{1}H NMR (400 MHz, chloroform-*d*) δ 7.48 (dd, *J* = 7.9, 1.3 Hz, 1H, Ar*H*), 7.30 – 7.08 (m, 7H, Ar*H*), 7.02 – 6.93 (m, 1H, Ar*H*), 5.06 (q, *J* = 12.2 Hz, 2H, OC*H*$_2$Ph), 4.26 (q, *J* = 8.5 Hz, 1H, SC*H*), 3.66 (tt, *J* = 5.5, 3.2 Hz, 1H, C*H*C(O)), 2.62 – 2.30 (m, 3H, SCHC*H*$_2$ + C*H*$_2$CHC(O)), 2.29 – 2.14 (m, 1H, C*H*$_2$CHC(O)).
^{13}C NMR (101 MHz, chloroform-*d*) δ 172.3, 138.6, 135.6, 132.8, 128.6, 128.5, 128.2, 127.9, 127.8, 126.5, 122.2, 66.9, 45.4, 42.0, 27.3, 21.2.
IR (ν_{max}, cm^{-1}) 3065 (w), 3033 (w), 2951 (m), 1730 (s), 1660 (w), 1447 (m), 1433 (m), 1382 (m), 1346 (m), 1242 (m), 1163 (s), 1022 (m), 748 (s).
HRMS (ESI/QTOF) m/z: [M + Na]$^+$ Calcd for $C_{18}H_{17}BrNaO_2S^+$ 399.0025; Found 399.0025.

Benzyl 2-((4-fluorophenyl)thio)cyclobutane-1-carboxylate (3b)

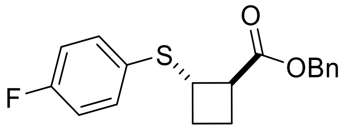

Prepared according to the general procedure C from 4-fluorobenzenethiol (38.5 mg, 32.3 µL, 300 µmol, 1.00 equiv.) and cyclobutene **2a** (62.1 mg, 330 µmol, 1.10 equiv.). The crude product (dr >95:5) was purified by flash chromatography using PE/Et$_2$O 98:2 to afford **3b** and **3'b** as an oil (83.4 mg, 264 µmol, 88% yield).

Data for the trans- diastereoisomer:

Rf(PE/EtOAc 98:2): 0.71.
^{1}H NMR (400 MHz, chloroform-*d*) δ 7.41 – 7.27 (m, 7H, Ar*H*), 7.02 – 6.86 (m, 2H, Ar*H*), 5.07 (s, 2H, OC*H*$_2$Ph), 3.97 (q, *J* = 8.6 Hz, 1H, SC*H*), 3.10 (q, *J* = 8.8 Hz, 1H, C*H*C(O)), 2.38 – 2.24 (m, 1H, SCHC*H*$_2$), 2.21 – 2.08 (m, 2H, C*H*$_2$CHC(O)), 2.04 – 1.86 (m, 1H, SCHC*H*$_2$).
^{13}C NMR (101 MHz, chloroform-*d*, signals not fully resolved) δ 173.1, 162.5 (d, *J* = 247.5 Hz), 135.9, 134.7 (d, *J* = 8.2 Hz), 128.7, 128.4, 128.3, 116.1 (d, *J* = 21.8 Hz), 66.6, 46.4, 44.3, 26.8, 21.9.
^{19}F NMR (376 MHz, chloroform-*d*) δ -114.2 (tt, *J* = 8.7, 5.3 Hz).
IR (ν_{max}, cm^{-1}) 3034 (w), 2989 (w), 2953 (w), 2114 (w), 1732 (s), 1589 (m), 1491 (s), 1455 (m), 1384 (m), 1350 (m), 1261 (s), 1224 (s), 1203 (s), 1159 (s), 1091 (m), 1033 (m), 917 (m), 824 (m), 744 (m).
HRMS (nanochip-ESI/LTQ-Orbitrap) m/z: [M + H]$^+$ Calcd for $C_{18}H_{18}FO_2S^+$ 317.1006; Found 317.1003.

Benzyl 2-((3-chlorophenyl)thio)cyclobutane-1-carboxylate (3c)

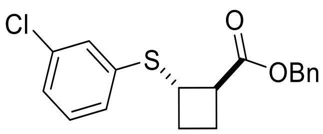

Prepared according to the general procedure C from 3-chlorobenzenethiol (43.4 mg, 34.4 µL, 300 µmol, 1.00 equiv.) and cyclobutene **2a** (62.1 mg, 330 µmol, 1.10 equiv.). The crude product (dr >95:5) was purified by flash chromatography using PE/Et$_2$O 95:5 to afford **3c** and **3'c** as an oil (81.9 mg, 246 µmol, 82% yield).

Data for the trans- diastereoisomer:

Rf(PE/EtOAc 98:2): 0.53.

^{1}H NMR (400 MHz, chloroform-*d*) δ 7.38 – 7.28 (m, 6H, Ar*H*), 7.20 – 7.13 (m, 3H, Ar*H*), 5.15 – 5.04 (m, 2H, OCH_2Ph), 4.10 (q, *J* = 8.5 Hz, 1H, SC*H*), 3.15 (q, *J* = 8.8 Hz, 1H, C*H*C(O)), 2.40 (qd, *J* = 8.5, 3.1 Hz, 1H, SCHCH_2), 2.30 – 2.12 (m, 2H, CH_2CHC(O)), 2.08 – 1.94 (m, 1H, SCHCH_2).
^{13}C NMR (101 MHz, chloroform-*d*) δ 173.1, 137.3, 135.9, 134.7, 130.1, 130.0, 128.7, 128.5, 128.4, 128.3, 126.9, 66.7, 46.5, 42.8, 27.2, 22.2.
IR (ν_{max}, cm^{-1}) 3035 (w), 2991 (w), 2944 (w), 1734 (s), 1579 (m), 1464 (m), 1385 (m), 1248 (m), 1198 (m), 1155 (s), 1083 (m), 1033 (m), 914 (m), 780 (s).
HRMS (nanochip-ESI/LTQ-Orbitrap) m/z: $[M + H]^+$ Calcd for $C_{18}H_{18}ClO_2S^+$ 333.0711; Found 333.0721.

Benzyl 2-((3-(trifluoromethyl)phenyl)thio)cyclobutane-1-carboxylate (3d)

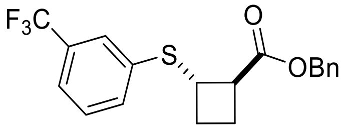

Prepared according to the general procedure C from 3-(trifluoromethyl)benzenethiol (53.5 mg, 40.8 µL, 300 µmol, 1.00 equiv.) and cyclobutene **2a** (62.1 mg, 330 µmol, 1.10 equiv.). The crude product (dr >95:5) was purified by flash chromatography using PE/Et_2O 95:5 to afford **3d** and **3’d** as an oil (97.9 mg, 267 µmol, 89% yield).

Data for the trans- diastereoisomer:

Rf(PE/EtOAc 98:2): 0.41.
^{1}H NMR (400 MHz, chloroform-*d*) δ 7.56 (s, 1H, Ar*H*), 7.44 (dd, *J* = 16.7, 7.8 Hz, 2H, Ar*H*), 7.39 – 7.27 (m, 6H, Ar*H*), 5.18 – 4.97 (m, 2H, OCH_2Ph), 4.15 (q, *J* = 8.6 Hz, 1H, SC*H*), 3.16 (qd, *J* = 9.2, 1.0 Hz, 1H, C*H*C(O)), 2.50 – 2.35 (m, 1H, SCHCH_2), 2.31 – 2.13 (m, 2H, CH_2CHC(O)), 2.03 (dq, *J* = 11.2, 9.4 Hz, 1H, SCHCH_2).
^{13}C NMR (101 MHz, chloroform-*d*) δ 173.1, 136.9, 135.8, 133.3, 131.4 (q, *J* = 32.3 Hz), 129.4, 128.7, 128.4, 128.3, 123.9 (q, *J* = 272.7 Hz), 123.3 (q, *J* = 3.7 Hz), 66.7, 46.5, 42.6, 27.2, 22.3.
^{19}F NMR (376 MHz, chloroform-*d*) δ -62.8.
IR (ν_{max}, cm^{-1}) 3044 (w), 2950 (w), 1728 (m), 1424 (w), 1321 (s), 1270 (m), 1166 (s), 1126 (s), 1073 (m), 908 (w), 798 (m), 738 (m).
HRMS (nanochip-ESI/LTQ-Orbitrap) m/z: $[M + H]^+$ Calcd for $C_{19}H_{18}F_3O_2S^+$ 367.0974; Found 367.0973.

Benzyl 2-((4-nitrophenyl)thio)cyclobutane-1-carboxylate (3e)

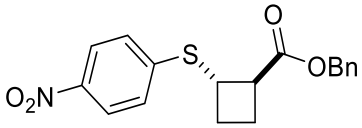

Prepared according to the general procedure C from 4-nitrobenzenethiol (46.6 mg, 300 µmol, 1.00 equiv.) and cyclobutene **2a** (62.1 mg, 330 µmol, 1.10 equiv.). The crude product (dr >95:5) was purified by flash chromatography using PE/EtOAc 90:10 to afford **3e** and **3’e** as an oil (60.1 mg, 175 µmol, 58% yield).

Data for the trans- diastereoisomer:

Rf(PE/EtOAc 95:5): 0.36.
^{1}H NMR (400 MHz, chloroform-*d*) δ 7.97 (d, *J* = 8.6 Hz, 2H, Ar*H*), 7.44 – 7.14 (m, 7H, Ar*H*), 5.22 – 4.94 (m, 2H, OCH_2Ph), 4.26 (q, *J* = 8.4 Hz, 1H, SC*H*), 3.20 (q, *J* = 8.8 Hz, 1H, C*H*C(O)),

2.56 – 2.45 (m, 1H, SCHC*H2*), 2.44 – 2.31 (m, 1H, C*H2*CHC(O)), 2.31 – 2.19 (m, 1H, C*H2*CHC(O)), 2.13 – 1.97 (m, 1H SCHC*H2*).
^{13}C NMR (101 MHz, chloroform-*d*) δ 173.0, 146.4, 145.4, 135.6, 128.8, 128.7, 128.5, 127.2, 124.1, 66.9, 46.6, 40.9, 27.0, 22.6.
IR (v_{max}, cm^{-1}) 3101 (w), 2991 (w), 2946 (w), 2363 (w), 2330 (w), 1728 (s), 1577 (m), 1511 (s), 1478 (w), 1451 (w), 1383 (w), 1337 (s), 1242 (m), 1199 (m), 1159 (m), 1093 (m), 1029 (w), 852 (m), 738 (m).
HRMS (nanochip-ESI/LTQ-Orbitrap) m/z: [M + H]$^+$ Calcd for $C_{18}H_{18}NO_4S^+$ 344.0951; Found 344.0947.

Methyl 2-((2-((benzyloxy)carbonyl)cyclobutyl)thio)benzoate (3f)

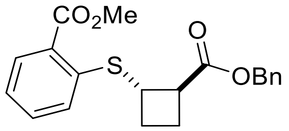

Prepared according to the general procedure C from 2-mercaptobenzoic acid methyl ester (50.5 mg, 41.4 µL, 300 µmol, 1.00 equiv.) and cyclobutene **2a** (62.1 mg, 330 µmol, 1.10 equiv.). The crude product (dr 86:14) was purified by flash chromatography using PE/EtOAc 85:15 to afford **3f** and **3'f** as an oil (106 mg, 299 µmol, quant.).

Prepared according to the general procedure D from 2-mercaptobenzoic acid methyl ester (50.5 mg, 41.4 µL, 300 µmol, 1.00 equiv.) and cyclobutene **2a** (62.1 mg, 330 µmol, 1.10 equiv.). The crude product (dr >95:5) was purified by flash chromatography using PE/EtOAc 85:15 to afford **3f** and **3'f** as an oil (74.9 mg, 210 µmol, 70% yield).

Data for the trans- diastereoisomer:

Rf(PE/EtOAc 8:2): 0.72.
^{1}H NMR (400 MHz, chloroform-*d*) δ 7.94 (dd, *J* = 7.8, 1.5 Hz, 1H, Ar*H*), 7.40 – 7.26 (m, 6H, Ar*H*), 7.20 (d, *J* = 7.4 Hz, 1H, Ar*H*), 7.16 – 7.08 (m, 1H, Ar*H*), 5.14 (s, 2H, OC*H2*Ph), 4.25 (q, *J* = 8.0 Hz, 1H, SC*H*), 3.90 (s, 3H, OC*H3*), 3.23 (q, *J* = 8.6 Hz, 1H, C*H*C(O)), 2.64 – 2.51 (m, 1H, SCHC*H2*), 2.48 – 2.35 (m, 1H, C*H2*CHC(O)), 2.27 (dq, *J* = 11.3, 8.9 Hz, 1H, C*H2*CHC(O)), 2.10 (dq, *J* = 11.5, 9.1 Hz, 1H, SCHC*H2*).
^{13}C NMR (101 MHz, chloroform-*d*) δ 173.6, 167.0, 141.2, 135.9, 132.5, 131.5, 128.7, 128.4, 128.3, 127.3, 126.6, 124.2, 66.7, 52.2, 45.6, 40.3, 27.1, 22.7.
IR (v_{max}, cm^{-1}) 2950 (w), 1717 (s), 1462 (m), 1436 (m), 1289 (m), 1246 (s), 1192 (m), 1146 (m), 1062 (s), 1044 (m), 743 (s).
HRMS (nanochip-ESI/LTQ-Orbitrap) m/z: [M + H]$^+$ Calcd for $C_{20}H_{21}O_4S^+$ 357.1155; Found 357.1156.

Benzyl 2-(phenylthio)cyclobutane-1-carboxylate (3g)

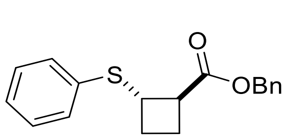

Prepared according to the general procedure C from benzenethiol (33.1 mg, 30.6 µL, 300 µmol, 1.00 equiv.) and cyclobutene **2a** (62.1 mg, 330 µmol, 1.10 equiv.). The crude product (dr 78:22) was purified by flash chromatography using PE/Et_2O 95:5 to afford **3g** and **3'g** as an oil (86.1 mg, 289 µmol, 96% yield).

Prepared according to the general procedure D from benzenethiol (33.1 mg, 30.6 µL, 300 µmol, 1.00 equiv.) and cyclobutene **2a** (62.1 mg, 330 µmol, 1.10 equiv.). The crude product

(dr >95:5) was purified by flash chromatography using PE/Et_2O 95:5 to afford **3g** and **3'g** as an oil (75.3 mg, 252 µmol, 84% yield).

Data for the trans- diastereoisomer:

Rf(PE/EtOAc 98:2): 0.46.
^{1}H NMR (400 MHz, chloroform-*d*) δ 7.33 (ddtt, *J* = 9.3, 7.2, 4.8, 2.1 Hz, 7H, Ar*H*), 7.25 – 7.17 (m, 3H, Ar*H*), 5.30 – 4.71 (m, 2H, OC*H₂*Ph), 4.08 (q, *J* = 8.5 Hz, 1H, SC*H*), 3.14 (q, *J* = 8.7 Hz, 1H, C*H*C(O)), 2.42 – 2.30 (m, 1H, SCHC*H₂*), 2.27 – 2.09 (m, 2H, C*H₂*CHC(O)), 2.00 (dq, *J* = 10.9, 9.4 Hz, 1H, SCHC*H₂*).
^{13}C NMR (101 MHz, chloroform-*d*) δ 173.3, 136.0, 134.7, 131.4, 129.0, 128.7, 128.4, 128.2, 127.0, 66.6, 46.4, 43.3, 27.2, 22.0.
IR ($ν_{max}$, cm^{-1}) 3062 (w), 2982 (w), 2949 (w), 1728 (s), 1585 (w), 1479 (m), 1441 (m), 1383 (m), 1348 (m), 1243 (s), 1195 (s), 1159 (s), 1091 (m), 1029 (m), 914 (m), 744 (s).
HRMS (nanochip-ESI/LTQ-Orbitrap) m/z: $[M + H]^+$ Calcd for $C_{18}H_{19}O_2S^+$ 299.1100; Found 299.1110.

Benzyl 2-((4-(tert-butyl)phenyl)thio)cyclobutane-1-carboxylate (3h)

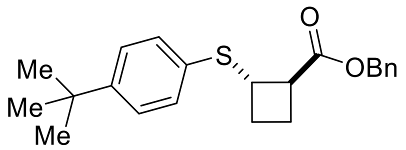

Prepared according to the general procedure C from 4-tert-butylbenzenethiol (49.9 mg, 50.4 µL, 300 µmol, 1.00 equiv.) and cyclobutene **2a** (62.1 mg, 330 µmol, 1.10 equiv.). The crude product (dr 84:16) was purified by flash chromatography using PE/Et_2O 98:2 to afford **3h** and **3'h** as an oil (80.9 mg, 228 µmol, 76% yield).

Prepared according to the general procedure D from 4-tert-butylbenzenethiol (49.9 mg, 50.4 µL, 300 µmol, 1.00 equiv.) and cyclobutene **2a** (62.1 mg, 330 µmol, 1.10 equiv.). The crude product (dr >95:5) was purified by flash chromatography using PE/Et_2O 98:2 to afford **3h** and **3'h** as an oil (95.9 mg, 271 µmol, 90% yield).

Data for the trans- diastereoisomer:

Rf(PE/EtOAc 98:2): 0.68.
^{1}H NMR (400 MHz, chloroform-*d*) δ 7.37 – 7.27 (m, 5H, Ar*H*), 7.25 (d, *J* = 8.5 Hz, 4H, Ar*H*), 5.15 – 5.00 (m, 2H, OC*H₂*Ph), 4.01 (q, *J* = 8.6 Hz, 1H, SC*H*), 3.18 – 3.05 (m, 1H, C*H*C(O)), 2.35 – 2.26 (m, 1H, SCHC*H₂*), 2.22 – 2.07 (m, 2H, C*H₂*CHC(O)), 1.97 (dq, *J* = 10.9, 9.4 Hz, 1H, SCHC*H₂*), 1.27 (s, 9H, $C(CH_3)_3$).
^{13}C NMR (101 MHz, chloroform-*d*) δ 173.3, 150.3, 136.0, 131.6, 130.9, 128.7, 128.3, 128.2, 126.1, 66.5, 46.4, 43.6, 34.6, 31.4, 27.1, 21.9.
IR ($ν_{max}$, cm^{-1}) 2961 (w), 2896 (w), 2872 (w), 1685 (m), 1614 (m), 1494 (m), 1407 (m), 1368 (m), 1182 (w), 1126 (w), 907 (s), 731 (s).
HRMS (Sicrit plasma/LTQ-Orbitrap) m/z: $[M + H]^+$ Calcd for $C_{22}H_{27}O_2S^+$ 355.1726; Found 355.1725.

Benzyl 2-((2,6-dimethylphenyl)thio)cyclobutane-1-carboxylate (3i)

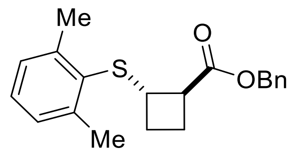

Prepared according to the general procedure C from 2,6-dimethylbenzenethiol (41.5 mg, 40.1 µL, 300 µmol, 1.00 equiv.) and cyclobutene **2a** (62.1 mg, 330 µmol, 1.10 equiv.). The crude product (dr 84:16) was purified by flash chromatography using PE/Et_2O 98:2 to afford **3i** and **3'i** as an oil (97.5 mg, 299 µmol, quant.).

Prepared according to the general procedure D from 2,6-dimethylbenzenethiol (41.5 mg, 40.1 µL, 300 µmol, 1.00 equiv.) and cyclobutene **2a** (62.1 mg, 330 µmol, 1.10 equiv.). The crude product (dr >95:5) was purified by flash chromatography using PE/Et_2O 98:2 to afford **3i** and **3'i** as an oil (91.0 mg, 279 µmol, 93% yield).

Data for the trans- diastereoisomer:

Rf(PE/EtOAc 98:2): 0.64.
^{1}H NMR (400 MHz, chloroform-*d*) δ 7.33 (td, *J* = 5.4, 2.7 Hz, 3H, Ar*H*), 7.29 – 7.22 (m, 2H, Ar*H*), 7.08 (q, *J* = 5.2 Hz, 3H, Ar*H*), 5.07 – 4.94 (m, 2H, OC*H$_2$*Ph), 3.81 – 3.67 (m, 1H, SC*H*), 3.16 – 3.03 (m, 1H, C*H*C(O)), 2.48 (s, 6H, ArC*H$_3$*), 2.27 – 2.17 (m, 1H, SCHC*H$_2$*), 2.14 – 1.92 (m, 3H, SCHC*H$_2$* + C*H$_2$*CHC(O)).
^{13}C NMR (101 MHz, chloroform-*d*) δ 173.1, 143.6, 136.0, 132.1, 128.6, 128.6, 128.3, 128.3, 128.2, 66.5, 47.0, 45.2, 27.7, 22.5, 22.0.
IR (ν_{max}, cm^{-1}) 3058 (w), 2987 (w), 2948 (w), 1732 (s), 1454 (m), 1382 (m), 1349 (m), 1253 (m), 1193 (m), 1159 (s), 1033 (m), 917 (m), 773 (s), 740 (m).
HRMS (Sicrit plasma/LTQ-Orbitrap) m/z: $[M + H]^+$ Calcd for $C_{20}H_{23}O_2S^+$ 327.1413; Found 327.1412.

Benzyl 2-((4-methyl-2-oxo-2H-chromen-7-yl)thio)cyclobutane-1-carboxylate (3j)

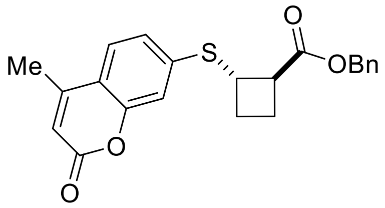

Prepared according to the general procedure C from 7-mercapto-4-methyl-coumarin (57.7 mg, 300 µmol, 1.00 equiv.) and cyclobutene **2a** (62.1 mg, 330 µmol, 1.10 equiv.). The crude product (dr 88:12) was purified by flash chromatography using PE/EtOAc 60:40 to afford **3j** and **3'j** as an oil (106 mg, 279 µmol, 93% yield).

Prepared according to the general procedure D from 7-mercapto-4-methyl-coumarin (57.7 mg, 300 µmol, 1.00 equiv.) and cyclobutene **2a** (62.1 mg, 330 µmol, 1.10 equiv.). The crude product (dr >95:5) was purified by flash chromatography using PE/EtOAc 60:40 to afford **3j** and **3'j** as an oil (37.7 mg, 99.1 µmol, 33% yield).

Data for the trans- diastereoisomer:

Rf(PE/EtOAc 6:4): 0.43.
^{1}H NMR (400 MHz, chloroform-*d*) δ 7.46 – 7.30 (m, 6H, Ar*H*), 7.18 (d, *J* = 1.8 Hz, 1H, Ar*H*), 7.13 (dd, *J* = 8.3, 1.8 Hz, 1H, Ar*H*), 6.21 (s, 1H, C=C*H*), 5.14 (s, 2H, OC*H$_2$*Ph), 4.24 (q, *J* = 8.4 Hz, 1H, SC*H*), 3.29 – 3.13 (m, 1H, C*H*C(O)), 2.59 – 2.45 (m, 1H, SCHC*H$_2$*), 2.41 – 2.20 (m, 5H, CH_3 + C*H$_2$*CHC(O)), 2.07 (dq, *J* = 11.4, 9.4 Hz, 1H, SCHC*H$_2$*).
^{13}C NMR (101 MHz, chloroform-*d*) δ 173.0, 160.7, 153.8, 152.1, 141.8, 135.8, 128.7, 128.4, 128.3, 124.8, 124.1, 117.8, 115.9, 114.4, 66.8, 46.3, 41.6, 27.3, 22.5, 18.7.
IR (ν_{max}, cm^{-1}) 3066 (w), 2950 (w), 2110 (w), 1727 (s), 1685 (w), 1602 (s), 1455 (w), 1386 (m), 1242 (w), 1202 (m), 1164 (m), 1029 (w), 961 (m), 852 (w), 752 (w).

HRMS (nanochip-ESI/LTQ-Orbitrap) m/z: $[M + H]^+$ Calcd for $C_{22}H_{21}O_4S^+$ 381.1155; Found 381.1152.

Benzyl 2-((3-methoxyphenyl)thio)cyclobutane-1-carboxylate (3k)

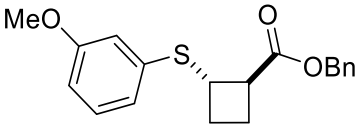

Prepared according to the general procedure C from 3-methoxybenzenethiol (42.1 mg, 37.2 µL, 300 µmol, 1.00 equiv.) and cyclobutene **2a** (62.1 mg, 330 µmol, 1.10 equiv.). The crude product (dr 86:14) was purified by flash chromatography using PE/Et_2O 95:5 to afford **3k** and **3'k** as an oil (98.4 mg, 299 µmol, quant.).

Prepared according to the general procedure D from 3-methoxybenzenethiol (42.1 mg, 37.2 µL, 300 µmol, 1.00 equiv.) and cyclobutene **2a** (62.1 mg, 330 µmol, 1.10 equiv.). The crude product (dr 89:11) was purified by flash chromatography using PE/Et_2O 95:5 to afford **3k** and **3'k** as an oil (98.1 mg, 299 µmol, quant. yield).

Data for the trans- diastereoisomer:

Rf(PE/EtOAc 98:2): 0.31.
^{1}H NMR (400 MHz, chloroform-*d*) δ 7.41 – 7.28 (m, 5H, Ar*H*), 7.16 (t, *J* = 7.9 Hz, 1H, Ar*H*), 6.95 – 6.87 (m, 2H, Ar*H*), 6.75 (ddd, *J* = 8.3, 2.5, 0.8 Hz, 1H, Ar*H*), 5.17 – 4.97 (m, 2H, OCH_2Ph), 4.11 (q, *J* = 8.5 Hz, 1H, SC*H*), 3.76 (s, 3H, OCH_3), 3.21 – 3.08 (m, 1H, C*H*C(O)), 2.44 – 2.32 (m, 1H, $SCHCH_2$), 2.19 (dtd, *J* = 17.9, 9.3, 2.3 Hz, 2H, $CH_2CHC(O)$), 2.01 (dq, *J* = 11.0, 9.4 Hz, 1H, $SCHCH_2$).
^{13}C NMR 13C NMR (101 MHz, $CDCl_3$) δ 173.3, 159.9, 136.0, 135.9, 129.8, 128.7, 128.3, 128.2, 123.2, 116.1, 112.9, 66.6, 55.4, 46.4, 43.0, 27.2, 22.1.
IR (v_{max}, cm^{-1}) 3065 (w), 2943 (w), 2835 (w), 1728 (m), 1589 (s), 1520 (w), 1480 (s), 1418 (s), 1383 (m), 1285 (s), 1242 (s), 1192 (m), 1156 (s), 1040 (s), 863 (m), 781 (s).
HRMS (nanochip-ESI/LTQ-Orbitrap) m/z: $[M + H]^+$ Calcd for $C_{19}H_{21}O_3S^+$ 329.1206; Found 329.1207.

Benzyl 2-((4-hydroxyphenyl)thio)cyclobutane-1-carboxylate (3l)

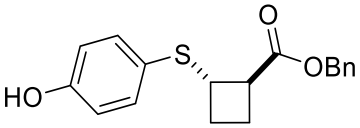

Prepared according to the general procedure C from 4-mercaptophenol (37.9 mg, 30.3 µL, 300 µmol, 1.00 equiv.) and cyclobutene **2a** (62.1 mg, 330 µmol, 1.10 equiv.). The crude product (dr 57:43) was purified by flash chromatography using PE/EtOAc 80:20 to afford **3l** and **3'l** as an oil (92.4 mg, 294 µmol, 98% yield).

Prepared according to the general procedure D from 4-mercaptophenol (37.9 mg, 30.3 µL, 300 µmol, 1.00 equiv.) and cyclobutene **2a** (62.1 mg, 330 µmol, 1.10 equiv.). The crude product (dr >95:5) was purified by flash chromatography using PE/EtOAc 80:20 to afford **3l** and **3'l** as an oil (64.3 mg, 205 µmol, 68% yield).

The configuration of the minor diastereoisomer obtained was determined by X-rays analysis.

Data for the trans- diastereoisomer:

Rf(PE/EtOAc 8:2): 0.43.

^{1}H NMR (400 MHz, chloroform-*d*, O*H* signal not resolved) δ 7.41 – 7.26 (m, 7H, Ar*H*), 6.77 – 6.69 (m, 2H, Ar*H*), 5.07 (d, *J* = 2.1 Hz, 2H, OC*H*$_2$Ph), 3.88 (q, *J* = 8.5 Hz, 1H, SC*H*), 3.07 (q, *J* = 8.9 Hz, 1H, C*H*C(O)), 2.28 – 2.17 (m, 1H, SCHC*H*$_2$), 2.09 (td, *J* = 9.3, 6.2 Hz, 2H, C*H*$_2$CHC(O)), 2.01 – 1.84 (m, 1H, SCHC*H*$_2$).
^{13}C NMR (101 MHz, chloroform-*d*) δ 173.4, 155.9, 136.0, 135.9, 128.7, 128.3, 128.2, 124.1, 116.1, 66.5, 46.1, 44.9, 26.6, 21.7.
IR (ν_{max}, cm^{-1}) 3405 (m), 2950 (m), 1732 (s), 1632 (m), 1602 (m), 1581 (m), 1497 (s), 1456 (m), 1433 (m), 1383 (m), 1350 (m), 1267 (s), 1216 (s), 1166 (s), 1029 (w), 845 (w), 744 (m).
HRMS (ESI/QTOF) m/z: [M + Na]$^+$ Calcd for $C_{18}H_{18}NaO_3S^+$ 337.0869; Found 337.0871.

Data for the cis- diastereoisomer:

m.p.: 96-98 °C.
Rf(PE/EtOAc 8:2): 0.30.
^{1}H NMR (400 MHz, chloroform-*d*) δ 7.32 (s, 5H, Ar*H*), 7.25 – 7.17 (m, 2H, Ar*H*), 6.76 – 6.66 (m, 2H, Ar*H*), 5.20 (d, *J* = 12.3 Hz, 1H, OC*H*$_2$Ph), 5.09 (d, *J* = 12.3 Hz, 1H, OC*H*$_2$Ph), 4.76 (s, 1H, O*H*), 4.06 (q, *J* = 8.4 Hz, 1H, SC*H*), 3.58 – 3.44 (m, 1H, C*H*C(O)), 2.49 – 2.22 (m, 3H, SCHC*H*$_2$ + C*H*$_2$CH(O)), 2.09 (dt, *J* = 10.7, 8.5 Hz, 1H, C*H*$_2$CH(O)).
^{13}C NMR (101 MHz, chloroform-*d*) δ 172.7, 155.0, 135.9, 133.1, 128.7, 128.6, 128.3, 127.0, 116.0, 66.7, 45.8, 45.3, 28.0, 20.8.
IR (ν_{max}, cm^{-1}) 3040 (m), 2953 (m), 2928 (m), 1728 (s), 1710 (s), 1605 (m), 1584 (m), 1496 (s), 1353 (m), 1263 (s), 1215 (s), 1173 (s), 1069 (m), 987 (s), 910 (s), 831 (m), 738 (m).
HRMS (ESI/QTOF) m/z: [M + Na]$^+$ Calcd for $C_{18}H_{18}NaO_3S^+$ 337.0869; Found 337.0871.

Benzyl 2-((4-acetamidophenyl)thio)cyclobutane-1-carboxylate (3m)

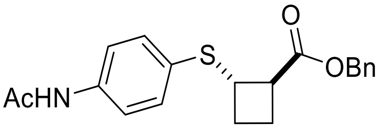

Prepared according to the general procedure C from N-(4-sulfanylphenyl)acetamide (50.2 mg, 300 µmol, 1.00 equiv.) and cyclobutene **2a** (62.1 mg, 330 µmol, 1.10 equiv.). The crude product (dr 75:25) was purified by flash chromatography using PE/EtOAc 70:30 to afford **3m** and **3'm** as an oil (101 mg, 285 µmol, 95% yield).

Prepared according to the general procedure D from N-(4-sulfanylphenyl)acetamide (50.2 mg, 300 µmol, 1.00 equiv.) and cyclobutene **2a** (62.1 mg, 330 µmol, 1.10 equiv.). The crude product (dr >95:5) was purified by flash chromatography using PE/EtOAc 70:30 to afford **3m** and **3'm** as an oil (93.9 mg, 264 µmol, 88% yield).

Data for the trans- diastereoisomer:

Rf(PE/EtOAc 7:3): 0.69.
^{1}H NMR (400 MHz, chloroform-*d*) δ 7.48 – 7.29 (m, 9H, Ar*H*), 7.19 (s, 1H, N*H*), 5.08 (q, *J* = 2.8 Hz, 2H, OC*H*$_2$Ph), 3.97 (q, *J* = 8.6 Hz, 1H, SC*H*), 3.09 (q, *J* = 8.9 Hz, 1H, C*H*C(O)), 2.29 (dtd, *J* = 11.3, 7.9, 3.4 Hz, 1H, SCHC*H*$_2$), 2.22 – 2.05 (m, 5H, C(O)C*H*$_3$ + C*H*$_2$CHC(O)), 2.02 – 1.88 (m, 1H, SCHC*H*$_2$).
^{13}C NMR (101 MHz, chloroform-*d*) δ 173.2, 168.3, 137.5, 136.0, 133.6, 129.0, 128.7, 128.3, 128.2, 120.2, 66.5, 46.2, 44.1, 26.9, 24.8, 21.9.
IR (ν_{max}, cm^{-1}) 3310 (m), 3101 (m), 2993 (m), 2946 (m), 2359 (w), 2104 (s), 1725 (s), 1671 (s), 1592 (s), 1527 (s), 1494 (s), 1455 (m), 1393 (s), 1372 (m), 1310 (s), 1252 (s), 1206 (s), 1158 (s), 1032 (m), 960 (m), 831 (m), 748 (s).

HRMS (nanochip-ESI/LTQ-Orbitrap) m/z: $[M + H]^+$ Calcd for $C_{20}H_{22}NO_3S^+$ 356.1315; Found 356.1312.

Benzyl 2-((2-aminophenyl)thio)cyclobutane-1-carboxylate (3n)

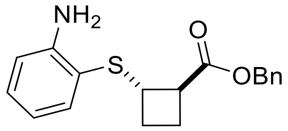

Prepared according to the general procedure C from 2-aminobenzenethiol (37.6 mg, 300 μmol, 1.00 equiv.) and cyclobutene **2a** (62.1 mg, 330 μmol, 1.10 equiv.). The crude product (dr 82:18) was purified by flash chromatography using PE/EtOAc 80:20 to afford **3n** and **3'n** as an oil (92.0 mg, 294 μmol, 98% yield).

Prepared according to the general procedure D from 2-aminobenzenethiol (37.6 mg, 300 μmol, 1.00 equiv.) and cyclobutene **2a** (62.1 mg, 330 μmol, 1.10 equiv.). The crude product (dr 94:6) was purified by flash chromatography using PE/EtOAc 80:20 to afford **3n** and **3'n** as an oil (82.5 mg, 263 μmol, 88% yield).

Data for the trans- diastereoisomer:

Rf(PE/EtOAc 8:2): 0.70.
^{1}H NMR (400 MHz, chloroform-*d*) δ 7.44 – 7.29 (m, 6H, Ar*H*), 7.14 (td, *J* = 8.0, 1.6 Hz, 1H, Ar*H*), 6.76 – 6.54 (m, 2H, Ar*H*), 5.09 (s, 2H, OCH_2Ph), 4.31 (br s, 2H, NH_2), 3.85 – 3.72 (m, 1H, SC*H*), 3.07 (q, *J* = 8.6 Hz, 1H, C*H*C(O)), 2.26 – 2.11 (m, 1H, SCHCH_2), 2.11 – 1.88 (m, 3H, CH_2CHC(O) + SCHCH_2).
^{13}C NMR (101 MHz, chloroform-*d*) δ 173.1, 149.5, 138.1, 136.1, 130.7, 128.7, 128.3, 128.2, 118.3, 115.1, 114.8, 66.5, 45.7, 44.5, 26.6, 21.6.
IR (ν_{max}, cm^{-1}) 3468 (w), 3357 (m), 3062 (m), 3029 (w), 2993 (m), 2950 (m), 1726 (s), 1607 (s), 1480 (s), 1448 (m), 1379 (w), 1348 (m), 1307 (m), 1242 (s), 1197 (s), 1159 (s), 1027 (m), 961 (w), 910 (w), 748 (s).
HRMS (nanochip-ESI/LTQ-Orbitrap) m/z: $[M + H]^+$ Calcd for $C_{18}H_{20}NO_2S^+$ 314.1209; Found 314.1224.

Benzyl 2-(pyridin-2-ylthio)cyclobutane-1-carboxylate (3o)

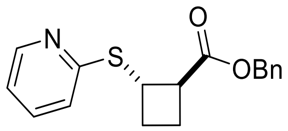

Prepared according to the general procedure C from 2-pyridinethiol (33.3 mg, 300 μmol, 1.00 equiv.) and cyclobutene **2a** (62.1 mg, 330 μmol, 1.10 equiv.). The crude product (dr 81:19) was purified by flash chromatography using PE/Et_2O 98:2 to afford **3o** and **3'o** as an oil (71.1 mg, 237 μmol, 79% yield).

Prepared according to the general procedure D from 2-pyridinethiol (33.3 mg, 300 μmol, 1.00 equiv.) and cyclobutene **2a** (62.1 mg, 330 μmol, 1.10 equiv.). The crude product (dr >95:5) was purified by flash chromatography using PE/Et_2O 98:2 to afford **3o** and **3'o** as an oil (63.0 mg, 210 μmol, 70% yield).

Data for the trans- diastereoisomer:

Rf(PE/EtOAc 98:2): 0.53.

^{1}H NMR (400 MHz, chloroform-*d*) δ 8.30 – 8.21 (m, 1H, Ar*H*), 7.42 (td, *J* = 7.7, 1.9 Hz, 1H, Ar*H*), 7.36 – 7.27 (m, 5H, Ar*H*), 7.11 (d, *J* = 8.1 Hz, 1H, Ar*H*), 6.92 (ddd, *J* = 7.4, 4.9, 1.0 Hz, 1H, Ar*H*), 5.19 – 5.07 (m, 2H, OC*H*$_2$Ph), 4.62 (q, *J* = 8.6 Hz, 1H, SC*H*), 3.33 – 3.21 (m, 1H, C*H*C(O)), 2.54 – 2.39 (m, 1H, SCHC*H*$_2$), 2.32 (td, *J* = 9.3, 6.3 Hz, 2H, C*H*$_2$CHC(O)), 2.19 – 2.04 (m, 1H, SCHC*H*$_2$).
^{13}C NMR (101 MHz, chloroform-*d*) δ 173.5, 159.1, 149.5, 136.1, 136.1, 128.6, 128.2, 128.2, 122.1, 119.6, 66.5, 47.2, 40.1, 26.7, 22.7.
IR (ν_{max}, cm^{-1}) 3041 (w), 2979 (w), 2946 (w), 1728 (s), 1578 (s), 1559 (m), 1453 (s), 1415 (s), 1383 (m), 1348 (m), 1267 (m), 1242 (m), 1191 (s), 1155 (s), 1127 (s), 1029 (m), 759 (s).
HRMS (ESI/QTOF) m/z: [M + Na]$^+$ Calcd for $C_{17}H_{17}NNaO_2S^+$ 322.0872; Found 322.0861.

Benzyl 2-((2-methylfuran-3-yl)thio)cyclobutane-1-carboxylate (3p)

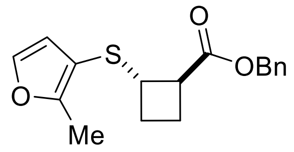

Prepared according to the general procedure C from 2-methyl-3-furanthiol (34.2 mg, 32.9 µL, 300 µmol, 1.00 equiv.) and cyclobutene **2a** (62.1 mg, 330 µmol, 1.10 equiv.). The crude product (dr 76:24) was purified by flash chromatography using PE/Et$_2$O 95:5 to afford **3p** and **3'p** as an oil (88.6 mg, 293 µmol, 98% yield).

Prepared according to the general procedure D from 2-methyl-3-furanthiol (34.2 mg, 32.9 µL, 300 µmol, 1.00 equiv.) and cyclobutene **2a** (62.1 mg, 330 µmol, 1.10 equiv.). The crude product (dr >95:5) was purified by flash chromatography using PE/Et$_2$O 95:5 to afford **3p** and **3'p** as an oil (69.2 mg, 229 µmol, 76% yield).

Data for the trans- diastereoisomer:

Rf(PE/EtOAc 98:2): 0.44.
^{1}H NMR (400 MHz, chloroform-*d*) δ 7.35 (dtt, *J* = 11.2, 7.4, 3.8 Hz, 5H, Ar*H*), 7.26 (d, *J* = 1.4 Hz, 1H, Ar*H*), 6.32 (d, *J* = 1.9 Hz, 1H, Ar*H*), 5.10 (s, 2H, OC*H*$_2$Ph), 3.69 (q, *J* = 8.8 Hz, 1H, SC*H*), 3.00 (q, *J* = 8.8 Hz, 1H, C*H*C(O)), 2.30 (s, 3H, C*H*$_3$), 2.21 – 2.10 (m, 1H, SCHC*H*$_2$), 2.10 – 1.99 (m, 2H, C*H*$_2$CHC(O)), 1.96 – 1.84 (m, 1H, SCHC*H*$_2$).
^{13}C NMR (101 MHz, chloroform-*d*) δ 173.1, 156.8, 140.7, 136.1, 128.7, 128.3, 128.2, 116.4, 107.6, 66.5, 45.7, 44.8, 26.2, 21.5, 12.0.
IR (ν_{max}, cm^{-1}) 2993 (w), 2946 (w), 2925 (w), 1732 (s), 1516 (w), 1451 (w), 1384 (w), 1348 (w), 1241 (m), 1191 (m), 1159 (s), 1091 (m), 1033 (w), 936 (w), 738 (m).
HRMS (nanochip-ESI/LTQ-Orbitrap) m/z: [M + H]$^+$ Calcd for $C_{17}H_{19}O_3S^+$ 303.1049; Found 303.1063.

Benzyl 2-(benzylthio)cyclobutane-1-carboxylate (3q)

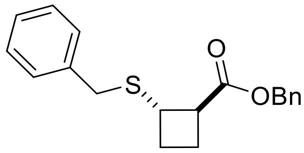

Prepared according to the general procedure C from phenylmethanethiol (37.3 mg, 35.2 µL, 300 µmol, 1.00 equiv.) and cyclobutene **2a** (62.1 mg, 330 µmol, 1.10 equiv.). The crude product (dr 50:50) was purified by flash chromatography using PE/Et$_2$O 98:2 to afford **3q** and **3'q** as an oil (93.4 mg, 299 µmol, quant.).

Prepared according to the general procedure D from phenylmethanethiol (37.3 mg, 35.2 µL, 300 µmol, 1.00 equiv.) and cyclobutene **2a** (62.1 mg, 330 µmol, 1.10 equiv.). The crude product (dr 93:7) was purified by flash chromatography using PE/Et_2O 98:2 to afford **3q** and **3'q** as an oil (93.3 mg, 299 µmol, quant.).

Data for the trans- diastereoisomer:

Rf(PE/EtOAc 98:2): 0.45.
^{1}H NMR (400 MHz, chloroform-*d*) δ 7.35 (q, *J* = 3.6 Hz, 5H, Ar*H*), 7.28 – 7.19 (m, 5H, Ar*H*), 5.11 (s, 2H, OCH_2Ph), 3.79 – 3.56 (m, 3H, SCH_2Ph + SC*H*), 3.13 (q, *J* = 8.7 Hz, 1H, C*H*C(O)), 2.30 – 2.01 (m, 3H, SCHCH_2 + CH_2CHC(O)), 1.97 – 1.78 (m, 1H, SCHCH_2).
^{13}C NMR (101 MHz, chloroform-*d*) δ 173.5, 138.6, 136.0, 128.9, 128.7, 128.6, 128.4, 128.3, 127.1, 66.6, 47.6, 41.2, 36.0, 26.7, 22.0.
IR (ν_{max}, cm^{-1}) 3062 (w), 3033 (w), 2950 (w), 1732 (s), 1497 (w), 1455 (m), 1382 (w), 1350 (m), 1242 (m), 1199 (m), 1159 (s), 1074 (w), 1033 (w), 957 (w), 911 (w).
HRMS (ESI/QTOF) m/z: [M + Na]$^+$ Calcd for $C_{19}H_{20}NaO_2S^+$ 335.1076; Found 335.1085.

Benzyl 2-((4-methoxybenzyl)thio)cyclobutane-1-carboxylate (3r)

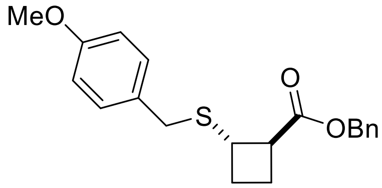

Prepared according to the general procedure D from (4-methoxyphenyl)methanethiol (46.3 mg, 41.3 µL, 300 µmol, 1.00 equiv.) and cyclobutene **2a** (62.1 mg, 330 µmol, 1.10 equiv.). The crude product (dr 92:8) was purified by flash chromatography using PE/Et_2O 95:5 to afford **3r** and **3'r** as an oil (98.8 mg, 289 µmol, 96% yield).

Data for the trans- diastereoisomer:

Rf(PE/EtOAc 98:2): 0.29.
^{1}H NMR (400 MHz, chloroform-*d*) δ 7.34 (d, *J* = 4.3 Hz, 5H, Ar*H*), 7.16 (d, *J* = 8.6 Hz, 2H, Ar*H*), 6.87 – 6.75 (m, 2H, Ar*H*), 5.12 (s, 2H, OCH_2), 3.77 (s, 3H, OCH_3), 3.72 – 3.50 (m, 3H, SCH_2 + SC*H*), 3.13 (q, *J* = 8.7 Hz, 1H, C*H*C(O)), 2.26 – 2.04 (m, 3H, SCHCH_2 + CH_2CHC(O)), 1.96 – 1.80 (m, 1H, SCHCH_2).
^{13}C NMR (101 MHz, chloroform-*d*) δ 173.6, 158.7, 136.0, 130.5, 130.0, 128.7, 128.4, 128.3, 114.0, 66.5, 55.4, 47.6, 41.1, 35.3, 26.6, 22.0.
IR (ν_{max}, cm^{-1}) 2996 (w), 2950 (m), 2838 (w), 1730 (s), 1613 (w), 1512 (s), 1455 (w), 1248 (s), 1177 (m), 1156 (m), 1035 (m), 831 (w), 744 (m).
HRMS (ESI/QTOF) m/z: [M + Na]$^+$ Calcd for $C_{20}H_{22}NaO_3S^+$ 365.1182; Found 365.1189.

Benzyl 2-((4-chlorobenzyl)thio)cyclobutane-1-carboxylate (3s)

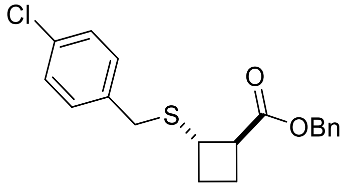

Prepared according to the general procedure D from (4-chlorophenyl)methanethiol (47.6 mg, 39.6 µL, 300 µmol, 1.00 equiv.) and cyclobutene **2a** (62.1 mg, 330 µmol, 1.10 equiv.). The

crude product (dr >95:5) was purified by flash chromatography using PE/EtOAc 95:5 to afford **3s** and **3's** as an oil (92.7 mg, 267 μmol, 89% yield).

Data for the trans- diastereoisomer:

Rf(PE/EtOAc 95:5): 0.68.
^{1}H NMR (400 MHz, chloroform-*d*) δ 7.40 – 7.29 (m, 5H, Ar*H*), 7.25 – 7.13 (m, 4H, Ar*H*), 5.17 – 5.02 (m, 2H, OCH_2), 3.77 – 3.43 (m, 3H, SC*H* + SCH_2), 3.18 – 3.04 (m, 1H, C*H*C(O)), 2.29 – 2.03 (m, 3H, SCHCH_2 + CH_2CHC(O)), 2.00 – 1.79 (m, 1H, SCHCH_2).
^{13}C NMR (101 MHz, chloroform-*d*) δ 173.5, 137.2, 135.9, 132.8, 130.2, 128.8, 128.7, 128.5, 128.4, 66.6, 47.7, 41.1, 35.3, 26.5, 22.0.
IR (ν_{max}, cm^{-1}) 3040 (w), 2984 (w), 2953 (w), 2872 (w), 1730 (s), 1492 (m), 1455 (w), 1382 (w), 1350 (w), 1242 (m), 1203 (m), 1159 (m), 1094 (m), 1015 (w), 910 (w), 830 (w), 748 (m).
HRMS (Nanochip-based ESI/LTQ-Orbitrap) m/z: [M + H]$^+$ Calcd for $C_{19}H_{20}ClO_2S^+$ 347.0867; Found 347.0868.

Benzyl 2-((furan-2-ylmethyl)thio)cyclobutane-1-carboxylate (3t)

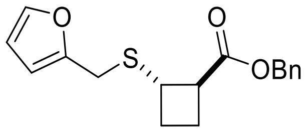

Prepared according to the general procedure D from 2-furylmethanethiol (34.2 mg, 30.3 μL, 300 μmol, 1.00 equiv.) and cyclobutene **2a** (62.1 mg, 330 μmol, 1.10 equiv.). The crude product (dr 94:6) was purified by flash chromatography using PE/Et_2O 95:5 to afford **3t** and **3't** as an oil (72.7 mg, 240 μmol, 80% yield).

Data for the trans- diastereoisomer:

Rf(PE/EtOAc 98:2): 0.51.
^{1}H NMR (400 MHz, chloroform-*d*) δ 7.43 – 7.29 (m, 6H, Ar*H*), 6.26 (dd, *J* = 3.0, 2.0 Hz, 1H, Ar*H*), 6.09 (d, *J* = 3.2 Hz, 1H, Ar*H*), 5.14 (s, 2H, OCH_2), 3.83 – 3.63 (m, 3H, SCH_2 + SC*H*), 3.14 (q, *J* = 8.7 Hz, 1H, C*H*C(O)), 2.30 – 2.07 (m, 3H, SCHCH_2 + CH_2CHC(O)), 1.92 (qd, *J* = 10.0, 1.4 Hz, 1H, SCHCH_2).
^{13}C NMR (101 MHz, chloroform-*d*) δ 173.4, 151.7, 142.1, 135.9, 128.7, 128.4, 128.3, 110.5, 107.4, 66.5, 47.6, 41.2, 28.0, 26.4, 21.9.
IR (ν_{max}, cm^{-1}) 3115 (w), 3033 (w), 2989 (w), 2950 (w), 2871 (w), 1727 (s), 1501 (m), 1455 (w), 1389 (w), 1350 (m), 1245 (s), 1192 (m), 1154 (s), 1076 (w), 1030 (m), 1011 (m), 935 (m), 737 (s).
HRMS (ESI/QTOF) m/z: [M + Na]$^+$ Calcd for $C_{17}H_{18}NaO_3S^+$ 325.0869; Found 325.0875.

Benzyl 2-(phenethylthio)cyclobutane-1-carboxylate (3u)

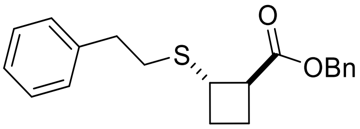

Prepared according to the general procedure C from 2-phenylethanethiol (41.5 mg, 40.3 μL, 300 μmol, 1.00 equiv.) and cyclobutene **2a** (62.1 mg, 330 μmol, 1.10 equiv.). The crude product (dr 50:50) was purified by flash chromatography using PE/Et_2O 95:5 to afford **3u** and **3'u** as an oil (97.5 mg, 299 μmol, 100% yield, quant.).

Prepared according to the general procedure D from 2-phenylethanethiol (41.5 mg, 40.3 μL, 300 μmol, 1.00 equiv.) and cyclobutene **2a** (62.1 mg, 330 μmol, 1.10 equiv.). The crude product

(dr >95:5) was purified by flash chromatography using PE/Et_2O 95:5 to afford **3u** and **3'u** as an oil (97.8 mg, 299 µmol, quant.).

Data for the trans- diastereoisomer:

Rf(PE/EtOAc 98:2): 0.44.
^{1}H NMR (400 MHz, chloroform-*d*) δ 7.38 – 7.25 (m, 7H, Ar*H*), 7.23 – 7.08 (m, 3H, Ar*H*), 5.13 (s, 2H, OCH_2Ph), 3.73 (q, *J* = 8.4 Hz, 1H, SC*H*), 3.12 (q, *J* = 9.0 Hz, 1H, C*H*C(O)), 2.89 – 2.70 (m, 4H, SCH_2CH_2Ph), 2.32 – 2.08 (m, 3H, SCHCH_2 + CH_2CHC(O)), 1.93 (p, *J* = 9.8 Hz, 1H, SCHCH_2).
^{13}C NMR (101 MHz, chloroform-*d*) δ 173.6, 140.6, 136.0, 128.7, 128.6, 128.6, 128.4, 128.4, 126.5, 66.6, 47.7, 41.7, 36.6, 33.0, 26.8, 22.0.
IR (ν_{max}, cm^{-1}) 3062 (w), 3028 (m), 2993 (w), 2870 (m), 2835 (w), 1725 (s), 1496 (m), 1382 (m), 1361 (m), 1329 (m), 1260 (s), 1243 (s), 1195 (s), 1087 (m), 1029 (m), 964 (m), 738 (m).
HRMS (ESI/QTOF) m/z: [M + Na]$^+$ Calcd for $C_{20}H_{22}NaO_2S^+$ 349.1233; Found 349.1232.

Benzyl 2-(cyclohexylthio)cyclobutane-1-carboxylate (3v)

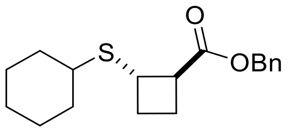

Prepared according to the general procedure D from cyclohexanethiol (34.9 mg, 36.7 µL, 300 µmol, 1.00 equiv.) and cyclobutene **2a** (62.1 mg, 330 µmol, 1.10 equiv.). The crude product (dr 93.7) was purified by flash chromatography using PE/Et_2O 95:5 to afford **3v** and **3'v** as an oil (90.9 mg, 299 µmol, quant.).

Data for the trans- diastereoisomer:

Rf(PE/EtOAc 98:2): 0.64.
^{1}H NMR (400 MHz, chloroform-*d*) δ 7.40 – 7.29 (m, 5H, Ar*H*), 5.13 (d, *J* = 1.8 Hz, 2H, OCH_2), 3.75 (q, *J* = 8.7 Hz, 1H, SC*H*CHC(O)), 3.09 (q, *J* = 8.5 Hz, 1H, C*H*C(O)), 2.66 (ddt, *J* = 10.5, 7.5, 3.7 Hz, 1H, SC$H^{cyclohex}$), 2.34 – 2.07 (m, 3H, SCHCH_2CH_2CHC(O)), 1.99 – 1.79 (m, 3H, SCHCH_2CH_2CHC(O) + C$H_2^{cyclohex}$), 1.74 – 1.64 (m, 2H, C$H_2^{cyclohex}$), 1.54 (d, *J* = 8.8 Hz, 1H, C$H_2^{cyclohex}$), 1.37 – 1.06 (m, 5H, C$H_2^{cyclohex}$).
^{13}C NMR (101 MHz, chloroform-*d*) δ 173.7, 136.1, 128.7, 128.4, 128.3, 66.5, 48.2, 44.0, 40.2, 34.2, 34.0, 27.8, 26.2, 26.1, 25.8, 22.1.
IR (ν_{max}, cm^{-1}) 3033 (w), 2932 (s), 2856 (m), 1732 (s), 1654 (w), 1584 (w), 1450 (m), 1343 (m), 1249 (m), 1190 (s), 1155 (s), 1083 (w), 1029 (m), 909 (w), 744 (m).
HRMS (ESI/QTOF) m/z: [M + Na]$^+$ Calcd for $C_{18}H_{24}NaO_2S^+$ 327.1389; Found 327.1389.

Benzyl 2-((adamantan-1-yl)thio)cyclobutane-1-carboxylate (3w)

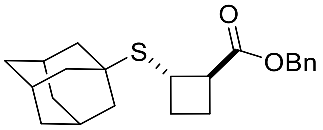

Prepared according to the general procedure C from adamantane-1-thiol (50.5 mg, 300 µmol, 1.00 equiv.) and cyclobutene **2a** (62.1 mg, 330 µmol, 1.10 equiv.). The crude product (dr 29:71) was purified by flash chromatography using PE/Et_2O 95:5 to afford **3w** and **3'w** as an oil (107 mg, 299 µmol, quant.).

Prepared according to the general procedure D from adamantane-1-thiol (50.5 mg, 300 µmol, 1.00 equiv.) and cyclobutene **2a** (62.1 mg, 330 µmol, 1.10 equiv.). The crude product (dr 92:8)

was purified by flash chromatography using PE/Et_2O 95:5 to afford **3w** and **3'w** as an oil (100 mg, 281 μmol, 94% yield).

The configuration of the two diastereoisomers obtained was determined by 2D-NOESY.

<u>*Data for the trans- diastereoisomer:*</u>

Rf(PE/EtOAc 98:2): 0.44.
^{1}H NMR (400 MHz, chloroform-*d*) δ 7.46 – 7.29 (m, 5H, Ar*H*), 5.24 – 5.02 (m, 2H, OCH_2Ph), 3.84 (q, *J* = 8.8 Hz, 1H, SC*H*), 3.06 (q, *J* = 9.0 Hz, 1H, C*H*C(O)), 2.30 (qd, *J* = 8.8, 2.7 Hz, 1H, SCHCH_2), 2.22 – 2.04 (m, 2H, CH_2CHC(O)), 2.02 – 1.85 (m, 4H, SCHCH_2 + SCCH_2C*H*), 1.79 (d, *J* = 2.4 Hz, 6H, SCCH_2), 1.63 (q, *J* = 12.3 Hz, 6H, SCCH_2CHCH_2).
^{13}C NMR (101 MHz, chloroform-*d*) δ 173.7, 136.1, 128.7, 128.36, 128.35, 66.5, 48.2, 45.9, 43.9, 36.6, 36.3, 29.8, 29.6, 22.6.
IR (ν_{max}, cm^{-1}) 2907 (s), 2849 (m), 1732 (s), 1452 (m), 1347 (m), 1254 (m), 1192 (m), 1152 (s), 1040 (m), 741 (m).
HRMS (nanochip-ESI/LTQ-Orbitrap) m/z: $[M + H]^+$ Calcd for $C_{22}H_{29}O_2S^+$ 357.1883; Found 357.1876

<u>*Data for the cis- diastereoisomer:*</u>

Rf(PE/EtOAc 98:2): 0.36.
^{1}H NMR (400 MHz, chloroform-*d*) δ 7.43 (dd, *J* = 8.1, 1.3 Hz, 2H, Ar*H*), 7.40 – 7.29 (m, 3H, Ar*H*), 5.24 (d, *J* = 12.2 Hz, 1H, OCH_2Ph), 5.16 (d, *J* = 12.2 Hz, 1H, OCH_2Ph), 3.92 (q, *J* = 9.0 Hz, 1H, SC*H*), 3.34 (tt, *J* = 8.5, 2.8 Hz, 1H, C*H*C(O)), 2.45 – 2.18 (m, 3H, SCHCH_2 + CH_2CHC(O)), 2.17 – 2.02 (m, 1H, CH_2CHC(O)), 1.97 (s, 3H, SCCH_2C*H*), 1.76 (q, *J* = 12.5 Hz, 6H, SCCH_2), 1.70 – 1.52 (m, 6H, SCCH_2CHCH_2).
^{13}C NMR (101 MHz, chloroform-*d*) δ 173.4, 136.2, 128.9, 128.6, 128.3, 66.4, 47.9, 45.4, 43.8, 36.5, 36.4, 30.4, 29.8, 21.4.
IR (ν_{max}, cm^{-1}) 3032 (w), 2903 (s), 2849 (m), 1729 (s), 1455 (m), 1382 (w), 1344 (m), 1278 (m), 1152 (s), 1043 (m), 910 (w), 752 (m), 734 (m).
HRMS (nanochip-ESI/LTQ-Orbitrap) m/z: $[M + H]^+$ Calcd for $C_{22}H_{29}O_2S^+$ 357.1883; Found 357.1880.

Benzyl 2-(acetylthio)cyclobutane-1-carboxylate (3x)

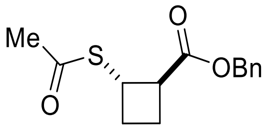

Prepared according to the general procedure C from thioacetic acid (22.8 mg, 21.1 μL, 300 μmol, 1.00 equiv.) and cyclobutene **2a** (62.1 mg, 330 μmol, 1.10 equiv.). The crude product dr (dr 67:33) was purified by flash chromatography using PE/Et_2O 95:5 to afford **3x** and **3'x** as an oil (56.3 mg, 213 μmol, 71% yield).

<u>*Data for the mixture of diastereoisomer:*</u>

Rf(PE/EtOAc 98:2): 0.21.
1H NMR (400 MHz, chloroform-*d,* 65:35 mixture of diastereoisomers (major:minor)) δ 7.34 (td, *J* = 7.9, 5.0 Hz, 5H, Ar*H* (major+minor)), 5.25 – 5.01 (m, 2H, OCH_2Ph (major+minor)), 4.49 (q, *J* = 8.4 Hz, 0.35H, SC*H* (minor)), 4.32 (q, *J* = 9.0 Hz, 0.65H, SC*H* (major)), 3.62 – 3.46 (m, 0.35H, C*H*C(O) (minor), 3.19 (q, *J* = 8.9 Hz, 0.65H, C*H*C(O) (major)), 2.44 – 2.14 (m, 6.35H, CH_2CHC(O) (major+minor) + C(O)CH_3 (major+minor) + SCHCH_2 (major+minor)), 2.12 – 1.98 (m, 0.65H, SCHCH_2 (major)).

^{13}C NMR (101 MHz, chloroform-*d*, 65:35 mixture of diastereoisomers (major:minor)) δ 195.2, 195.2, 173.0, 172.8, 136.0, 136.0, 128.7, 128.6, 128.4, 128.3, 66.7, 66.6, 45.9, 44.9, 39.1, 38.3, 30.6, 30.4, 27.7, 26.6, 22.7, 21.9.
IR (ν_{max}, cm^{-1}) 2982 (w), 2950 (w), 1736 (s), 1692 (s), 1455 (w), 1389 (w), 1353 (m), 1245 (m), 1186 (m), 1159 (s), 1132 (m), 1032 (w), 950 (m), 752 (m).
HRMS (nanochip-ESI/LTQ-Orbitrap) m/z: [M + Na]+ Calcd for C14H16NaO3S+ 287.0712; Found 287.0708.

Butyl 2-((2-bromophenyl)thio)cyclobutane-1-carboxylate (3y)

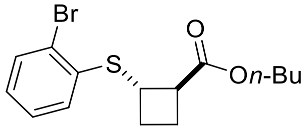

Prepared according to the general procedure C from 2-bromothiophenol (56.7 mg, 36.1 µL, 300 µmol, 1.00 equiv.) and cyclobutene **2b** (50.9 mg, 330 µmol, 1.10 equiv.). The crude product (dr 73:27) was purified by flash chromatography using PE/EtOAc 98:2 to afford **3y** and **3'y** as an oil (101 mg, 294 µmol, 98% yield).

Prepared according to the general procedure D from 2-bromothiophenol (56.7 mg, 36.1 µL, 300 µmol, 1.00 equiv.) and cyclobutene **2b** (50.9 mg, 330 µmol, 1.10 equiv.). The crude product (dr 94:6) was purified by flash chromatography using PE/EtOAc 98:2 to afford **3y** and **3'y** as an oil (98.8 mg, 288 µmol, 96% yield).

Data for the trans- diastereoisomer:

Rf(PE/EtOAc 98:2): 0.61.
^{1}H NMR (400 MHz, chloroform-*d*) δ 7.53 (d, *J* = 8.1 Hz, 1H, Ar*H*), 7.33 – 7.17 (m, 2H, Ar*H*), 7.05 – 6.96 (m, 1H, Ar*H*), 4.18 (q, *J* = 8.2 Hz, 1H, SC*H*), 4.07 (t, *J* = 6.7 Hz, 2H, OCH_2), 3.17 (q, *J* = 8.7 Hz, 1H, C*H*C(O)), 2.50 (qd, *J* = 8.6, 3.5 Hz, 1H, SCHCH_2), 2.41 – 2.28 (m, 1H, CH_2CHC(O)), 2.28 – 2.14 (m, 1H, CH_2CHC(O)), 2.14 – 2.00 (m, 1H, SCHCH_2), 1.70 – 1.49 (m, 2H, OCH_2CH_2), 1.34 (dq, *J* = 14.6, 7.4 Hz, 2H, CH_2CH_3), 0.92 (t, *J* = 7.4 Hz, 3H, CH_3).
^{13}C NMR (101 MHz, chloroform-*d*) δ 173.6, 137.4, 133.1, 129.3, 127.9, 127.1, 123.7, 64.9, 46.2, 41.6, 30.8, 27.0, 22.6, 19.2, 13.9.
IR (ν_{max}, cm^{-1}) 2960 (s), 2933 (m), 2872 (m), 1730 (s), 1447 (s), 1359 (m), 1231 (s), 1202 (s), 1163 (s), 1123 (m), 1089 (m), 1015 (m), 936 (m), 750 (s).
HRMS (nanochip-ESI/LTQ-Orbitrap) m/z: $[M + H]^+$ Calcd for $C_{15}H_{20}{}^{79}BrO_2S^+$ 343.0362; Found 343.0373.

2-((2-Bromophenyl)thio)-N-methoxy-N-methylcyclobutane-1-carboxamide (3z)

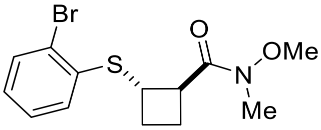

Prepared according to the general procedure C from 2-bromothiophenol (56.7 mg, 36.1 µL, 300 µmol, 1.00 equiv.) and cyclobutene **2c** (46.6 mg, 330 µmol, 1.10 equiv.). The crude product (dr 60:40) was purified by flash chromatography using PE/EtOAc 80:20 to afford **3z** and **3'z** as an oil (53.5 mg, 162 µmol, 54% yield).

Prepared according to the general procedure D from 2-bromothiophenol (56.7 mg, 36.1 µL, 300 µmol, 1.00 equiv.) and cyclobutene **2c** (46.6 mg, 330 µmol, 1.10 equiv.). The crude product

(dr >95:5) was purified by flash chromatography using PE/EtOAc 80:20 to afford **3z** and **3'z** as an oil (92.1 mg, 279 µmol, 93% yield).

The configuration of the two diastereoisomers obtained was determined by 2D-NOESY.

Data for the trans- diastereoisomer:

Rf(PE/EtOAc 8:2): 0.48.
^{1}H NMR (400 MHz, chloroform-*d*) δ 7.50 (d, *J* = 7.5 Hz, 1H, Ar*H*), 7.32 – 7.16 (m, 2H, Ar*H*), 6.98 (ddd, *J* = 8.7, 6.6, 2.4 Hz, 1H, Ar*H*), 4.33 (q, *J* = 8.3 Hz, 1H, SC*H*), 3.72 – 3.48 (m, 4H, C*H*C(O) + OC*H₃*), 3.17 (s, 3H, NC*H₃*), 2.52 (qd, *J* = 8.2, 3.1 Hz, 1H, SCHC*H₂*), 2.40 – 2.26 (m, 1H, C*H₂*CHC(O)), 2.26 – 2.16 (m, 1H, C*H₂*CHC(O)), 2.16 – 2.03 (m, 1H, SCHC*H₂*).
^{13}C NMR (101 MHz, chloroform-*d*) δ 173.8, 138.0, 133.0, 128.3, 127.9, 126.5, 122.4, 61.8, 43.5, 40.2, 32.4, 27.2, 22.8.
IR (ν_{max}, cm^{-1}) 2941 (m), 1656 (s), 1449 (s), 1426 (s), 1383 (m), 1256 (m), 1173 (m), 1109 (m), 1021 (m), 991 (m), 748 (s).
HRMS (nanochip-ESI/LTQ-Orbitrap) m/z: [M + H]$^+$ Calcd for $C_{13}H_{17}BrNO_2S^+$ 330.0158; Found 330.0167.

Data for the cis- diastereoisomer:

Rf(PE/EtOAc 8:2): 0.33.
^{1}H NMR (400 MHz, chloroform-*d*) δ 7.51 (d, *J* = 7.9 Hz, 1H, Ar*H*), 7.25 – 7.15 (m, 2H, Ar*H*), 7.03 – 6.92 (m, 1H, Ar*H*), 4.25 (q, *J* = 7.4 Hz, 1H, SC*H*), 3.95 (d, *J* = 7.4 Hz, 1H, C*H*C(O)), 3.61 (s, 3H, OC*H₃*), 3.17 (s, 3H, NC*H₃*), 2.76 – 2.59 (m, 1H, C*H₂*CH(O)), 2.60 – 2.45 (m, 1H, SCHC*H₂*), 2.22 (tt, *J* = 11.8, 6.3 Hz, 1H, SCHC*H₂*), 2.16 – 2.01 (m, 1H, C*H₂*CH(O)).
^{13}C NMR (101 MHz, chloroform-*d*) δ 172.8, 138.3, 133.0, 129.1, 127.6, 126.7, 123.9, 61.3, 43.3, 41.0, 32.4, 28.2, 21.1.
IR (ν_{max}, cm^{-1}) 2941 (m), 1660 (s), 1447 (s), 1426 (m), 1384 (m), 1246 (m), 1173 (m), 1116 (m), 1022 (m), 993 (m), 748 (s).
HRMS (nanochip-ESI/LTQ-Orbitrap) m/z: [M + H]$^+$ Calcd for $C_{13}H_{17}BrNO_2S^+$ 330.0158; Found 330.0167.

Ethyl 4-(2-((2-bromophenyl)thio)cyclobutane-1-carboxamido)benzoate (3aa)

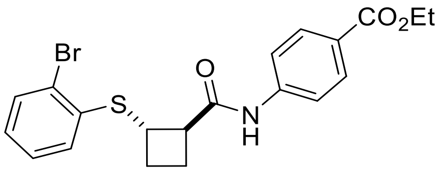

Prepared according to the general procedure D from 2-bromothiophenol (56.7 mg, 36.1 µL, 300 µmol, 1.00 equiv.) and cyclobutene **2d** (80.9 mg, 330 µmol, 1.10 equiv.). The crude product (dr >95:5) was purified by flash chromatography using PE/Et_2O 30:70 to afford **3aa** and **3'aa** as an oil (88.9 mg, 205 µmol, 68% yield).

Data for the trans- diastereoisomer:

Rf(PE/Et_2O 2:8): 0.71.
^{1}H NMR (400 MHz, chloroform-*d*) δ 8.05 – 7.89 (m, 2H, Ar*H*), 7.60 – 7.51 (m, 3H, Ar*H*), 7.47 (s, 1H, N*H*), 7.33 (dd, *J* = 7.9, 1.6 Hz, 1H, Ar*H*), 7.26 – 7.18 (m, 1H, Ar*H*), 7.05 (td, *J* = 7.7, 1.6 Hz, 1H, Ar*H*), 4.36 (q, *J* = 7.1 Hz, 2H, OC*H₂*), 4.14 (q, *J* = 8.4 Hz, 1H, SC*H*), 3.13 (q, *J* = 8.6 Hz, 1H, C*H*C(O)), 2.53 – 2.32 (m, 2H, SCHC*H₂* + C*H₂*CHC(O)), 2.30 – 2.19 (m, 1H, C*H₂*CHC(O)), 2.18 – 2.05 (m, 1H, SCHC*H₂*), 1.38 (t, *J* = 7.1 Hz, 3H, OCH₂C*H₃*).

^{13}C NMR (101 MHz, chloroform-*d*) δ 171.0, 166.2, 141.8, 136.1, 133.4, 131.0, 130.9, 128.2, 128.1, 126.2, 124.9, 118.9, 61.0, 48.7, 43.2, 27.1, 21.4, 14.5.
IR (ν_{max}, cm^{-1}) 3332 (w), 3060 (w), 2989 (w), 2939 (m), 2857 (w), 1712 (s), 1689 (s), 1597 (s), 1531 (s), 1447 (m), 1408 (m), 1278 (s), 1253 (s), 1173 (s), 1108 (s), 1020 (m), 860 (m), 767 (m), 745 (m).
HRMS (nanochip-ESI/LTQ-Orbitrap) m/z: $[M + Na]^+$ Calcd for $C_{20}H_{20}BrNNaO_3S^+$ 456.0239; Found 456.0252.

1.4. Crossover experiment

In a sealed vial under nitrogen, bromothiophenol **1a** (21.4 mg, 13.6 µL, 110 µmol, 1.10 equiv.) and cyclobutene **2a** (18.8 mg, 100 µmol, 1.00 equiv.) were diluted in 3 mL of MeCN. DBU (137 mg, 134 µL, 900 µmol, 3.00 equiv.) was then added. The reaction was stirred at rt for 1 h, at which time full conversion of **2a** was observed. Methyl thiosalicylate (18.5 mg, 15.2 µL, 110 µmol, 1.10 equiv) was then added and the reaction was stirred at rt for 18 h. The reaction mixture concentrated under reduced pressure and analyzed by ^1H NMR using dibromomethane as internal standard; **3a** was observed in 71% NMR yield, **3f** in 19% NMR yield.

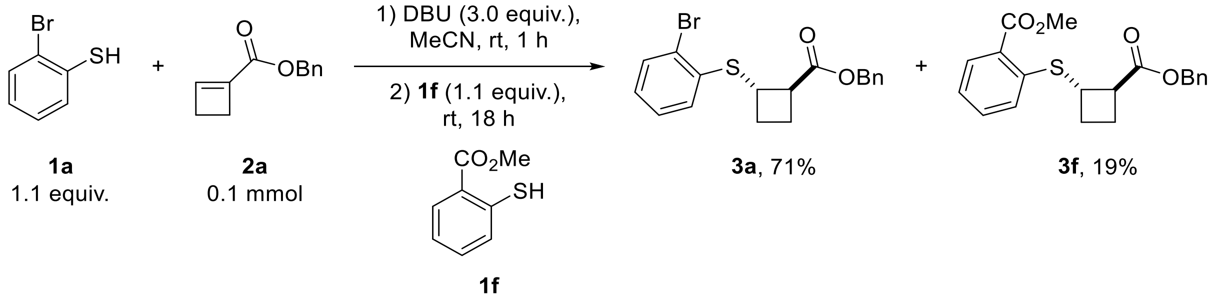

1.5. Optimization of the enantioselective sulfa-Michael addition

All catalysts were commercially available, purchased from Fluorochem or Combi-Blocks, and used as such.

General procedure for the optimization of the reaction

In a sealed vial under nitrogen, the chiral catalyst and cyclobutene **2e** were diluted in 1 mL of solvent. Thiol **1a** was then added. The reaction was stirred at the indicated temperature for the indicated time. The reaction mixture was concentrated under reduced pressure. The reaction mixture was analyzed by ^{1}H NMR (400 MHz, chloroform-*d*) using dibromomethane as an internal standard and by integration of the SC*H* proton at 4.28 (q, *J* = 8.5 Hz, 1H) ppm (yield and dr were measured). The crude was then purified by prep-TLC using PE/Et_2O 30:70 and submitted to SFC analysis (IC column, 9% MeCN in supercritical CO_2, flow rate 0.75 mL/min, λ = 250.4 nm. tR(major) = 23.2 min, tR(minor) = 20.4 min).

Screen of catalysts

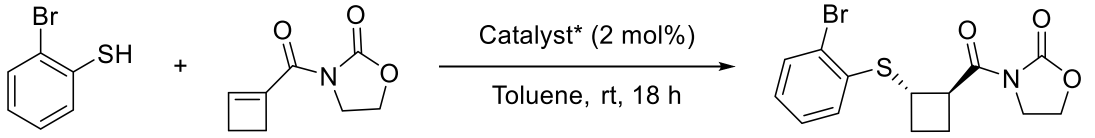

1a 0.1 mmol

2e 1.2 equiv.

4a

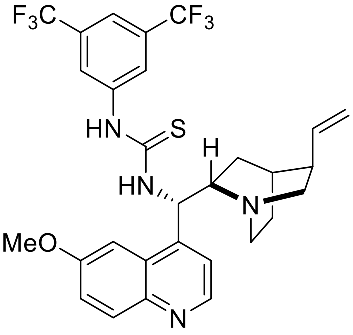

Cat*1
92% yield
dr 79:21
er 96:4

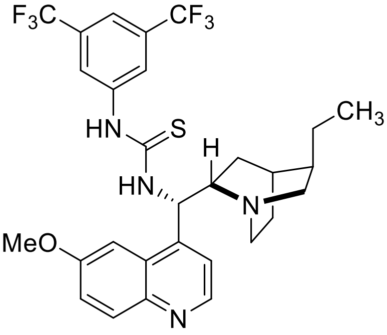

Cat*2
quant. yield
dr 85:15
er 96:4

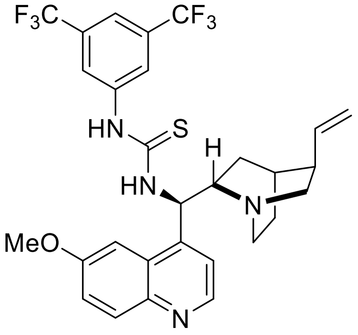

Cat*3
quant. yield
dr 71:29
er 6:94

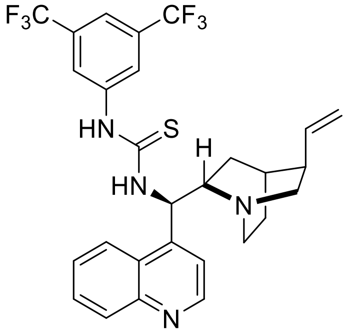

Cat*4
86% yield
dr 48:52
er 5:95

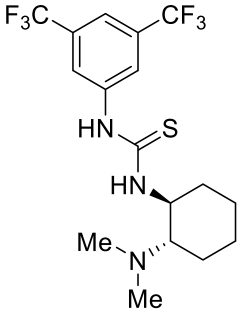

Cat*5
98% yield
dr 82:18
er 92:8

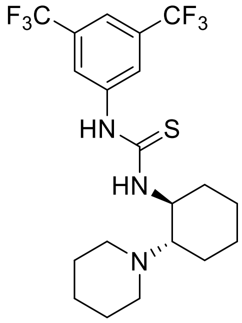

Cat*6
quant. yield
dr 55:45
er 38:62

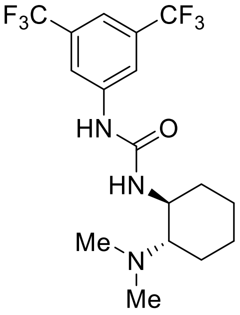

Cat*7
84% yield
dr 66:34
er 86:13

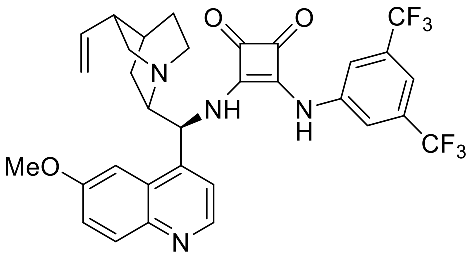

Cat*8
99% yield
dr 89:11
er 98:2

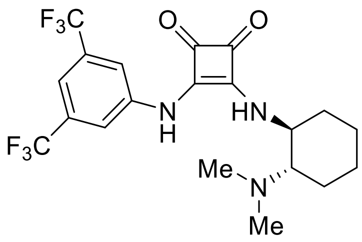

Cat*9
98% yield
dr 91:9
er 90:10

Optimization table

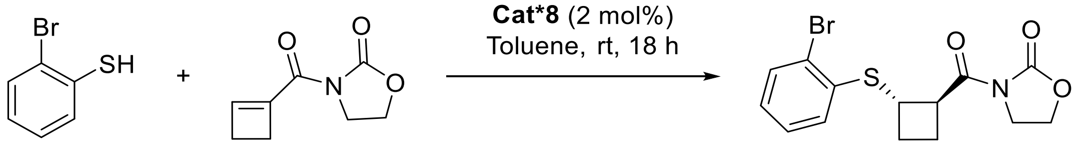

1a 0.1 mmol **2e** 1.2 equiv. **4a**

Entry	Deviation from standard conditions	NMR Yield (%)[b]	dr [c]	er [d]
1	None	99%	89:11	98:2
2	5 mol% instead of 2 mol% of **Cat*8**	quant.	90:10	98:2
3	$CHCl_3$ instead of toluene	quant.	90:10	97:3
4	DCM instead of toluene	97%	78:22	96:4
5	0 °C instead of rt	quant.	86:14	99:1
6	1.2 equiv. **1a** and 1.0 equiv. **2e**	91%	89:11	98:2
7	2 h instead of 18 h	69%	90:10	98:2

[a]Reaction conditions: 1.0 equiv. thiol **1a** (0.1 mmol), 1.2 equiv. cyclobutene **2e**, 2 mol% **Cat*8**. [b]1H NMR of the crude mixture with dibromomethane as an internal standard. [c]Measured on the crude ^{1}H NMR. [d]Measured on the SFC chromatogram.

1.6. Enantioselective sulfa-Michael addition onto cyclobutenes

General procedure (E) for the enantioselective sulfa-Michael addition

In a sealed vial under nitrogen, **Cat*8** (1.26 mg, 2.00 µmol, 0.0200 equiv.) and cyclobutene **2e** (18.4 mg, 110 µmol, 1.10 equiv.) were diluted in 1 mL of dry toluene. Thiol **1a-u** was then added. The reaction was stirred at room temperature for 18 h. The reaction mixture was concentrated under reduced pressure and purified by prep-TLC. The mixture of diastereoisomers was isolated and the yield measured. For characterization purposes, a prep-TLC was then performed and only the major diastereoisomer was isolated and characterized.

The dr was measured from the crude ^{1}H NMR spectra by integration of the SC*H* proton. The er was measured from the SFC analysis of the purified major diastereoisomer.

3-(2-((2-Bromophenyl)thio)cyclobutane-1-carbonyl)oxazolidin-2-one (4a)

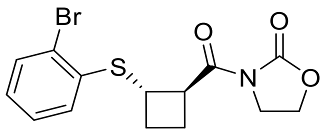

Racemic: Prepared according to the general procedure C from 2-bromothiophenol (18.9 mg, 12.0 µL, 100 µmol, 1.00 equiv.) and cyclobutene **2e** (55.2 mg, 330 µmol, 1.10 equiv.). The crude product (dr >95:5) was purified by flash chromatography using PE/Et_2O 30:70 to afford **4a** and **4'a** as an oil (88.4 mg, 248 µmol, 83% yield).

Enantioenriched: Prepared according to the general procedure E from 2-bromothiophenol (18.9 mg, 12.0 µL, 100 µmol, 1.00 equiv.). The crude product (dr 89:11) was purified by prep-TLC using PE/Et_2O 30:70 to afford **4a** and **4'a** as an oil (35.2 mg, 98.8 µmol, 99% yield).

A scale up experiment with 2-bromothiophenol (151 mg, 96.2 µL, 800 µmol, 1.00 equiv.), cyclobutene **2e** (160 mg, 960 µmol, 1.20 equiv.) and **Cat*8** (10.1 mg, 16.0 µmol, 0.0200 equiv.) was also accomplished using the same procedure and led to **4a** (271 mg, 761 µmol, 95% yield, 91:9 dr, 98:2 er).

Rf(PE/EtOAc 8:2): 0.39.
$[\alpha]_D^{23}$ = 100.3 (c = 0.0467, $CHCl_3$).
^{1}H NMR (400 MHz, chloroform-*d*) δ 7.53 (dd, *J* = 8.0, 1.3 Hz, 1H, Ar*H*), 7.33 (dd, *J* = 7.9, 1.5 Hz, 1H, Ar*H*), 7.29 – 7.20 (m, 1H, Ar*H*), 7.02 (td, *J* = 7.7, 1.6 Hz, 1H, Ar*H*), 4.49 – 4.33 (m, 3H, OCH_2 + SC*H*), 4.28 (q, *J* = 8.5 Hz, 1H, C*H*C(O)), 4.06 – 3.91 (m, 2H, NCH_2), 2.63 – 2.43 (m, 2H, SCHCH_2 + CH_2CH(O)), 2.22 – 1.98 (m, 2H, SCHCH_2 + CH_2CH(O)).
^{13}C NMR (101 MHz, chloroform-*d*) δ 172.9, 153.1, 137.4, 133.1, 129.5, 127.9, 127.1, 123.8, 62.3, 45.5, 42.6, 39.4, 27.0, 23.9.
IR (ν_{max}, cm^{-1}) 2921 (w), 1782 (s), 1692 (m), 1479 (w), 1386 (s), 1278 (w), 1227 (m), 1118 (w), 1042 (w), 911 (m), 740 (m).
HRMS (APCI/QTOF) m/z: $[M + Na]^+$ Calcd for $C_{14}H_{14}BrNNaO_3S^+$ 377.9770; Found 377.9758.

The enantiomeric ratio was determined to be 98:2 by SFC analysis: IC column, 9% MeCN in supercritical CO_2, flow rate 0.75 mL/min, λ = 250.4 nm. tR(major) = 23.2 min, tR(minor) = 20.4 min.).

Methyl 2-((2-(2-oxooxazolidine-3-carbonyl)cyclobutyl)thio)benzoate (4b)

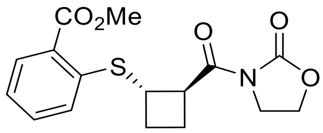

Racemic: Prepared according to the general procedure C from methyl 2-mercaptobenzoate (16.8 mg, 13.8 µL, 100 µmol, 1.00 equiv.) and cyclobutene **2e** (18.4 mg, 110 µmol, 1.10 equiv.). The crude product (dr >95:5) was purified by flash chromatography using PE/Et_2O 30:70 to afford **4b** and **4'b** as a white solid (31.4 mg, 93.6 µmol, 94% yield).

Enantioenriched: Prepared according to the general procedure E from methyl 2-mercaptobenzoate (16.8 mg, 13.8 µL, 100 µmol, 1.00 equiv.). The crude product (dr >95:5) was purified by prep-TLC using PE/Et_2O 25:75 to afford **4b** and **4'b** as a white solid (31.2 mg, 93.0 µmol, 93% yield).

m.p.:102-104 °C.
Rf(PE/Et_2O 3:7): 0.31.
$[\alpha]_D^{23}$ = 80.4 (c = 0.0367, $CHCl_3$).
^{1}H NMR (400 MHz, chloroform-*d*) δ 7.94 (dd, *J* = 7.8, 1.4 Hz, 1H, Ar*H*), 7.44 – 7.37 (m, 1H, Ar*H*), 7.33 (d, *J* = 7.9 Hz, 1H, Ar*H*), 7.15 (t, *J* = 7.5 Hz, 1H, Ar*H*), 4.49 (q, *J* = 7.9 Hz, 1H, SC*H*), 4.40 (td, *J* = 8.3, 3.3 Hz, 2H, OC*H*$_2$), 4.25 (q, *J* = 8.2 Hz, 1H, C*H*C(O)), 4.05 – 3.96 (m, 2H, NC*H*$_2$), 3.90 (s, 3H, OC*H*$_3$), 2.70 – 2.49 (m, 2H, SCHC*H*$_2$ + C*H*$_2$CHC(O)), 2.24 – 2.00 (m, 2H, SCHC*H*$_2$ + C*H*$_2$CHC(O)).
^{13}C NMR (101 MHz, chloroform-*d*) δ 173.1, 167.0, 153.0, 141.3, 132.5, 131.4, 127.6, 126.9, 124.3, 62.3, 52.3, 45.1, 42.6, 38.1, 27.2, 24.2.
IR (v_{max}, cm^{-1}) 3011 (w), 2957 (w), 1782 (m), 1714 (s), 1465 (m), 1437 (m), 1390 (m), 1280 (s), 1252 (s), 1145 (w), 1116 (w), 1062 (m), 746 (s).
HRMS (nanochip-ESI/LTQ-Orbitrap) m/z: $[M + Na]^+$ Calcd for $C_{16}H_{17}NNaO_5S^+$ 358.0720; Found 358.0722.

The enantiomeric ratio was determined to be 97:3 by SFC analysis: IA column, 9% MeCN in supercritical CO_2, flow rate 2.0 mL/min, λ = 260.4 nm. tR(major) = 5.8 min, tR(minor) = 12.2 min.

3-(2-((4-(tert-Butyl)phenyl)thio)cyclobutane-1-carbonyl)oxazolidin-2-one (4c)

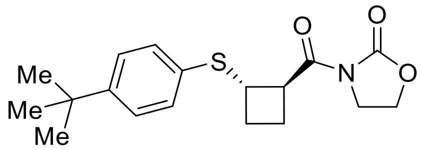

Racemic: Prepared according to the general procedure C from 4-tert-butylbenzenethiol (16.6 mg, 17.3 µL, 100 µmol, 1.00 equiv.) and cyclobutene **2e** (18.4 mg, 110 µmol, 1.10 equiv.). The crude product (dr 93:7) was purified by flash chromatography using PE/Et_2O 20:80 to afford **4c** and **4'c** as an oil (30.9 mg, 92.7 µmol, 93% yield).

Enantioenriched: Prepared according to the general procedure E from 4-tert-butylbenzenethiol (16.6 mg, 17.3 µL, 100 µmol, 1.00 equiv.). The crude product (dr 91:9) was purified by prep-TLC using PE/Et_2O 25:75 to afford **4c** and **4'c** as an oil (27.4 mg, 82.2 µmol, 82% yield).

Rf(PE/Et_2O 2:8): 0.56.
$[\alpha]_D^{23}$ = 182.4 (c = 0.0167, $CHCl_3$).

^{1}H NMR (400 MHz, chloroform-*d*) δ 7.33 (s, 4H, Ar*H*), 4.51 – 4.33 (m, 2H, OC*H₂*), 4.32 – 4.19 (m, 2H, SC*H* + C*H*C(O)), 3.97 (qdd, *J* = 11.0, 9.1, 7.1 Hz, 2H, NC*H₂*), 2.51 – 2.28 (m, 2H, SCHC*H₂* + C*H₂*CHC(O)), 2.18 – 1.90 (m, 2H, SCHC*H₂* + C*H₂*CHC(O)), 1.32 (s, 9H, C(C*H₃*)₃).
^{13}C NMR (101 MHz, chloroform-*d*) δ 173.1, 153.2, 150.1, 131.3, 131.3, 126.0, 62.2, 45.5, 42.6, 41.5, 34.7, 31.4, 27.0, 23.3.
IR (ν_{max}, cm^{-1}) 2960 (m), 2909 (w), 2871 (w), 1779 (s), 1692 (s), 1491 (w), 1385 (s), 1270 (m), 1222 (m), 1119 (m), 1083 (w), 1044 (m), 824 (m), 759 (m).
HRMS (APCI/QTOF) m/z: [M + Na]$^+$ Calcd for $C_{18}H_{23}NNaO_3S^+$ 356.1291; Found 356.1281.

The enantiomeric ratio was determined to be 99.7:0.3 by SFC analysis: IA column, 9% MeCN in supercritical CO_2, flow rate 2.0 mL/min, λ = 214.4 nm. tR(major) = 3.3 min, tR(minor) = 2.2 min.

3-(2-((3-Methoxyphenyl)thio)cyclobutane-1-carbonyl)oxazolidin-2-one (4d)

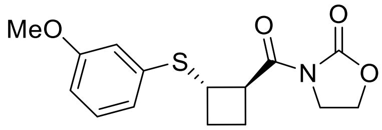

Racemic: Prepared according to the general procedure C from 3-methoxybenzenethiol (14.0 mg, 12.4 µL, 100 µmol, 1.00 equiv.) and cyclobutene **2e** (18.4 mg, 110 µmol, 1.10 equiv.). The crude product (dr 93:7) was purified by flash chromatography using PE/Et_2O 20:80 to afford **4d** and **4'd** as an oil (27.3 mg, 88.8 µmol, 89% yield).

Enantioenriched: Prepared according to the general procedure E from 3-methoxybenzenethiol (14.0 mg, 12.4 µL, 100 µmol, 1.00 equiv.). The crude product (dr 88:12) was purified by prep-TLC using PE/Et_2O 25:75 to afford **4d** and **4'd** as an oil (30.6 mg, 99.6 µmol, quant.).

Rf(PE/Et_2O 2:8): 0.45.
$[\alpha]_D^{23}$ = 88.3 (c = 0.0433, $CHCl_3$).
^{1}H NMR (400 MHz, chloroform-*d*) δ 7.18 (t, *J* = 7.9 Hz, 1H, Ar*H*), 6.98 – 6.87 (m, 2H, Ar*H*), 6.74 (ddd, *J* = 8.3, 2.5, 0.9 Hz, 1H, Ar*H*), 4.45 – 4.16 (m, 4H, SC*H* + C*H*C(O) + OC*H₂*), 4.07 – 3.83 (m, 2H, NC*H₂*), 3.80 (s, 3H, OC*H₃*), 2.50 – 2.28 (m, 2H, SCHC*H₂* + C*H₂*CHC(O)), 2.16 – 1.93 (m, 2H, SCHC*H₂* + C*H₂*CHC(O)).
^{13}C NMR (101 MHz, chloroform-*d*) δ 173.1, 159.9, 153.1, 136.5, 129.8, 122.9, 115.6, 112.8, 62.2, 55.5, 45.7, 42.6, 41.2, 27.0, 23.3.
IR (ν_{max}, cm^{-1}) 2991 (w), 2946 (w), 1778 (s), 1692 (s), 1588 (m), 1478 (m), 1385 (s), 1281 (m), 1228 (s), 1117 (m), 1079 (m), 1037 (s), 860 (m), 760 (m).
HRMS (APCI/QTOF) m/z: [M + Na]$^+$ Calcd for $C_{15}H_{17}NNaO_4S^+$ 330.0770; Found 330.0763.

The enantiomeric ratio was determined to be 99:1 by SFC analysis: IA column, 18% MeOH in supercritical CO_2, flow rate 2.0 mL/min, λ = 214.4 nm. tR(major) = 1.2 min, tR(minor) = 2.4 min.

3-(2-(Pyridin-2-ylthio)cyclobutane-1-carbonyl)oxazolidin-2-one (4e)

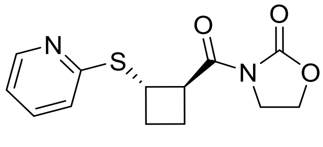

Racemic: Prepared according to the general procedure C from 2-pyridinethiol (11.1 mg, 100 µmol, 1.00 equiv.) and cyclobutene **2e** (18.4 mg, 110 µmol, 1.10 equiv.). The crude product (dr >95:5) was purified by flash chromatography using PE/Et_2O 20:80 to afford **4e** and **4'e** as an oil (20.3 mg, 72.9 µmol, 73% yield).

Enantioenriched: Prepared according to the general procedure E from pyridine-2-thiol (11.1 mg, 100 μmol, 1.00 equiv.). The crude product (dr 61:39) was purified by prep-TLC using PE/Et_2O 25:75 to afford **4e** and **4'e** as an oil (26.4 mg, 94.9 μmol, 95% yield).

Rf(PE/Et_2O 2:8): 0.34.
$[\alpha]_D^{23}$ = 92.6 (c = 0.0300, $CHCl_3$).
^{1}H NMR (400 MHz, chloroform-*d*) δ 8.39 – 8.29 (m, 1H, Ar*H*), 7.46 (td, *J* = 7.7, 1.9 Hz, 1H, Ar*H*), 7.16 (d, *J* = 8.1 Hz, 1H, Ar*H*), 6.95 (ddd, *J* = 7.3, 4.9, 0.9 Hz, 1H, Ar*H*), 4.71 (q, *J* = 8.3 Hz, 1H, SC*H*), 4.48 (q, *J* = 8.7 Hz, 1H, C*H*C(O)), 4.42 – 4.26 (m, 2H, OC*H*$_2$), 4.07 – 3.95 (m, 2H, NC*H*$_2$), 2.47 (ddt, *J* = 14.8, 12.7, 4.4 Hz, 2H, SCHC*H*$_2$ + C*H*$_2$CHC(O)), 2.30 – 2.06 (m, 2H, SCHC*H*$_2$ + C*H*$_2$CHC(O)).
^{13}C NMR (101 MHz, chloroform-*d*) δ 173.2, 159.5, 153.2, 149.2, 136.1, 122.3, 119.6, 62.1, 45.3, 42.8, 39.2, 26.7, 23.4.
IR (ν_{max}, cm^{-1}) 2993 (w), 2953 (w), 1779 (s), 1695 (s), 1578 (m), 1455 (m), 1415 (m), 1386 (s), 1275 (m), 1227 (m), 1127 (m), 1044 (m), 986 (w), 917 (w), 759 (m).
HRMS (APCI/QTOF) m/z: $[M + Na]^+$ Calcd for $C_{13}H_{14}N_2NaO_3S^+$ 301.0617; Found 301.0604.

The enantiomeric ratio was determined to be 96:4 by SFC analysis: IA column, 9% MeCN in supercritical CO_2, flow rate 2.0 mL/min, λ = 250.4 nm. tR(major) = 3.7 min, tR(minor) = 7.0 min.

3-(2-(Benzylthio)cyclobutane-1-carbonyl)oxazolidin-2-one (4f)

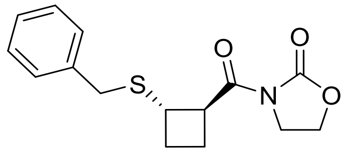

Racemic: Prepared according to the general procedure C from phenylmethanethiol (12.4 mg, 11.7 μL, 100 μmol, 1.00 equiv.) and cyclobutene **2e** (18.4 mg, 110 μmol, 1.10 equiv.). The crude product (dr 90:10) was purified by flash chromatography using PE/EtOAc 80:20 to afford **4f** and **4'f** as an oil (29.0 mg, 99.5 μmol, quant.).

Enantioenriched: Prepared according to the general procedure E from phenylmethanethiol (12.4 mg, 11.7 μL, 100 μmol, 1.00 equiv.). The crude product (dr 68:32) was purified by prep-TLC using PE/Et_2O 30:70 to afford **4f** and **4'f** as an oil (29.0 mg, 99.5 μmol, quant. yield).

Rf(PE/EtOAc 8:2): 0.41.
$[\alpha]_D^{23}$ = 145.4 (c = 0.0200, $CHCl_3$).
^{1}H NMR (400 MHz, chloroform-*d*) δ 7.35 (d, *J* = 7.2 Hz, 2H, Ar*H*), 7.29 (dd, *J* = 8.2, 6.7 Hz, 2H, Ar*H*), 7.24 – 7.18 (m, 1H, Ar*H*), 4.47 – 4.27 (m, 2H, OC*H*$_2$), 4.12 (q, *J* = 9.1 Hz, 1H, SC*H*), 4.01 – 3.82 (m, 3H, CHC(O) + NC*H*$_2$), 3.79 – 3.61 (m, 2H, SC*H*$_2$Ph), 2.47 – 2.32 (m, 1H, SCHC*H*$_2$), 2.21 – 2.10 (m, 1H, C*H*$_2$CHC(O)), 2.03 – 1.79 (m, 2H, SCHC*H*$_2$ + C*H*$_2$CHC(O)).
^{13}C NMR (101 MHz, chloroform-*d*) δ 173.2, 153.1, 138.9, 129.0, 128.5, 126.9, 62.2, 46.7, 42.5, 39.4, 36.3, 26.5, 23.8.
IR (ν_{max}, cm^{-1}) 2987 (w), 2943 (w), 1779 (s), 1692 (s), 1483 (w), 1453 (w), 1386 (s), 1267 (m), 1224 (s), 1116 (m), 1077 (m), 1044 (m), 759 (m).
HRMS (ESI/QTOF) m/z: $[M + Na]^+$ Calcd for $C_{15}H_{17}NNaO_3S^+$ 314.0821; Found 314.0824.

The enantiomeric ratio was determined to be 98:2 by SFC analysis: IA column, 9% MeCN in supercritical CO_2, flow rate 2.0 mL/min, λ = 220.4 nm. tR(major) = 2.9 min, tR(minor) = 8.7 min.

3-(2-((4-Methoxybenzyl)thio)cyclobutane-1-carbonyl)oxazolidin-2-one (4g)

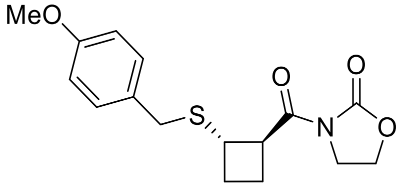

Racemic: Prepared according to the general procedure C from (4-methoxyphenyl)methanethiol (15.4 mg, 13.8 µL, 100 µmol, 1.00 equiv.) and cyclobutene **2e** (18.4 mg, 110 µmol, 1.10 equiv.). The crude product (dr 91:9) was purified by flash chromatography using PE/Et_2O 20:80 to afford **4g** and **4'g** as an oil (26.7 mg, 83.1 µmol, 83% yield).

Enantioenriched: Prepared according to the general procedure E from (4-methoxyphenyl)methanethiol (15.4 mg, 13.8 µL, 100 µmol, 1.00 equiv.). The crude product (dr 91:9) was purified by prep-TLC using PE/Et_2O 30:70 to afford **4g** and **4'g** as an oil (19.9 mg, 61.9 µmol, 62% yield).

Rf(PE/Et_2O 2:8): 0.44.
$[\alpha]_D^{23}$ = 119.6 (c = 0.0167, $CHCl_3$).
^{1}H NMR (400 MHz, chloroform-*d*) δ 7.30 – 7.22 (m, 2H, Ar*H*), 6.86 – 6.78 (m, 2H, Ar*H*), 4.36 (t, *J* = 8.1 Hz, 2H, OC*H₂*), 4.11 (q, *J* = 9.1 Hz, 1H, SC*H*), 3.97 – 3.83 (m, 3H, C*H*C(O) + NC*H₂*), 3.79 (s, 3H, OC*H₃*), 3.75 – 3.58 (m, 2H, SC*H₂*), 2.47 – 2.31 (m, 1H, SCHC*H₂*), 2.18 (dt, *J* = 9.9, 8.6 Hz, 1H, C*H₂*CHC(O)), 2.01 – 1.82 (m, 2H, SCHC*H₂* + C*H₂*CHC(O)).
^{13}C NMR (101 MHz, chloroform-*d*) δ 173.2, 158.6, 153.1, 130.8, 130.1, 113.9, 62.2, 55.4, 46.7, 42.6, 39.4, 35.7, 26.4, 23.9.
IR (v_{max}, cm^{-1}) 2992 (w), 2927 (w), 2842 (w), 1779 (s), 1689 (s), 1613 (w), 1512 (s), 1386 (s), 1246 (s), 1181 (m), 1119 (w), 1076 (w), 1044 (m), 837 (w), 759 (m).
HRMS (ESI/QTOF) m/z: [M + Na]$^+$ Calcd for $C_{16}H_{19}NNaO_4S^+$ 344.0927; Found 344.0931.

The enantiomeric ratio was determined to be 97:3 by SFC analysis: IA column, 16% MeCN in supercritical CO_2, flow rate 2.0 mL/min, λ = 214.4 nm. tR(major) = 1.8 min, tR(minor) = 5.5 min.

3-(2-((4-Chlorobenzyl)thio)cyclobutane-1-carbonyl)oxazolidin-2-one (4h)

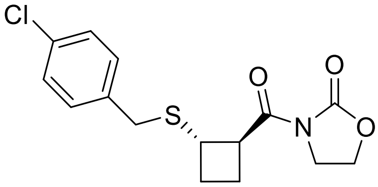

Racemic: Prepared according to the general procedure C from (4-chlorophenyl)methanethiol (15.9 mg, 13.2 µL, 100 µmol, 1.00 equiv.) and cyclobutene **2e** (18.4 mg, 110 µmol, 1.10 equiv.). The crude product (dr 93:7) was purified by flash chromatography using PE/Et_2O 20:80 to afford **4h** and **4'h** as an oil (30.2 mg, 92.7 µmol, 93% yield).

Enantioenriched: Prepared according to the general procedure E from (4-chlorophenyl)methanethiol (15.9 mg, 13.2 µL, 100 µmol, 1.00 equiv.). The crude product (dr >95:5) was purified by prep-TLC using PE/Et_2O 30:70 to afford **4h** and **4'h** as an oil (32.5 mg, 99.8 µmol, quant.).

Rf(PE/Et_2O 2:8): 0.67.
$[\alpha]_D^{23}$ = 76.7 (c = 0.0867, $CHCl_3$).
^{1}H NMR (400 MHz, chloroform-*d*) δ 7.34 – 7.20 (m, 4H, Ar*H*), 4.46 – 4.28 (m, 2H, OC*H₂*), 4.19 – 4.04 (m, 1H, SC*H*), 4.00 – 3.77 (m, 3H, NC*H₂* + C*H*C(O)), 3.74 – 3.60 (m, 2H, SC*H₂*), 2.48

– 2.31 (m, 1H, SCHC*H₂*), 2.25 – 2.08 (m, 1H, C*H₂*CHC(O)), 1.99 – 1.77 (m, 2H, SCHC*H₂* + C*H₂*CHC(O)).
^{13}C NMR (101 MHz, chloroform-*d*) δ 173.1, 153.1, 137.5, 132.6, 130.4, 128.6, 62.2, 46.9, 42.5, 39.3, 35.7, 26.4, 23.9.
IR (ν_{max}, cm^{-1}) 2986 (w), 2948 (w), 2923 (w), 2866 (w), 1777 (s), 1689 (s), 1490 (m), 1384 (s), 1274 (m), 1220 (s), 1116 (m), 1091 (m), 1045 (m), 835 (w), 755 (m).
HRMS (ESI/QTOF) m/z: [M + Na]$^+$ Calcd for $C_{15}H_{16}ClNNaO_3S^+$ 348.0432; Found 348.0439.

The enantiomeric ratio was determined to be 98:2 by SFC analysis: IA column, 16% MeCN in supercritical CO_2, flow rate 2.0 mL/min, λ = 214.4 nm. tR(major) = 2.0 min, tR(minor) = 5.4 min.

3-(2-((Furan-2-ylmethyl)thio)cyclobutane-1-carbonyl)oxazolidin-2-one (4i)

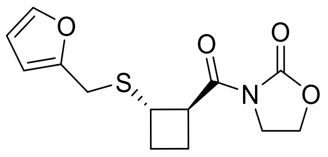

Racemic: Prepared according to the general procedure C from 2-furylmethanethiol (11.4 mg, 10.1 µL, 100 µmol, 1.00 equiv.) and cyclobutene **2e** (18.4 mg, 110 µmol, 1.10 equiv.). The crude product (dr >95:5) was purified by flash chromatography using PE/Et_2O 30:70 to afford **4i** and **4'i** as an oil (28.0 mg, 99.5 µmol, quant.).

Enantioenriched: Prepared according to the general procedure E from 2-furylmethanethiol (11.4 mg, 10.1 µL, 100 µmol, 1.00 equiv.). The crude product (dr 90:10) was purified by prep-TLC using PE/Et_2O 25:75 to afford **4i** and **4'i** as an oil (22.2 mg, 78.9 µmol, 79% yield).

Rf(PE/Et_2O 3:7): 0.39.
$[\alpha]_D^{23}$ = 94.3 (c = 0.0267, $CHCl_3$).
^{1}H NMR (400 MHz, chloroform-*d*) δ 7.34 (dd, *J* = 1.8, 0.8 Hz, 1H, Ar*H*), 6.29 (dd, *J* = 3.2, 1.9 Hz, 1H, Ar*H*), 6.23 – 6.16 (m, 1H, Ar*H*), 4.40 (t, *J* = 8.1 Hz, 2H, OC*H₂*), 4.19 – 4.06 (m, 1H, SC*H*), 4.03 – 3.90 (m, 3H, NC*H₂* + C*H*C(O)), 3.81 – 3.67 (m, 2H, SC*H₂*), 2.53 – 2.38 (m, 1H, SCHC*H₂*), 2.28 – 2.12 (m, 1H, C*H₂*CHC(O)), 2.04 – 1.86 (m, 2H, SCHC*H₂* + C*H₂*CHC(O)).
^{13}C NMR (101 MHz, chloroform-*d*) δ 173.2, 153.1, 152.1, 142.1, 110.5, 107.4, 62.3, 46.8, 42.6, 39.4, 28.3, 26.5, 23.7.
IR (ν_{max}, cm^{-1}) 2990 (w), 2946 (w), 1780 (s), 1689 (s), 1472 (w), 1386 (s), 1270 (m), 1220 (m), 1123 (w), 1077 (w), 1045 (m), 743 (m).
HRMS (nanochip-ESI/LTQ-Orbitrap) m/z: [M + Na]$^+$ Calcd for $C_{13}H_{15}NNaO_4S^+$ 304.0614; Found 304.0625.

The enantiomeric ratio was determined to be 99:1 by SFC analysis: IA column, 18% MeOH in supercritical CO_2, flow rate 2.0 mL/min, λ = 214.4 nm. tR(major) = 1.1 min, tR(minor) = 2.8 min.

3-(2-(Phenethylthio)cyclobutane-1-carbonyl)oxazolidin-2-one (4j)

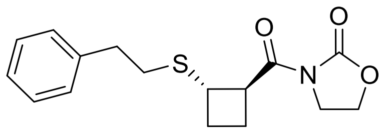

Racemic: Prepared according to the general procedure C from 2-phenylethanethiol (13.8 mg, 13.6 µL, 100 µmol, 1.00 equiv.) and cyclobutene **2e** (18.4 mg, 110 µmol, 1.10 equiv.). The crude product (dr 95:5) was purified by flash chromatography using PE/Et_2O 20:80 to afford **4j** and **4'j** as an oil (23.8 mg, 77.9 µmol, 78% yield).

Enantioenriched: Prepared according to the general procedure E from 2-phenylethanethiol (13.8 mg, 13.6 µL, 100 µmol, 1.00 equiv.). The crude product (dr 85:15) was purified by prep-TLC using PE/Et_2O 25:75 to afford **4j** and **4'j** as an oil (28.0 mg, 91.7 µmol, 92% yield).

Rf(PE/Et_2O 2:8): 0.51.
$[\alpha]_D^{23}$ = 163.4 (c = 0.0133, $CHCl_3$).
^{1}H NMR (400 MHz, chloroform-*d*) δ 7.34 – 7.23 (m, 2H, Ar*H*), 7.21 (dd, *J* = 7.1, 3.6 Hz, 3H, Ar*H*), 4.48 – 4.29 (m, 2H, OCH_2), 4.19 – 4.05 (m, 1H, SC*H*), 4.04 – 3.83 (m, 3H, NCH_2 + C*H*C(O)), 2.98 – 2.67 (m, 4H, SCH_2CH_2Ph), 2.54 – 2.40 (m, 1H, SCHCH_2), 2.32 – 2.16 (m, 1H, CH_2CHC(O)), 2.09 – 1.85 (m, 2H, SCHCH_2 + CH_2CHC(O)).
^{13}C NMR (101 MHz, chloroform-*d*) δ 173.3, 153.2, 140.8, 128.7, 128.6, 126.4, 62.3, 46.9, 42.6, 39.4, 36.7, 33.0, 26.7, 23.6.
IR (v_{max}, cm^{-1}) 3027 (w), 2947 (w), 2925 (w), 2859 (w), 1782 (s), 1691 (s), 1496 (w), 1476 (w), 1389 (s), 1265 (m), 1225 (m), 1119 (m), 1083 (w), 1051 (w).
HRMS (APCI/QTOF) m/z: $[M + Na]^+$ Calcd for $C_{16}H_{19}NNaO_3S^+$ 328.0978; Found 328.0975.

The enantiomeric ratio was determined to be 95:5 by SFC analysis: IA column, 9% MeCN in supercritical CO_2, flow rate 2.0 mL/min, λ = 214.4 nm. tR(major) = 2.7 min, tR(minor) = 6.3 min.

1.7. Product modifications

Benzyl 2-((2-bromophenyl)thio)cyclobutane-1-carboxylate (3a)

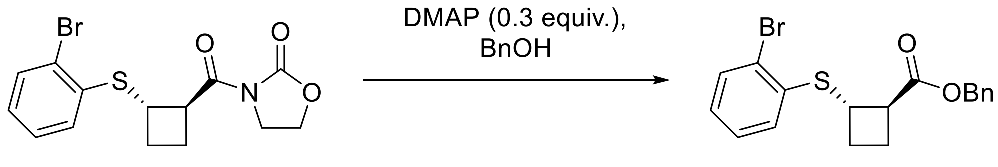

4a 97:3 er

3a, 46% 97:3 er

Racemic: The racemic compound was described above as **3a** (page 11).

Enantioenriched: Cyclobutane **4a** (28.0 mg, 78.6 μmol, 1.00 equiv.) was diluted in BnOH (786 μL) and DMAP (2.88 mg, 23.6 μmol, 0.300 equiv.) was added. The reaction mixture was stirred at rt for 18 h. The mixture was then concentrated under reduced pressure. The crude mixture was purified by prep-TLC using PE/Et_2O 95:5 to afford **3a** as an oil (13.5 mg, 35.8 μmol, 46% yield).

$[\alpha]_D^{23}$ = 56.4 (c = 0.0333, $CHCl_3$).

The enantiomeric ratio was determined to be 97:3 by SFC analysis: IA column, 15% MeOH in supercritical CO_2, flow rate 2.0 mL/min, λ = 214.4 nm. tR(major) = 22.9 min, tR(minor) = 26.9 min.

Benzyl 2-((2-bromophenyl)sulfonyl)cyclobutane-1-carboxylate (5)

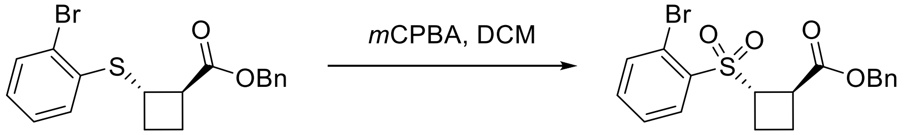

3a 97:3 er

5, 95% 97:3 er

Racemic: Cyclobutane **3a** (37.7 mg, 100 μmol, 1.00 equiv.) was diluted in DCM (1.00 mL) and *m*CPBA (56.0 mg, 250 μmol, 2.50 equiv.) was then added at 0 °C. The reaction was stirred at rt for 3 h. The reaction was then diluted in DCM (10 mL) and washed with sat. $NaHCO_3$ solution (10 mL). The combined organic layers were dried over $MgSO_4$, filtered, and concentrated under reduced pressure. The crude product **5** (40.0 mg, 97.7 μmol, 98% yield) was pure enough without further purification.

Enantioenriched: the same procedure as described above was implemented on enantioenriched **3a** (15.1 mg, 40.0 μmol, 1.00 equiv.) to afford **5** as an oil (10.3 mg, 25.2 μmol, 95% yield).

$[\alpha]_D^{23}$ = 7.3 (c = 0.0400, $CHCl_3$).
^{1}H NMR (400 MHz, chloroform-*d*) δ 8.10 (dd, *J* = 7.7, 1.9 Hz, 1H, Ar*H*), 7.63 (dd, *J* = 7.8, 1.3 Hz, 1H, Ar*H*), 7.46 – 7.32 (m, 5H, Ar*H*), 7.19 (dd, *J* = 6.6, 3.0 Hz, 2H, Ar*H*), 4.99 – 4.87 (m, 2H, OCH_2Ph), 4.83 – 4.70 (m, 1H, SC*H*), 3.67 – 3.53 (m, 1H, C*H*C(O)), 2.71 – 2.54 (m, 1H, SCHCH_2), 2.43 – 2.12 (m, 3H, CH_2CHC(O) + SCHCH_2).
^{13}C NMR (101 MHz, chloroform-*d*) δ 171.5, 137.0, 135.6, 135.4, 134.8, 132.6, 128.7, 128.5, 128.4, 127.9, 121.4, 67.0, 56.0, 39.1, 21.1, 18.9.

IR (ν_{max}, cm^{-1}) 3069 (w), 2957 (w), 1733 (s), 1573 (w), 1447 (m), 1314 (s), 1249 (m), 1199 (m), 1150 (s), 1026 (m), 737 (s).
HRMS (nanochip-ESI/LTQ-Orbitrap) m/z: $[M + H]^+$ Calcd for $C_{18}H_{18}BrO_4S^+$ 409.0104; Found 409.0120.

The enantiomeric ratio was determined to be 97:3 by SFC analysis: IC column, 7% MeCN in supercritical CO_2, flow rate 0.75 mL/min, λ = 270.4 nm. tR(major) = 65.4 min, tR(minor) = 70.3 min.

Methyl 2-((2-bromophenyl)thio)cyclobutane-1-carboxylate (6)

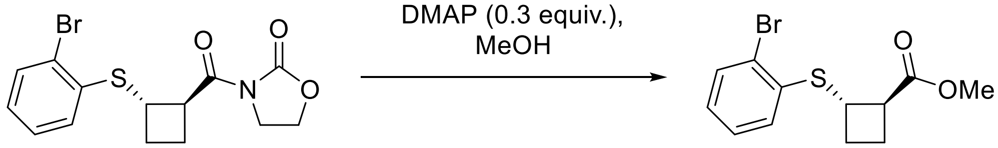

4a
97:3 er

8, 95%
97:3 er

Racemic: Cyclobutane **4a** (35.6 mg, 100 μmol, 1.00 equiv.) was diluted in dry MeOH (1.0 mL) and DMAP (3.67 mg, 30.0 μmol, 0.300 equiv.) was added. The reaction mixture was stirred at rt for 2 h. The mixture was then concentrated under reduced pressure. The crude mixture was purified by prep-TLC using PE/Et_2O 90:10 to afford **8** as an oil (10.5 mg, 34.9 μmol, 87% yield).

Enantioenriched: the same procedure as described above was implemented on enantioenriched **4a** (35.6 mg, 100 μmol, 1.00 equiv.) to afford **8** as an oil (11.4 mg, 37.8 μmol, 95% yield).

Rf(PE/Et_2O 9:1): 0.37.
$[\alpha]_D^{23}$ = 91.3 (c = 0.0100, $CHCl_3$).
^1H NMR (400 MHz, chloroform-*d*) δ 7.54 (d, *J* = 8.0 Hz, 1H, Ar*H*), 7.36 – 7.17 (m, 2H, Ar*H*), 7.03 (dt, *J* = 8.4, 4.5 Hz, 1H, Ar*H*), 4.18 (q, *J* = 8.3 Hz, 1H, SC*H*), 3.66 (s, 3H, OCH_3), 3.19 (q, *J* = 8.8 Hz, 1H, C*H*C(O)), 2.56 – 2.43 (m, 1H, SCHCH_2), 2.40 – 2.28 (m, 1H, CH_2CHC(O)), 2.22 (p, *J* = 9.2 Hz, 1H, CH_2CHC(O)), 2.16 – 1.91 (m, 1H, SCHCH_2).
^{13}C NMR (101 MHz, chloroform-*d*) δ 173.9, 137.2, 133.2, 129.3, 127.9, 127.1, 123.7, 52.1, 45.9, 41.7, 27.2, 22.6.
IR (ν_{max}, cm^{-1}) 3059 (w), 2993 (w), 2951 (m), 2922 (m), 2852 (w), 1732 (s), 1574 (w), 1448 (m), 1361 (m), 1247 (m), 1210 (m), 1165 (m), 1039 (m), 1022 (m).
HRMS (ESI/QTOF) m/z: $[M + Na]^+$ Calcd for $C_{12}H_{13}BrNaO_2S^+$ 322.9712; Found 322.9716.

The enantiomeric ratio was determined to be 97:3 by SFC analysis: IC column, 6% MeOH in supercritical CO_2, flow rate 2.0 mL/min, λ = 214.4 nm. tR(major) = 1.3 min, tR(minor) = 1.5 min.

***tert*-Butyl 2-((2-bromophenyl)thio)cyclobutane-1-carboxylate (7)**

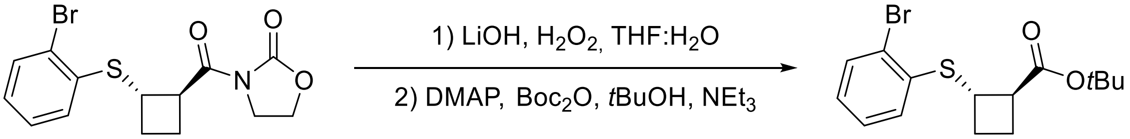

4a
97:3 er

9, 42%
97:3 er

Racemic: Cyclobutane **4a** (71.2 mg, 200 µmol, 1.00 equiv.) was diluted in THF (1.3 mL) and H_2O (0.7 mL). LiOH monohydrate (16.8 mg, 400 µmol, 2.00 equiv.) and hydrogen peroxide 30w% (136 mg, 123 µL, 1.20 mmol, 6.00 equiv) were added at 0 °C. The reaction mixture was stirred at rt for 18 h. The reaction mixture was quenched with sat. Na_2SO_3 (1 mL), diluted with sat. $NaHCO_3$ (10 mL), washed with EtOAc (10 mL). The pH value of the aqueous layer was adjusted to 1 using HCl (1 M). The mixture was extracted with EtOAc (3 x 20 mL), dried over $MgSO_4$, filtered, and concentrated under reduced pressure.

To the crude mixture was added DMAP (2.44 mg, 20.0 µmol, 0.100 equiv.), Boc_2O (65.5 mg, 300 µmol, 1.50 equiv.), NEt_3 (40.5 mg, 55.8 µL, 400 µmol, 2.00 equiv.) and *t*BuOH (29.6 mg, 38.0 µL, 400 µmol, 2.00 equiv.) in this order. The reaction was stirred neat at rt for 18 h. The reaction mixture was diluted with DCM (10 mL), washed with brine (2 x 10 mL), dried over $MgSO_4$, filtered, and concentrated under reduced pressure. The crude mixture was purified by prep-TLC using PE/Et_2O 90:10 to afford **9** as an oil (31.6 mg, 92.1 µmol, 46% yield).

Enantioenriched: the same procedure as described above was implemented on enantioenriched **4a** (71.2 mg, 200 µmol, 1.00 equiv.) to afford **11** as an oil (28.9 mg, 84.2 µmol, 42% yield).

Rf(PE/EtOAc 9:1): 0.85.
$[\alpha]_D^{23}$ = 37.8 (c = 0.0270, $CHCl_3$).
^{1}H NMR (400 MHz, chloroform-*d*) δ 7.53 (dd, *J* = 7.9, 1.3 Hz, 1H, Ar*H*), 7.37 – 7.14 (m, 2H, Ar*H*), 7.02 (ddd, *J* = 7.9, 7.3, 1.8 Hz, 1H, Ar*H*), 4.14 (q, *J* = 8.2 Hz, 1H, SC*H*), 3.14 – 3.01 (m, 1H, C*H*C(O)), 2.54 – 2.41 (m, 1H, SCHCH_2), 2.41 – 2.24 (m, 1H, CH_2CHC(O)), 2.22 – 1.95 (m, 2H, SCHCH_2 + CH_2CHC(O)), 1.42 (s, 9H, C(CH_3)$_3$).
^{13}C NMR (101 MHz, chloroform-*d*) δ 172.9, 137.6, 133.1, 129.2, 127.9, 126.9, 123.5, 81.0, 47.5, 41.4, 28.1, 26.7, 22.5.
IR (ν_{max}, cm^{-1}) 3062 (w), 2978 (m), 2938 (w), 2873 (w), 1721 (s), 1451 (m), 1367 (m), 1249 (m), 1151 (s), 1021 (w), 844 (w).
HRMS (ESI/QTOF) m/z: $[M + Na]^+$ Calcd for $C_{15}H_{19}BrNaO_2S^+$ 365.0181; Found 365.0190.

The enantiomeric ratio was determined to be 97:3 by SFC analysis: IA column, 1% MeCN in supercritical CO_2, flow rate 0.75 mL/min, λ = 214.4 nm. tR(major) = 7.1 min, tR(minor) = 8.3 min.

(2-((2-Bromophenyl)thio)cyclobutyl)methanol (8)

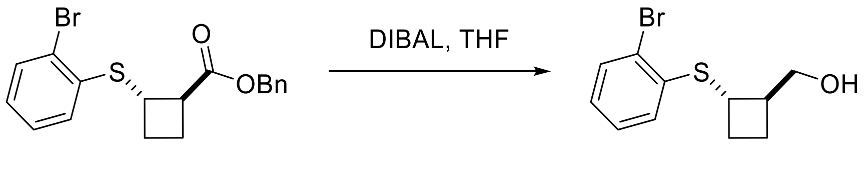

3a **7**, quant.

Racemic: Cyclobutane **3a** (37.7 mg, 100 µmol, 1.00 equiv.) was diluted in dry THF (2.50 mL) and the mixture was cooled to 0 °C. Diisobutylaluminium hydride (56.9 mg, 400 µL, 400 µmol, 1 M in toluene, 4.00 equiv.) was added dropwise and the reaction mixture was stirred at room temperature for 10 minutes. The reaction was diluted with ether and cooled to 0°C, 400 µL of water were added, followed by 400 µL of 15 % sodium hydroxide and 900 µL of water. The mixture was warmed to room temperature and stirred for 15 min. $MgSO_4$ was added and the mixture stirred for an additional 15 min. The mixture was filtered and concentrated under reduced pressure. The crude product was purified by prep-TLC using PE/EtOAc 60:40 to afford **7** as an oil (27.2 mg, 99.6 µmol, quant.).

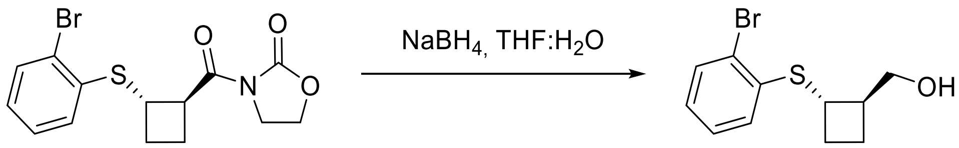

3a
97:3 er

7, 92%
97:3 er

Enantioenriched: To a solution of cyclobutane **4a** (35.6 mg, 100 μmol, 1.00 equiv.) in THF (0.8 mL) and H_2O (0.2 mL) was added $NaBH_4$ (15.1 mg, 400 μmol, 4.00 equiv.). The reaction was stirred at rt for 2 h. The reaction mixture was quenched with sat. NH_4Cl (10 mL), extracted with EtOAc (3 x 10 mL) dried over $MgSO_4$, filtered, and concentrated under reduced pressure. The crude product was purified by prep-TLC using PE/EtOAc 60:40 to afford **7** as an oil (25.1 mg, 91.9 μmol, 92% yield).

Rf(PE/EtOAc 6:4): 0.63.
$[\alpha]_D^{23}$ = 129.3 (c = 0.0700, $CHCl_3$).
^1H NMR (400 MHz, chloroform-*d*) δ 7.53 (d, *J* = 7.9 Hz, 1H, Ar*H*), 7.26 – 7.23 (m, 2H, Ar*H*), 7.01 (dp, *J* = 8.4, 4.1 Hz, 1H, Ar*H*), 3.76 (q, *J* = 8.0 Hz, 1H, SC*H*), 3.69 (t, *J* = 4.5 Hz, 2H, C*H₂*OH), 2.60 (ddt, *J* = 13.7, 8.4, 4.2 Hz, 1H, SCHC*H*), 2.55 – 2.43 (m, 1H, SCHC*H₂*), 2.14 (qd, *J* = 9.0, 3.1 Hz, 1H, SCHCH₂C*H₂*), 2.02 (dq, *J* = 11.2, 8.9 Hz, 1H, SCHC*H₂*), 1.92 – 1.79 (m, 1H, SCHCH₂C*H₂*), 1.41 (t, *J* = 5.0 Hz, 1H, O*H*).
^{13}C NMR (101 MHz, chloroform-*d*) δ 138.3, 133.1, 128.6, 127.9, 126.7, 123.3, 64.7, 45.0, 41.0, 28.1, 21.1.
IR (ν_{max}, cm^{-1}) 3372 (m), 2979 (m), 2938 (m), 2863 (m), 1574 (w), 1448 (s), 1429 (m), 1253 (m), 1112 (m), 1067 (m), 1022 (s).
HRMS (ESI/QTOF) m/z: $[M + Na]^+$ Calcd for $C_{11}H_{13}BrNaOS^+$ 294.9763; Found 294.9763.

The enantiomeric ratio was determined to be 97:3 by SFC analysis: IA column, 6% MeOH in supercritical CO_2, flow rate 0.50 mL/min, λ = 270.4 nm. tR(major) = 35.6 min, tR(minor) = 41.3 min.

Enantioenriched 2-((2-Bromophenyl)thio)-N-methoxy-N-methylcyclobutane-1-carboxamide (3z)

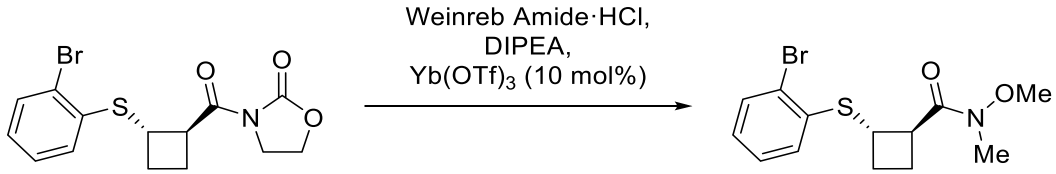

4a,
er 97:3

3z, 62%
er 97:3

Racemic: The racemic compound was described above as **3z** (page 25).

Enantioenriched: Cyclobutane **4a** (17.8 mg, 50.0 μmol, 1.00 equiv.), methoxy(methyl)amine;hydrochloride (14.6 mg, 150 μmol, 3.00 equiv.), and $Yb(OTf)_3$ (3.10 mg, 5.00 μmol, 0.100 equiv.) were diluted in dry MeCN (0.5 mL) and DIPEA (19.4 mg, 26.1 μL, 150 μmol, 3.00 equiv.) was added. The reaction mixture was stirred at 90 °C for 48 h. The mixture was then concentrated under reduced pressure. The crude mixture was purified by prep-TLC using PE/EtOAc 80:20 to afford **3z** as an oil (10.2 mg, 30.9 μmol, 62% yield).

$[\alpha]_D^{23}$ = 62.7 (c = 0.0110, $CHCl_3$).

The enantiomeric ratio was determined to be 97:3 by SFC analysis: IC column, 10% MeOH in supercritical CO_2, flow rate 2.0 mL/min, λ = 214.4 nm. tR(major) = 2.3 min, tR(minor) = 2.1 min.

1-(2-((2-Bromophenyl)thio)cyclobutyl)ethan-1-one (9)

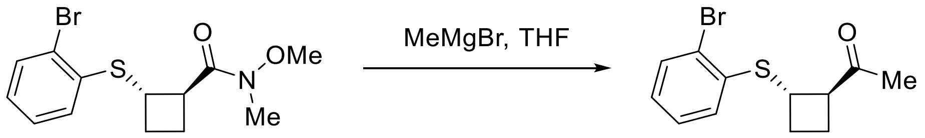

3z
97:3 er

10, 91%
97:3 er

Racemic: Cyclobutane **3z** (66.0 mg, 200 µmol, 1.00 equiv.) was diluted in dry THF (2.0 mL) and MeMgBr (28.6 mg, 80.0 µL, 240 µmol, 3.00M in Et_2O, 1.20 equiv.) was added at -78 °C. The reaction mixture was stirred at 0 °C for 1 h. The reaction mixture was quenched with sat. NH_4Cl (10 mL), extracted with EtOAc (3 x 10 mL), dried over $MgSO_4$, filtered, and concentrated under reduced pressure to afford **10** as an oil (52.4 mg, 184 µmol, 92% yield) which was pure enough without further purification.

Enantioenriched: the same procedure as described above was implemented on enantioenriched **3z** (7.90 mg, 23.9 µmol, 1.00 equiv.) to afford **10** as an oil (6.20 mg, 21.7 µmol, 91% yield).

$[\alpha]_D^{23}$ = 60.7 (c = 0.0567, $CHCl_3$).
^{1}H NMR (400 MHz, chloroform-*d*) δ 7.62 – 7.46 (m, 1H, Ar*H*), 7.32 – 7.16 (m, 2H, Ar*H*), 7.03 (ddd, *J* = 8.0, 6.6, 2.3 Hz, 1H, Ar*H*), 4.12 (q, *J* = 8.0 Hz, 1H, SC*H*), 3.32 (q, *J* = 8.3 Hz, 1H, C*H*C(O)), 2.50 – 2.39 (m, 1H, SCHCH_2), 2.39 – 2.23 (m, 1H, CH_2CHC(O)), 2.21 – 1.96 (m, 5H, CH_3 + SCHCH_2 + CH_2CHC(O)).
^{13}C NMR (101 MHz, chloroform-*d*) δ 207.5, 137.4, 133.2, 129.1, 128.0, 127.1, 123.4, 53.0, 40.3, 28.3, 26.6, 21.8.
IR (ν_{max}, cm^{-1}) 3059 (w), 2947 (m), 2862 (w), 2098 (w), 1744 (m), 1710 (s), 1476 (m), 1448 (s), 1430 (m), 1361 (m), 1252 (m), 1188 (m), 1022 (m), 850 (m).
HRMS (nanochip-ESI/LTQ-Orbitrap) m/z: $[M + H]^+$ Calcd for $C_{12}H_{14}BrOS^+$ 284.9943; Found 284.9948.

The enantiomeric ratio was determined to be 97:3 by chiral HPLC analysis: IA column, 99:1 hexane/isopropanol, flow rate 1.0 mL/min, λ = 214.4 nm. tR(major) = 9.6 min, tR(minor) = 10.7 min.

2-[(2-Bromophenyl)thio]cyclobutanecarboxylic acid (10)

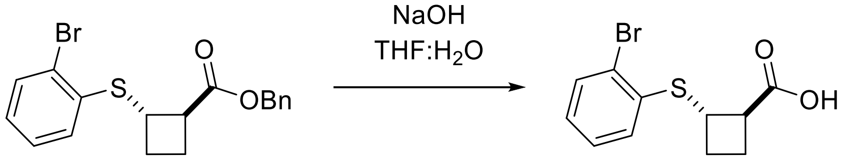

3a

6, quant.

To a solution of cyclobutane **3a** (113 mg, 300 µmol, 1.00 equiv.) in THF (1.20 mL) and water (1.20 mL) was added sodium hydroxide (48.0 mg, 1.20 mmol, 4.00 equiv.). The reaction was stirred at rt for 4 h. The mixture was washed with EtOAc (3 x 20 mL). The pH value of the

aqueous layer was adjusted to 1 using HCl (1 M). The mixture was extracted with EtOAc (3 x 20 mL), dried over $MgSO_4$, filtered, and concentrated under reduced pressure to afford **6** (86.0 mg, 299 μmol, quant.) as an amorphous solid that was pure enough without further purification.

^{1}H NMR (400 MHz, chloroform-*d*) δ 7.55 (d, *J* = 8.1 Hz, 1H, Ar*H*), 7.30 – 7.20 (m, 2H, Ar*H*), 7.09 – 6.95 (m, 1H, Ar*H*), 4.18 (q, *J* = 8.2 Hz, 1H, SC*H*), 3.22 (q, *J* = 8.7 Hz, 1H, C*H*C(O)), 2.52 (ddt, *J* = 11.8, 8.7, 4.4 Hz, 1H, SCHCH_2), 2.40 (qd, *J* = 9.4, 3.5 Hz, 1H, CH_2CHC(O)), 2.32 – 2.18 (m, 1H, CH_2CHC(O)), 2.09 (dq, *J* = 11.3, 9.1 Hz, 1H, SCHCH_2).
^{13}C NMR (101 MHz, chloroform-*d*) δ 178.6, 136.9, 133.2, 129.6, 128.0, 127.4, 124.0, 45.4, 41.5, 27.2, 22.6.
IR (v_{max}, cm^{-1}) 3059 (m), 2953 (m), 1704 (s), 1559 (w), 1451 (s), 1422 (m), 1260 (m), 1224 (m), 1019 (m), 937 (w), 910 (w), 744 (s).
HRMS (ESI/QTOF) m/z: $[M + H_{-1}]^-$ Calcd for $C_{11}H_{10}BrO_2S^-$ 284.9590; Found 284.9586.

1.8. Proposed model for stereoinduction

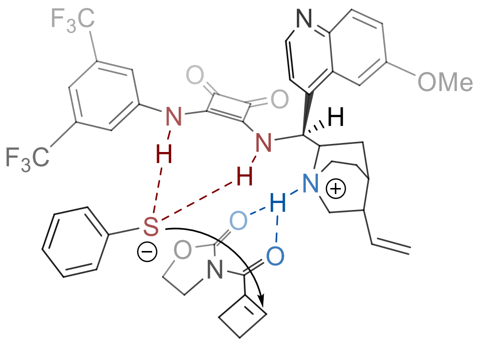

Figure S1: Proposed stereoinduction model applying Houk's Brønsted acid-hydrogen bonding model.[7]

[7] a) M. N. Grayson, K. N. Houk, *J. Am. Chem. Soc.* **2016**, *138*, 1170–1173; b) J. Guo, M. W. Wong, *J. Org. Chem.* **2017**, *82*, 4362–4368.

2. References

[1] E. G. L. Robert, V. Pirenne, M. D. Wodrich, J. Waser, *Angew. Chem. Int. Ed.* **2023**, *62*, e202302420.

[2] A. A. Homon, O. V. Hryshchuk, S. Trofymchuk, O. Michurin, Y. Kuchkovska, D. S. Radchenko, O. O. Grygorenko, *Eur. J. Org. Chem.* **2018**, *2018*, 5596–5604.

[3] E. G. L. Robert, J. Waser, *Chem. Eur. J.* **2025**, *31*, e202403986.

[4] H. Xu, W. Zhang, D. Shu, J. B. Werness, W. Tang, *Angew. Chem. Int. Ed.* **2008**, *47*, 8933–8936.

[5] J. M. Robinson, S. F. Tlais, J. Fong, R. L. Danheiser, *Tetrahedron* **2011**, *67*, 9890–9898.

[6] L. Ghisu, N. Melis, L. Serusi, A. Luridiana, F. Soddu, F. Secci, P. Caboni, R. Guillot, D. J. Aitken, A. Frongia, *Org. Biomol. Chem.* **2019**, *17*, 6143–6147.

[7] a) M. N. Grayson, K. N. Houk, *J. Am. Chem. Soc.* **2016**, *138*, 1170–1173; b) J. Guo, M. W. Wong, *J. Org. Chem.* **2017**, *82*, 4362–4368.

3. X-rays crystallographic data

3.1 Compound **3’l** (CCDC number 2383043)

The crystal suitable for X-ray measurement for compound **3’l** was obtained by evaporation of dichloromethane.

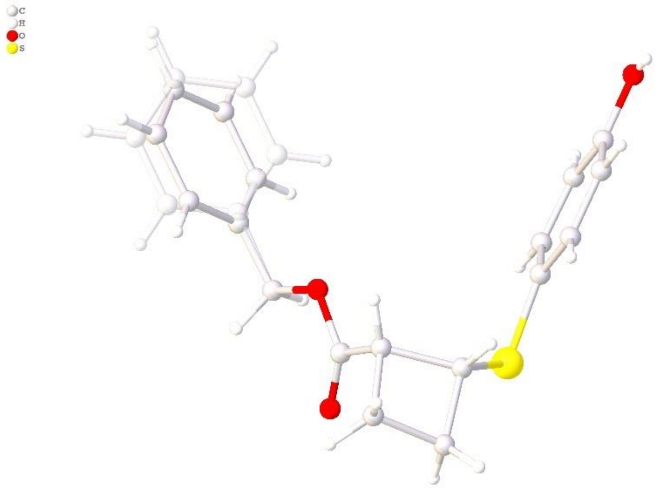

Experimental. Single clear pale colourless prism-shaped crystals of **6x** were used as supplied. A suitable crystal with dimensions 0.22 × 0.04 × 0.04 mm^3 was selected and mounted on a XtaLAB Synergy R, DW system, HyPix-Arc 150 diffractometer. The crystal was kept at a steady T = 139.98(10) K during data collection. The structure was solved with the ShelXT (Sheldrick, 2015) solution program using dual methods and by using Olex2 1.5 (Dolomanov et al., 2009) as the graphical interface. The model was refined with ShelXL 2019/3 (Sheldrick, 2015) using full matrix least squares minimisation on $\boldsymbol{F^2}$.

**Compound**	**3'l**
Formula	$C_{18}H_{18}O_3S$
$D_{calc.}$/ g cm^{-3}	1.317
μ/mm^{-1}	1.896
Formula Weight	314.38
Colour	clear pale colourless
Shape	prism
Size/mm^3	0.22×0.04×0.04
T/K	139.98(10)
Crystal System	monoclinic
Space Group	$C2/c$
a/Å	19.0857(3)
b/Å	5.32972(8)
c/Å	31.1946(4)
α/°	90
β/°	92.5193(14)
γ/°	90
V/Å^3	3170.09(8)
Z	8
Z'	1
Wavelength/Å	1.54184
Radiation type	Cu K$_\alpha$
Θ_{min}/°	2.836
Θ_{max}/°	75.774
Measured Refl's.	31855
Indep't Refl's	3263
Refl's I≥2 σ(I)	2815
R_{int}	0.0363
Parameters	274
Restraints	0
Largest Peak	0.303
Deepest Hole	-0.291
GooF	1.040
wR_2 (all data)	0.0997
wR_2	0.0948
R_1 (all data)	0.0438
R_1	0.0365

Table 1: Fractional Atomic Coordinates (×10^4) and Equivalent Isotropic Displacement Parameters (Å^2×10^3) for **3'l**. U_{eq} is defined as 1/3 of the trace of the orthogonalised U_{ij}.

Atom	x	y	z	U_{eq}
S1	2099.9(2)	6857.5(7)	3004.5(2)	32.07(12)
O1	4937.3(6)	5359(2)	3807.5(4)	38.7(3)
O2	527.5(6)	6121(2)	3479.3(4)	39.2(3)
O3	1215.1(6)	4102(2)	3969.5(3)	35.5(3)
C1	1674.4(8)	3880(3)	2914.1(5)	29.3(3)
C2	1020.1(8)	3983(3)	2604.3(5)	32.2(3)
C3	678.4(8)	2013(3)	2890.1(5)	32.7(3)
C4	1218.5(8)	2622(3)	3260.7(5)	30.0(3)
C5	2926.4(7)	6150(3)	3257.6(5)	28.4(3)
C6	3345.5(8)	4157(3)	3139.4(5)	32.7(3)
C7	4012.9(8)	3850(3)	3321.2(5)	31.9(3)
C8	4279.0(8)	5561(3)	3620.0(5)	31.3(3)
C9	3869.2(9)	7567(3)	3737.8(6)	42.9(4)
C10	3194.6(9)	7830(3)	3562.1(6)	39.5(4)
C11	945.2(7)	4470(3)	3574.0(5)	30.7(3)
C12	937.0(10)	5709(4)	4302.5(5)	45.3(4)

Atom	x	y	z	U_{eq}
C13	1139.4(15)	4712(6)	4709.1(7)	33.7(15)
C14	1604.2(18)	6034(6)	4980.1(7)	43.1(10)
C15	1815.5(18)	5037(7)	5376.7(7)	47.7(11)
C16	1562.1(18)	2718(6)	5502.3(8)	51.6(18)
C17	1097(2)	1396(5)	5231.3(11)	47.2(12)
C18	885.9(18)	2393(6)	4834.7(11)	44.4(10)
C19	1197.3(14)	4485(6)	4743.3(7)	41.6(19)
C24	728.9(11)	3065(8)	4970.9(11)	41.2(10)
C23	946.3(14)	1962(7)	5358.0(11)	42.7(11)
C22	1632.2(16)	2278(6)	5517.5(8)	39.0(13)
C21	2100.6(14)	3698(7)	5289.9(9)	43.4(10)
C20	1883.1(14)	4802(7)	4902.8(8)	42.1(10)

Table 2: Anisotropic Displacement Parameters (×10[4]) for **3'l.** The anisotropic displacement factor exponent takes the form: $-2\pi^2[h^2a^{*2} \times U_{11}+ ... +2hka^* \times b^* \times U_{12}]$

Atom	U_{11}	U_{22}	U_{33}	U_{23}	U_{13}	U_{12}
S1	28.1(2)	25.4(2)	42.6(2)	1.40(15)	-0.05(14)	3.13(13)
O1	29.4(6)	35.2(6)	50.9(7)	-4.0(5)	-3.2(5)	4.0(5)
O2	40.0(6)	37.6(6)	40.2(6)	2.0(5)	5.0(5)	13.8(5)
O3	36.1(6)	38.6(6)	31.8(5)	-4.7(5)	-0.2(4)	7.5(5)
C1	26.9(7)	27.0(7)	34.1(8)	-1.1(6)	3.5(6)	2.3(6)
C2	32.4(8)	32.9(8)	31.4(8)	0.0(6)	1.3(6)	0.5(6)
C3	30.7(8)	31.6(8)	35.8(8)	-0.1(6)	1.2(6)	-1.2(6)
C4	30.0(7)	28.1(7)	32.0(7)	2.2(6)	1.5(6)	3.4(6)
C5	26.5(7)	24.6(7)	34.2(7)	2.1(6)	4.2(6)	1.1(5)
C6	34.8(8)	30.2(8)	33.1(8)	-4.5(6)	1.0(6)	4.5(6)
C7	32.6(8)	28.8(7)	34.4(8)	-1.8(6)	2.4(6)	7.4(6)
C8	27.1(7)	27.9(7)	39.0(8)	2.1(6)	1.6(6)	0.5(6)
C9	34.6(9)	30.8(8)	62.7(11)	-15.8(8)	-5.2(8)	2.4(7)
C10	33.1(8)	28.2(8)	56.8(10)	-10.5(7)	-0.7(7)	5.0(6)
C11	27.2(7)	30.5(7)	34.6(8)	2.1(6)	2.2(6)	1.7(6)
C12	51.8(10)	45.1(10)	39.3(9)	-9.2(8)	5.0(7)	12.3(8)
C13	32(3)	35(3)	34(3)	-7(2)	5(2)	4(2)
C14	51(2)	46(2)	33.3(17)	-6.9(15)	7.1(15)	-3.8(19)
C15	54(2)	57(3)	32.2(18)	-8.9(17)	1.0(15)	-7(2)
C16	72(4)	55(3)	28(3)	-1(2)	7(3)	9(3)
C17	54(3)	46(2)	43(3)	0.8(19)	12(2)	-2(2)
C18	45(2)	44(2)	45(3)	-7.9(18)	3.4(18)	0.2(18)
C19	52(4)	46(4)	27(3)	-4(3)	-1(3)	16(3)
C24	37(2)	48(2)	39(2)	-5.4(18)	1.8(16)	-0.1(17)
C23	49(2)	44(2)	36(2)	0.6(18)	6.8(18)	0.9(19)
C22	45(3)	38(2)	34(3)	-1.3(19)	2(2)	3(2)
C21	46(2)	43(2)	40.5(19)	0.0(16)	-6.7(16)	3.6(18)
C20	45(2)	42(2)	39.9(19)	2.6(16)	3.7(15)	1.7(17)

Table 3: Bond Lengths in Å for **3'l.**

Atom	Atom	Length/Å
S1	C1	1.7992(15)
S1	C5	1.7730(15)
O1	C8	1.3667(18)
O2	C11	1.2149(18)
O3	C11	1.3304(18)
O3	C12	1.4640(19)
C1	C2	1.546(2)

Atom	Atom	Length/Å
C1	C4	1.568(2)
C2	C3	1.541(2)
C3	C4	1.549(2)
C4	C11	1.497(2)
C5	C6	1.389(2)
C5	C10	1.387(2)
C6	C7	1.381(2)

Atom	Atom	Length/Å
C7	C8	1.384(2)
C8	C9	1.384(2)
C9	C10	1.384(2)
C12	C13	1.413(3)
C12	C19	1.582(3)
C13	C14	1.3900
C13	C18	1.3900
C14	C15	1.3900
C15	C16	1.3900
C16	C17	1.3900
C17	C18	1.3900
C19	C24	1.3900
C19	C20	1.3900
C24	C23	1.3900
C23	C22	1.3900
C22	C21	1.3900
C21	C20	1.3900

Table 4: Bond Angles in ° for **3'l.**

Atom	Atom	Atom	Angle/°
C5	S1	C1	105.59(7)
C11	O3	C12	115.72(12)
C2	C1	S1	114.41(11)
C2	C1	C4	89.35(11)
C4	C1	S1	121.97(10)
C3	C2	C1	87.99(11)
C2	C3	C4	90.24(11)
C3	C4	C1	86.93(11)
C11	C4	C1	112.88(12)
C11	C4	C3	112.68(12)
C6	C5	S1	123.76(12)
C10	C5	S1	117.51(11)
C10	C5	C6	118.43(14)
C7	C6	C5	120.86(14)
C6	C7	C8	120.19(14)
O1	C8	C7	122.64(14)
O1	C8	C9	117.84(14)
C9	C8	C7	119.52(14)
C8	C9	C10	120.01(15)
C9	C10	C5	120.97(15)
O2	C11	O3	123.76(14)
O2	C11	C4	124.13(13)
O3	C11	C4	112.11(12)
O3	C12	C19	105.41(16)
C13	C12	O3	108.88(17)
C14	C13	C12	119.8(2)
C14	C13	C18	120.0
C18	C13	C12	120.2(2)
C13	C14	C15	120.0
C14	C15	C16	120.0
C15	C16	C17	120.0
C18	C17	C16	120.0
C17	C18	C13	120.0
C24	C19	C12	118.95(19)
C24	C19	C20	120.0
C20	C19	C12	121.05(19)
C19	C24	C23	120.0
C22	C23	C24	120.0
C21	C22	C23	120.0
C22	C21	C20	120.0
C21	C20	C19	120.0

Table 5: Torsion Angles in ° for **3'l.**

Atom	Atom	Atom	Atom	Angle/°
S1	C1	C2	C3	-142.77(11)
S1	C1	C4	C3	136.19(12)
S1	C1	C4	C11	22.83(17)
S1	C5	C6	C7	173.69(12)
S1	C5	C10	C9	-172.51(14)
O1	C8	C9	C10	-178.94(16)
O3	C12	C13	C14	113.4(2)
O3	C12	C13	C18	-65.3(3)
O3	C12	C19	C24	-102.1(2)
O3	C12	C19	C20	77.6(3)
C1	S1	C5	C6	41.02(15)
C1	S1	C5	C10	-145.38(13)
C1	C2	C3	C4	17.80(11)
C1	C4	C11	O2	65.42(19)
C1	C4	C11	O3	-114.37(14)
C2	C1	C4	C3	17.50(11)
C2	C1	C4	C11	-95.86(13)
C2	C3	C4	C1	-17.56(11)
C2	C3	C4	C11	95.99(14)
C3	C4	C11	O2	-31.1(2)

Atom	Atom	Atom	Atom	Angle/°
C3	C4	C11	O3	149.13(13)
C4	C1	C2	C3	-17.58(11)
C5	S1	C1	C2	-166.56(10)
C5	S1	C1	C4	87.87(12)
C5	C6	C7	C8	-1.2(2)
C6	C5	C10	C9	1.4(3)
C6	C7	C8	O1	-179.48(14)
C6	C7	C8	C9	0.6(2)
C7	C8	C9	C10	1.0(3)
C8	C9	C10	C5	-2.0(3)
C10	C5	C6	C7	0.1(2)
C11	O3	C12	C13	165.89(18)
C11	O3	C12	C19	166.77(16)
C12	O3	C11	O2	4.6(2)
C12	O3	C11	C4	-175.65(13)
C12	C13	C14	C15	-178.7(3)
C12	C13	C18	C17	178.7(3)
C12	C19	C24	C23	179.7(3)
C12	C19	C20	C21	-179.7(3)
C13	C14	C15	C16	0.0
C14	C13	C18	C17	0.0
C14	C15	C16	C17	0.0
C15	C16	C17	C18	0.0
C16	C17	C18	C13	0.0
C18	C13	C14	C15	0.0
C19	C24	C23	C22	0.0
C24	C19	C20	C21	0.0
C24	C23	C22	C21	0.0
C23	C22	C21	C20	0.0
C22	C21	C20	C19	0.0
C20	C19	C24	C23	0.0

Table 6: Hydrogen Fractional Atomic Coordinates (×10⁴) and Equivalent Isotropic Displacement Parameters (Å²×10³) for **3'l**. U_{eq} is defined as 1/3 of the trace of the orthogonalised U_{ij}.

Atom	x	y	z	U_{eq}
H1	5101(13)	4080(50)	3720(8)	74(8)
H1A	2012(9)	2720(30)	2807(5)	34(4)
H2A	755(10)	5630(40)	2625(6)	49(5)
H2B	1099(9)	3590(30)	2302(6)	36(5)
H3A	185(10)	2290(30)	2956(5)	36(5)
H3B	741(9)	300(40)	2788(5)	38(5)
H4	1464(9)	1240(30)	3414(5)	33(4)
H6	3178(10)	2980(40)	2929(6)	43(5)
H7	4289(10)	2540(40)	3249(6)	45(5)
H9	4055(11)	8730(40)	3944(7)	58(6)
H10	2911(11)	9190(40)	3656(6)	54(6)
H12A	1122.74	7433.43	4275.54	54
H12B	419.12	5782.09	4269.47	54
H12C	418.24	5757.4	4277.95	54
H12D	1118.48	7441.05	4278.45	54
H14	1777.46	7619.17	4894.22	52
H15	2133.23	5940.34	5561.91	57
H16	1706.49	2036.19	5773.4	62
H17	923.97	-189.14	5317.21	57
H18	568.19	1489.65	4649.51	53
H24	260.15	2848.7	4861.96	49
H23	626.19	990.96	5513.59	51
H22	1780.77	1524.01	5782.04	47

Atom	x	y	z	U_{eq}
H21	2569.32	3914.78	5398.86	52
H20	2203.29	5772.53	4747.23	51

Table 7: Hydrogen Bond information for **3'l.**

D	H	A	d(D-H)/Å	d(H-A)/Å	d(D-A)/Å	D-H-A/deg
O1	H1	$O2^{1}$	0.80(3)	1.94(3)	2.7424(17)	176(3)

––––

^{1}1/2+x,-1/2+y,+z

Table 8: Atomic Occupancies for all atoms that are not fully occupied in **3'l**.

Atom	Occupancy	Atom	Occupancy	Atom	Occupancy
H12A	0.503(5)	C16	0.503(5)	C23	0.497(5)
H12B	0.503(5)	H16	0.503(5)	H23	0.497(5)
H12C	0.497(5)	C17	0.503(5)	C22	0.497(5)
H12D	0.497(5)	H17	0.503(5)	H22	0.497(5)
C13	0.503(5)	C18	0.503(5)	C21	0.497(5)
C14	0.503(5)	H18	0.503(5)	H21	0.497(5)
H14	0.503(5)	C19	0.497(5)	C20	0.497(5)
C15	0.503(5)	C24	0.497(5)	H20	0.497(5)
H15	0.503(5)	H24	0.497(5)		

3.2 Compound **4b** (CCDC number 2415842)

The crystal suitable for X-ray measurement for compound **4b** was obtained by evaporation of dichloromethane.

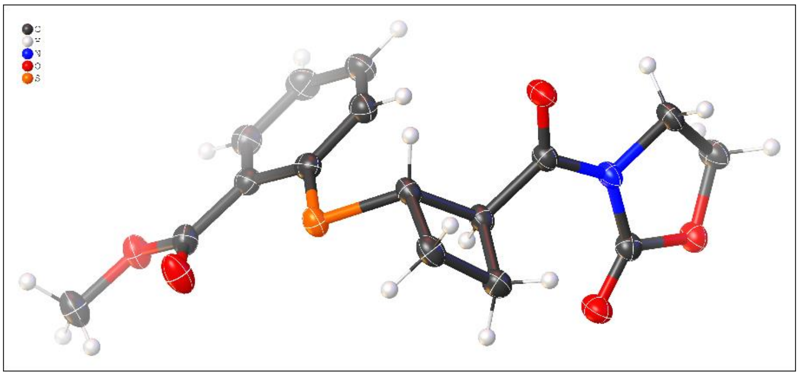

Experimental. Single colourless plate-shaped crystals of **4b** were used as supplied. A suitable crystal with dimensions 0.09 × 0.04 × 0.02 mm^3 was selected and mounted on an XtaLAB Synergy R, DW system, HyPix-Arc 150 diffractometer. The crystal was kept at a steady T = 140.00(10) K during data collection. The structure was solved with the ShelXT 2018/2 (Sheldrick, 2015) solution program using dual methods and by using Olex2 1.5 (Dolomanov et al., 2009) as the graphical interface. The model was refined with ShelXL 2019/3 (Sheldrick, 2015) using full matrix least squares minimisation on $\boldsymbol{F^2}$.

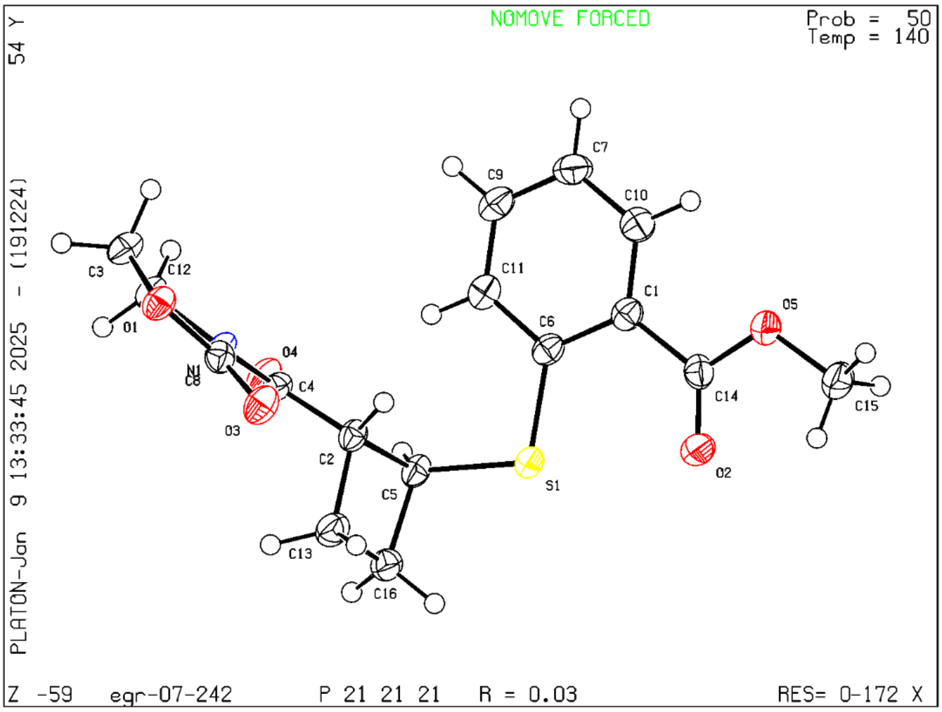

*Model has Chirality at **C2** (Sohncke SpGr)* ***R*** *Verify*
*Model has Chirality at **C5** (Sohncke SpGr)* ***S*** *Verify*

Table 9: Fractional Atomic Coordinates (×10^4) and Equivalent Isotropic Displacement Parameters (Å^2×10^3) for **4b**. U_{eq} is defined as 1/3 of the trace of the orthogonalised U_{ij}.

Atom	x	y	z	U_{eq}
S1	1509.4(15)	8641.2(8)	6260.3(2)	25.50(18)

Atom	x	y	z	U_{eq}
O1	-1391(5)	4158(2)	6045.1(6)	29.7(5)
O2	4880(4)	4750(2)	5216.4(7)	30.8(5)
O3	5220(4)	2351(2)	5293.2(6)	26.6(5)
O4	4509(5)	10824(2)	6582.0(7)	37.5(6)
O5	8013(5)	10560(2)	6999.7(7)	30.3(5)
N1	2058(5)	3469(3)	5653.9(7)	21.8(5)
C1	-340(6)	7185(3)	6027.9(9)	23.8(6)
C2	1357(6)	6078(3)	5784.2(9)	22.7(6)
C3	406(7)	6788(3)	5379.0(9)	29.9(7)
C4	-1524(6)	7762(3)	5617.8(9)	27.6(6)
C5	511(6)	4529(3)	5839.0(9)	22.0(6)
C6	4126(6)	3656(3)	5376.7(8)	23.6(6)
C7	4055(6)	1256(3)	5558.8(9)	28.2(6)
C8	1512(6)	1936(3)	5722.3(10)	25.0(6)
C9	3366(6)	7757(3)	6647.0(8)	24.1(6)
C10	5401(6)	8494(3)	6864.3(8)	25.0(6)
C11	6946(7)	7736(4)	7146.6(9)	29.2(7)
C12	6512(7)	6283(4)	7223.8(9)	33.3(7)
C13	4485(7)	5573(3)	7019.9(10)	31.4(7)
C14	2938(6)	6290(4)	6737.1(9)	28.4(7)
C15	5877(6)	10064(3)	6795.3(9)	27.2(7)
C16	8589(8)	12087(3)	6944.5(11)	34.9(7)

Table 10: Anisotropic Displacement Parameters (×10[4]) for **4b**. The anisotropic displacement factor exponent takes the form: $-2\pi^2[h^2a^{*2} \times U_{11} + \ldots + 2hka^* \times b^* \times U_{12}]$

Atom	U_{11}	U_{22}	U_{33}	U_{23}	U_{13}	U_{12}
S1	28.2(4)	18.3(3)	29.9(4)	-0.6(3)	-2.9(3)	-0.5(3)
O1	29.0(11)	20.8(10)	39.2(11)	1.2(9)	10.1(11)	-1.1(10)
O2	28.9(12)	24.5(12)	39.1(12)	0.8(9)	7.7(10)	-2.8(9)
O3	23.4(11)	22.6(11)	34.0(11)	-1.7(9)	3.6(9)	4.1(9)
O4	38.4(13)	23.9(11)	50.1(14)	3.1(10)	-16.3(11)	-1.0(10)
O5	31.7(12)	23.8(11)	35.5(11)	-2.2(9)	-6.4(10)	-2.1(10)
N1	18.9(12)	15.2(12)	31.1(12)	0.6(10)	3.0(9)	1.6(10)
C1	21.3(14)	17.5(14)	32.4(15)	-0.9(12)	1.8(12)	-1.2(12)
C2	19.1(13)	18.7(14)	30.3(14)	-1.5(11)	1.5(12)	-1.2(12)
C3	35.8(17)	23.6(15)	30.1(16)	2.8(12)	1.0(14)	-0.9(14)
C4	23.8(15)	23.0(15)	36.0(16)	1.0(12)	-4.6(13)	-1.9(14)
C5	21.0(14)	18.6(14)	26.4(14)	1.1(11)	-0.6(12)	2.2(12)
C6	19.7(14)	23.8(14)	27.4(14)	-2.3(13)	0.0(11)	-0.9(13)
C7	25.3(15)	21.4(14)	38.0(16)	3.6(13)	3.2(12)	3.4(13)
C8	22.4(14)	16.1(13)	36.5(15)	1.4(11)	1.4(13)	-0.3(13)
C9	25.5(15)	21.5(14)	25.4(14)	-0.1(11)	4.6(12)	3.3(13)
C10	26.6(15)	24.0(15)	24.4(14)	-1.6(12)	3.9(12)	1.7(13)
C11	26.8(17)	30.5(16)	30.2(16)	-1.2(13)	1.0(13)	2.0(14)
C12	38.5(18)	31.1(16)	30.5(15)	6.3(14)	-2.6(15)	6.6(18)
C13	38.2(18)	22.5(16)	33.4(17)	4.2(13)	4.2(14)	1.7(14)
C14	30.6(17)	23.7(14)	30.8(15)	-1.0(13)	2.3(12)	0.4(14)
C15	28.6(17)	26.1(16)	26.7(15)	-5.3(12)	-1.2(12)	-0.7(13)
C16	36.2(18)	23.2(15)	45.4(18)	-1.5(14)	-6.0(17)	-3.7(16)

Table 11: Bond Lengths in Å for **4b**.

Atom	Atom	Length/Å
S1	C1	1.801(3)
S1	C9	1.769(3)
O1	C5	1.217(4)
O2	C6	1.199(4)

Atom	Atom	Length/Å
O3	C6	1.351(4)
O3	C7	1.455(4)
O4	C15	1.204(4)
O5	C15	1.343(4)
O5	C16	1.450(4)
N1	C5	1.387(4)
N1	C6	1.388(4)
N1	C8	1.459(4)
C1	C2	1.550(4)
C1	C4	1.559(4)
C2	C3	1.552(4)

Atom	Atom	Length/Å
C2	C5	1.503(4)
C3	C4	1.534(4)
C7	C8	1.518(4)
C9	C10	1.417(4)
C9	C14	1.403(4)
C10	C11	1.393(4)
C10	C15	1.486(4)
C11	C12	1.382(5)
C12	C13	1.381(5)
C13	C14	1.375(4)

Table 12: Bond Angles in ° for **4b**.

Atom	Atom	Atom	Angle/°
C9	S1	C1	103.13(14)
C6	O3	C7	109.7(2)
C15	O5	C16	115.4(3)
C5	N1	C6	127.9(2)
C5	N1	C8	120.8(2)
C6	N1	C8	111.1(2)
C2	C1	S1	115.2(2)
C2	C1	C4	89.6(2)
C4	C1	S1	107.6(2)
C1	C2	C3	89.5(2)
C5	C2	C1	114.3(2)
C5	C2	C3	114.6(2)
C4	C3	C2	90.5(2)
C3	C4	C1	89.7(2)
O1	C5	N1	118.8(3)
O1	C5	C2	123.7(3)
N1	C5	C2	117.5(2)
O2	C6	O3	122.3(3)

Atom	Atom	Atom	Angle/°
O2	C6	N1	128.7(3)
O3	C6	N1	108.9(2)
O3	C7	C8	105.0(2)
N1	C8	C7	100.9(2)
C10	C9	S1	121.0(2)
C14	C9	S1	121.0(2)
C14	C9	C10	118.0(3)
C9	C10	C15	120.5(3)
C11	C10	C9	119.4(3)
C11	C10	C15	120.1(3)
C12	C11	C10	121.4(3)
C13	C12	C11	119.2(3)
C14	C13	C12	120.7(3)
C13	C14	C9	121.3(3)
O4	C15	O5	122.9(3)
O4	C15	C10	124.4(3)
O5	C15	C10	112.7(3)

Table 13: Torsion Angles in ° for **4b**.

Atom	Atom	Atom	Atom	Angle/°
S1	C1	C2	C3	-103.2(2)
S1	C1	C2	C5	139.9(2)
S1	C1	C4	C3	110.1(2)
S1	C9	C10	C11	-175.6(2)
S1	C9	C10	C15	4.9(4)
S1	C9	C14	C13	176.0(3)
O3	C7	C8	N1	-20.1(3)
C1	S1	C9	C10	170.5(2)
C1	S1	C9	C14	-7.1(3)
C1	C2	C3	C4	-6.3(2)
C1	C2	C5	O1	2.3(4)
C1	C2	C5	N1	-175.4(2)
C2	C1	C4	C3	-6.3(2)
C2	C3	C4	C1	6.2(2)
C3	C2	C5	O1	-99.0(3)
C3	C2	C5	N1	83.3(3)
C4	C1	C2	C3	6.2(2)
C4	C1	C2	C5	-110.8(3)
C5	N1	C6	O2	-6.1(5)
C5	N1	C6	O3	175.9(3)
C5	N1	C8	C7	-165.8(2)

Atom	Atom	Atom	Atom	Angle/°
C5	C2	C3	C4	110.3(3)
C6	O3	C7	C8	17.4(3)
C6	N1	C5	O1	173.0(3)
C6	N1	C5	C2	-9.2(4)
C6	N1	C8	C7	17.3(3)
C7	O3	C6	O2	175.1(3)
C7	O3	C6	N1	-6.7(3)
C8	N1	C5	O1	-3.3(4)
C8	N1	C5	C2	174.5(3)
C8	N1	C6	O2	170.5(3)
C8	N1	C6	O3	-7.5(3)
C9	S1	C1	C2	-68.5(2)
C9	S1	C1	C4	-166.6(2)
C9	C10	C11	C12	-0.9(5)
C9	C10	C15	O4	5.6(5)
C9	C10	C15	O5	-174.8(3)
C10	C9	C14	C13	-1.6(4)
C10	C11	C12	C13	-0.8(5)
C11	C10	C15	O4	-173.9(3)
C11	C10	C15	O5	5.7(4)
C11	C12	C13	C14	1.2(5)
C12	C13	C14	C9	0.0(5)
C14	C9	C10	C11	2.0(4)
C14	C9	C10	C15	-177.4(3)
C15	C10	C11	C12	178.6(3)
C16	O5	C15	O4	-0.4(5)
C16	O5	C15	C10	179.9(3)

Table 14: Hydrogen Fractional Atomic Coordinates (×10[4]) and Equivalent Isotropic Displacement Parameters (Å^2×10^3) for **4b**. U_{eq} is defined as 1/3 of the trace of the orthogonalised U_{ij}.

Atom	x	y	z	U_{eq}
H1	-1687.76	6730.55	6213.47	29
H2	3312.07	6214.06	5830.68	27
H3A	1824.4	7330.28	5234.26	36
H3B	-497.91	6104.52	5191.2	36
H4A	-3424.65	7510.75	5577.11	33
H4B	-1221.64	8810.25	5573.35	33
H7A	5284.51	1012.11	5785.77	34
H7B	3652.95	362.86	5402.78	34
H8A	-72.34	1608.87	5566.81	30
H8B	1254.19	1720.23	6016.68	30
H11	8326.24	8227.91	7289.18	35
H12	7596.85	5778.3	7414.92	40
H13	4154.47	4579.38	7075.52	38
H14	1554.02	5781.07	6600.15	34
H1 xx	7913.86	12405.79	6677.68	52
H16B	10520.28	12241.59	6956.55	52
H16C	7721.22	12645.93	7162.18	52

4. NMR Spectra

^{1}H-NMR (400 MHz, chloroform-*d*) (2d)

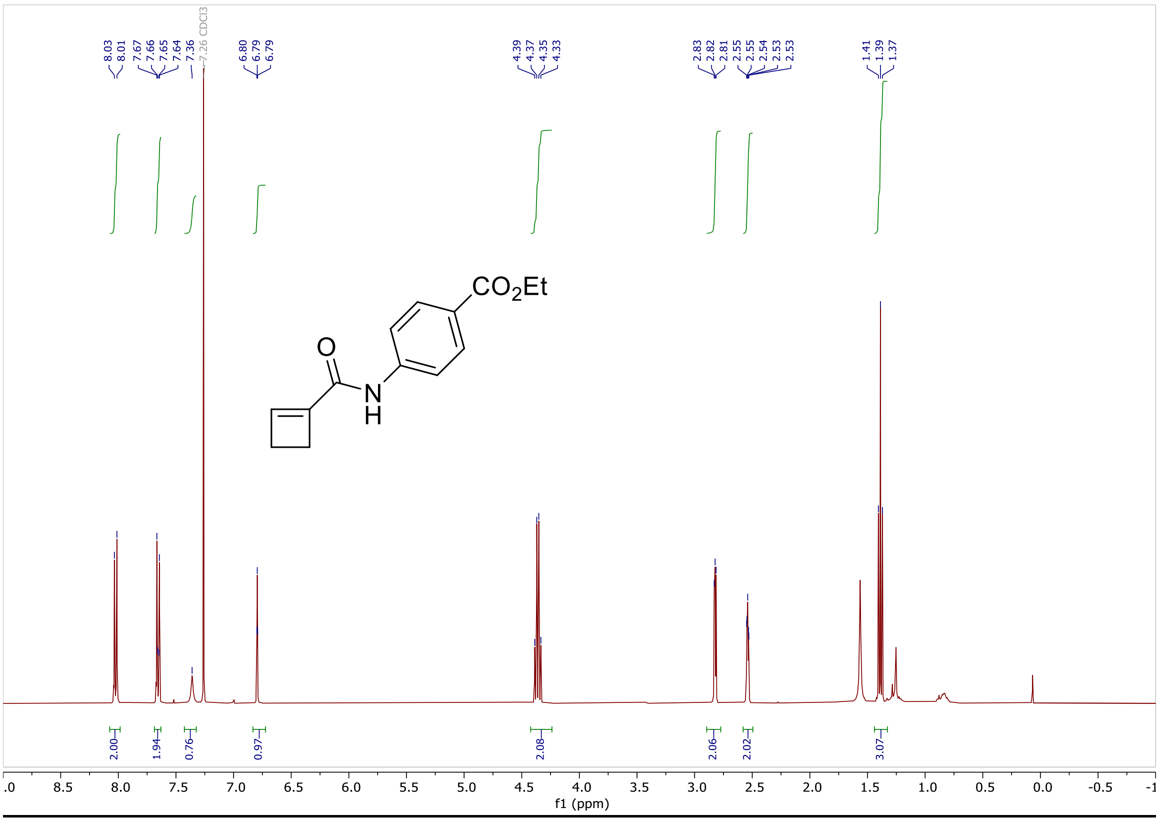

^{13}C-NMR (101 MHz, chloroform-*d*) (2d)

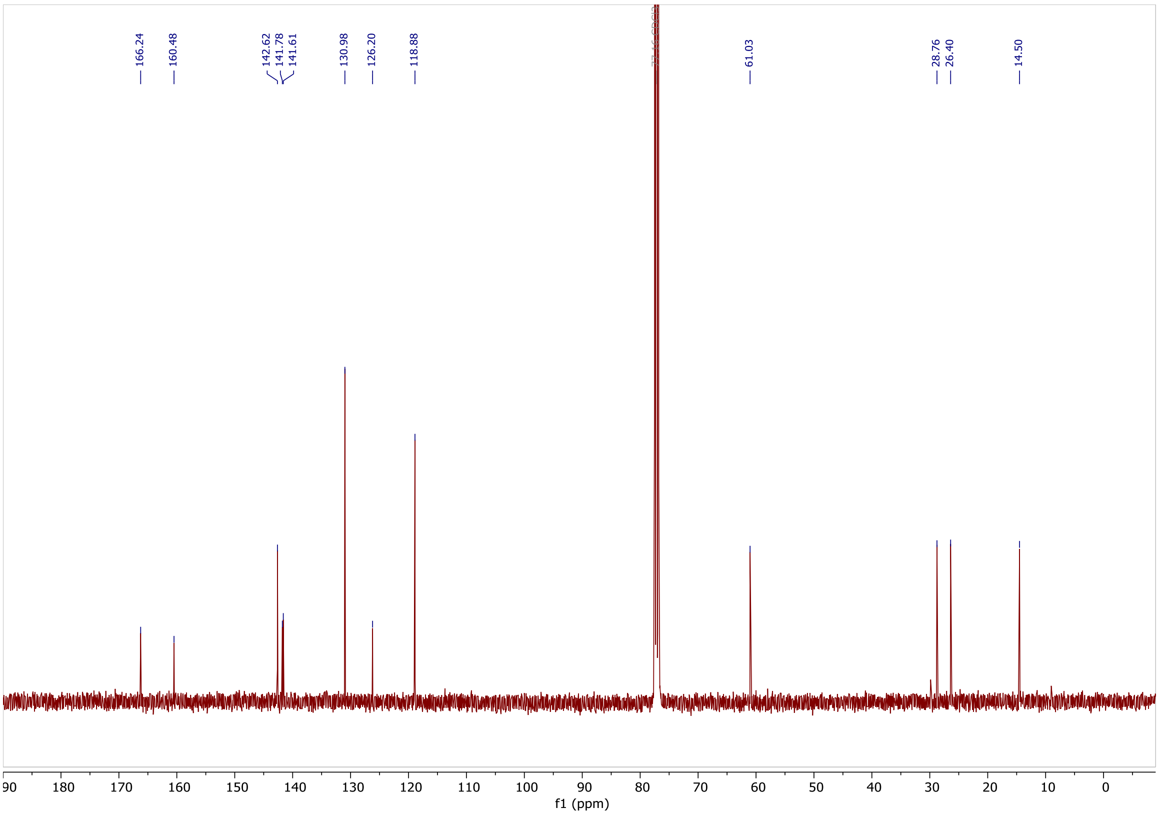

^{1}H-NMR (400 MHz, chloroform-*d*) (3a)

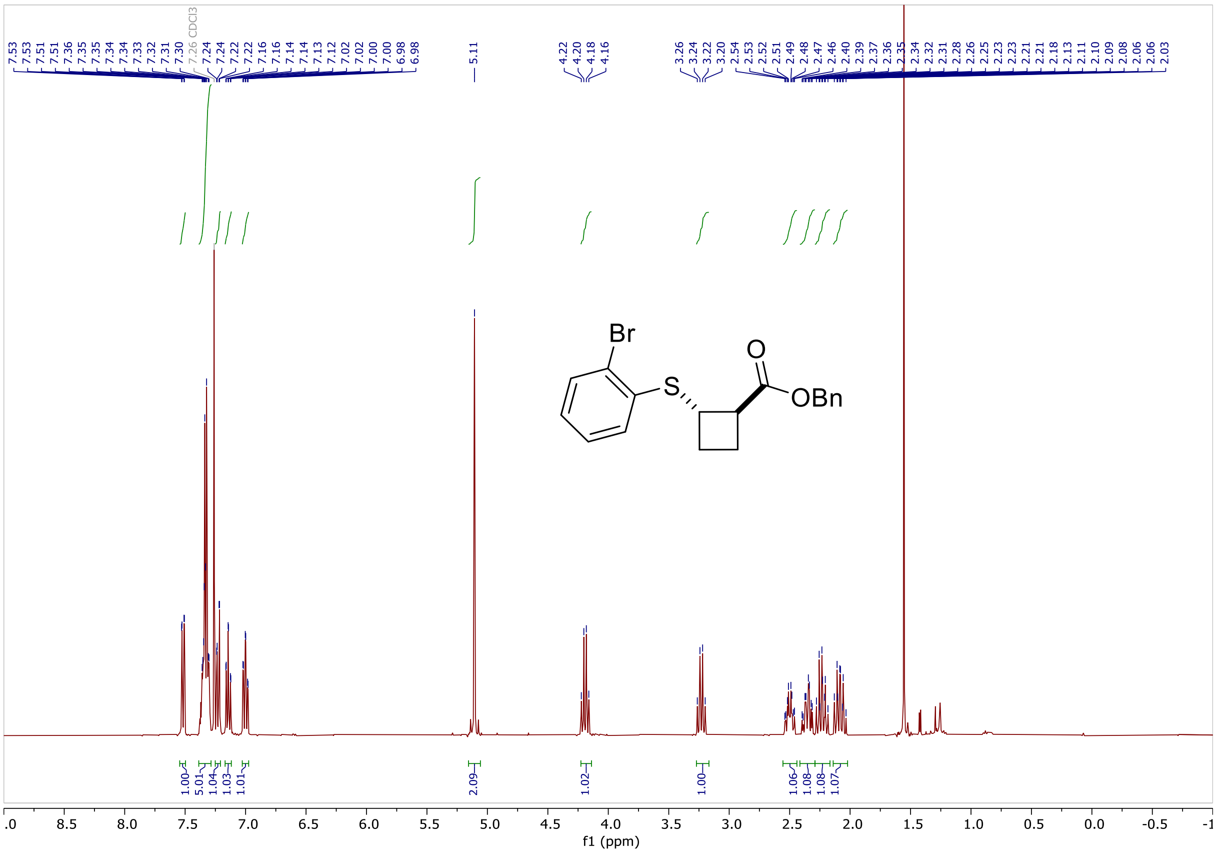

^{13}C-NMR (101 MHz, chloroform-*d*) (3a)

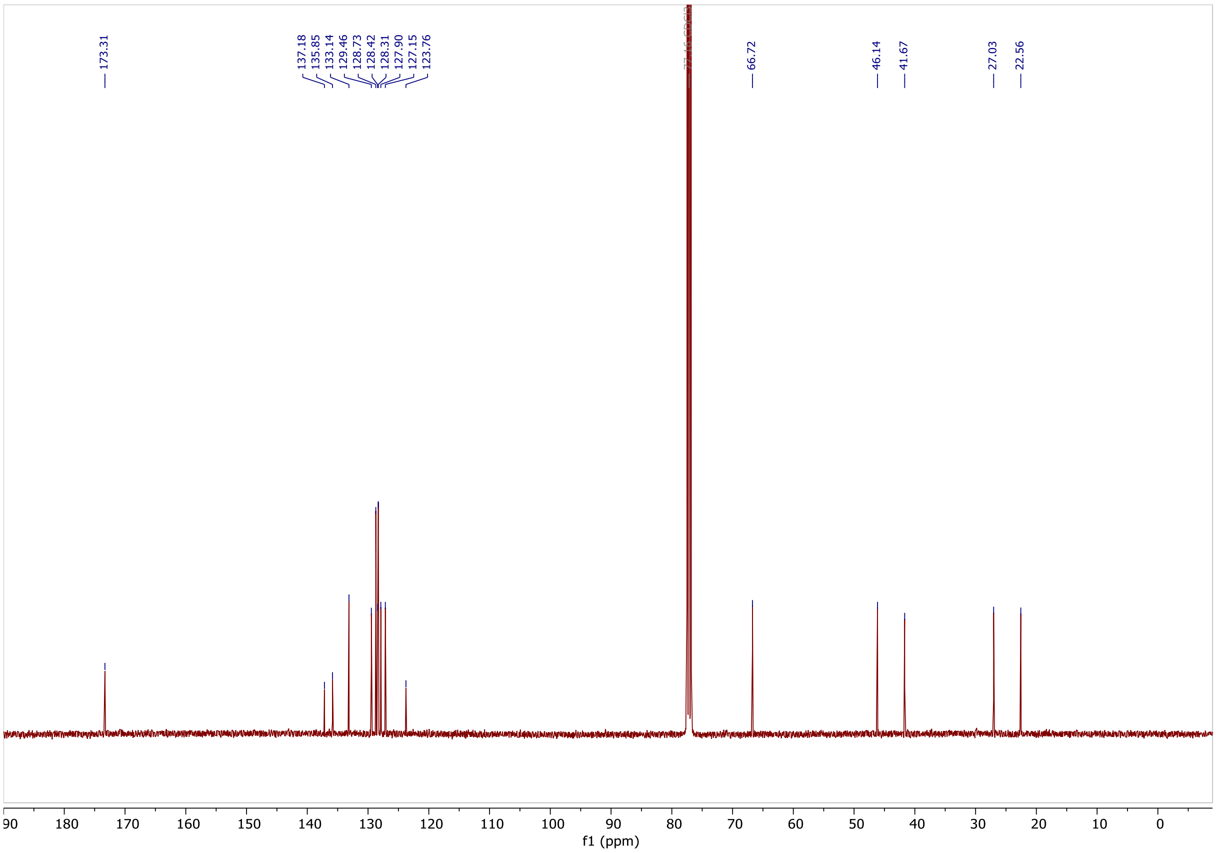

^{1}H-NMR (400 MHz, chloroform-*d*) (3’a)

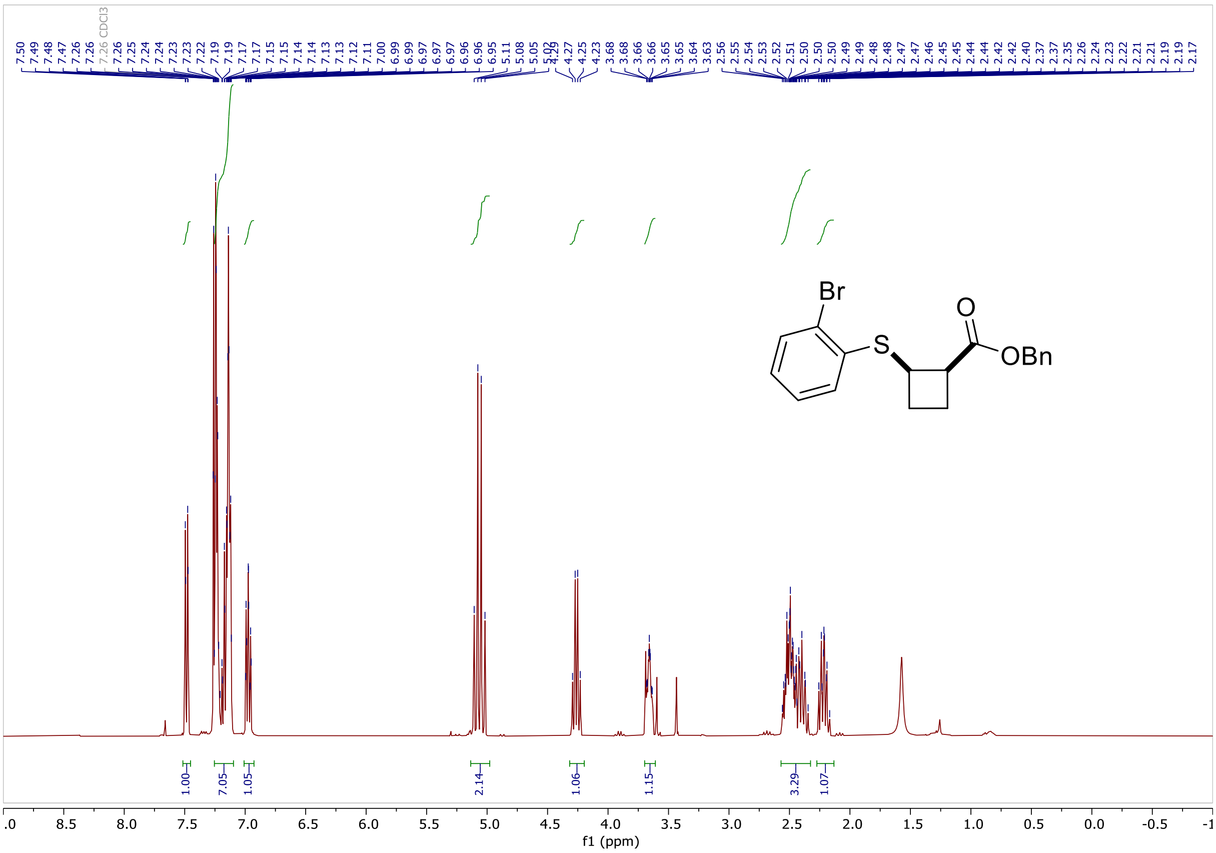

^{13}C-NMR (101 MHz, chloroform-*d*) (3’a)

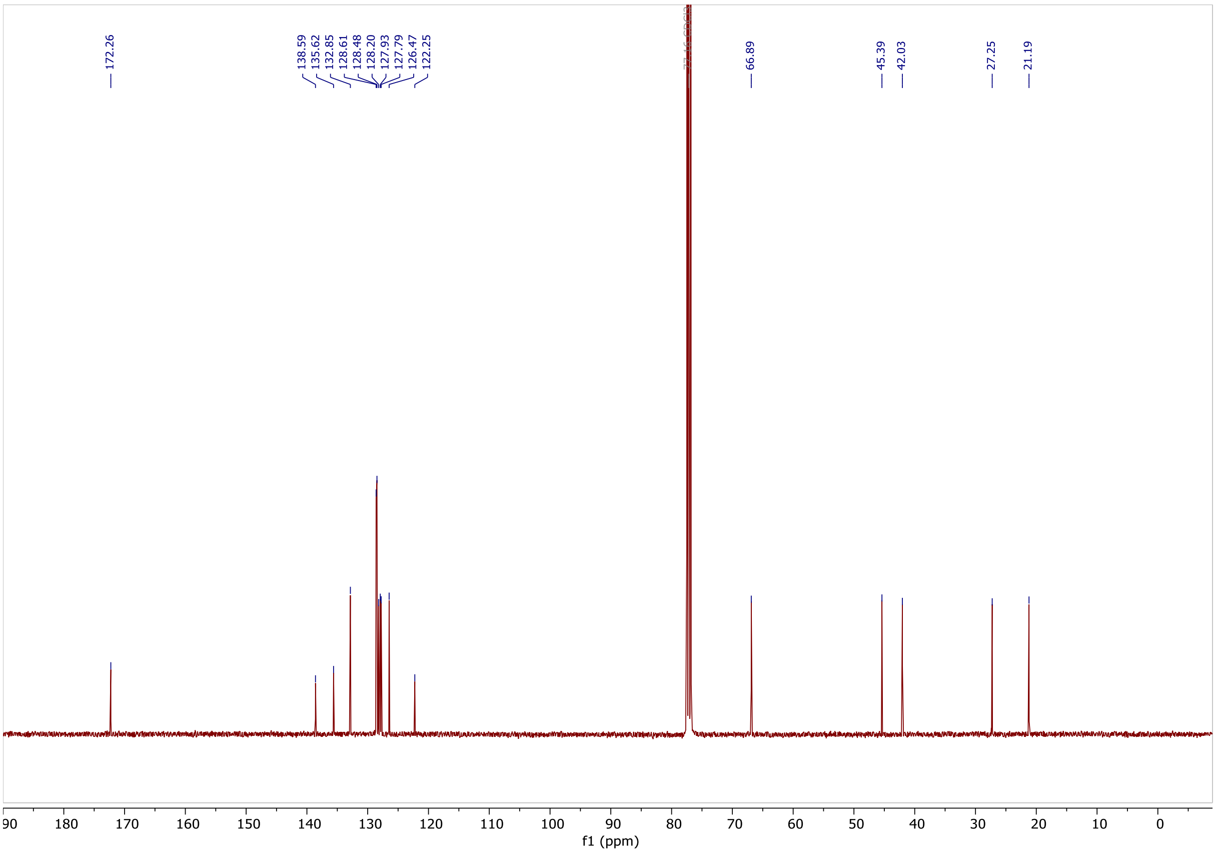

2D-NOESY (400 MHz, chloroform-*d*) (3'a)

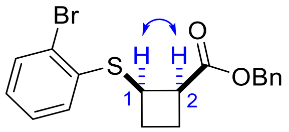

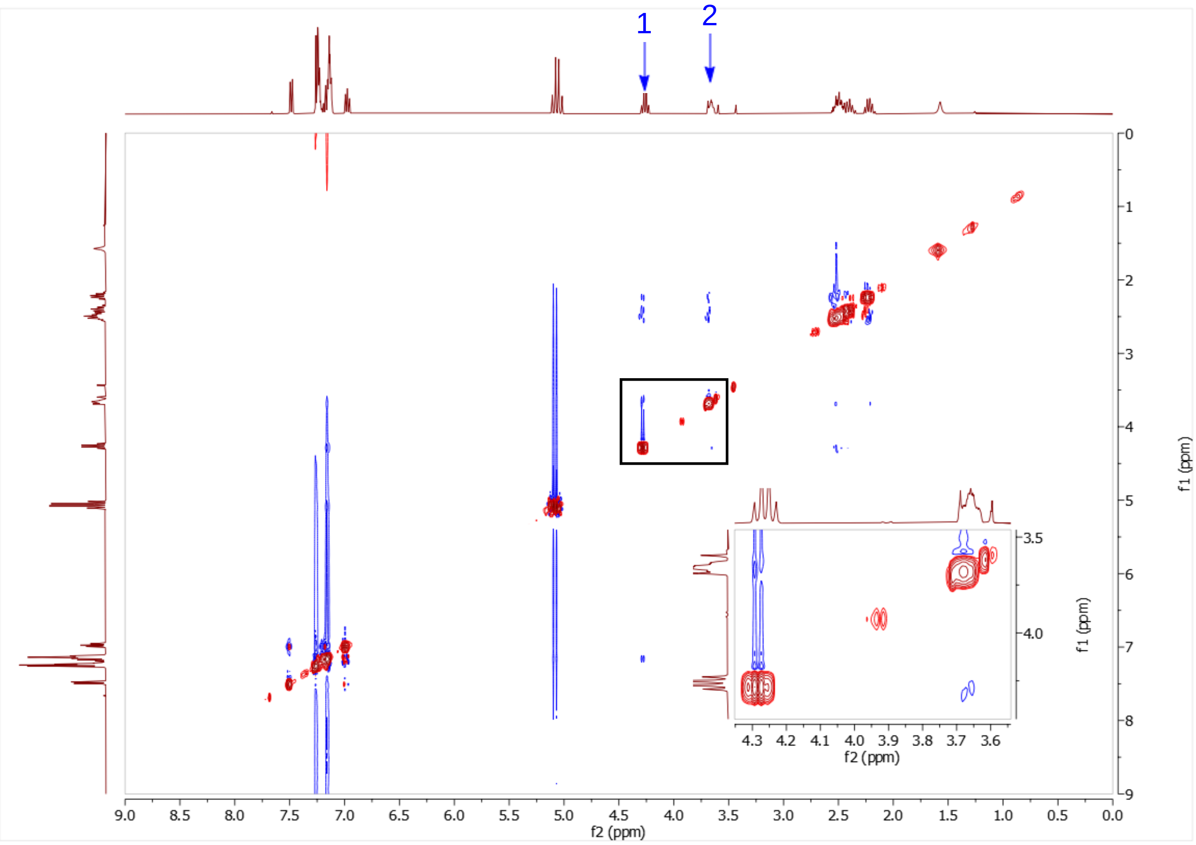

^{1}H-NMR (400 MHz, chloroform-*d*) (3b)

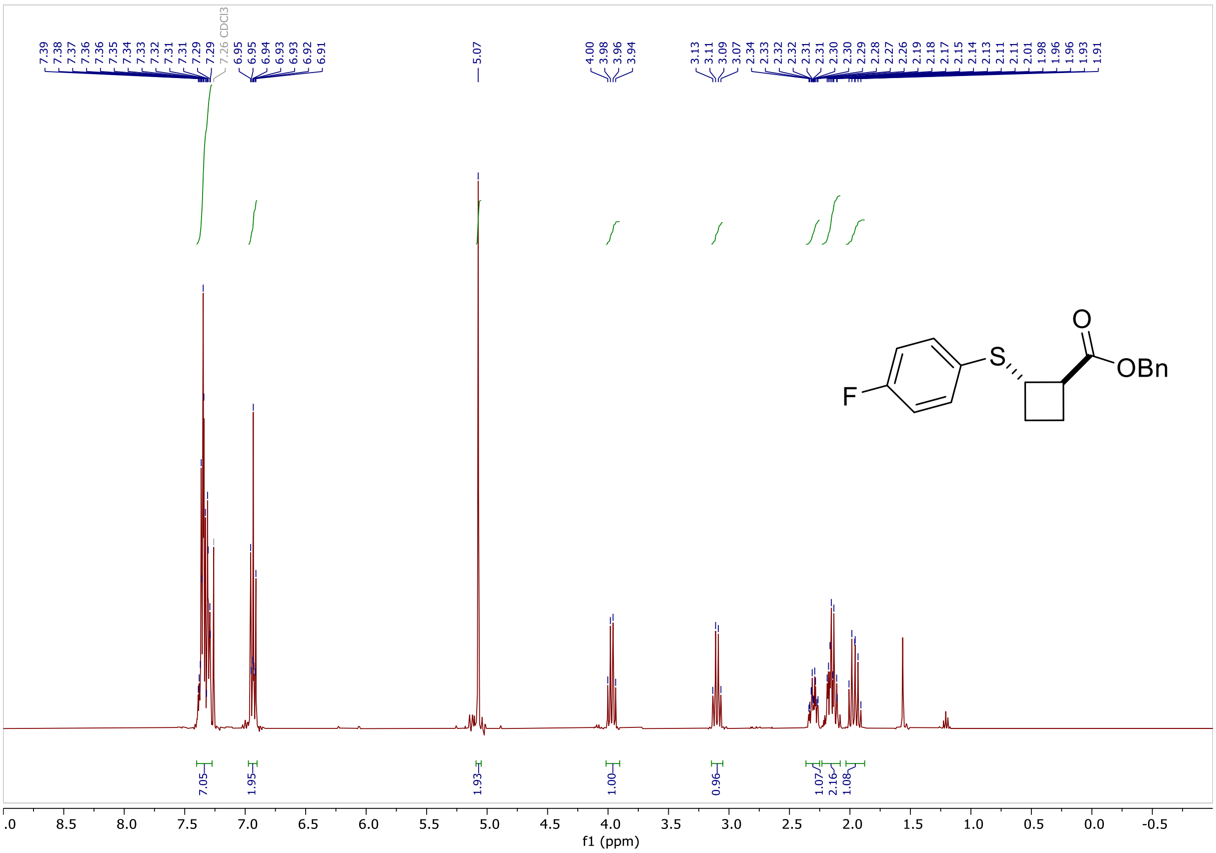

^{13}C-NMR (101 MHz, chloroform-*d*) (3b)

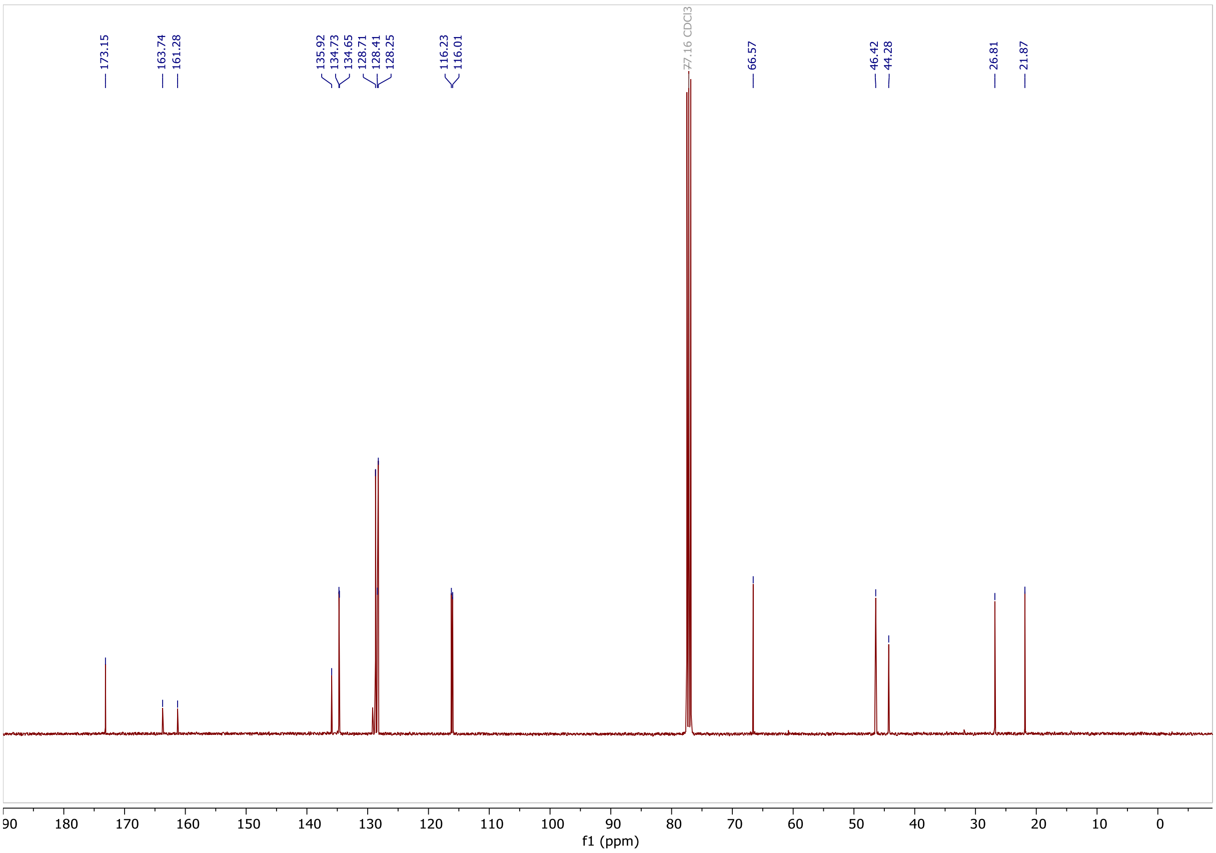

^{19}F-NMR (376 MHz, chloroform-*d*) (3b)

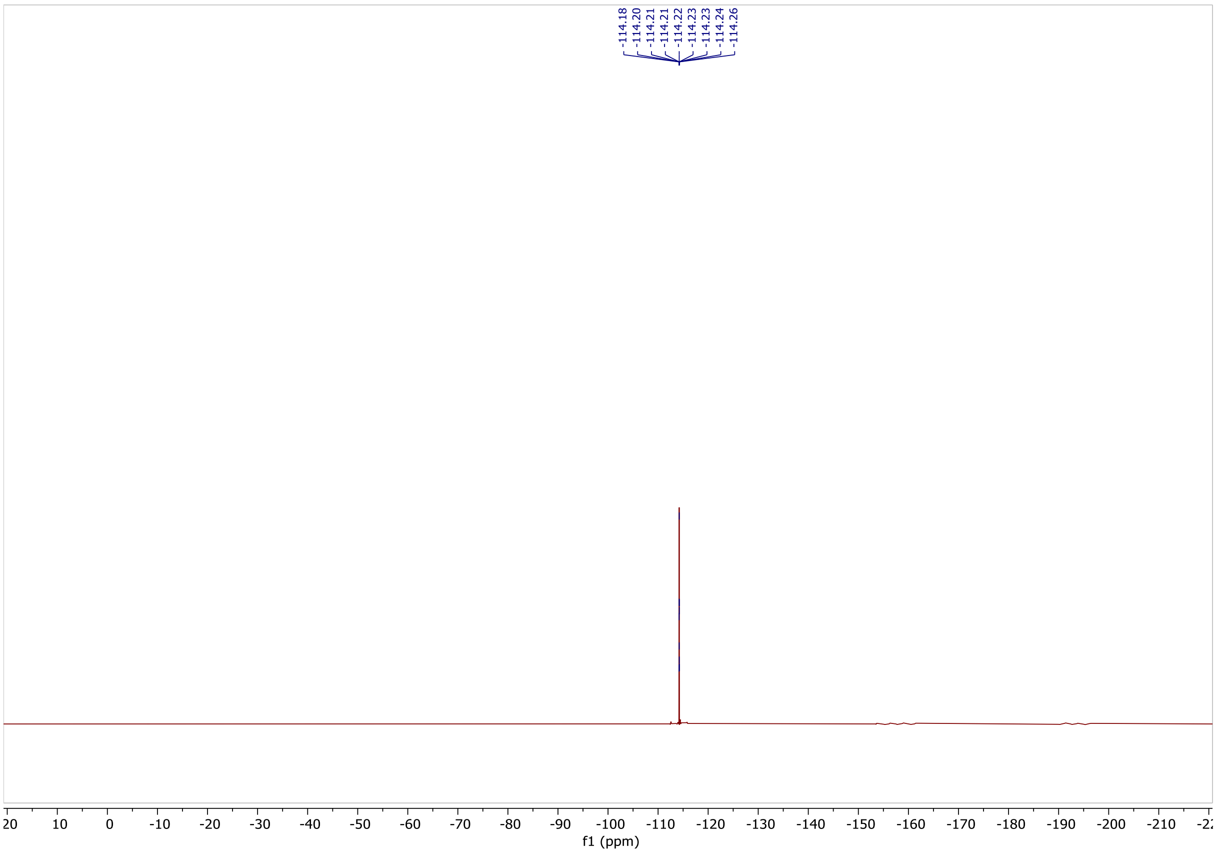

^{1}H-NMR (400 MHz, chloroform-*d*) (3c)

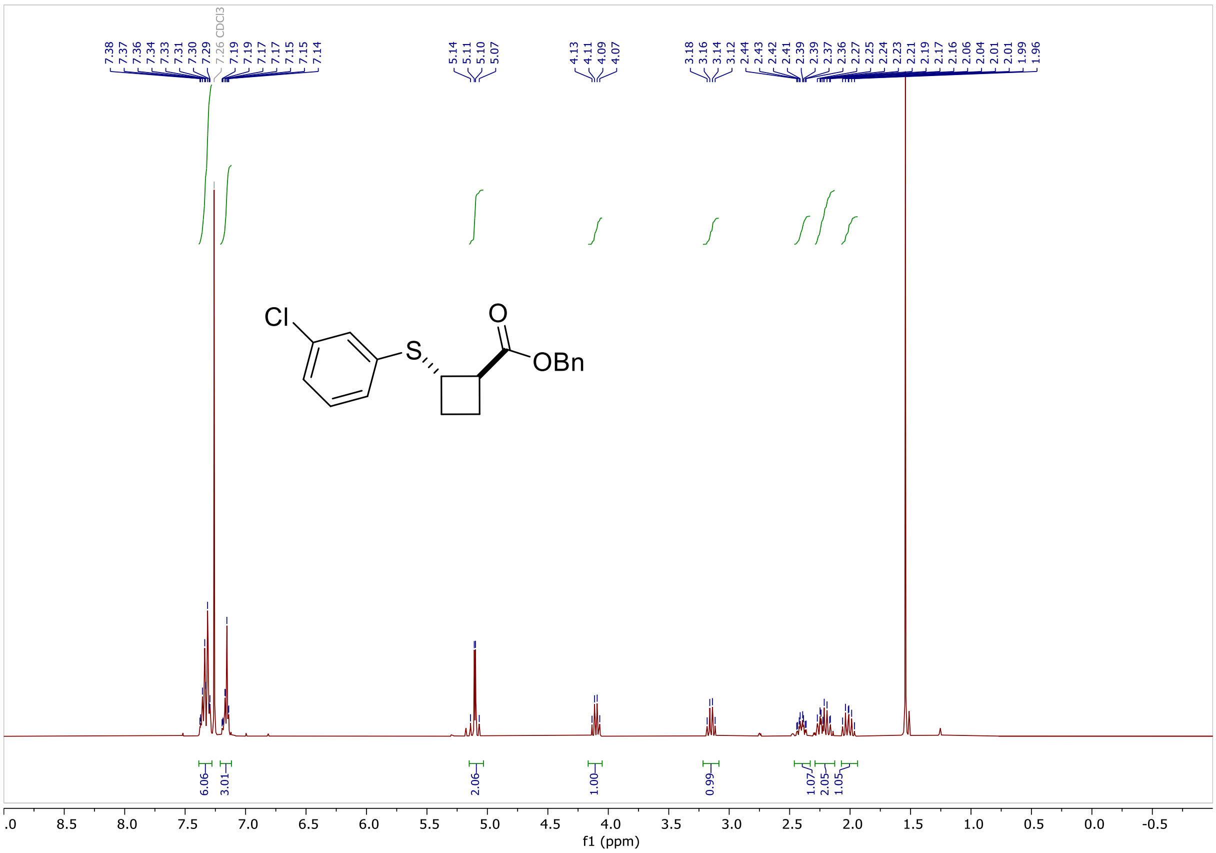

^{13}C-NMR (101 MHz, chloroform-*d*) (3c)

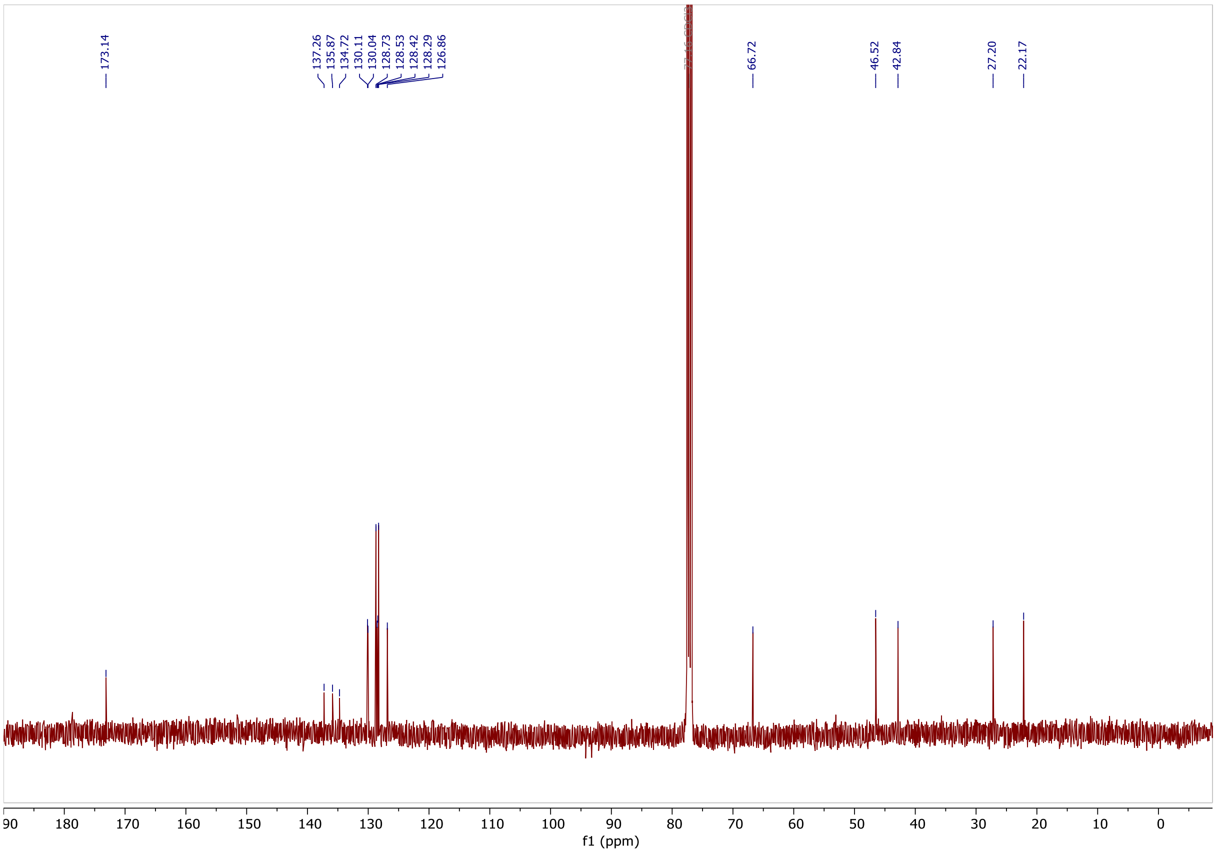

^{1}H-NMR (400 MHz, chloroform-*d*) (3d)

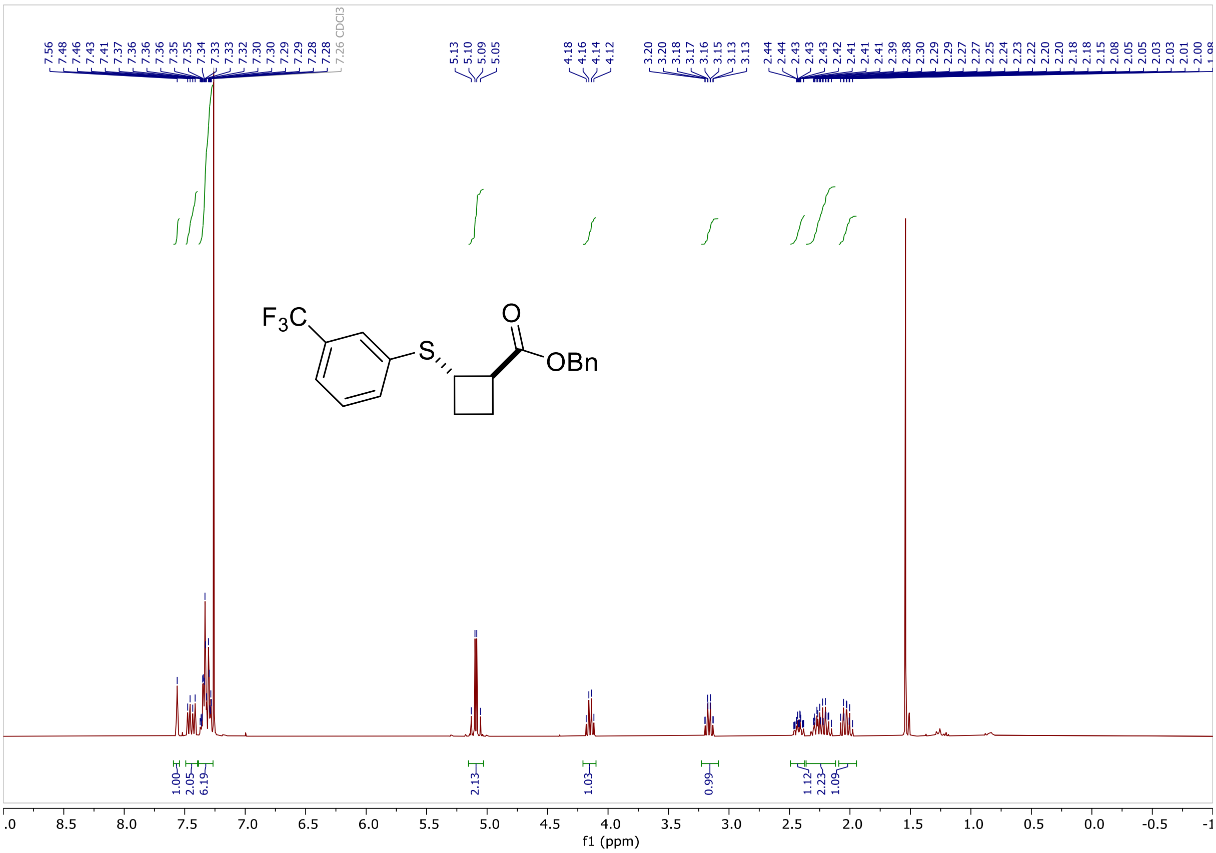

^{13}C-NMR (101 MHz, chloroform-*d*) (3d)

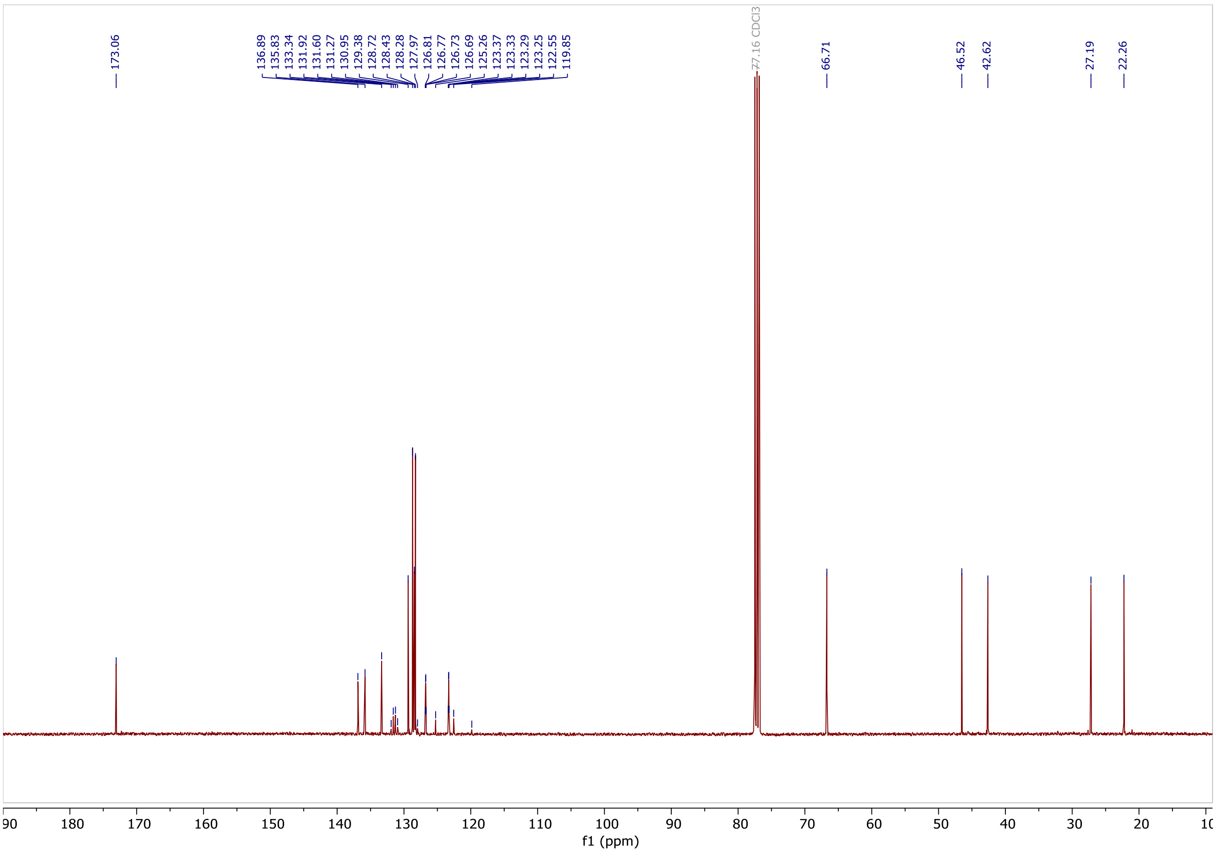

^{19}F-NMR (376 MHz, chloroform-*d*) (3d)

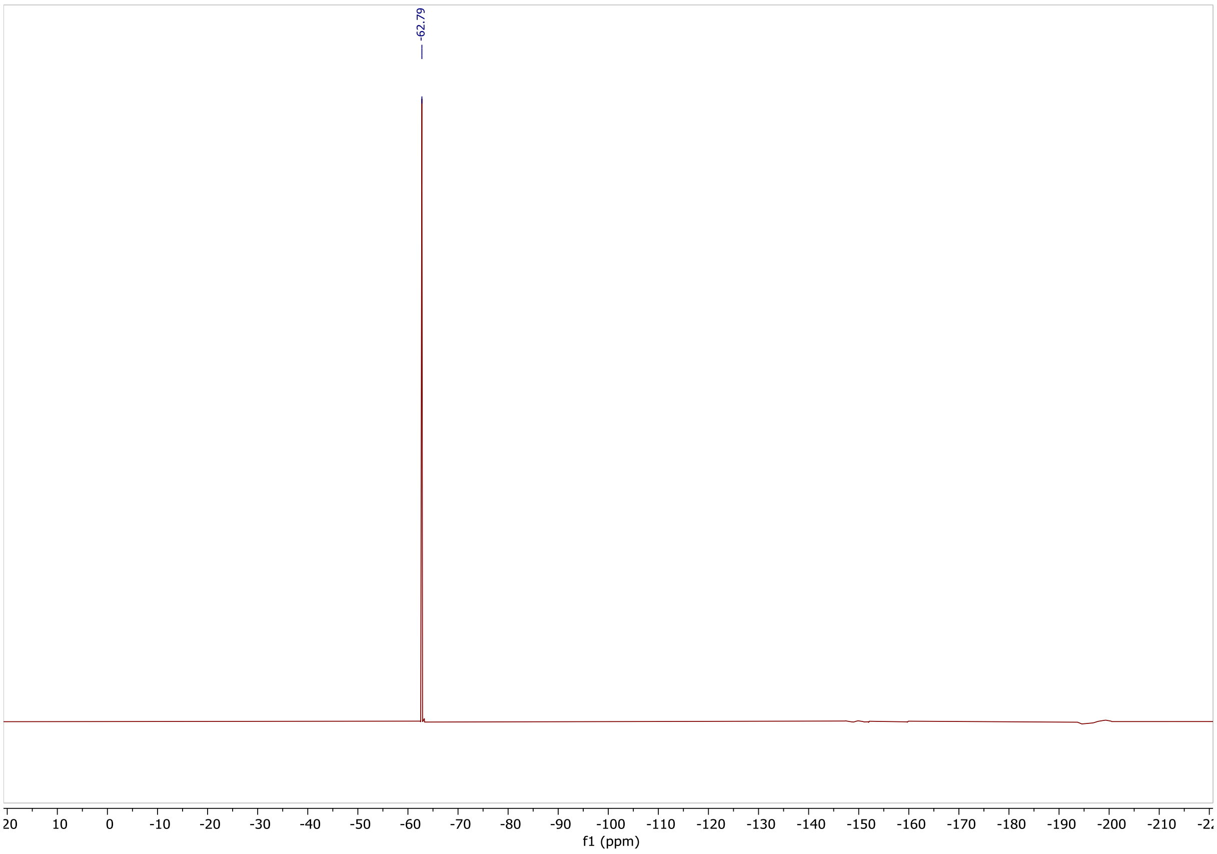

^{1}H-NMR (400 MHz, chloroform-*d*) (3e)

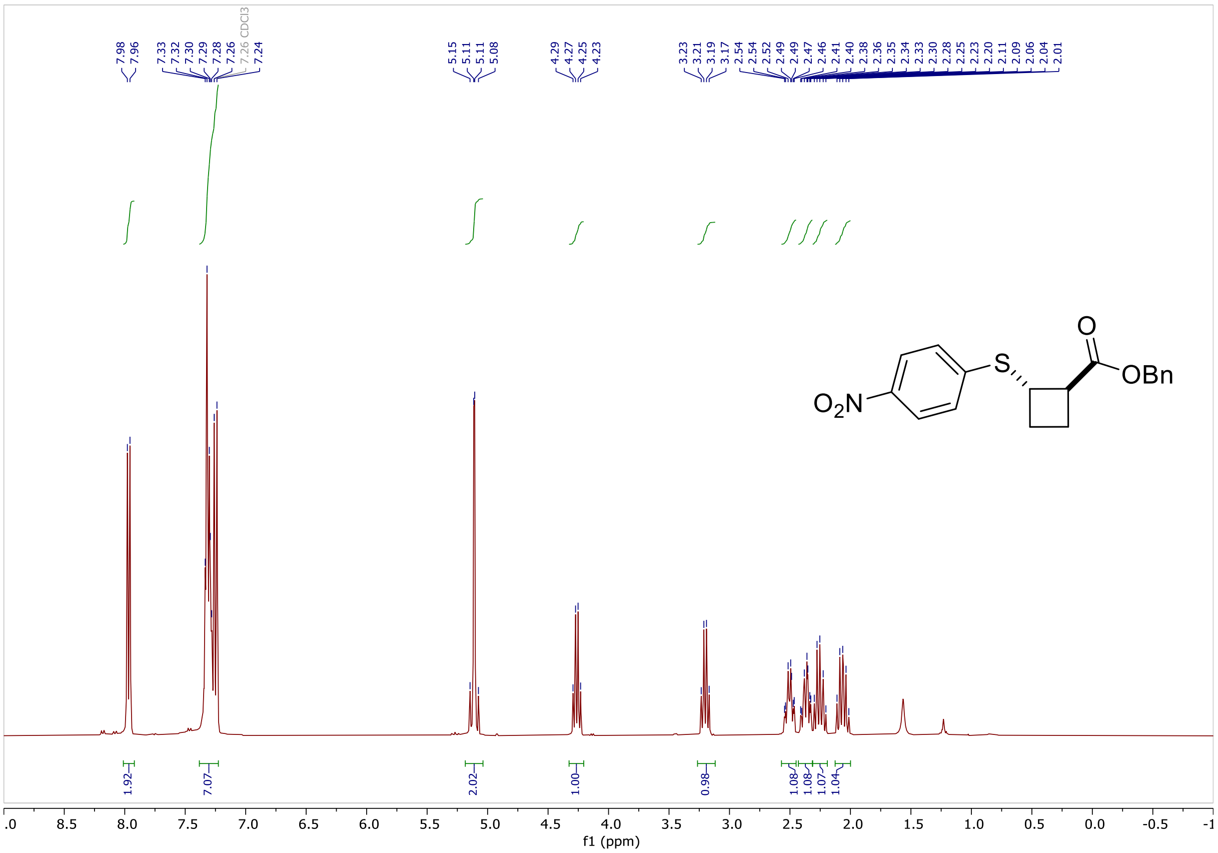

^{13}C-NMR (101 MHz, chloroform-*d*) (3e)

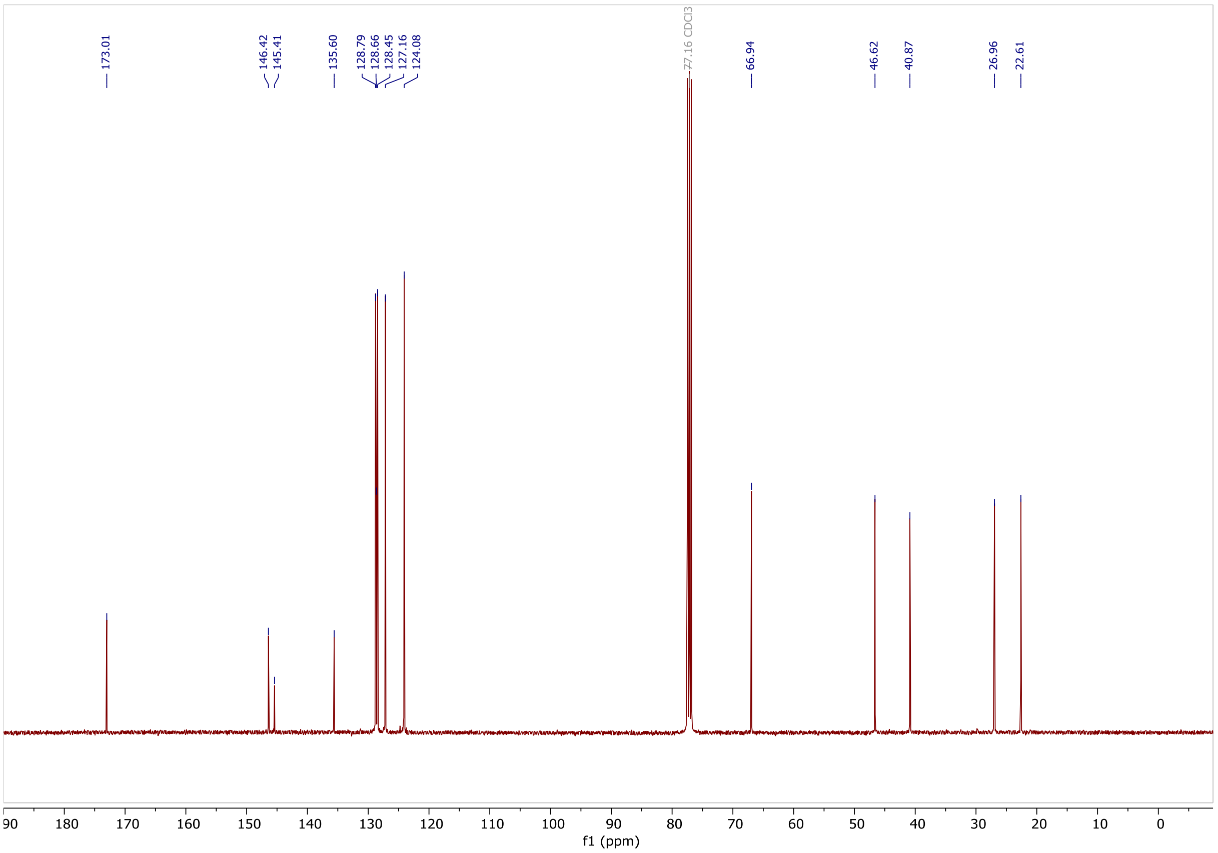

^{1}H-NMR (400 MHz, chloroform-*d*) (3f)

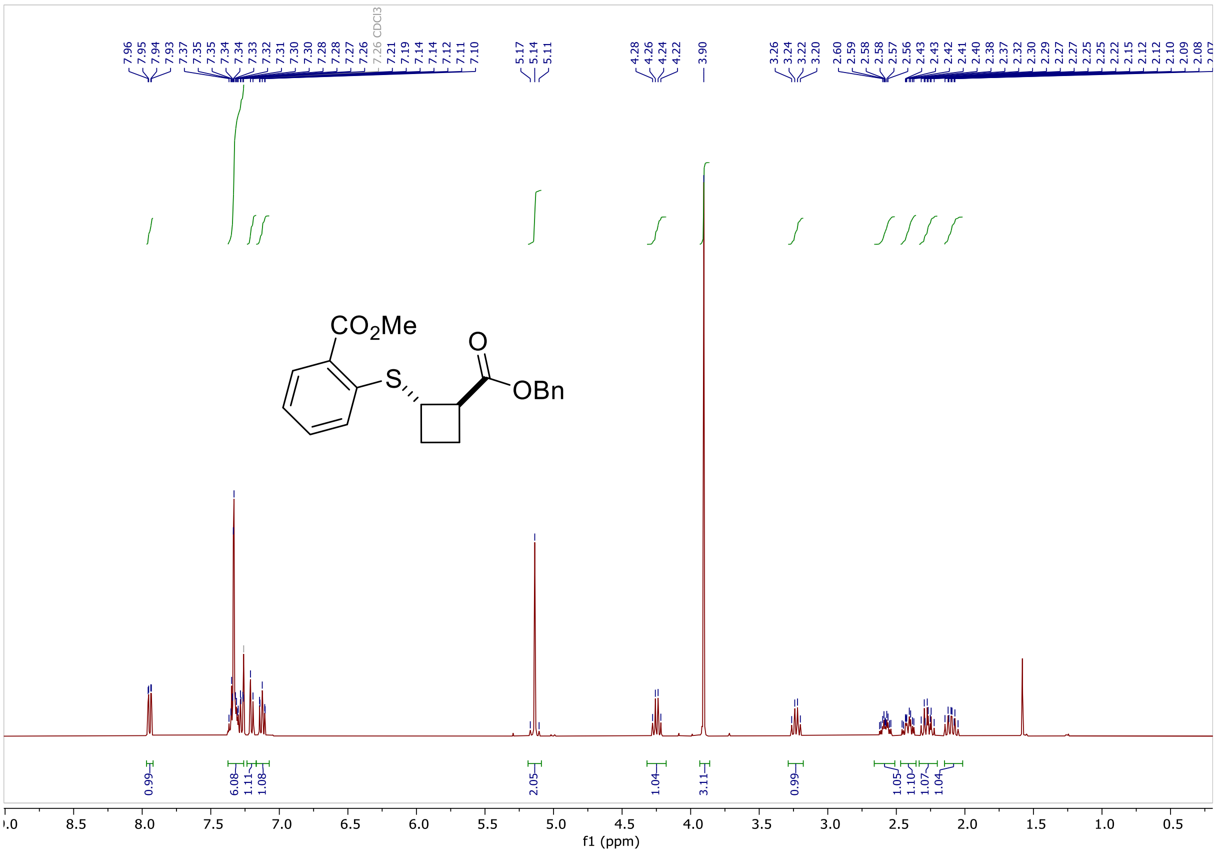

^{13}C-NMR (101 MHz, chloroform-*d*) (3f)

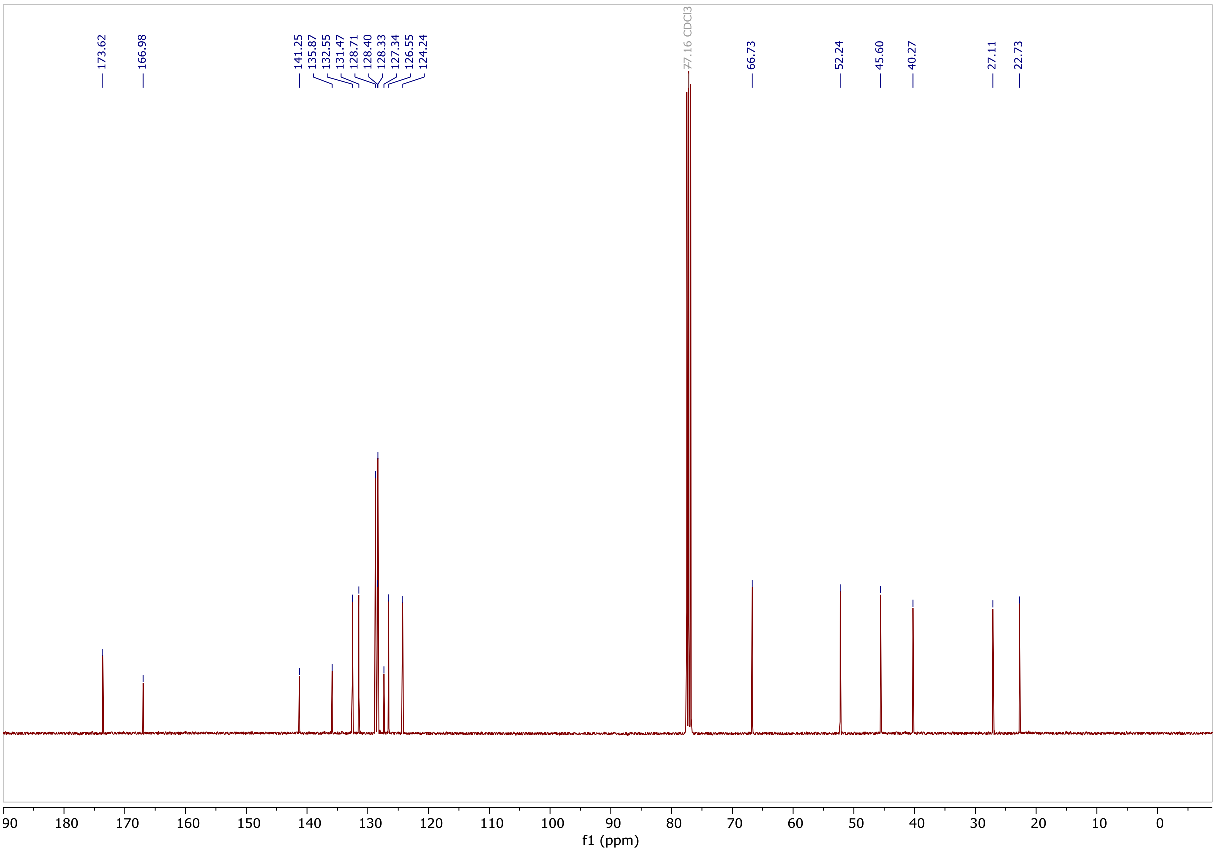

^{1}H-NMR (400 MHz, chloroform-*d*) (3g)

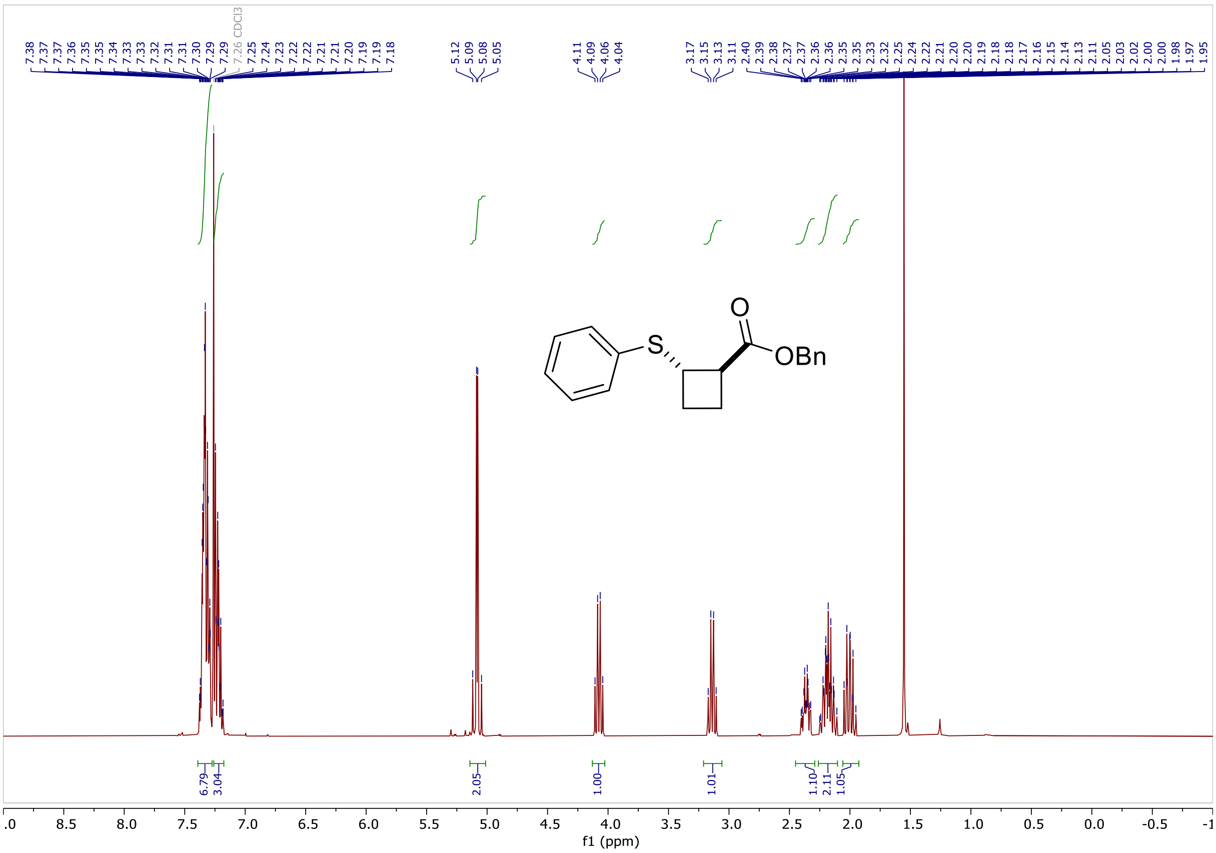

^{13}C-NMR (101 MHz, chloroform-*d*) (3g)

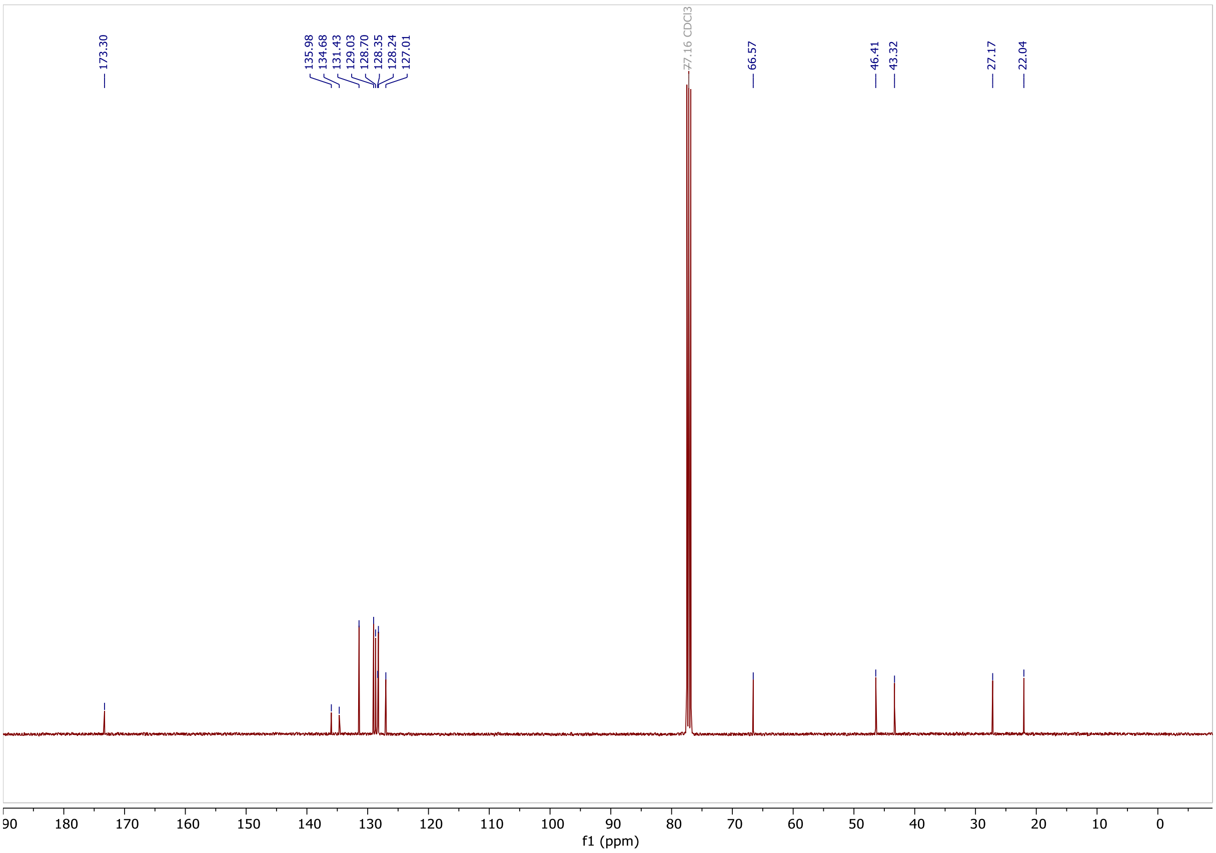

^{1}H-NMR (400 MHz, chloroform-*d*) (3h)

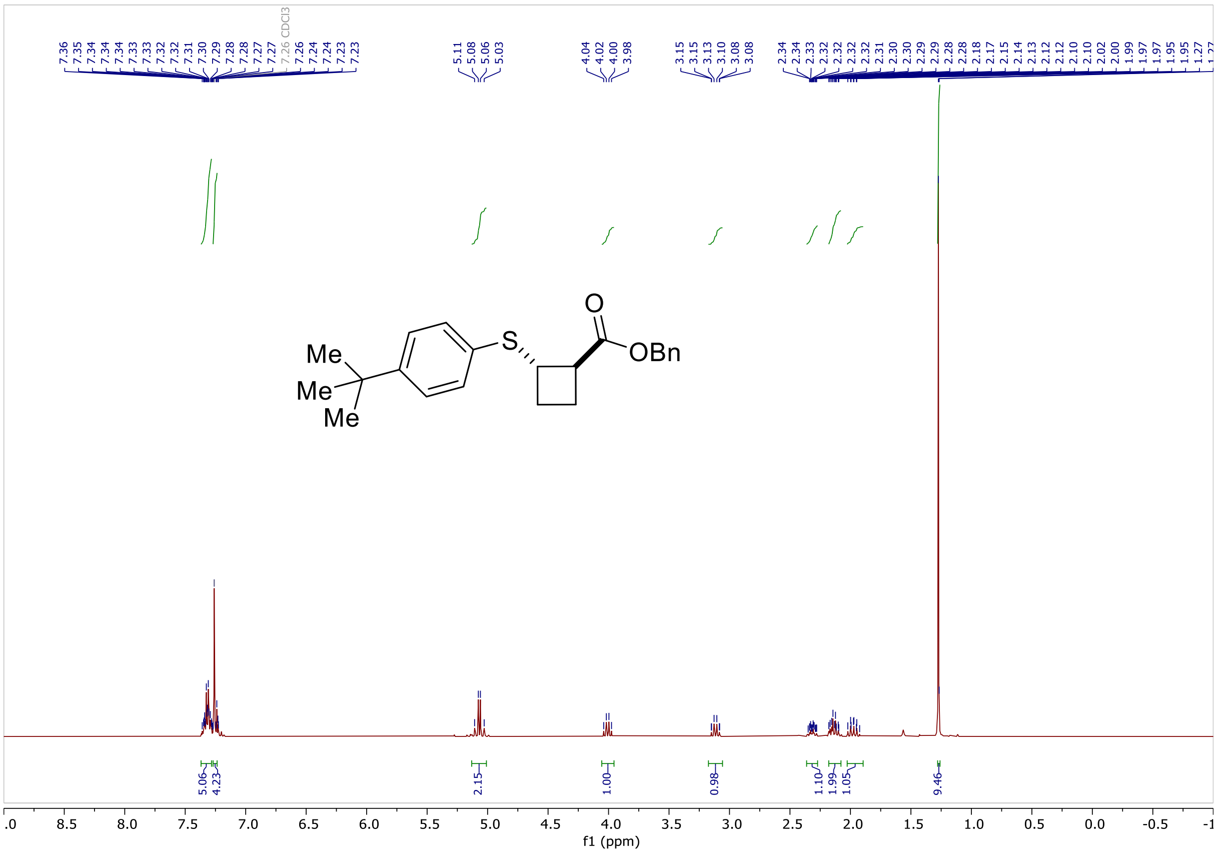

^{13}C-NMR (101 MHz, chloroform-*d*) (3h)

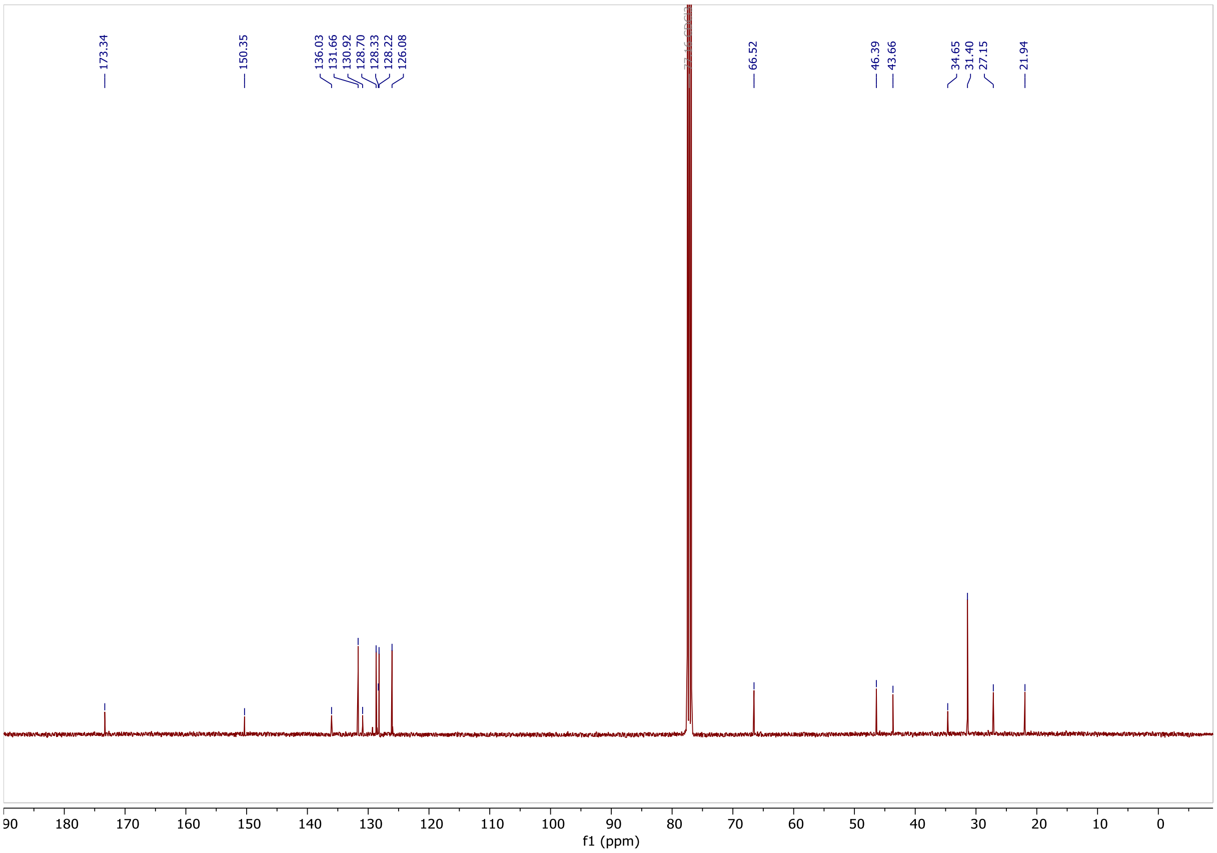

^{1}H-NMR (400 MHz, chloroform-*d*) (3i)

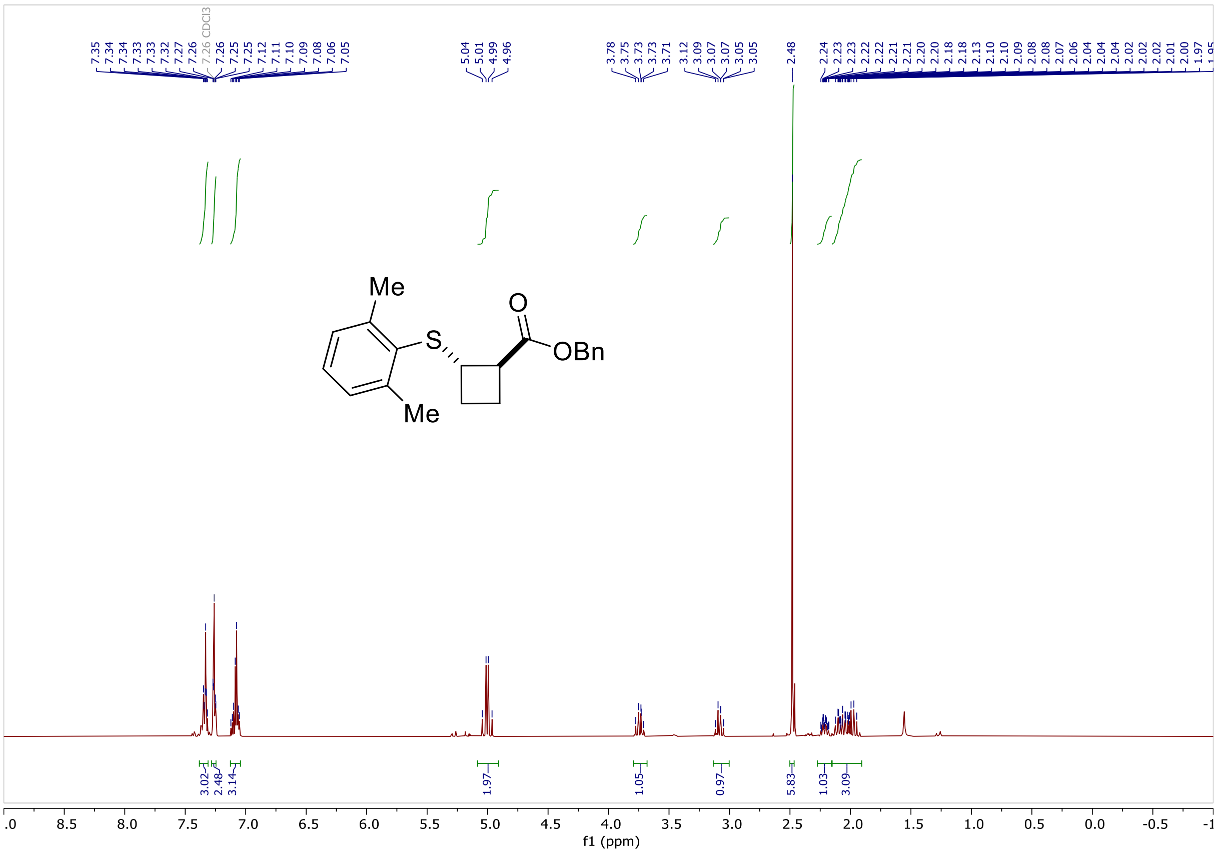

^{13}C-NMR (101 MHz, chloroform-*d*) (3i)

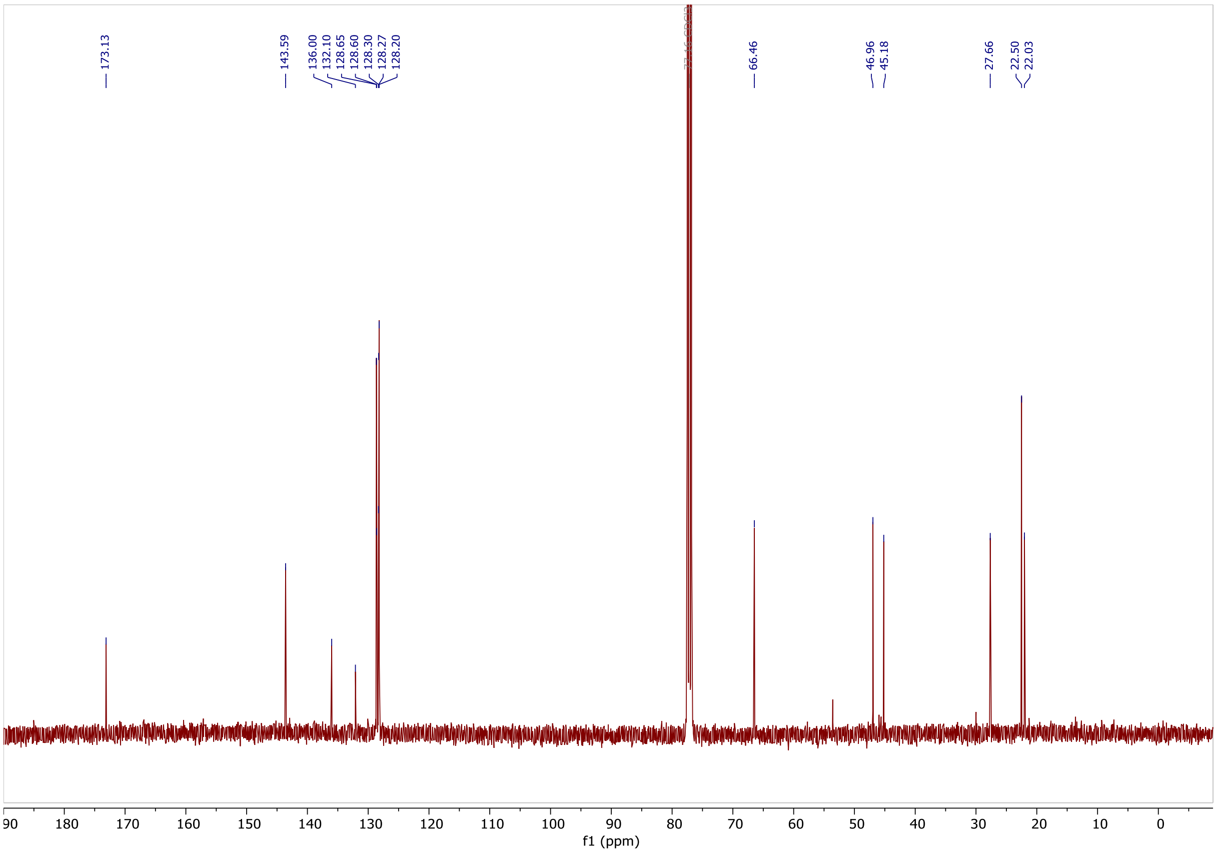

^{1}H-NMR (400 MHz, chloroform-*d*) (3j)

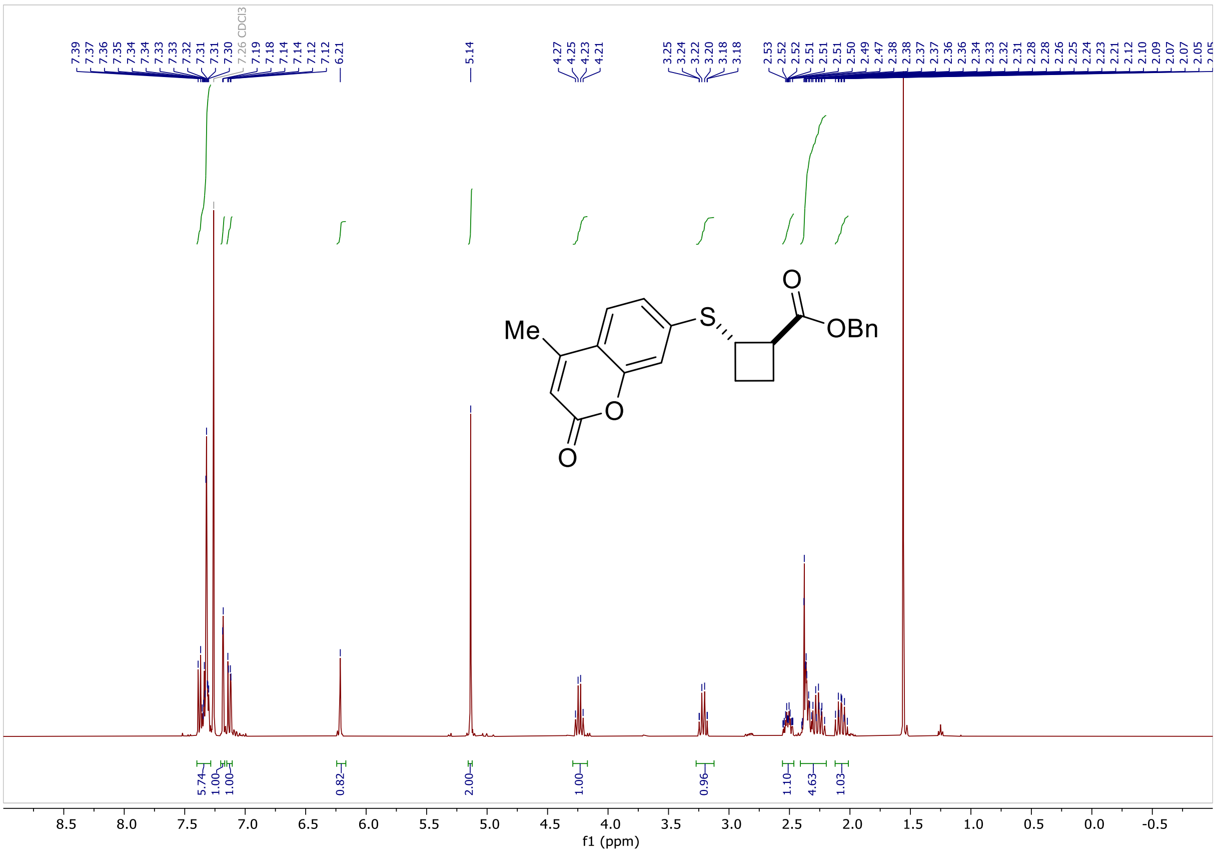

^{13}C-NMR (101 MHz, chloroform-*d*) (3j)

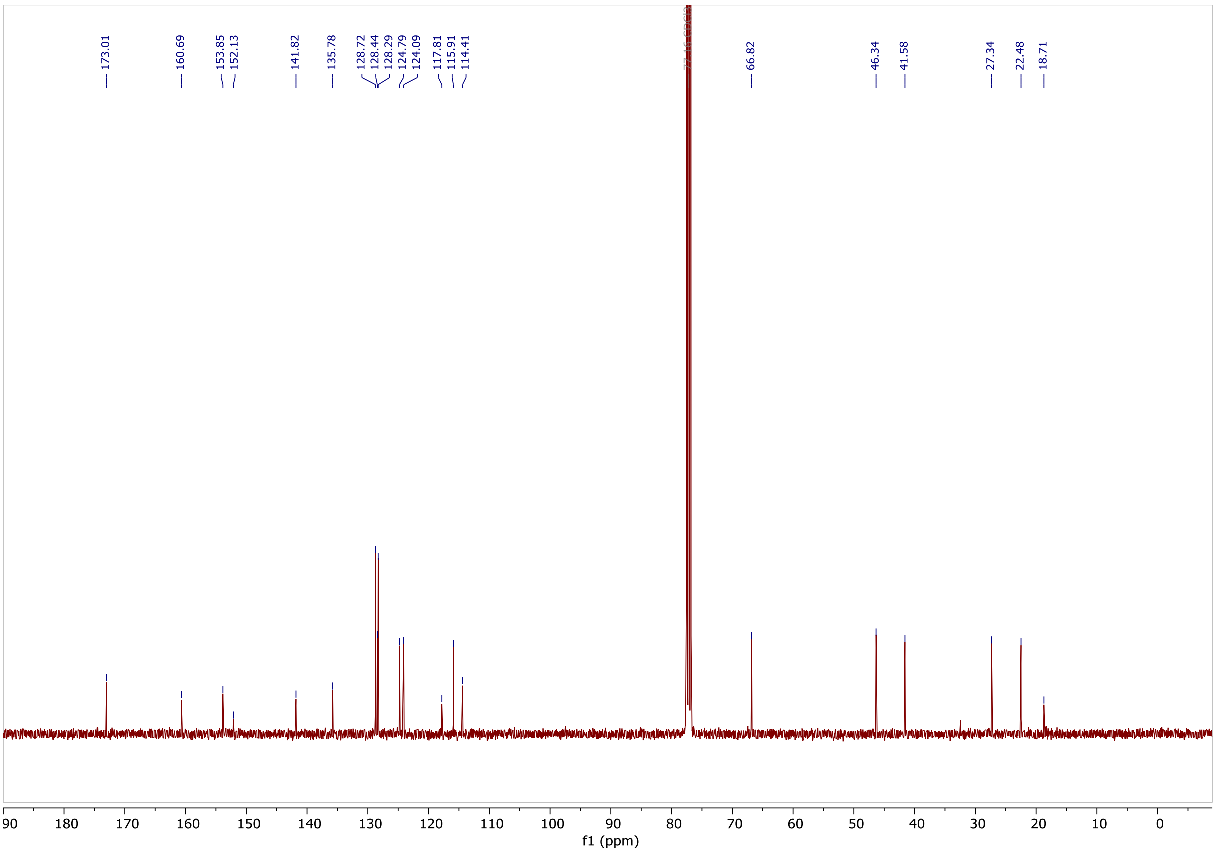

^{1}H-NMR (400 MHz, chloroform-*d*) (3k)

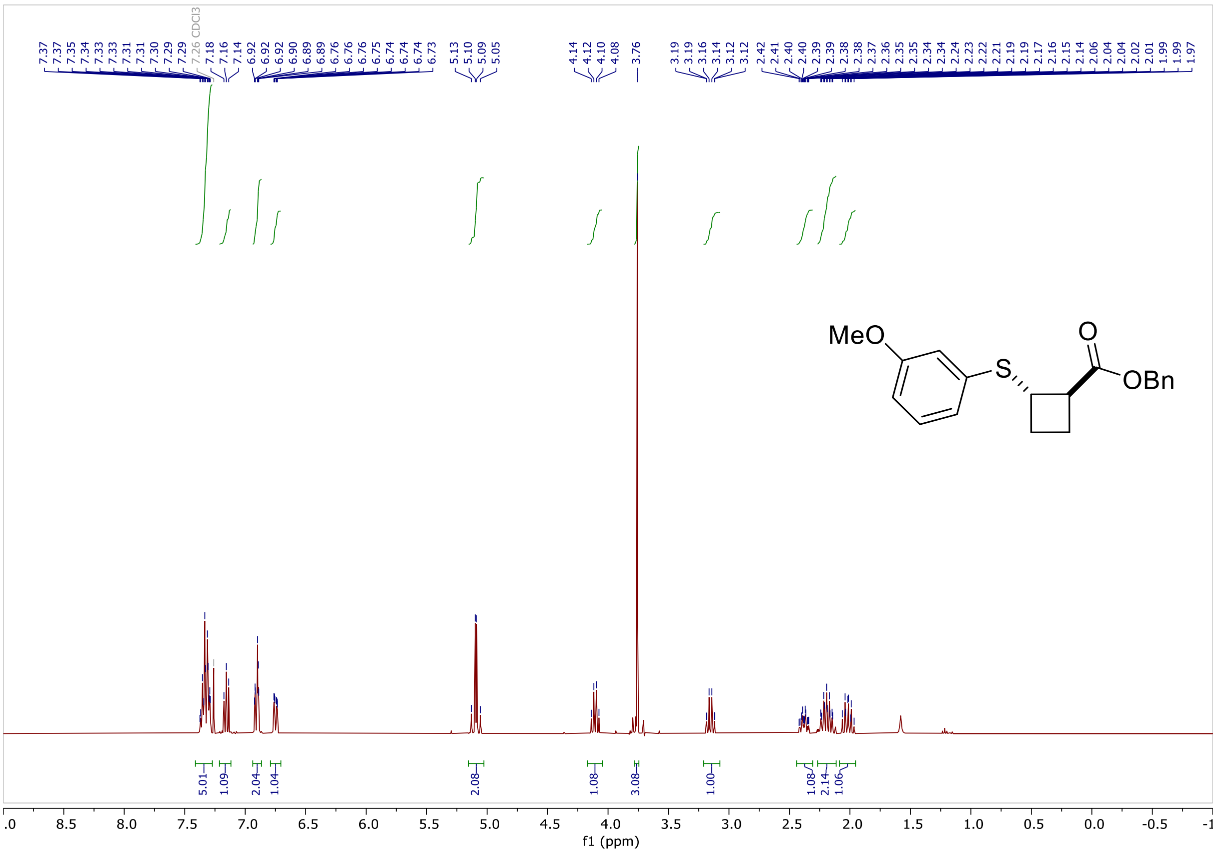

^{13}C-NMR (101 MHz, chloroform-*d*) (3k)

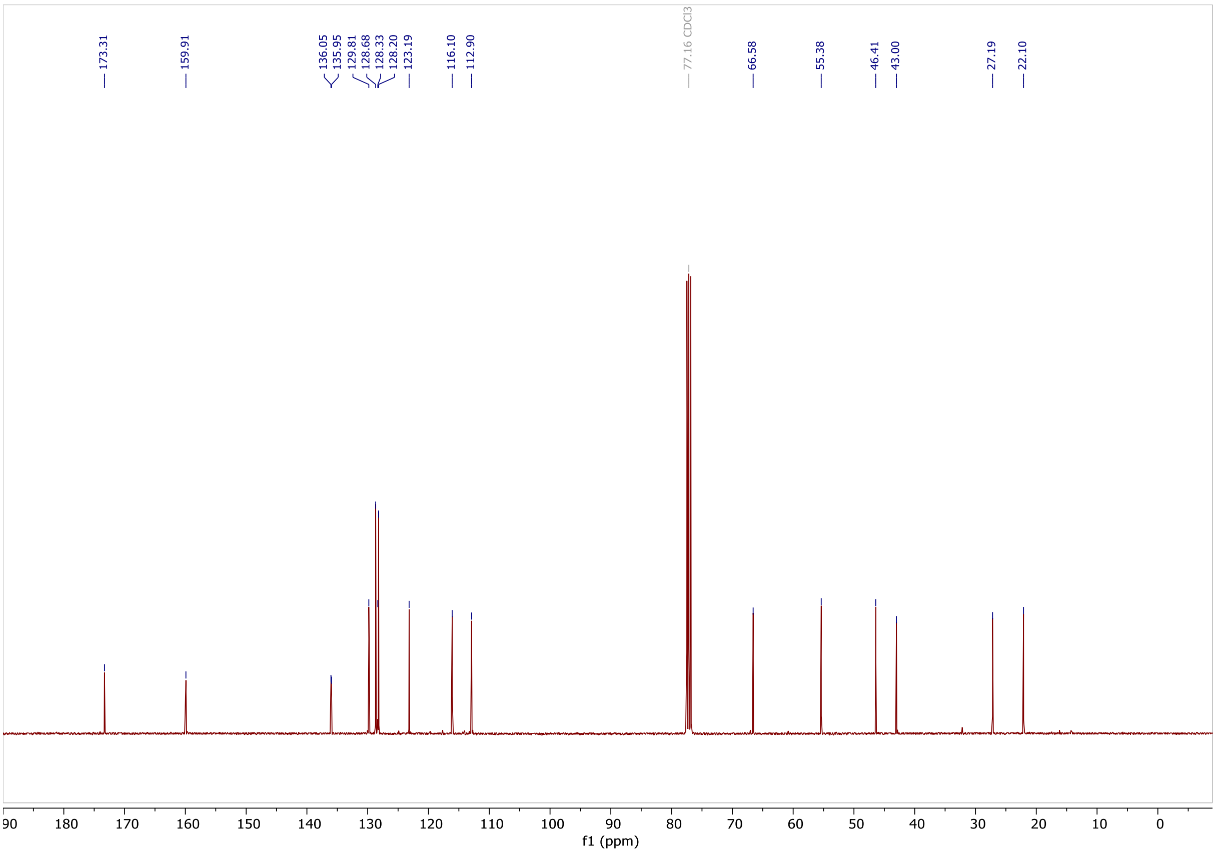

^{1}H-NMR (400 MHz, chloroform-*d*) (3l)

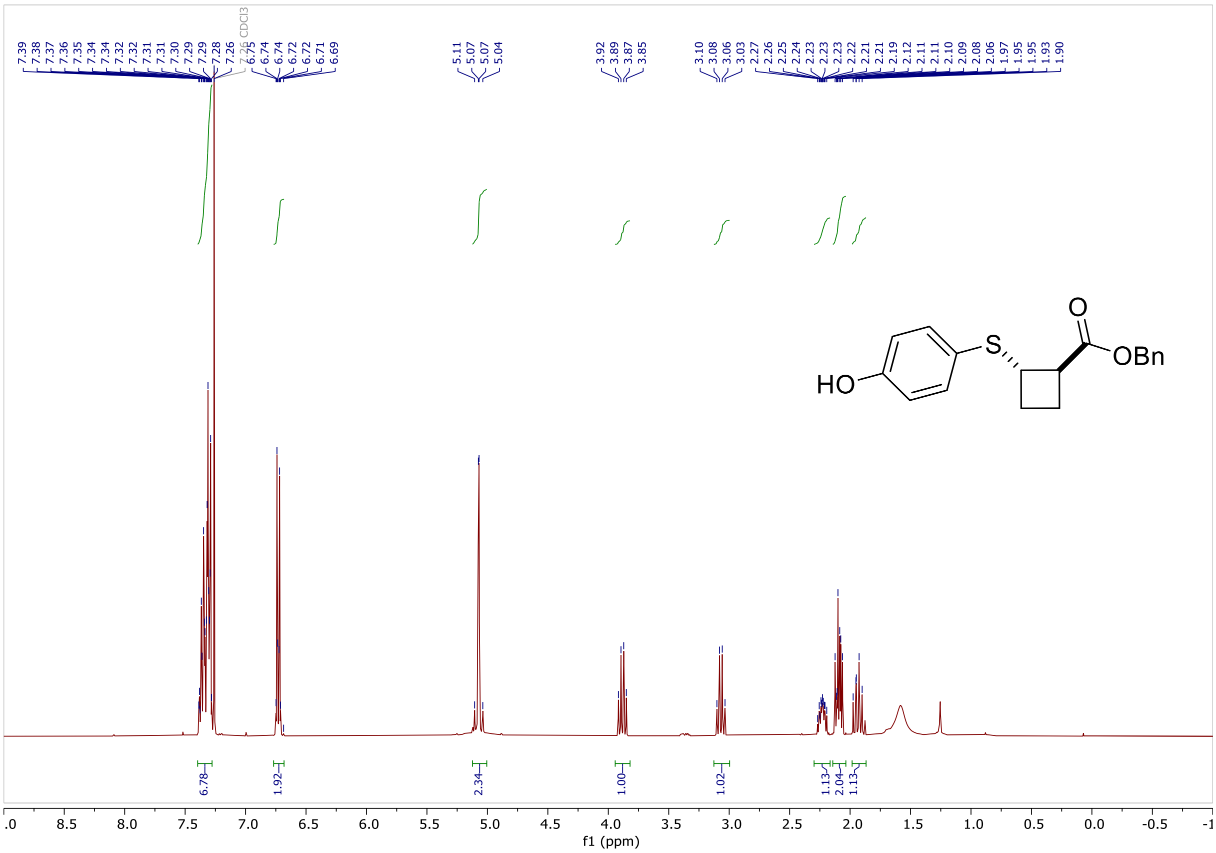

^{13}C-NMR (101 MHz, chloroform-*d*) (3l)

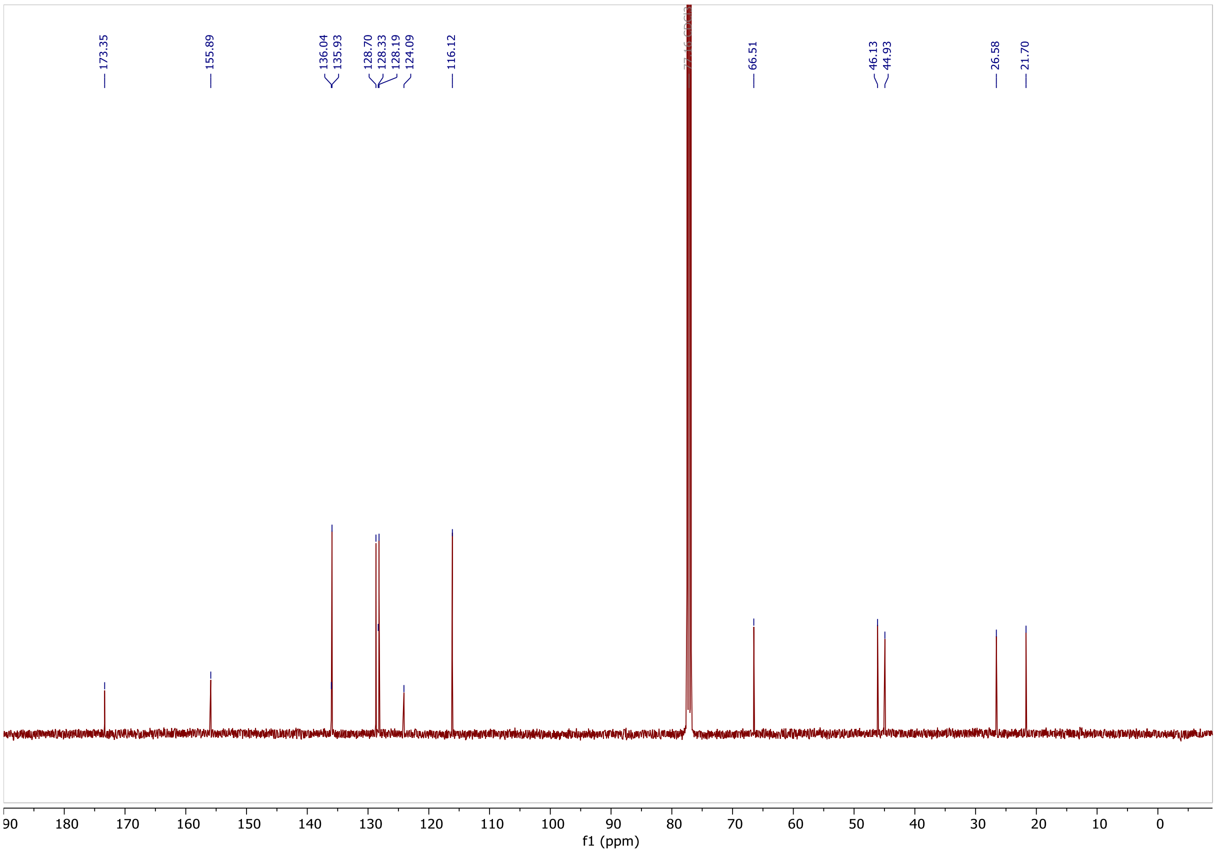

^{1}H-NMR (400 MHz, chloroform-*d*) (3’l)

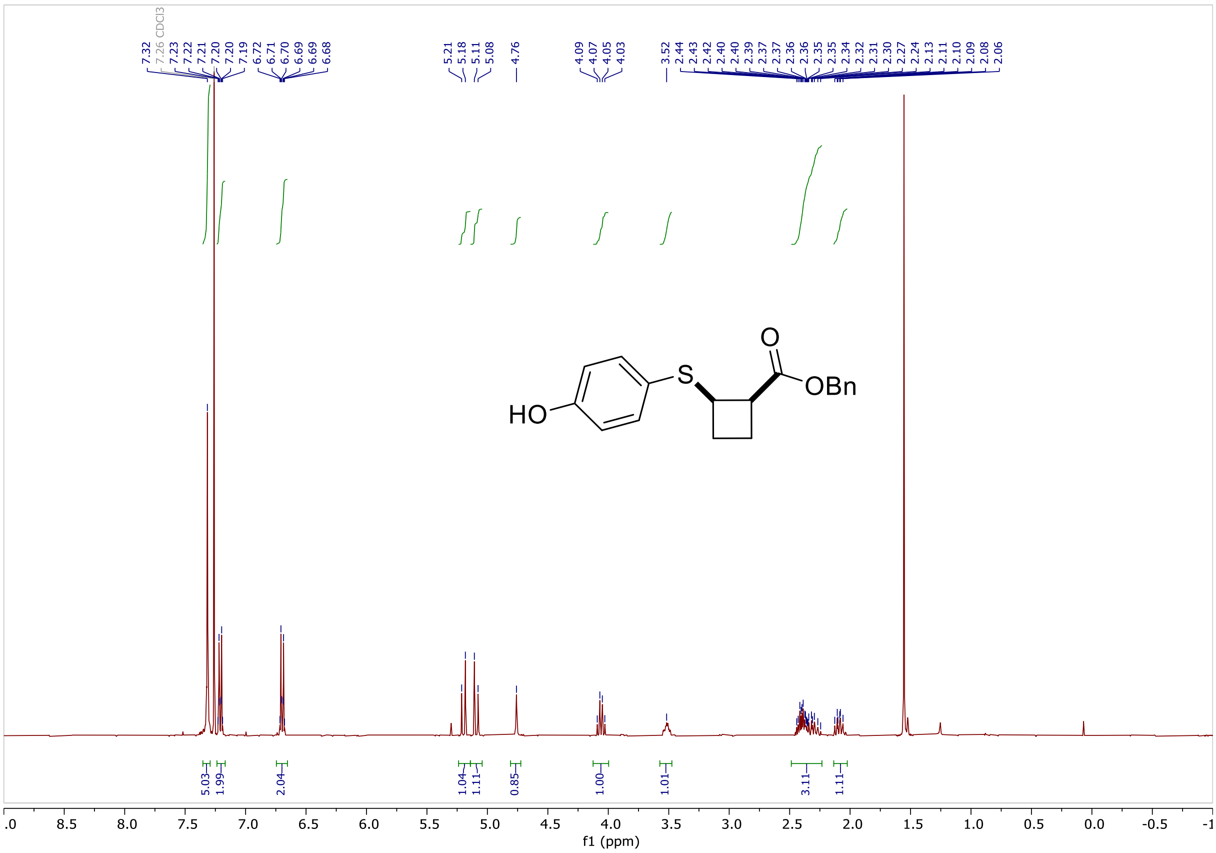

^{13}C-NMR (101 MHz, chloroform-*d*) (3’l)

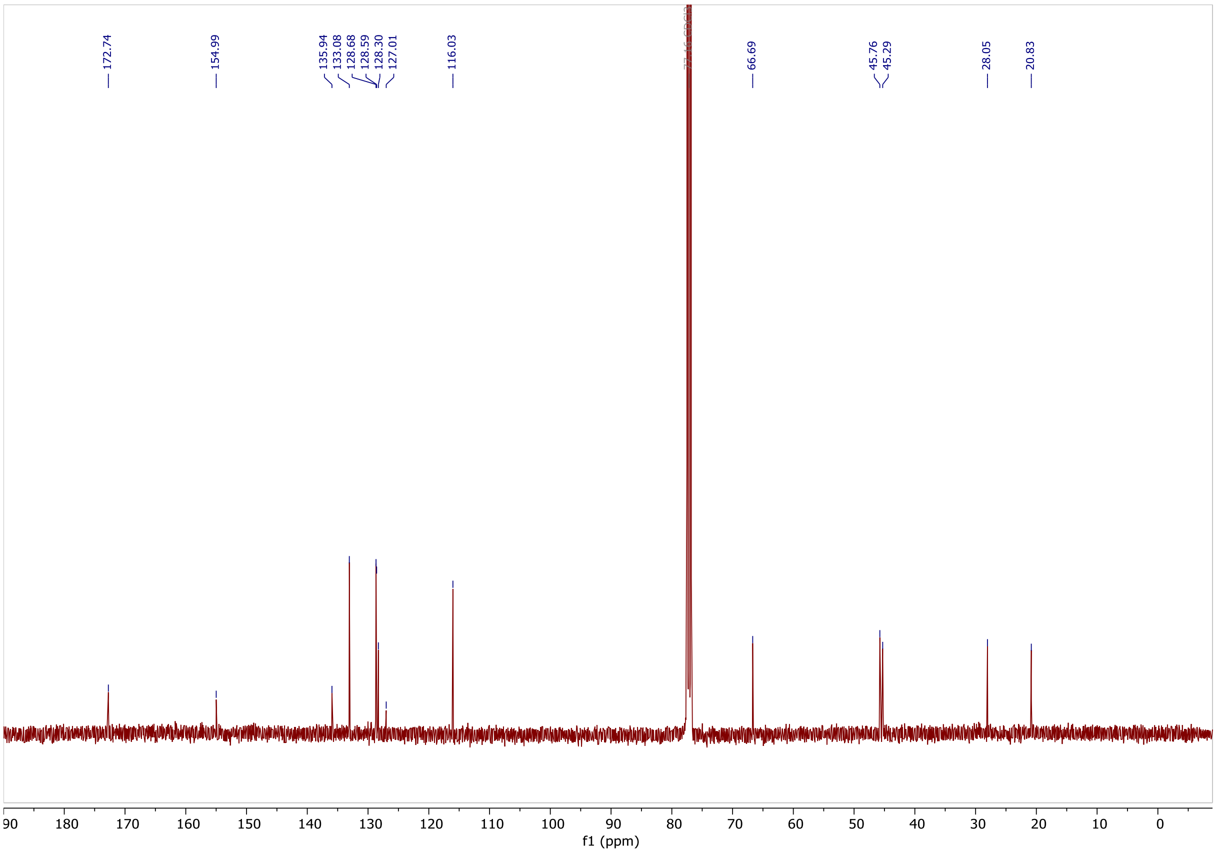

^{1}H-NMR (400 MHz, chloroform-*d*) (3m)

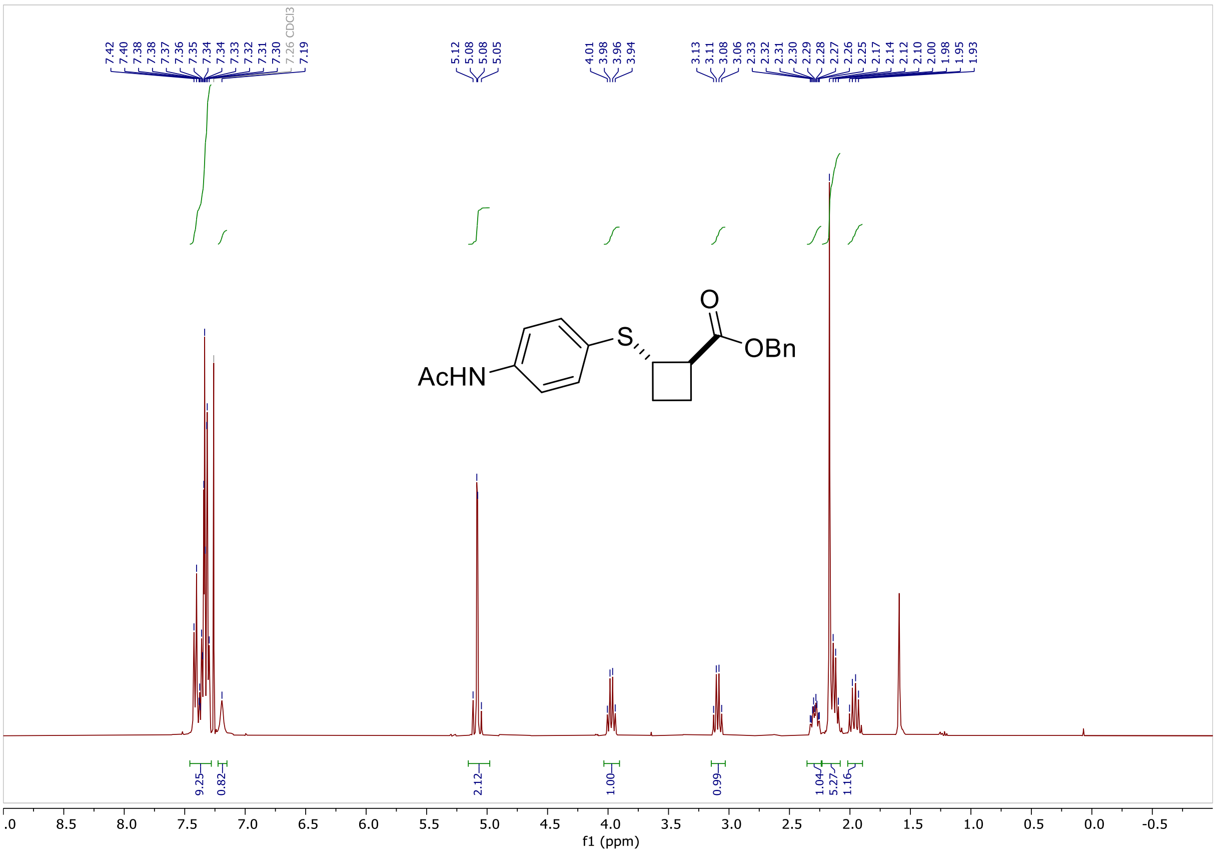

^{13}C-NMR (101 MHz, chloroform-*d*) (3m)

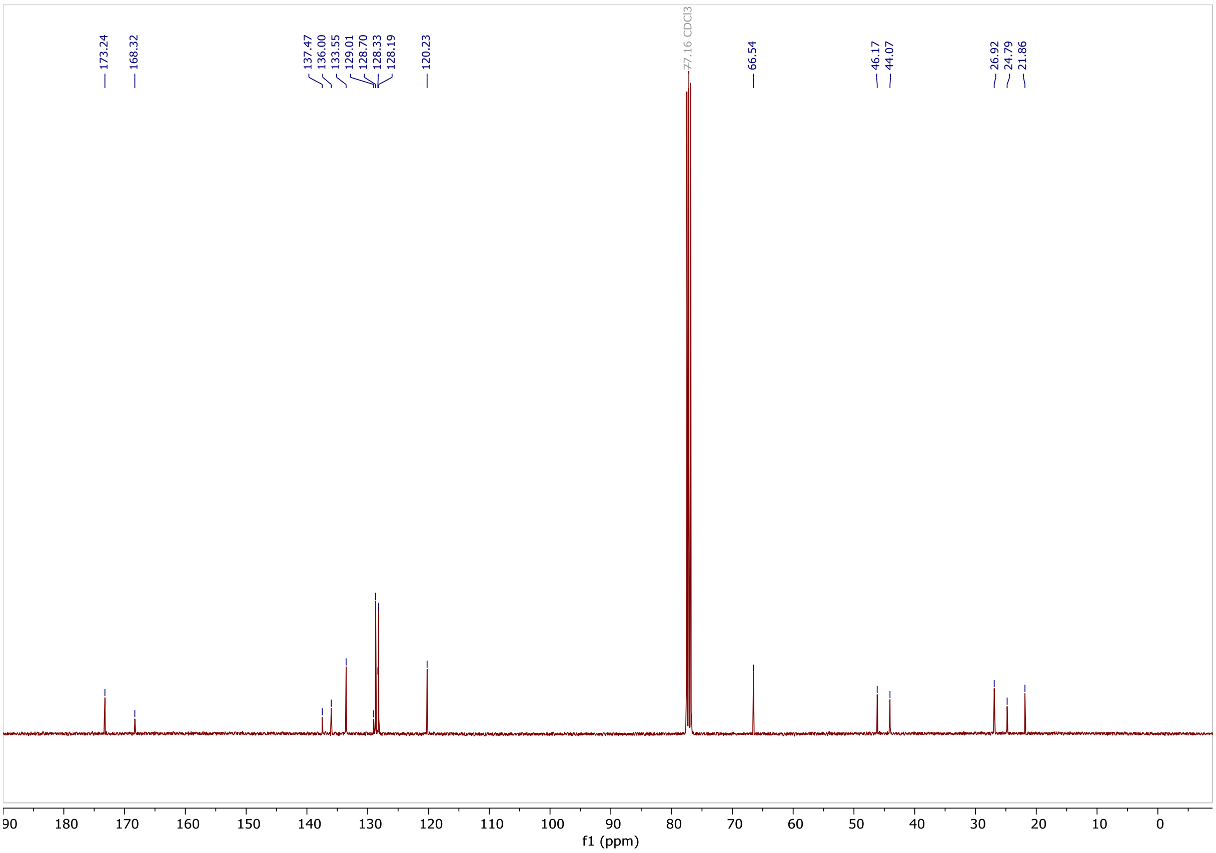

^{1}H-NMR (400 MHz, chloroform-*d*) (3n)

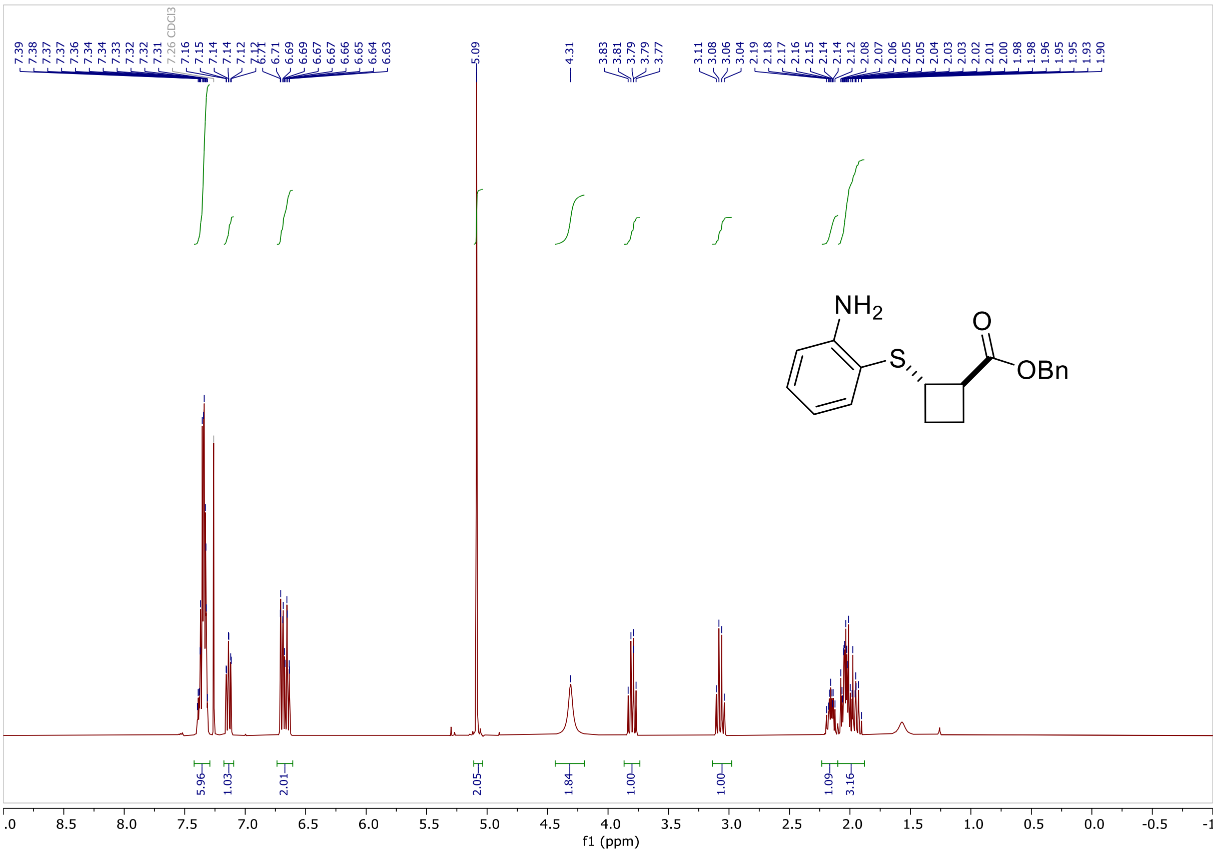

^{13}C-NMR (101 MHz, chloroform-*d*) (3n)

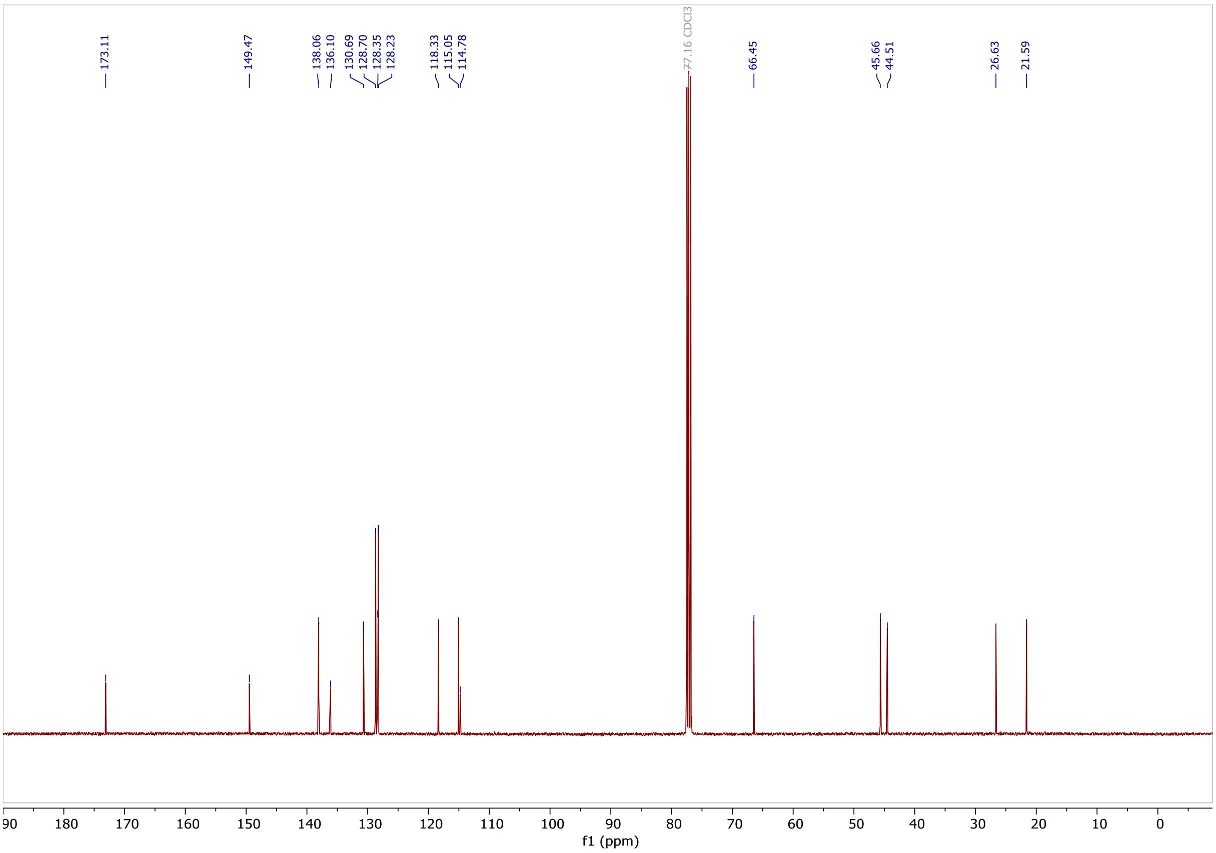

^{1}H-NMR (400 MHz, chloroform-*d*) (3o)

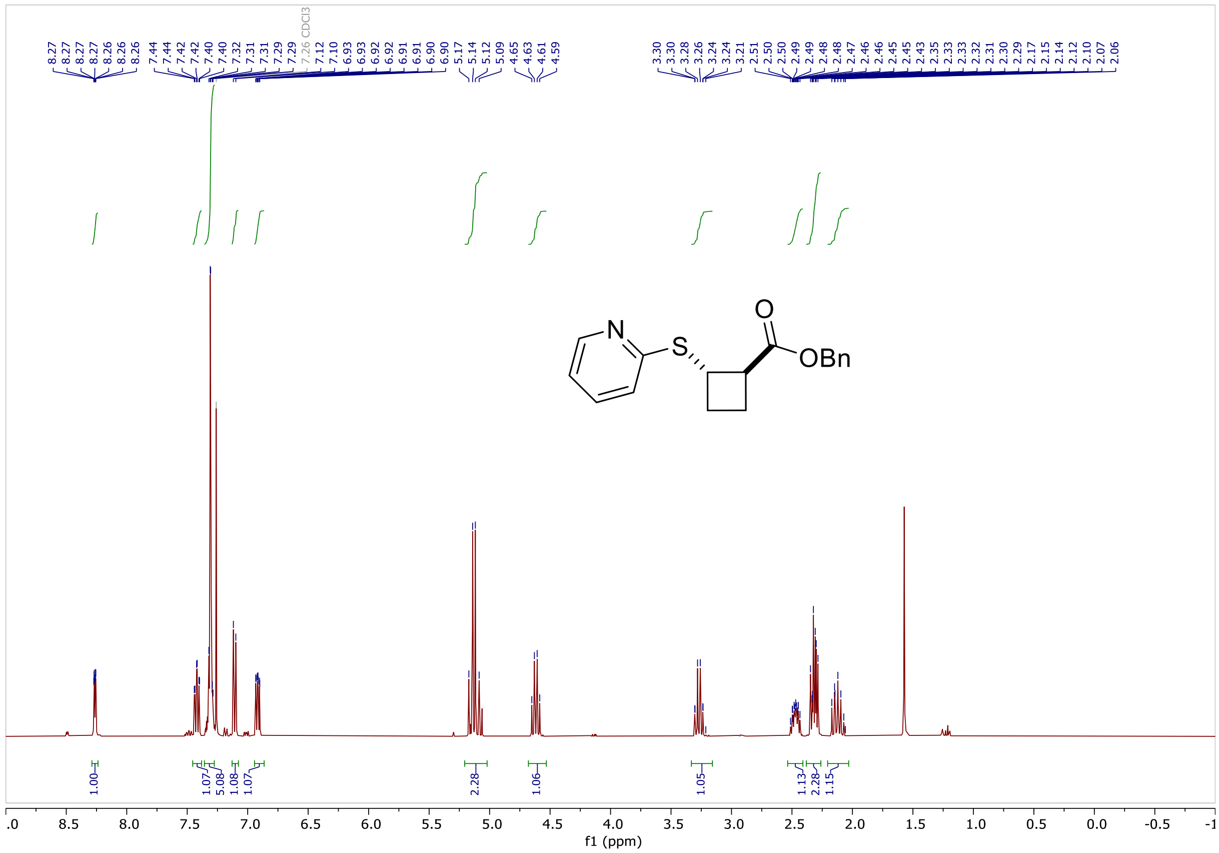

^{13}C-NMR (101 MHz, chloroform-*d*) (3o)

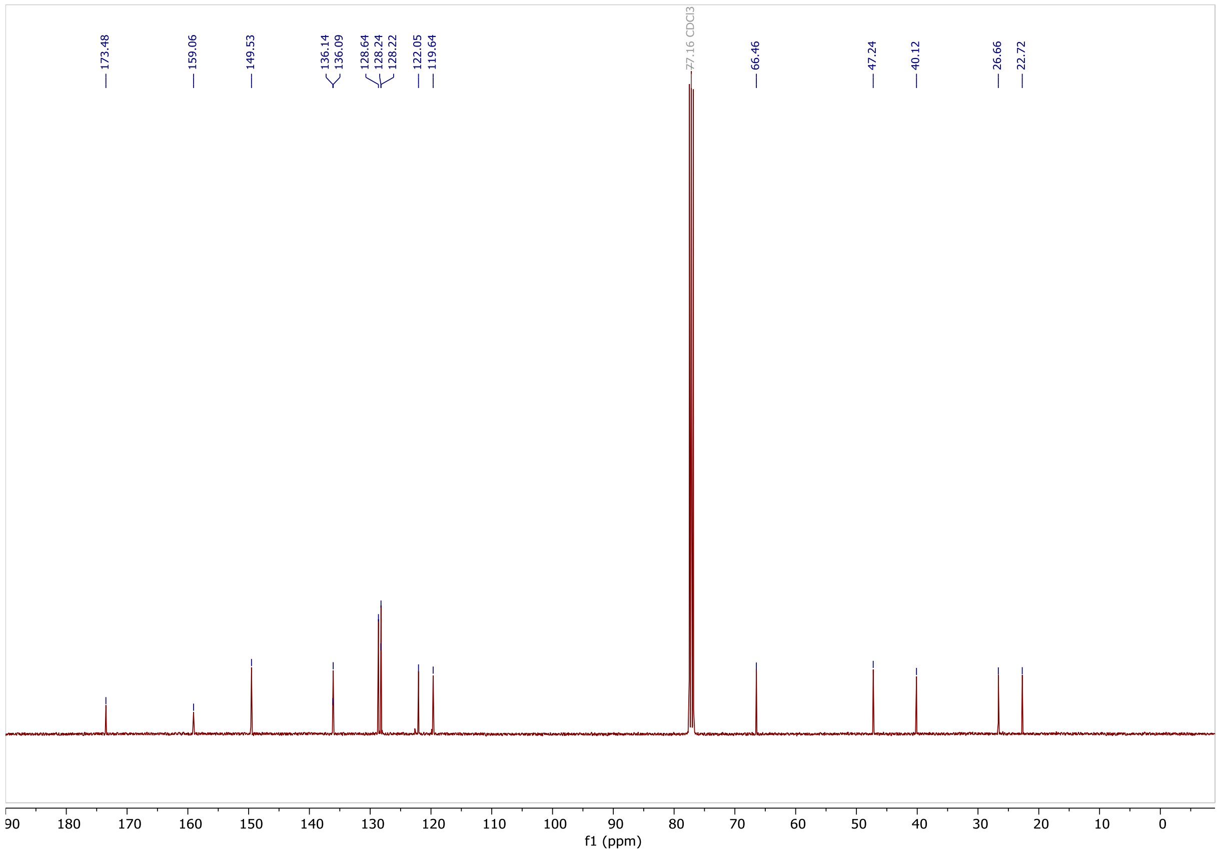

^{1}H-NMR (400 MHz, chloroform-*d*) (3p)

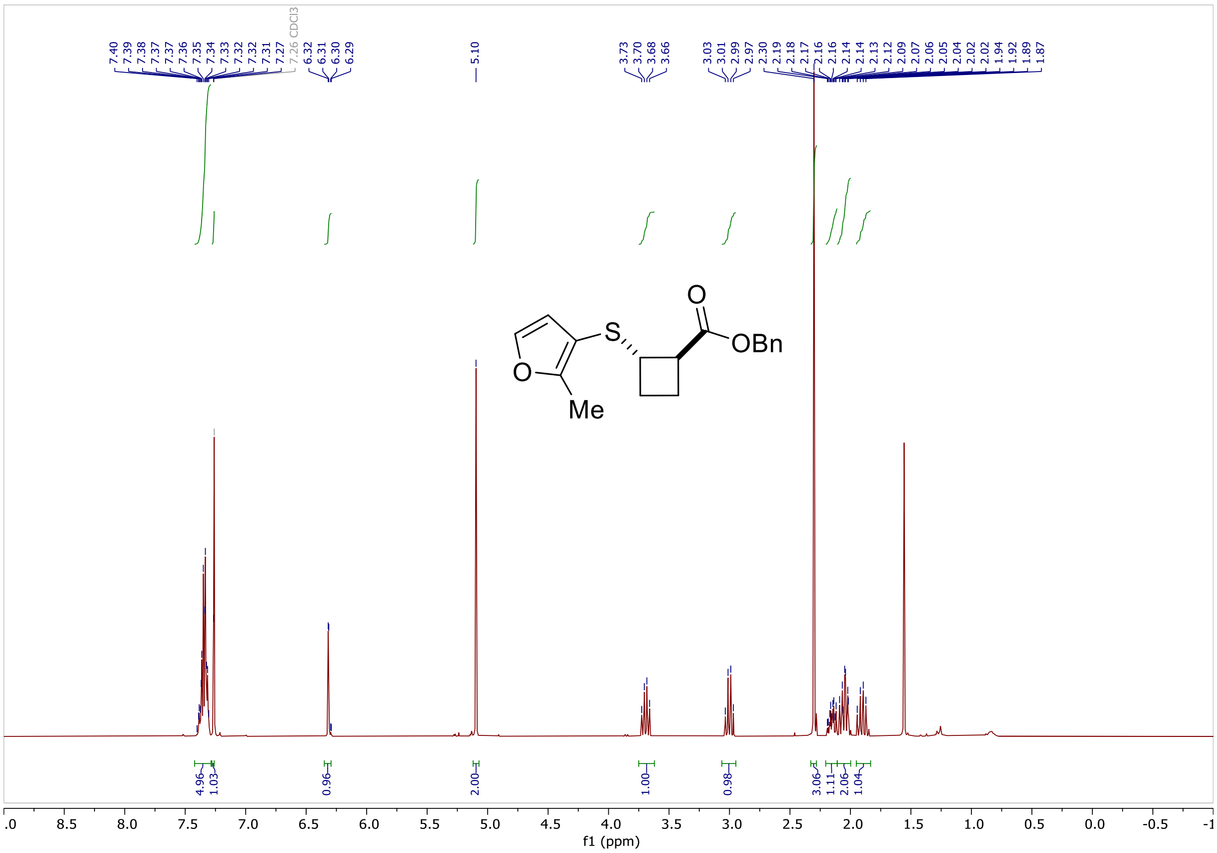

^{13}C-NMR (101 MHz, chloroform-*d*) (3p)

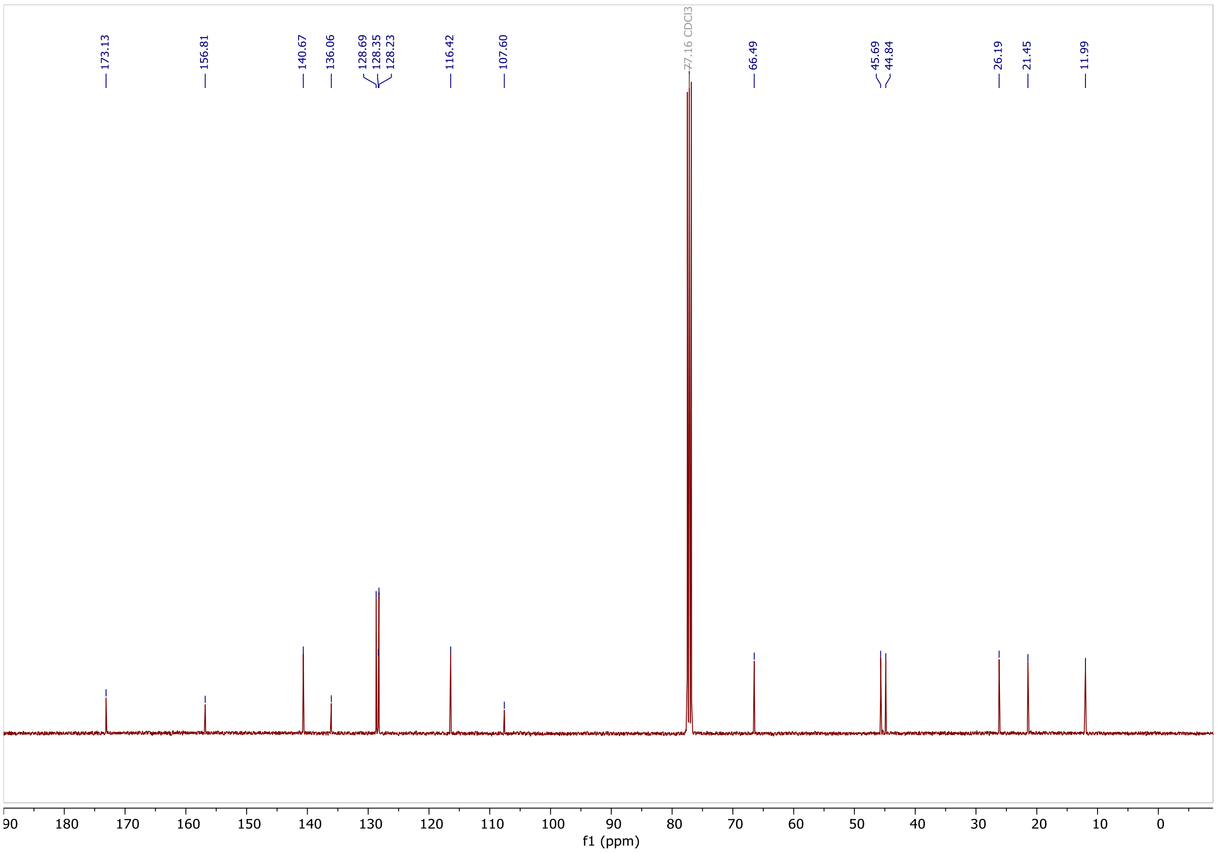

^{1}H-NMR (400 MHz, chloroform-*d*) (3q)

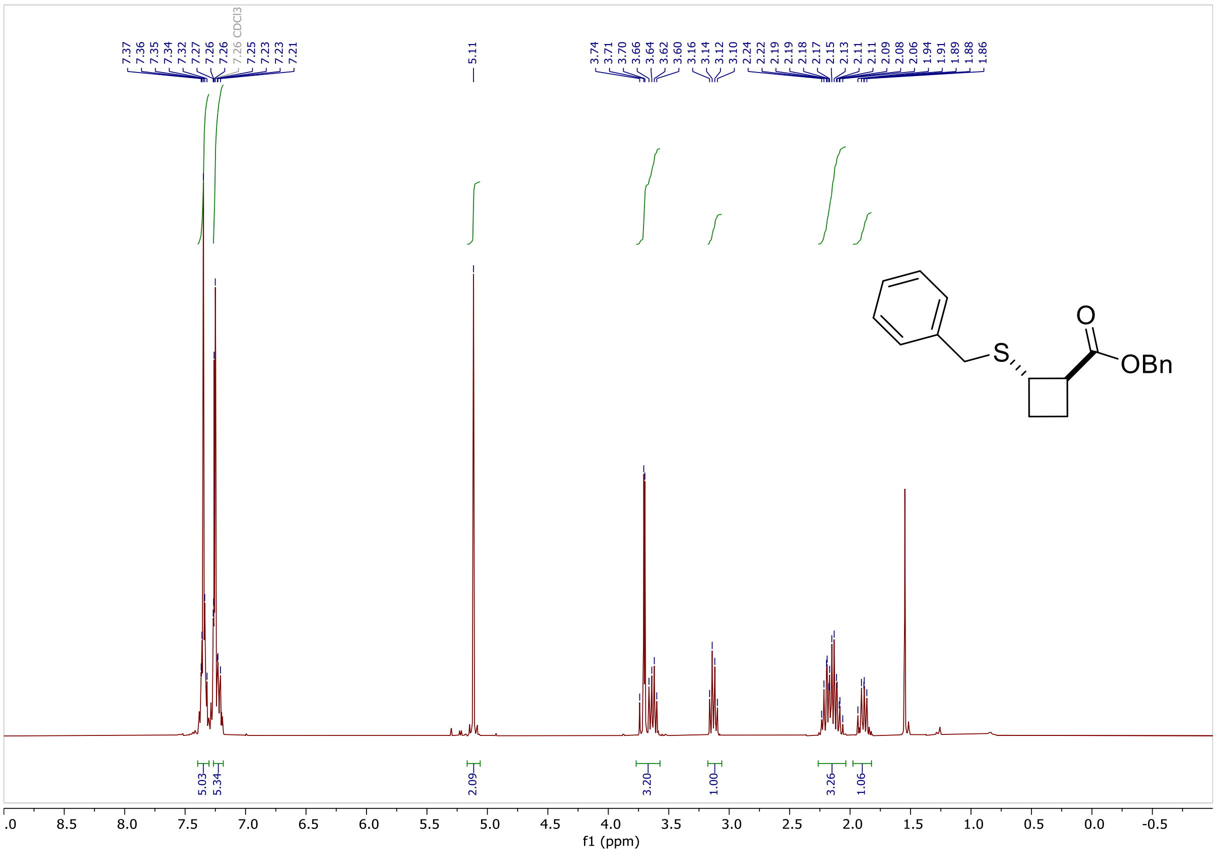

^{13}C-NMR (101 MHz, chloroform-*d*) (3q)

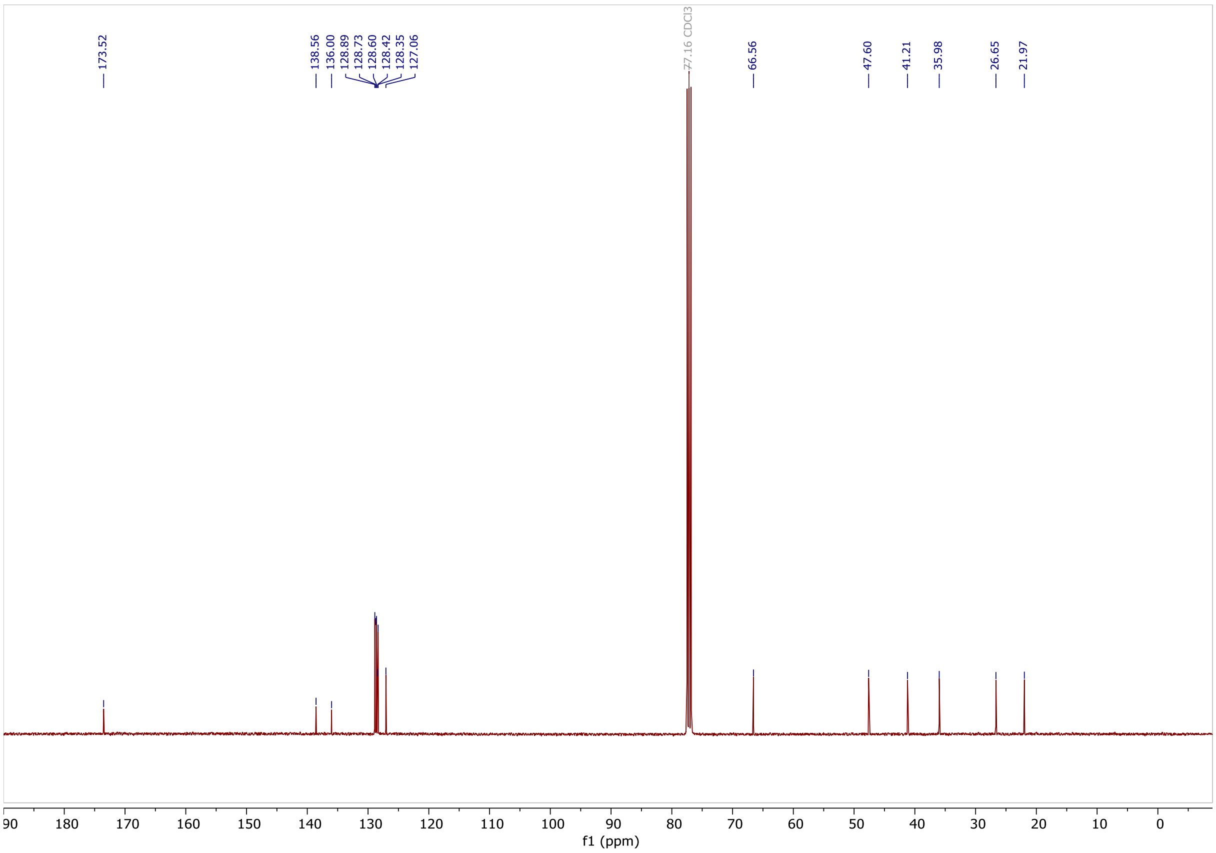

^{1}H-NMR (400 MHz, chloroform-*d*) (3r)

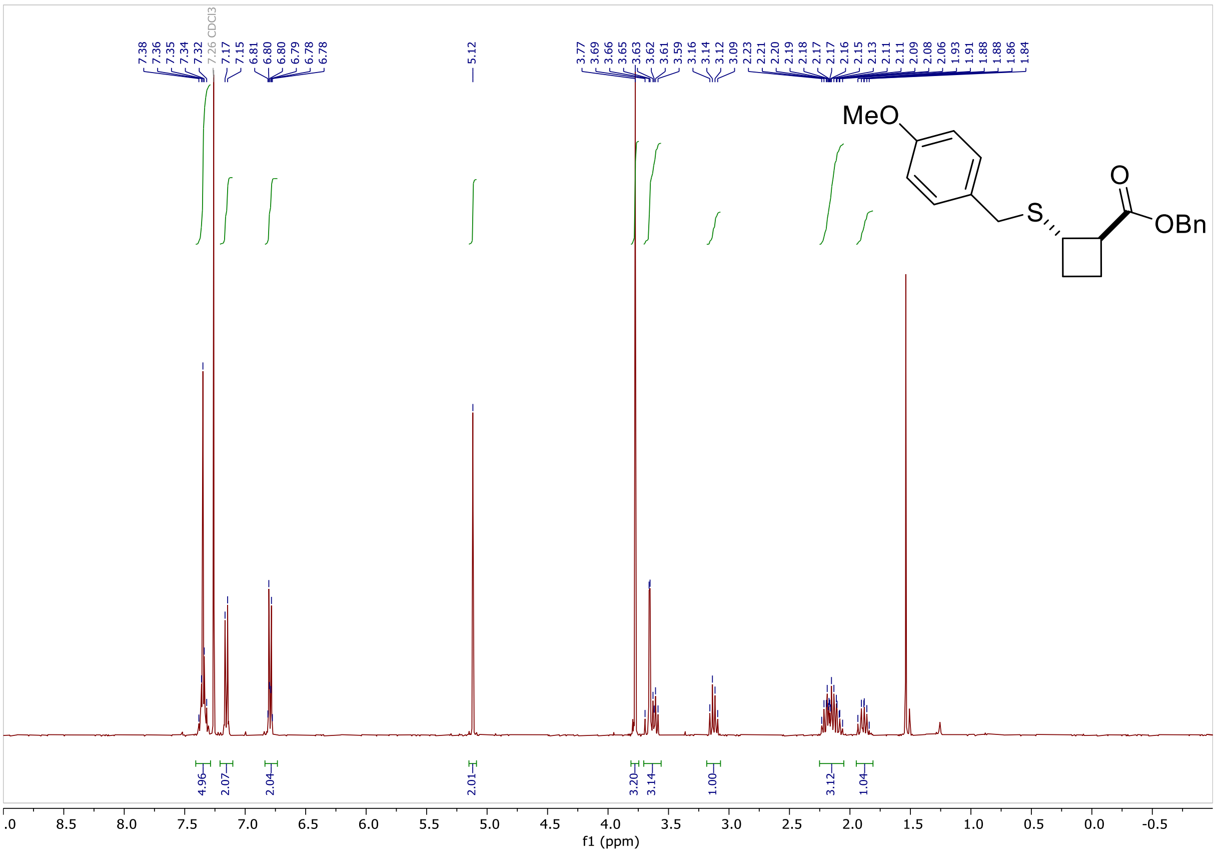

^{13}C-NMR (101 MHz, chloroform-*d*) (3r)

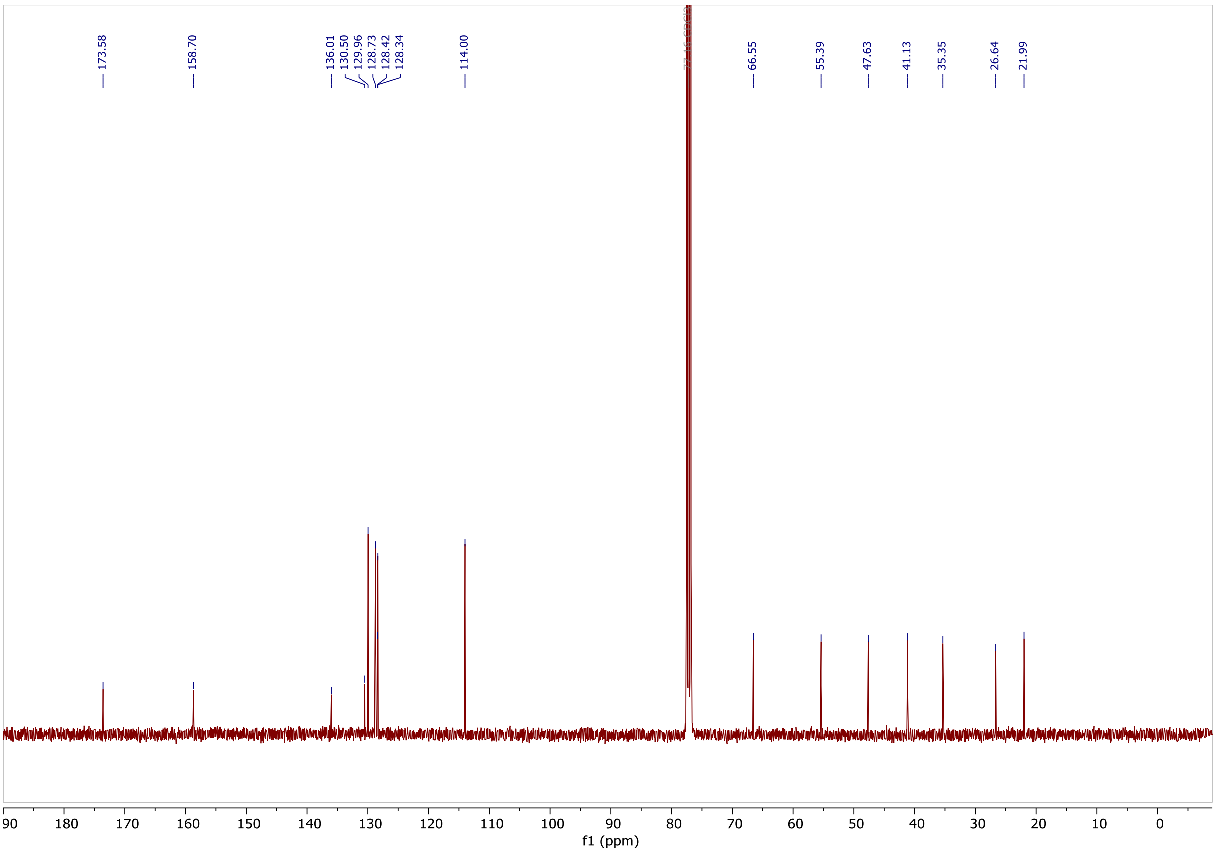

^{1}H-NMR (400 MHz, chloroform-*d*) (3s)

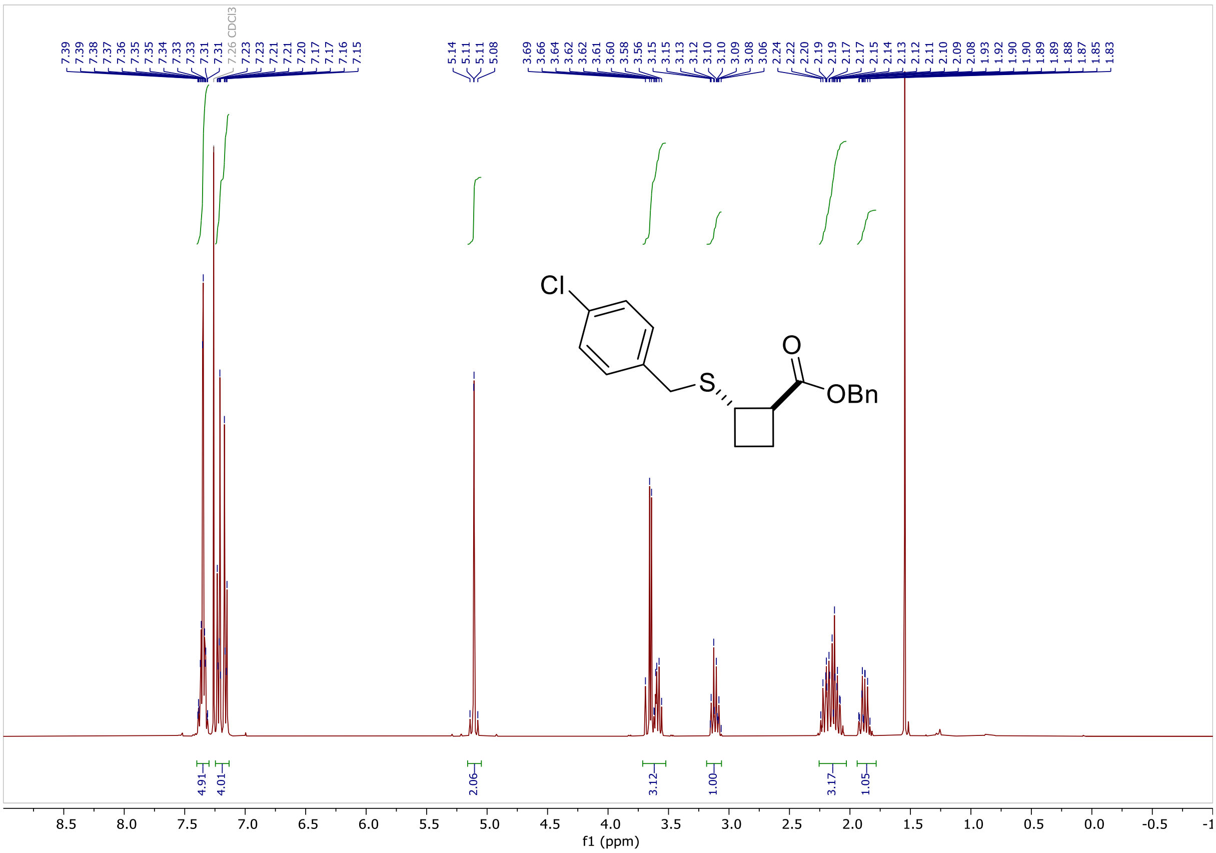

^{13}C-NMR (101 MHz, chloroform-*d*) (3s)

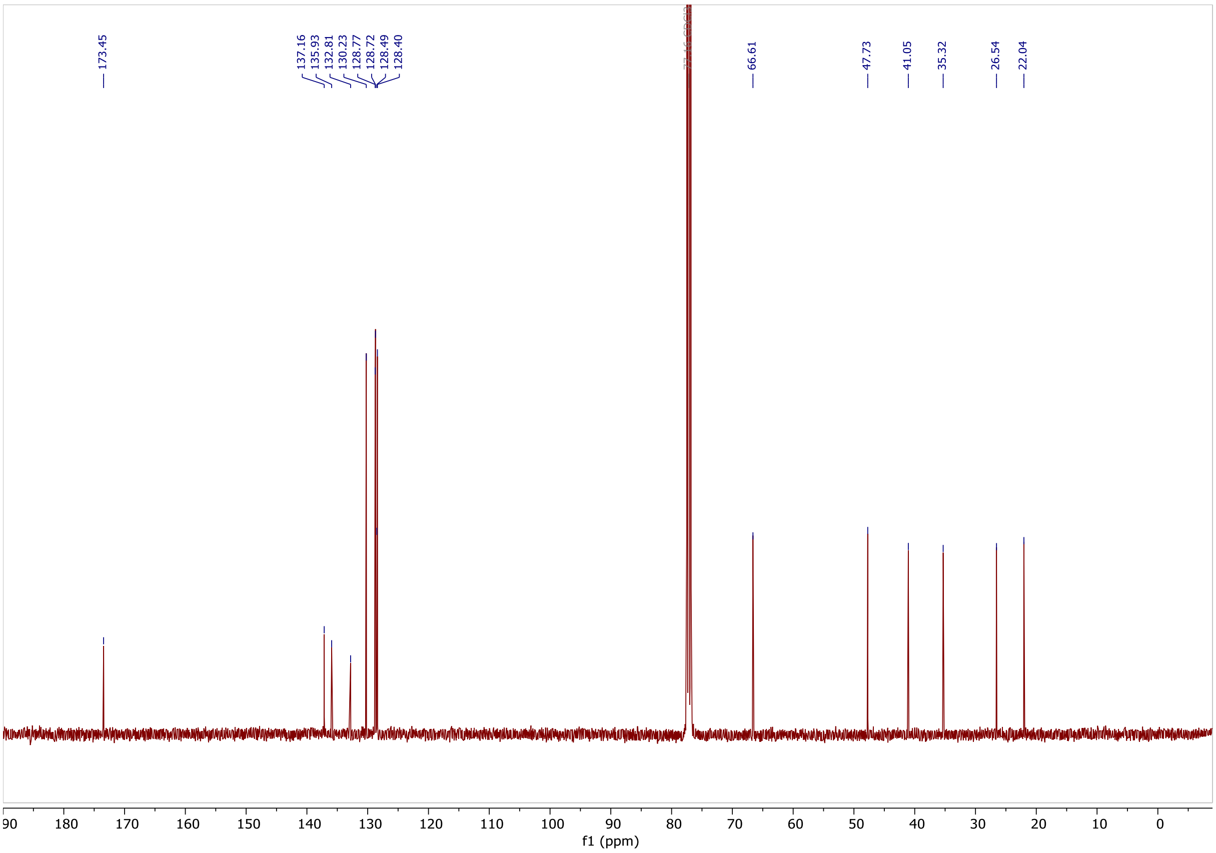

^{1}H-NMR (400 MHz, chloroform-*d*) (3t)

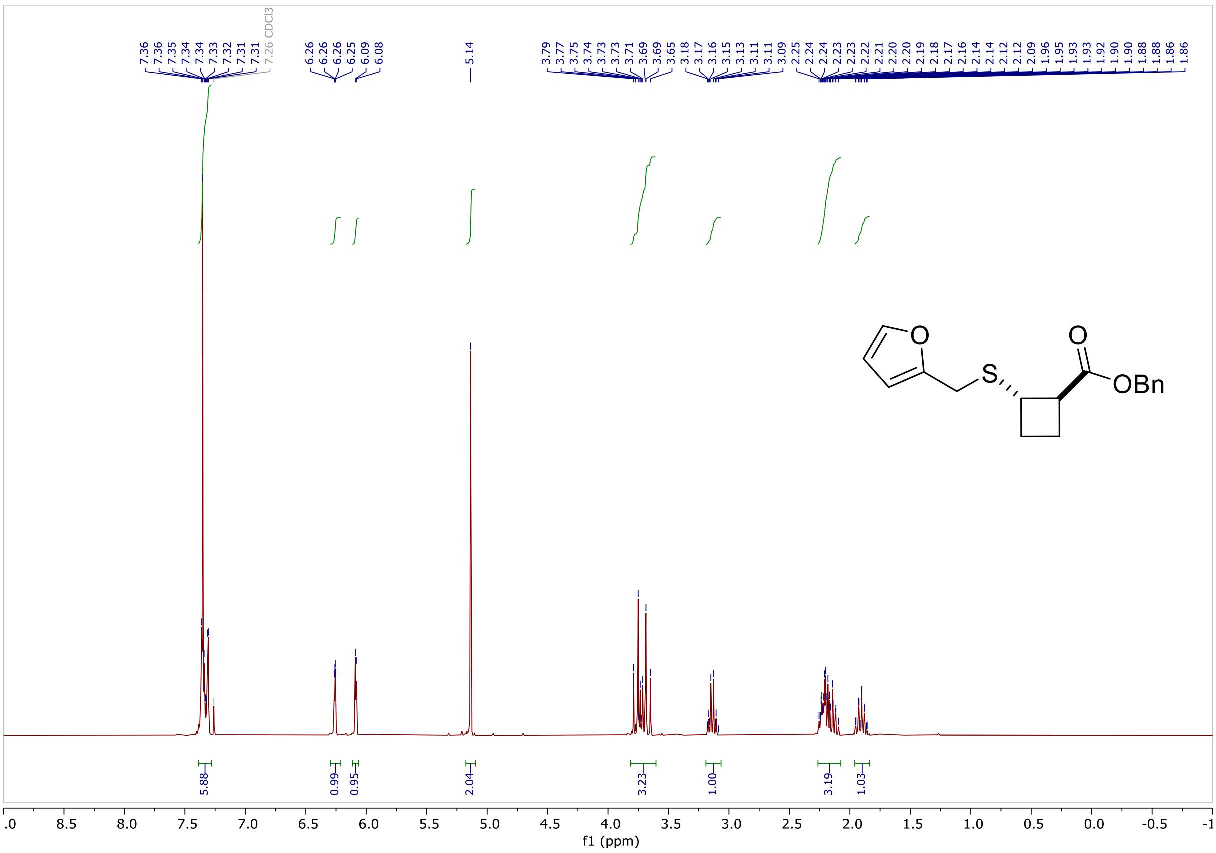

^{13}C-NMR (101 MHz, chloroform-*d*) (3t)

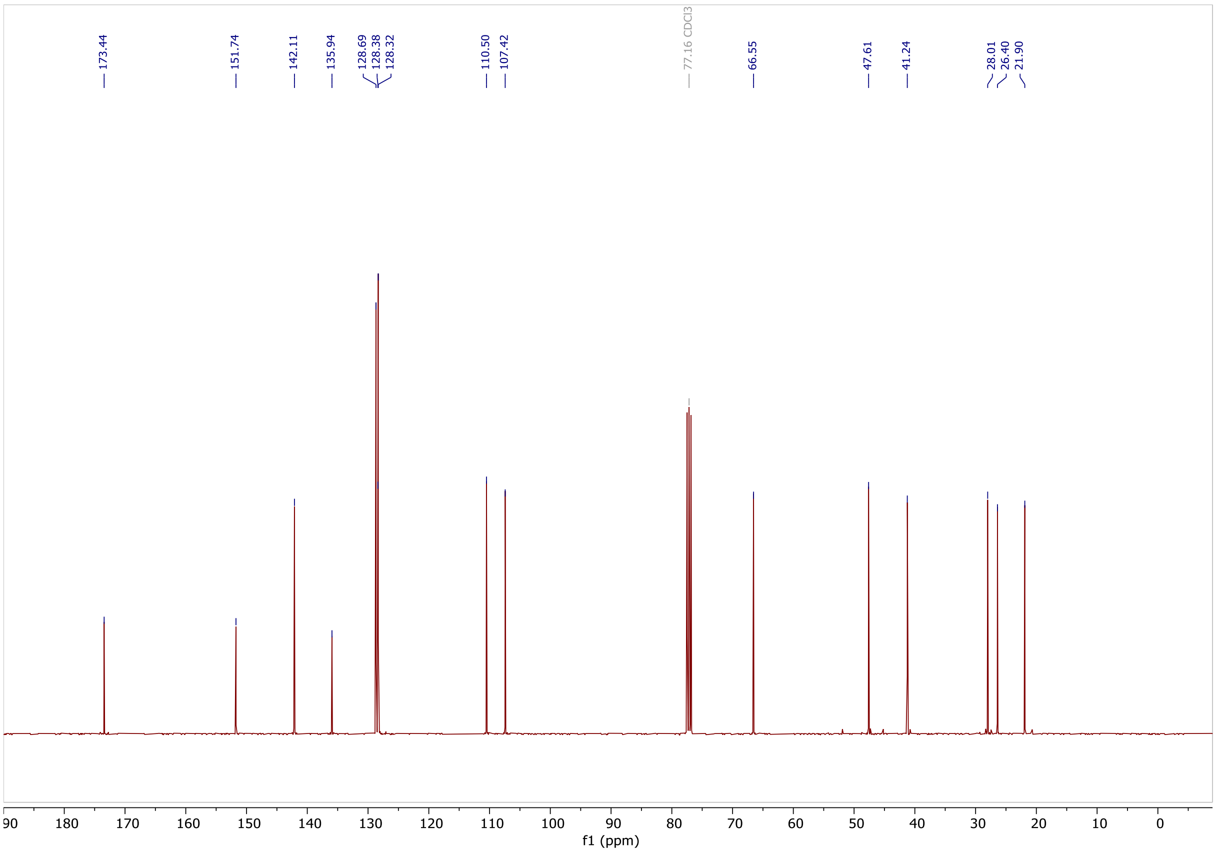

^{1}H-NMR (400 MHz, chloroform-*d*) (3u)

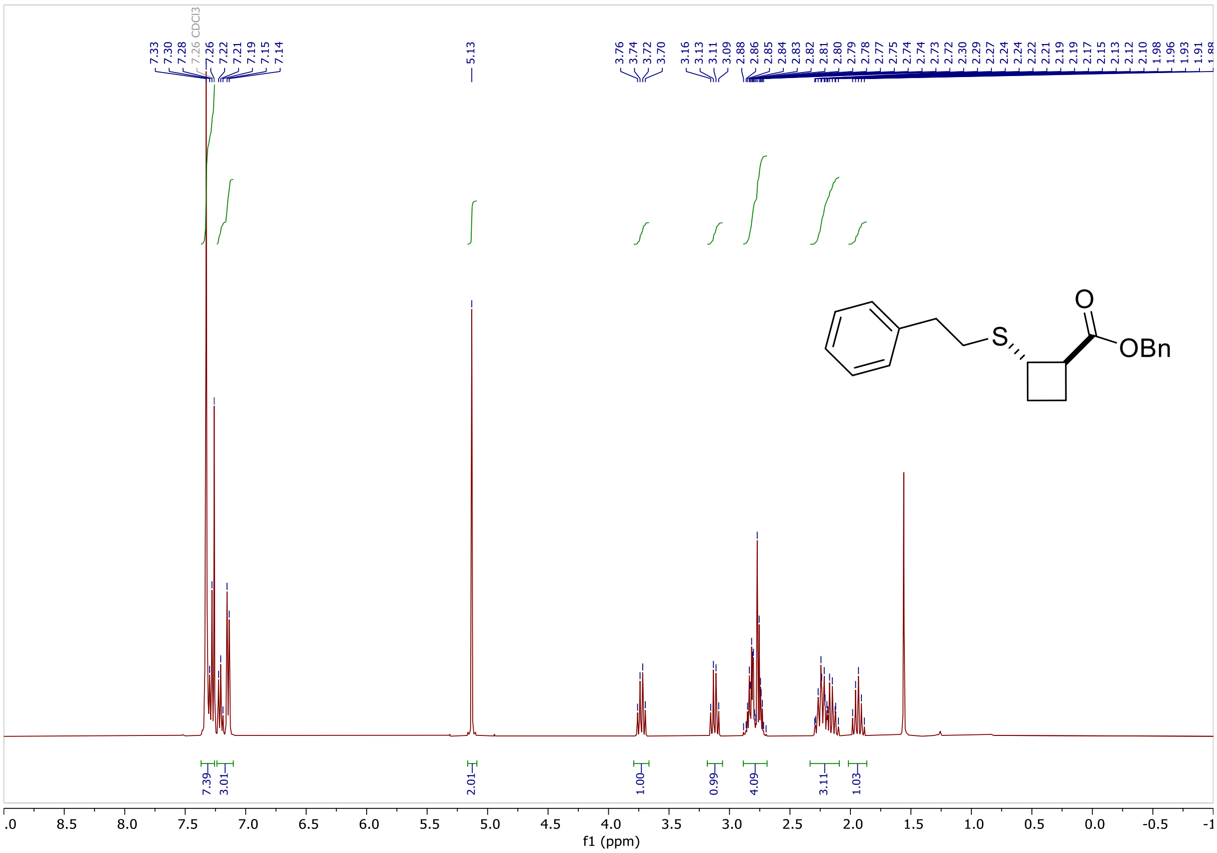

^{13}C-NMR (101 MHz, chloroform-*d*) (3u)

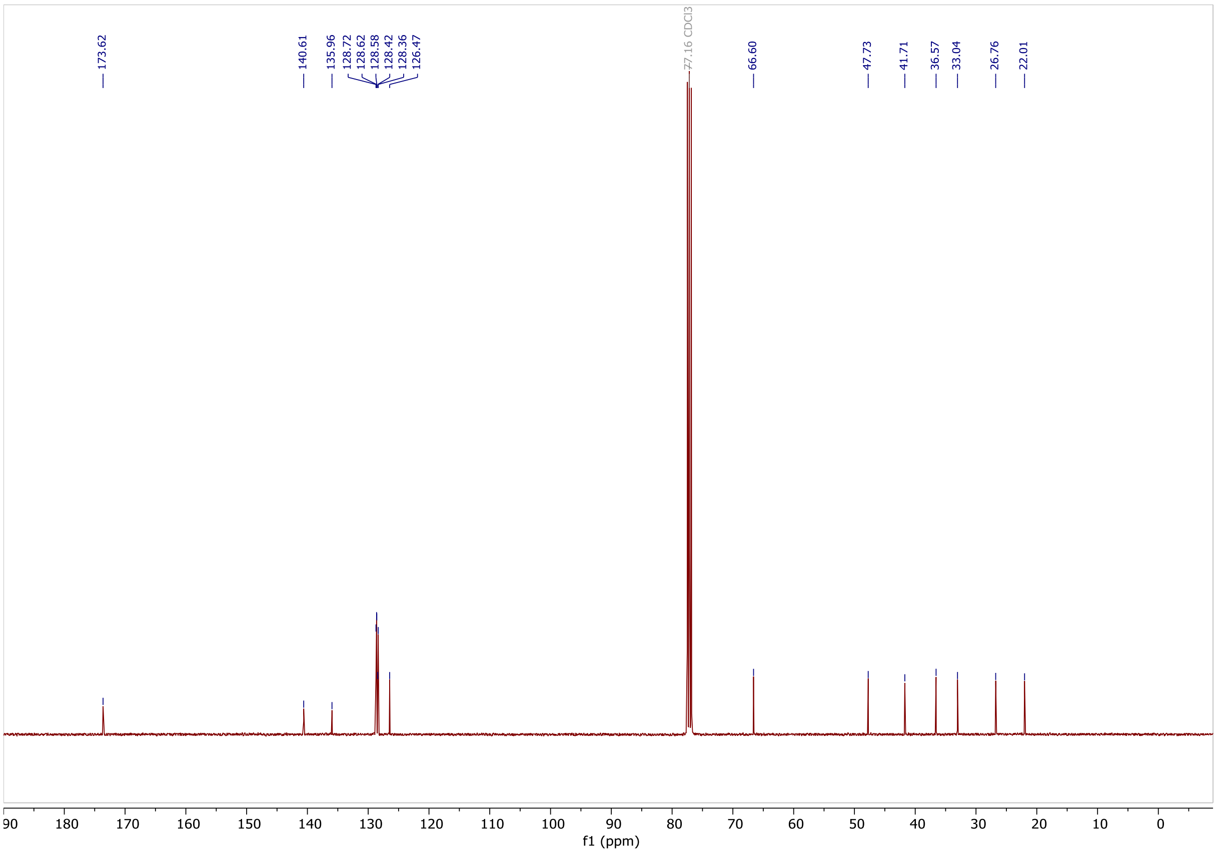

^{1}H-NMR (400 MHz, chloroform-*d*) (3v)

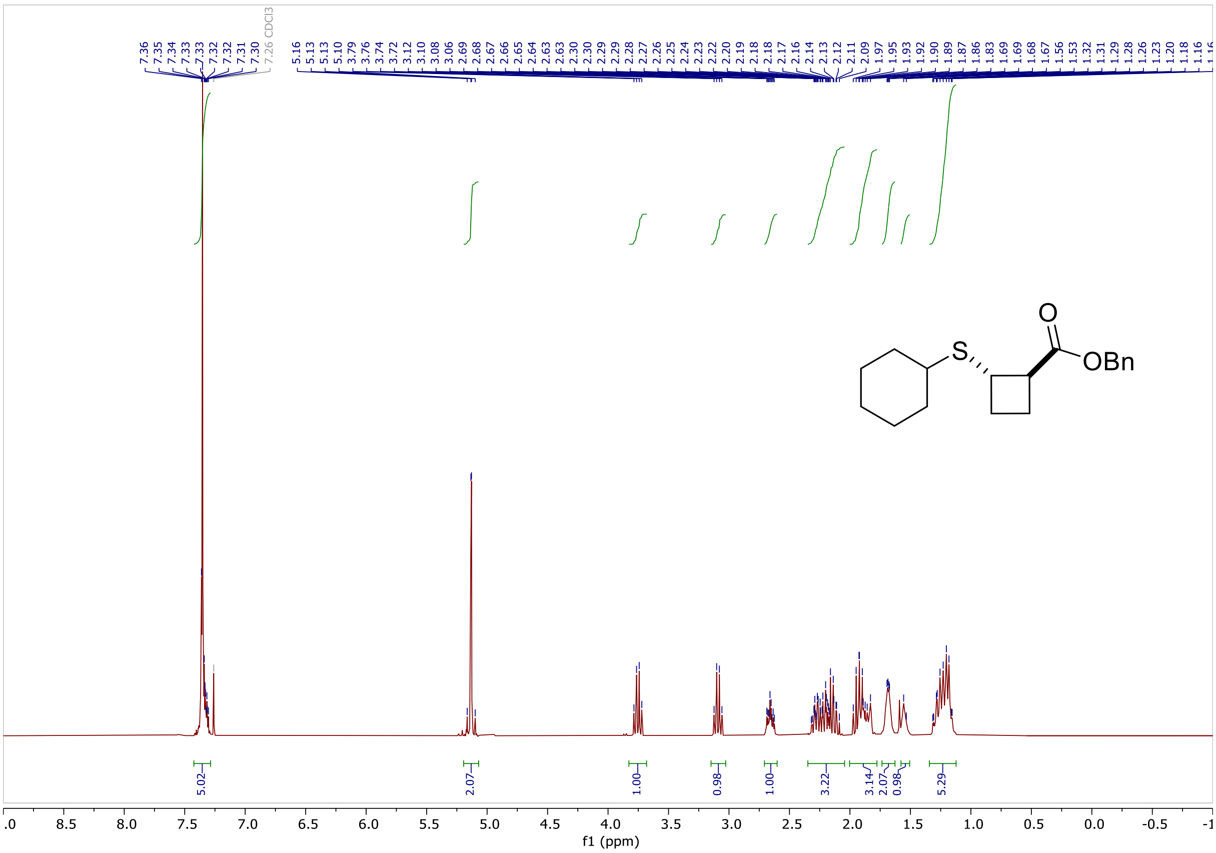

^{13}C-NMR (101 MHz, chloroform-*d*) (3v)

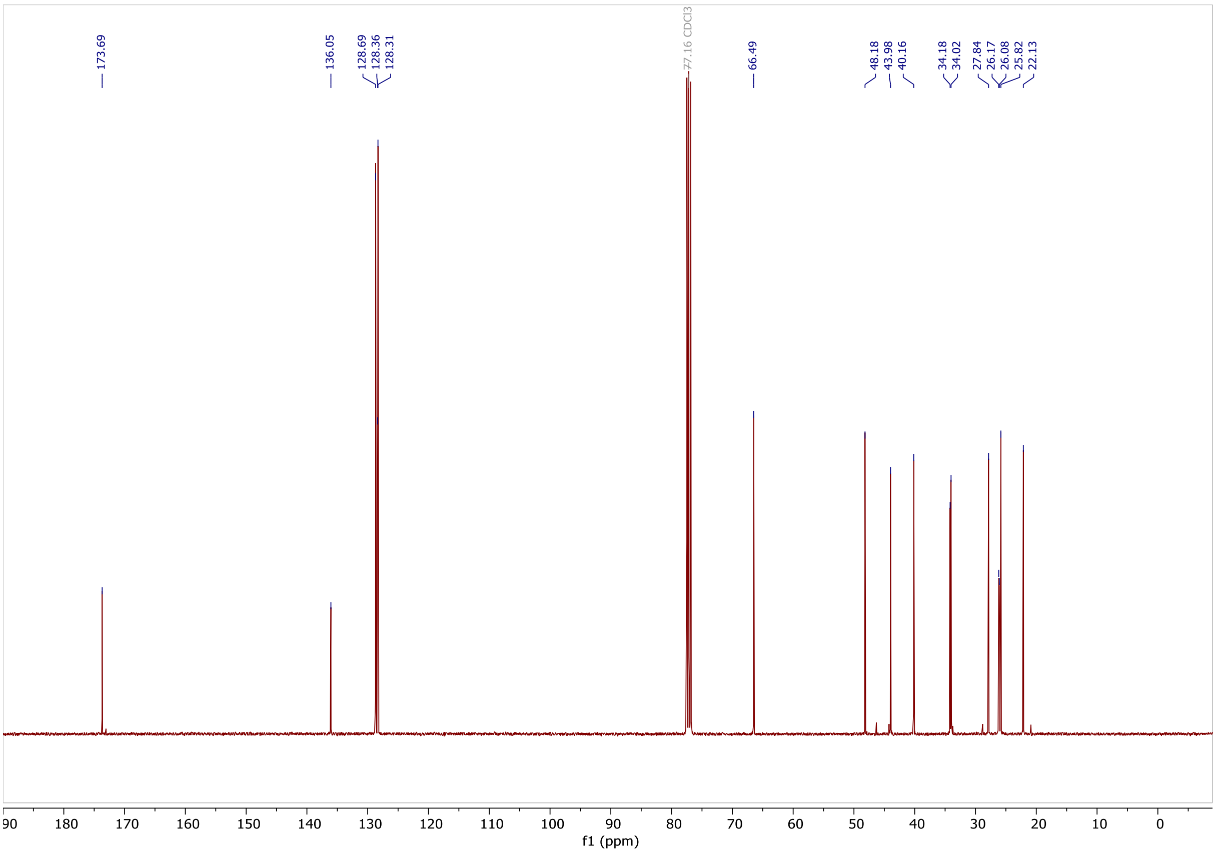

^{1}H-NMR (400 MHz, chloroform-*d*) (3w)

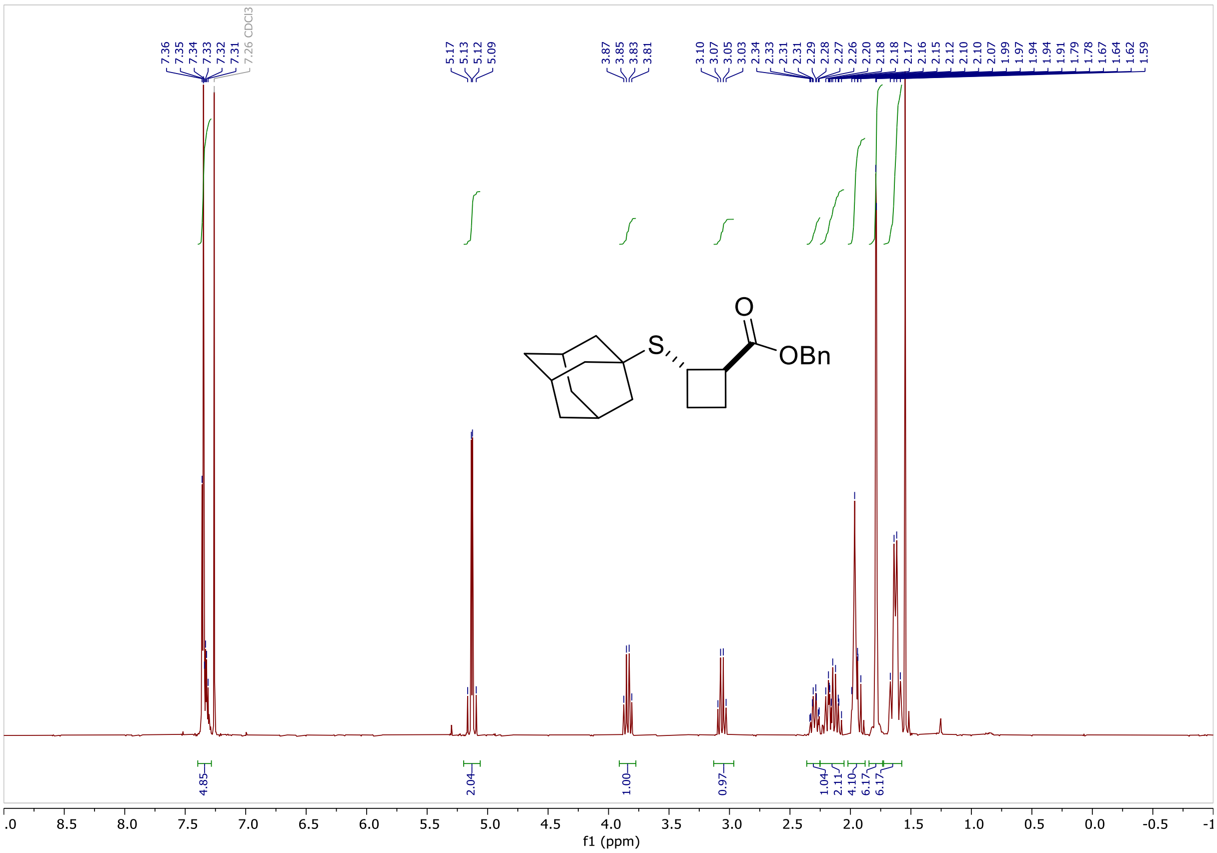

^{13}C-NMR (101 MHz, chloroform-*d*) (3w)

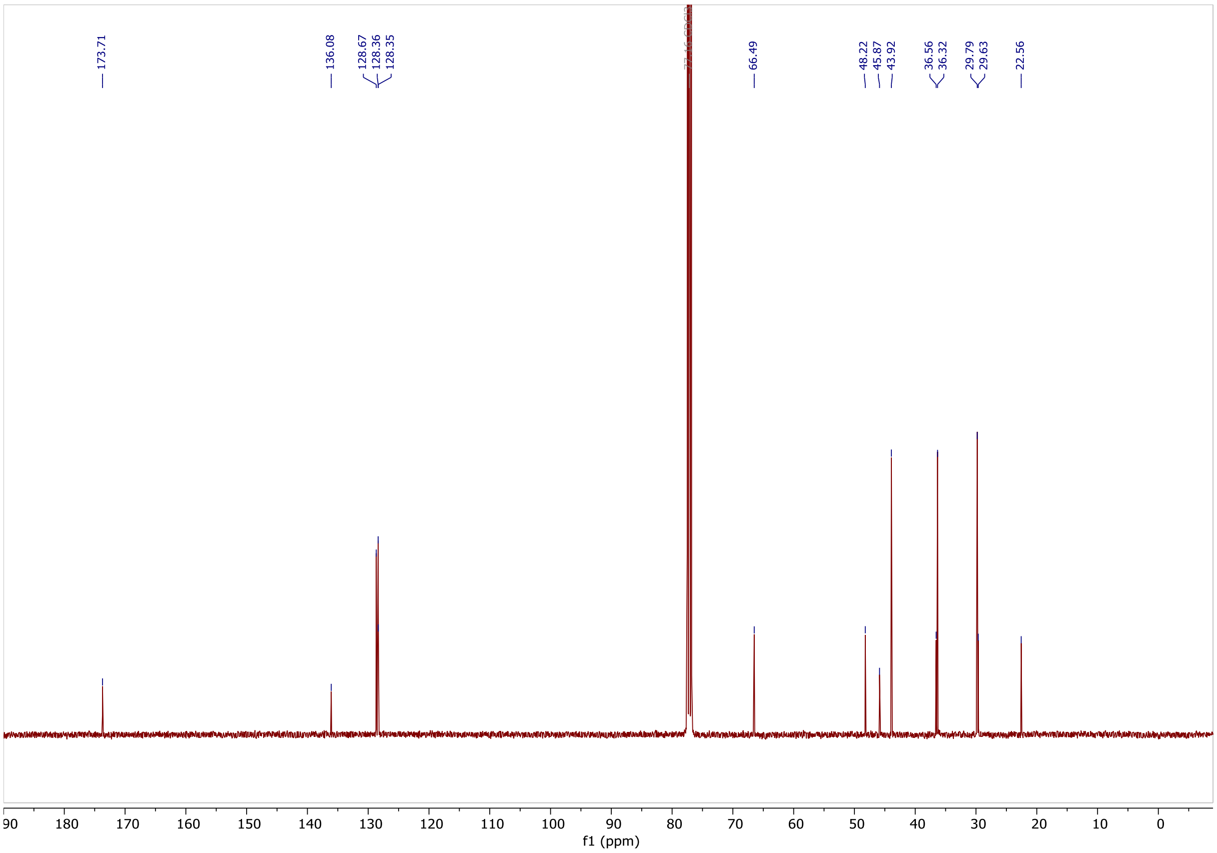

^{1}H-NMR (400 MHz, chloroform-*d*) (3'w)

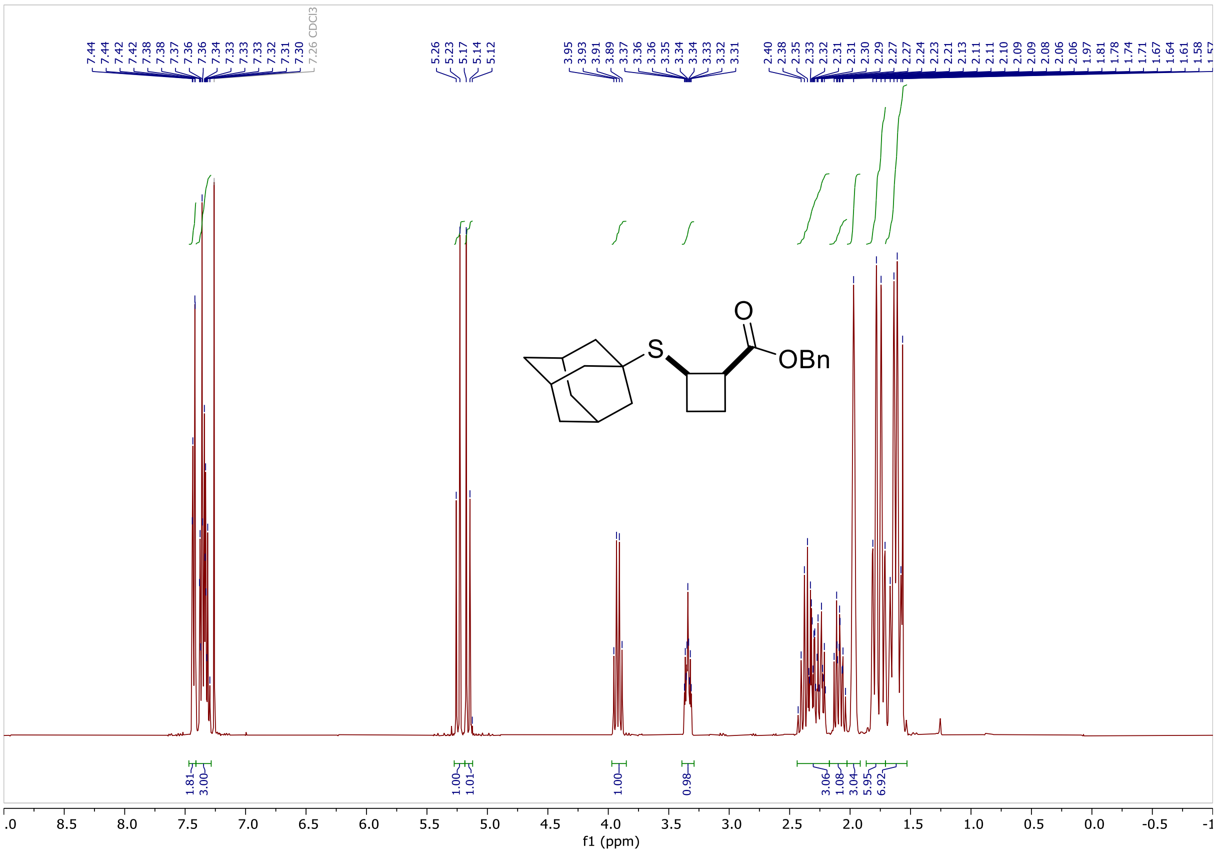

^{13}C-NMR (101 MHz, chloroform-*d*) (3'w)

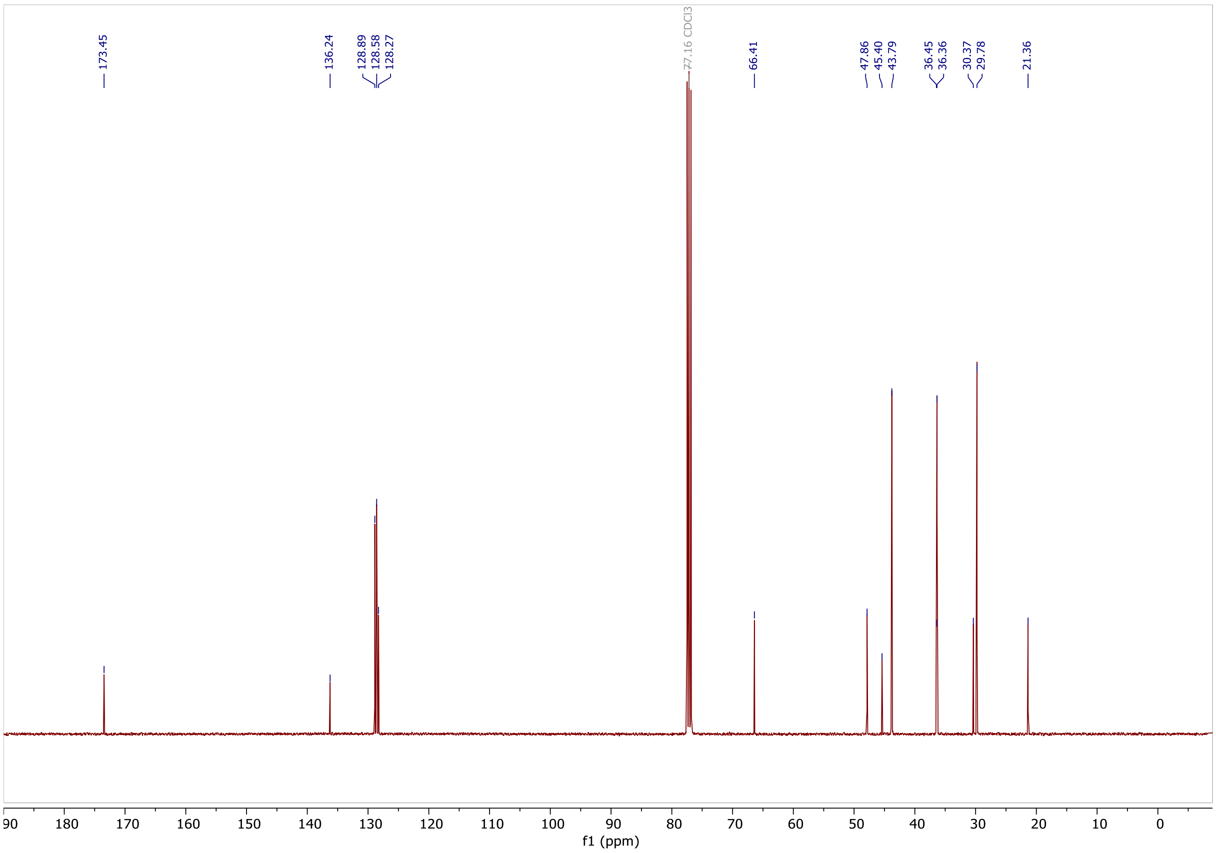

2D-NOESY (400 MHz, chloroform-*d*) (3’w)

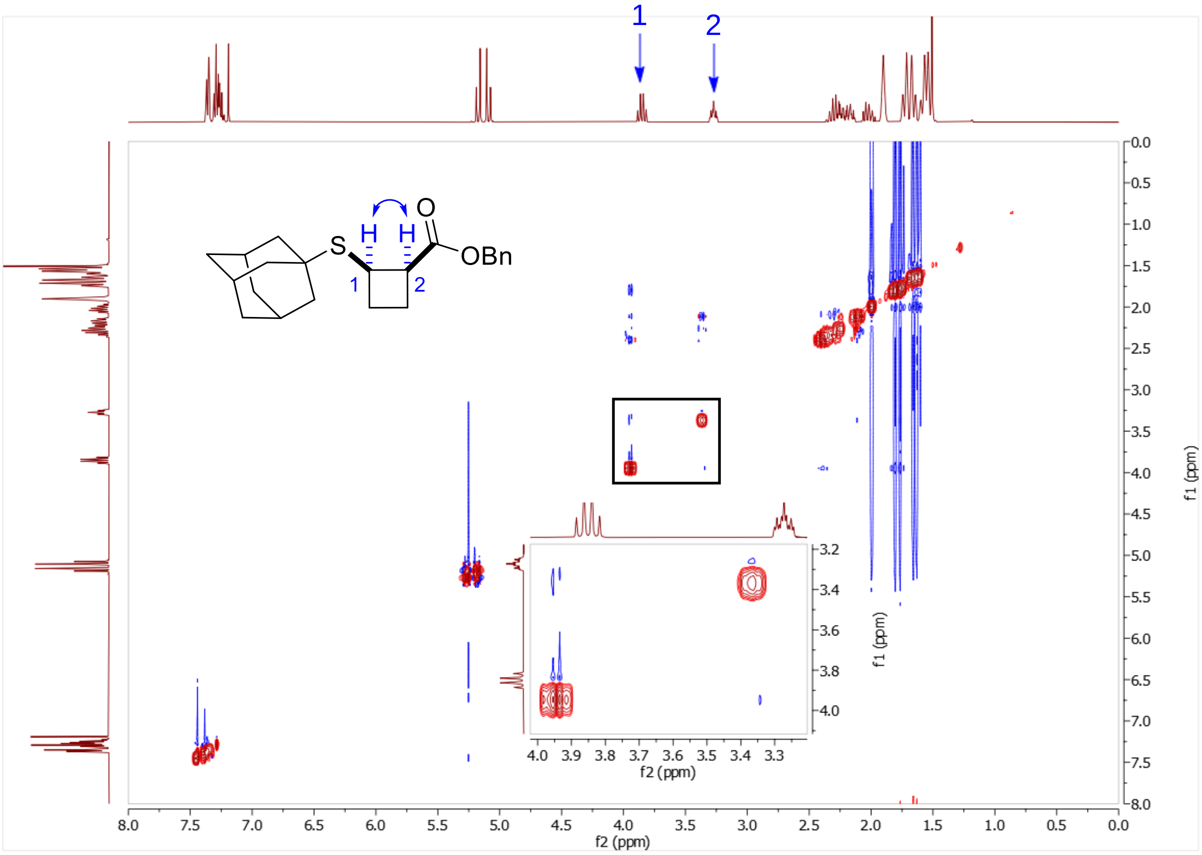

^{1}H-NMR (400 MHz, chloroform-*d*) (3x:3’x 65:35 mixture of diastereoisomers)

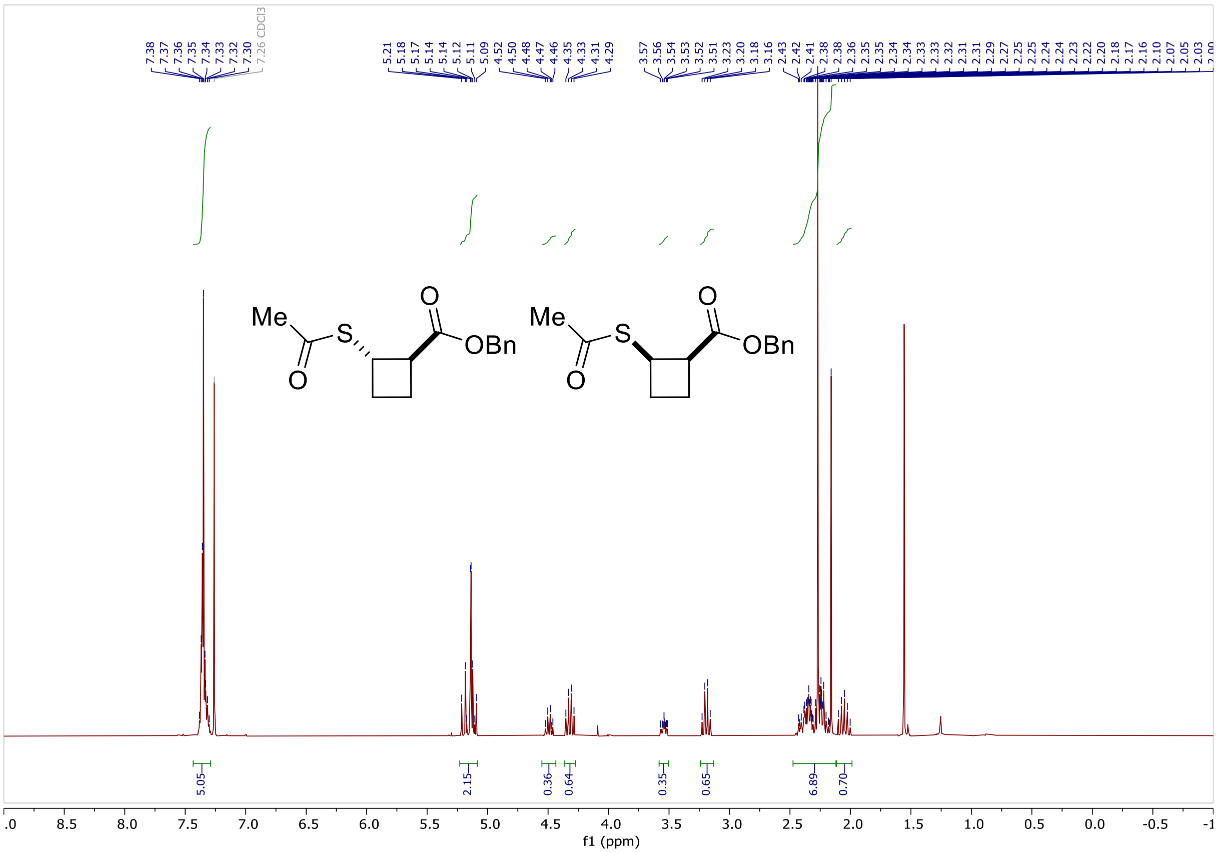

^{13}C-NMR (101 MHz, chloroform-*d*) (3x:3’x 65:35 mixture of diastereoisomers)

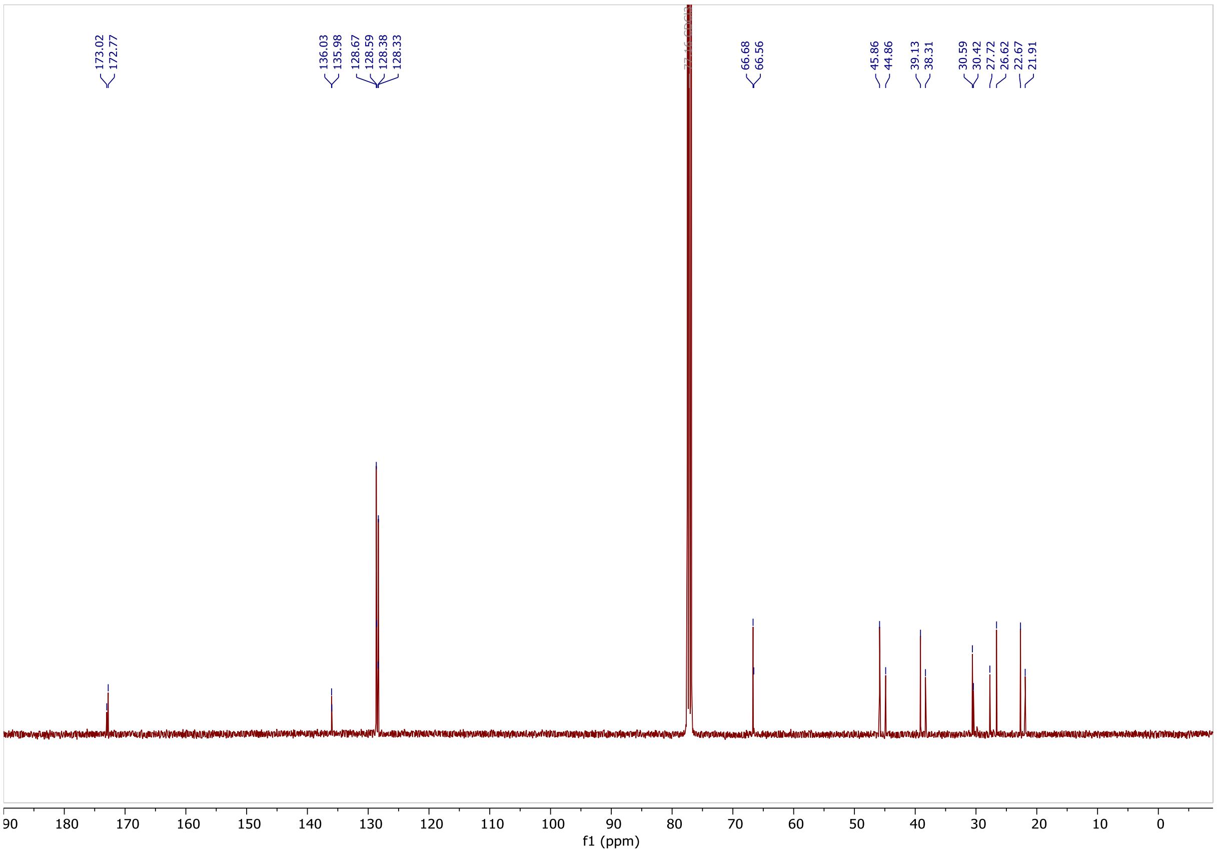

^{1}H-NMR (400 MHz, chloroform-*d*) (3y)

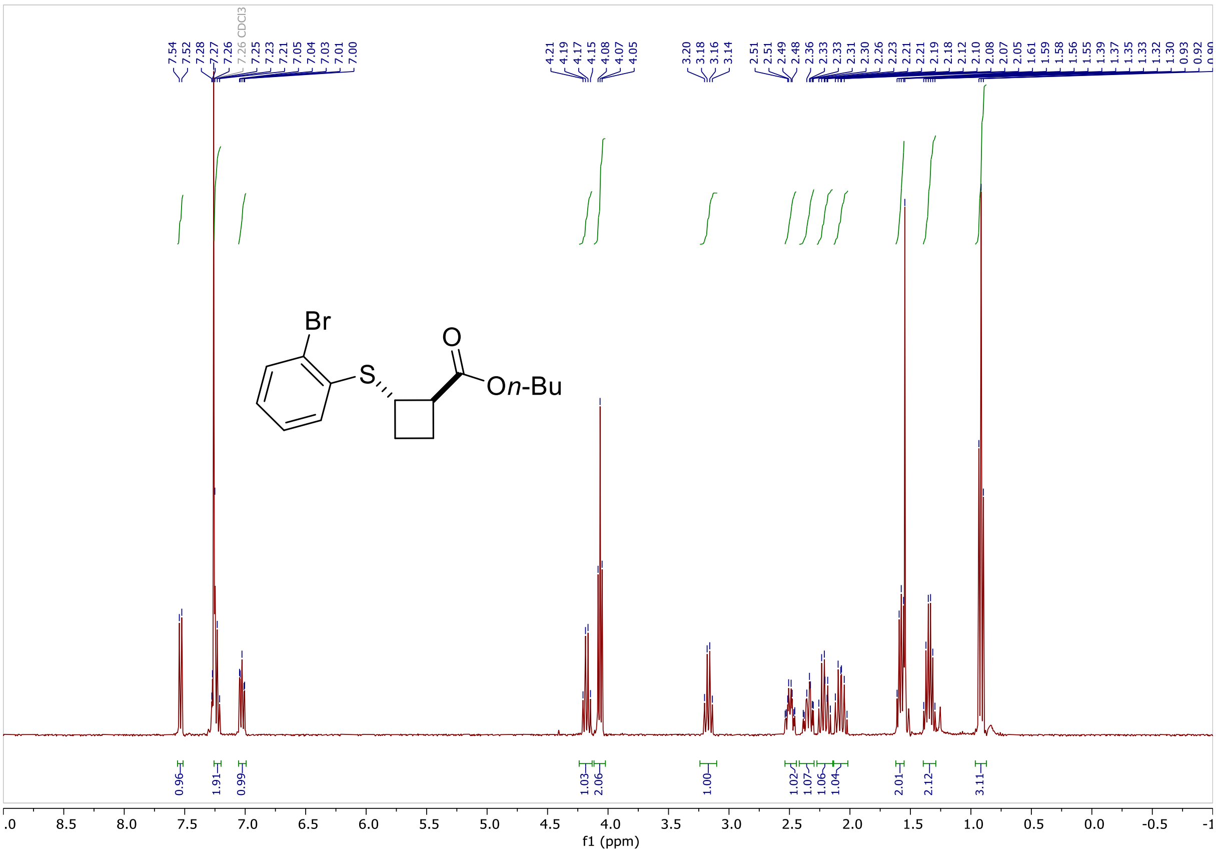

^{13}C-NMR (101 MHz, chloroform-*d*) (3y)

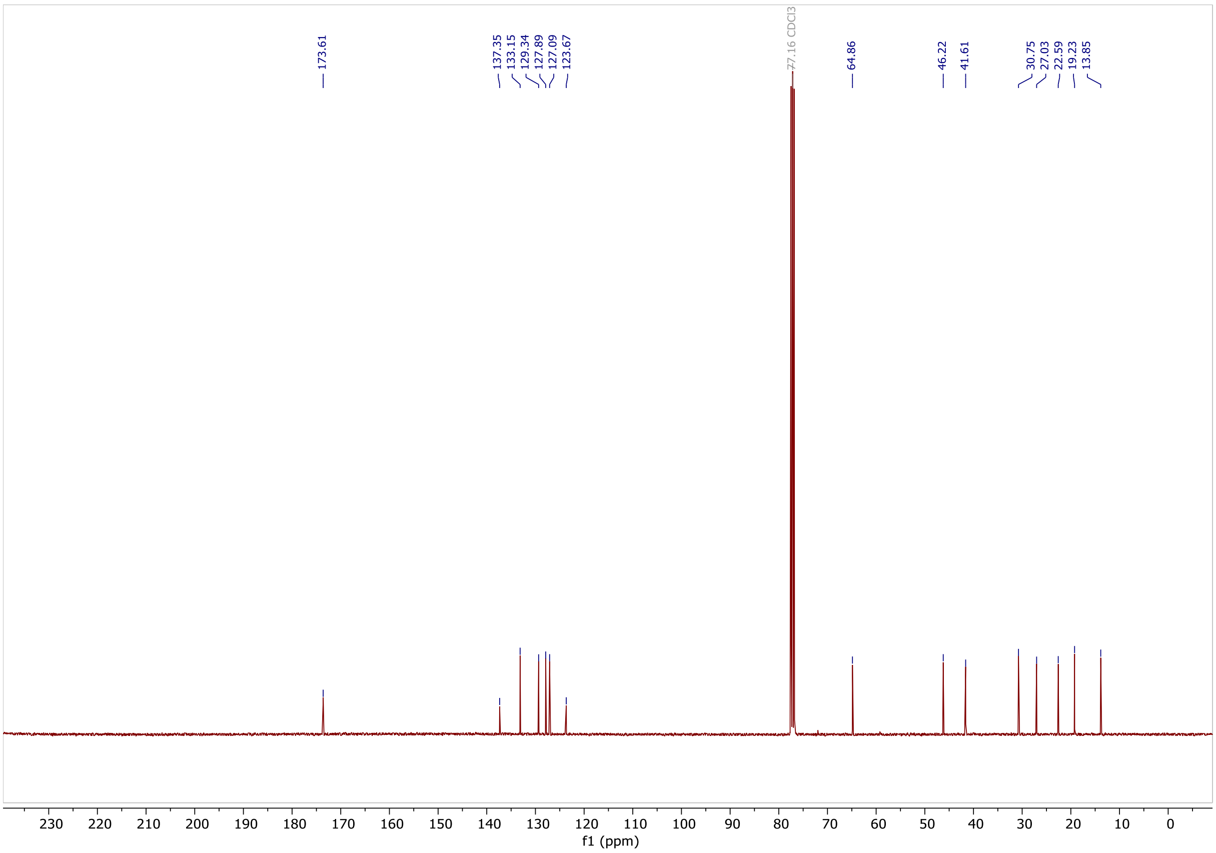

^{1}H-NMR (400 MHz, chloroform-*d*) (3z)

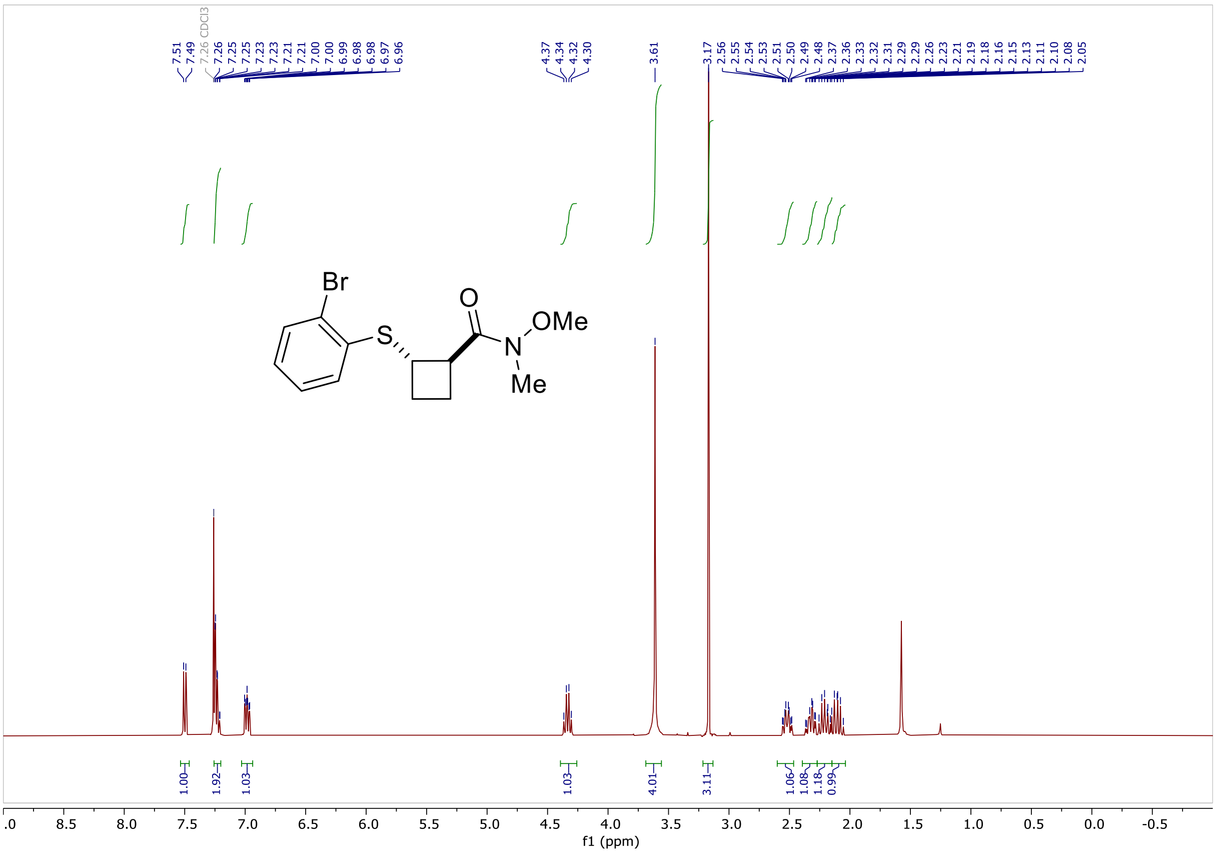

^{13}C-NMR (101 MHz, chloroform-*d*) (3z)

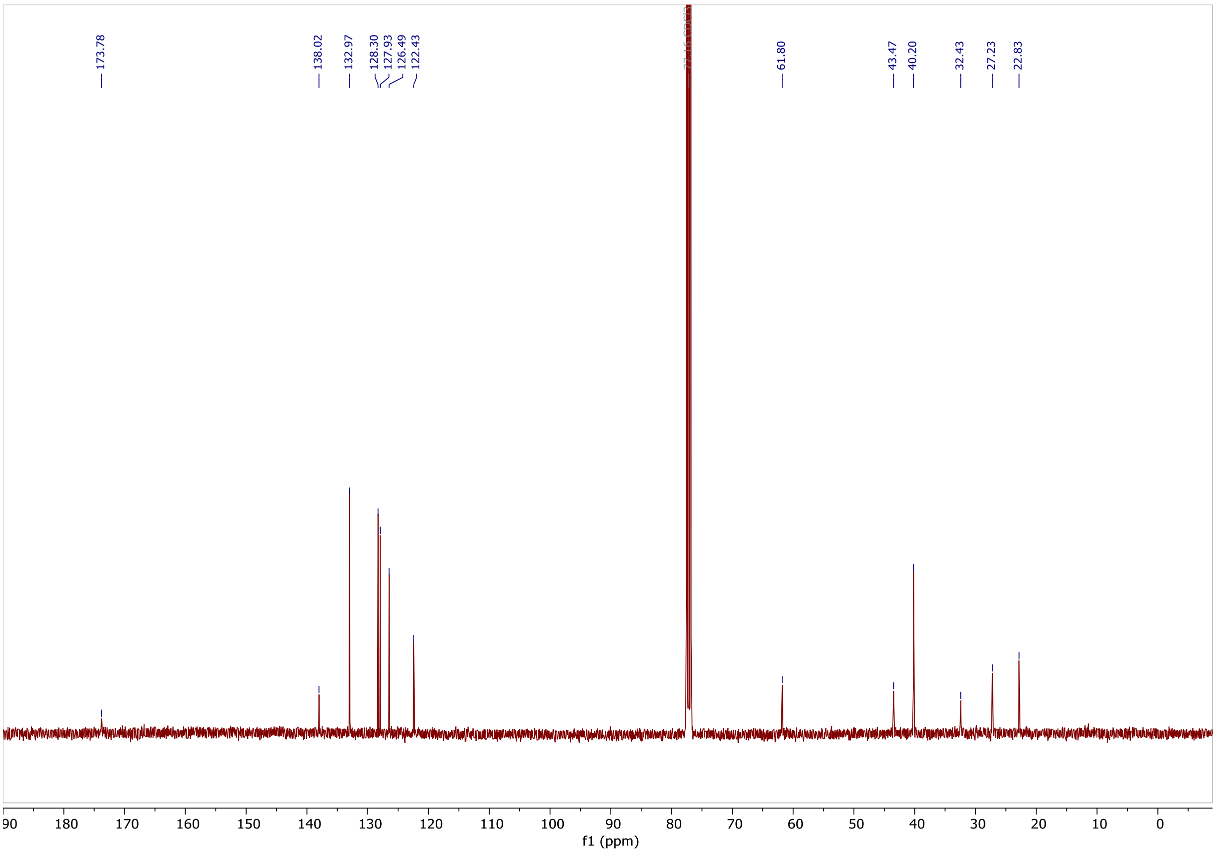

^{1}H-NMR (400 MHz, chloroform-*d*) (3’z)

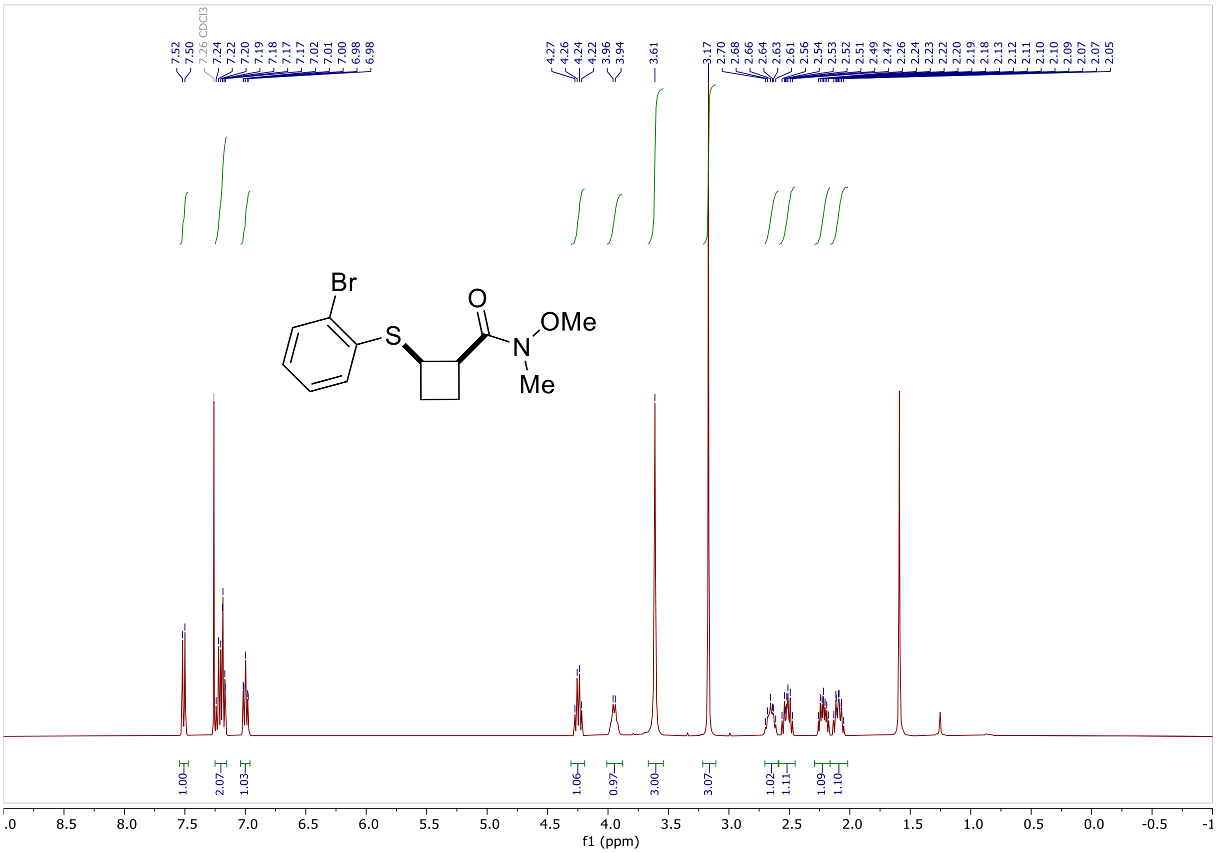

^{13}C-NMR (101 MHz, chloroform-*d*) (3’z)

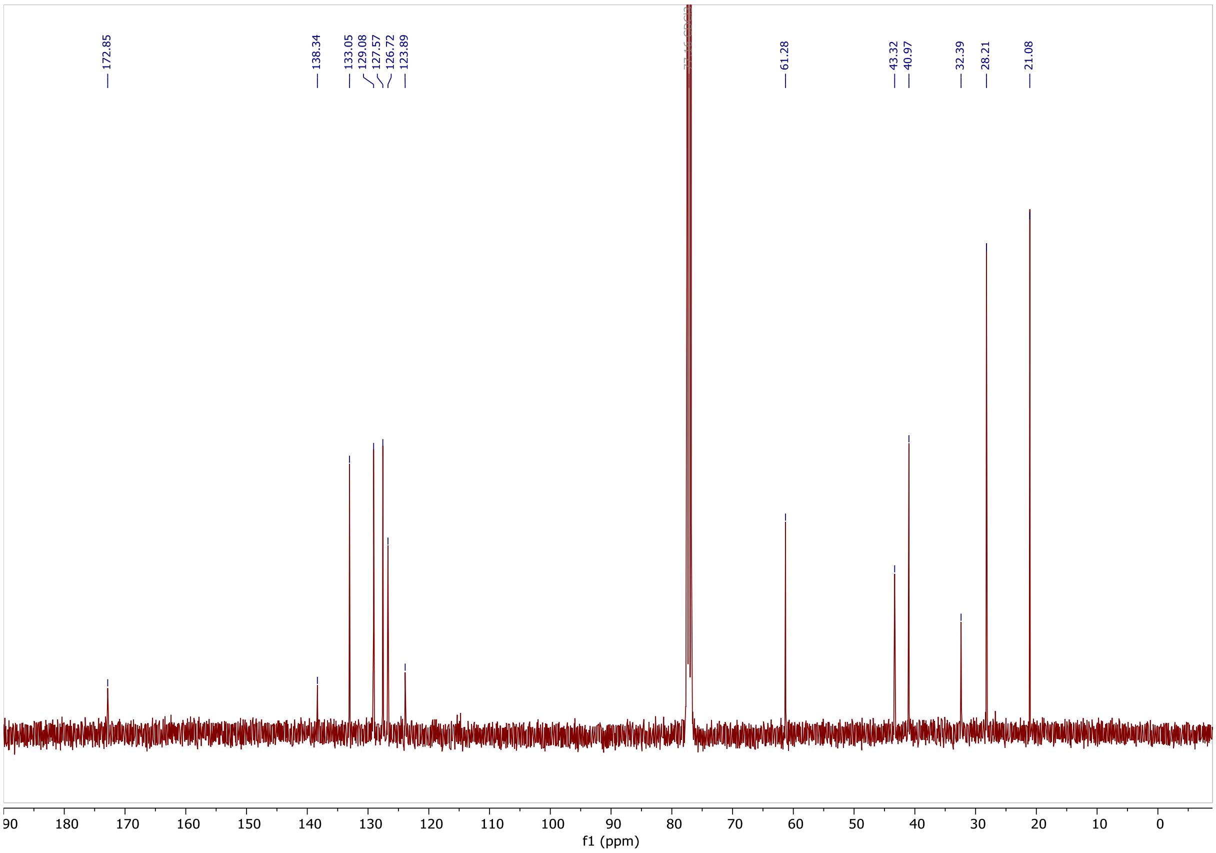

2D-NOESY (400 MHz, chloroform-*d*) (3’z)

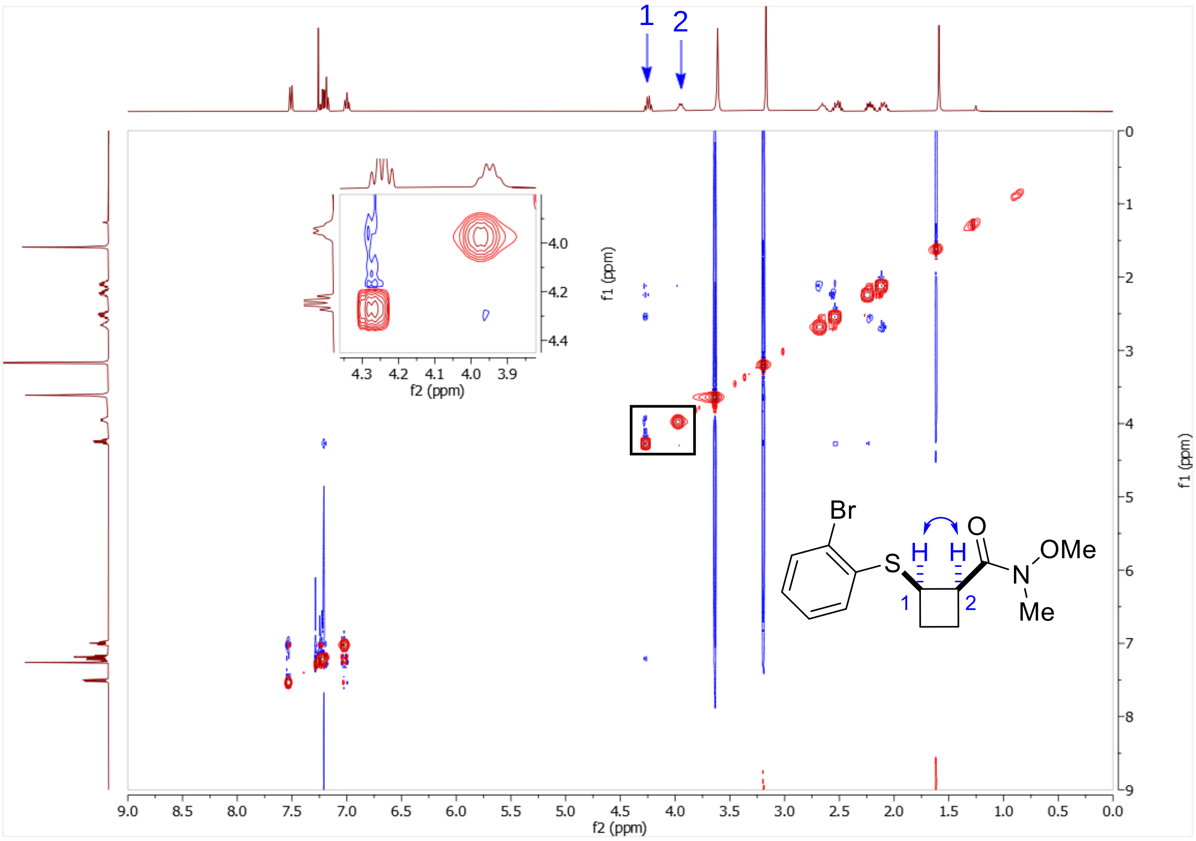

^{1}H-NMR (400 MHz, chloroform-*d*) (3aa)

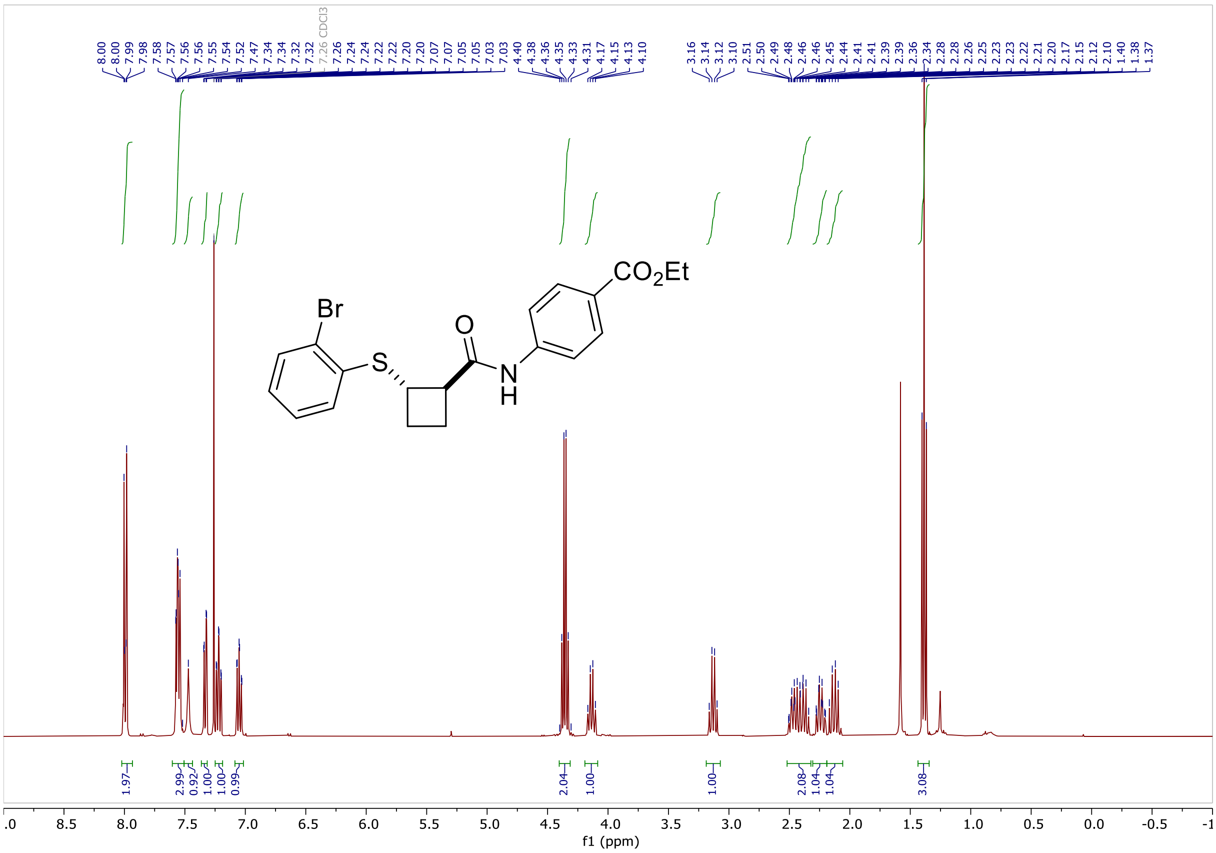

^{13}C-NMR (101 MHz, chloroform-*d*) (3aa)

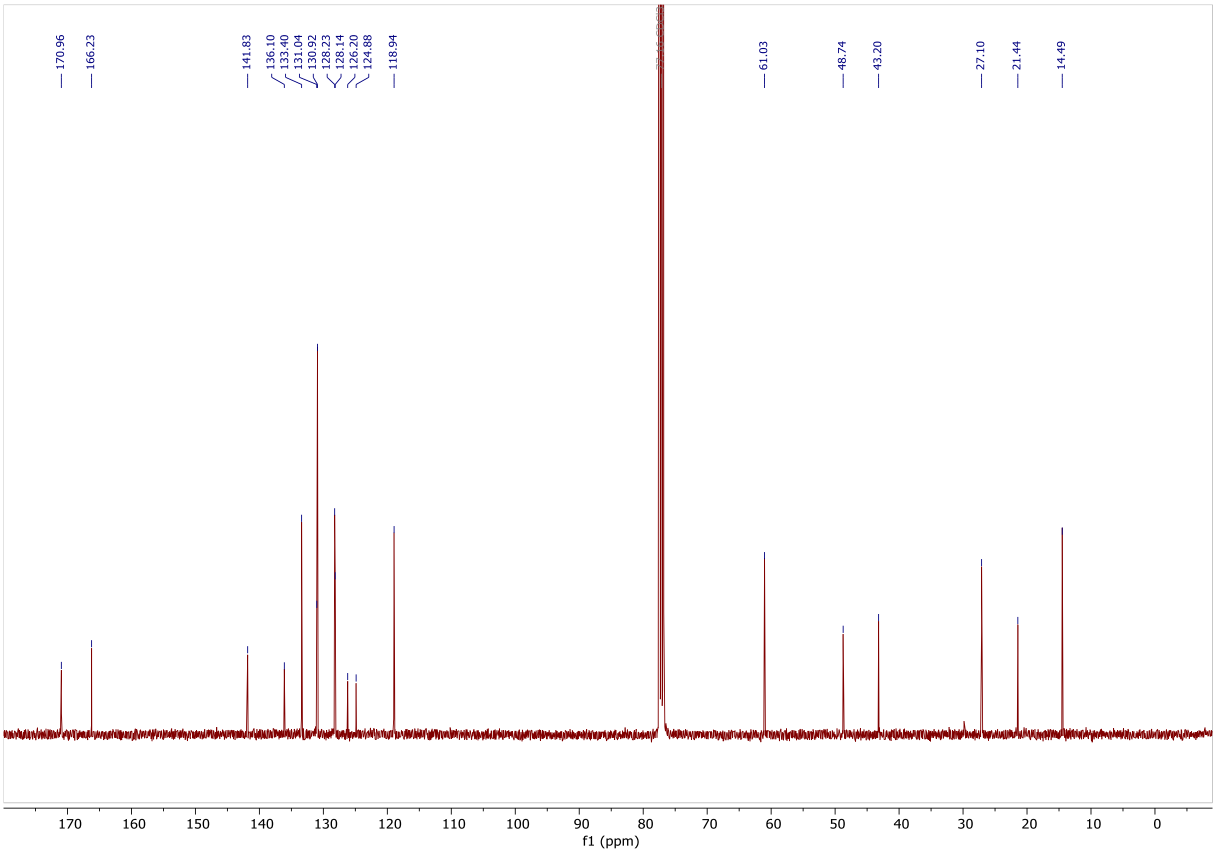

^{1}H-NMR (400 MHz, chloroform-*d*) (4a)

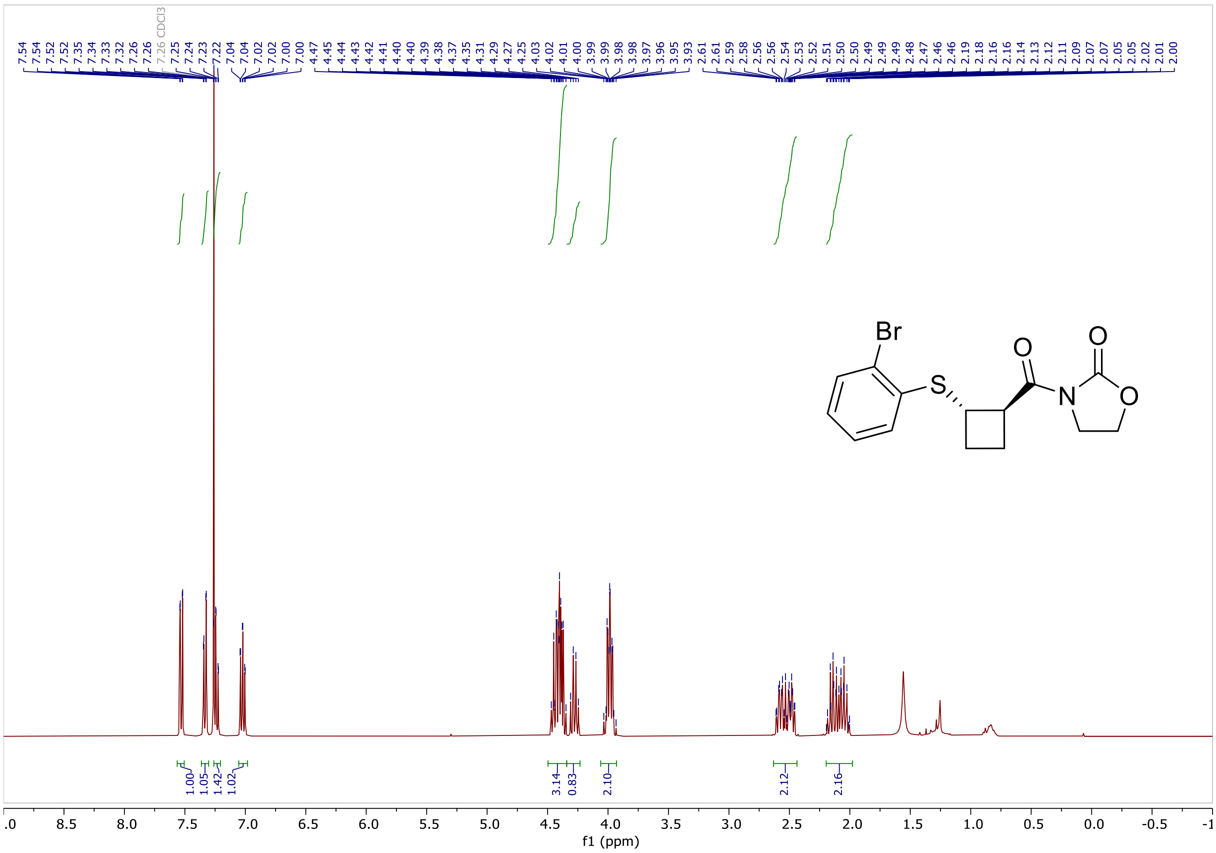

^{13}C-NMR (101 MHz, chloroform-*d*) (4a)

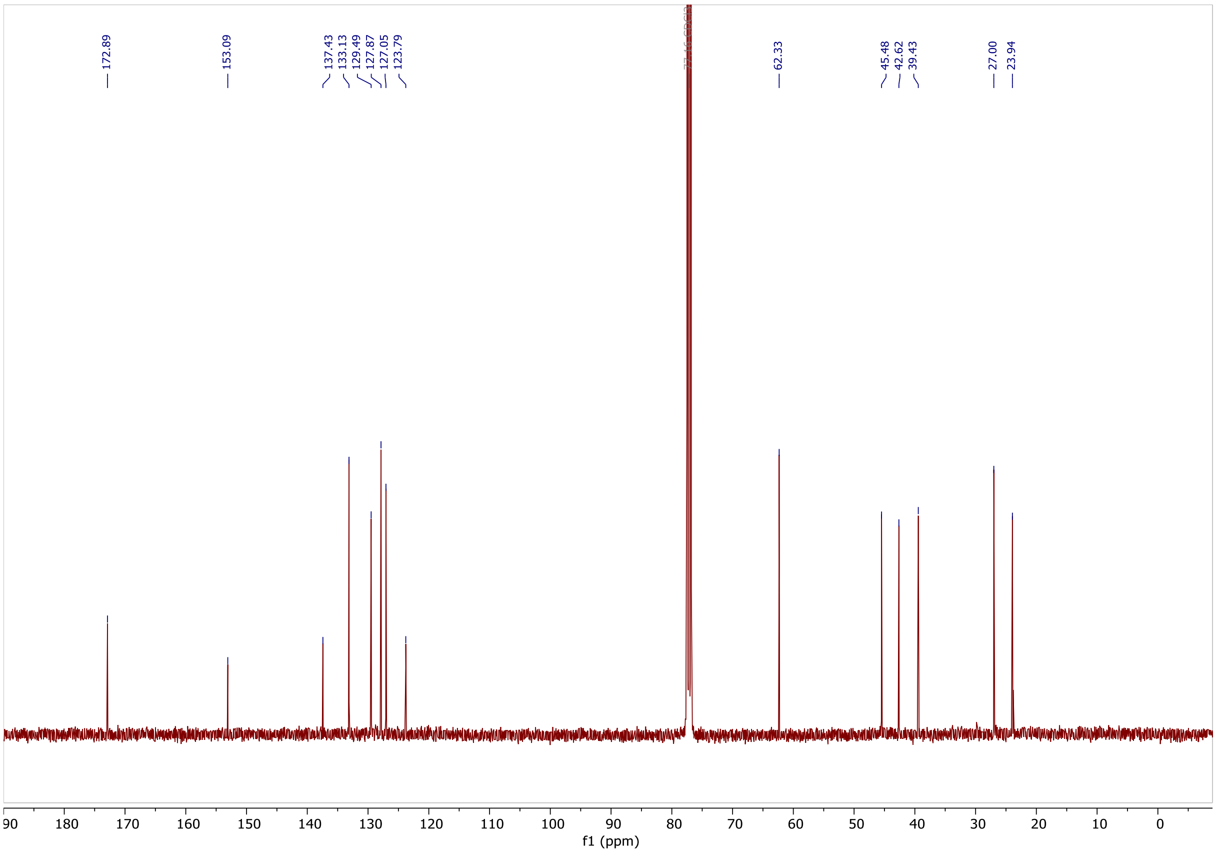

^{1}H-NMR (400 MHz, chloroform-*d*) (4b)

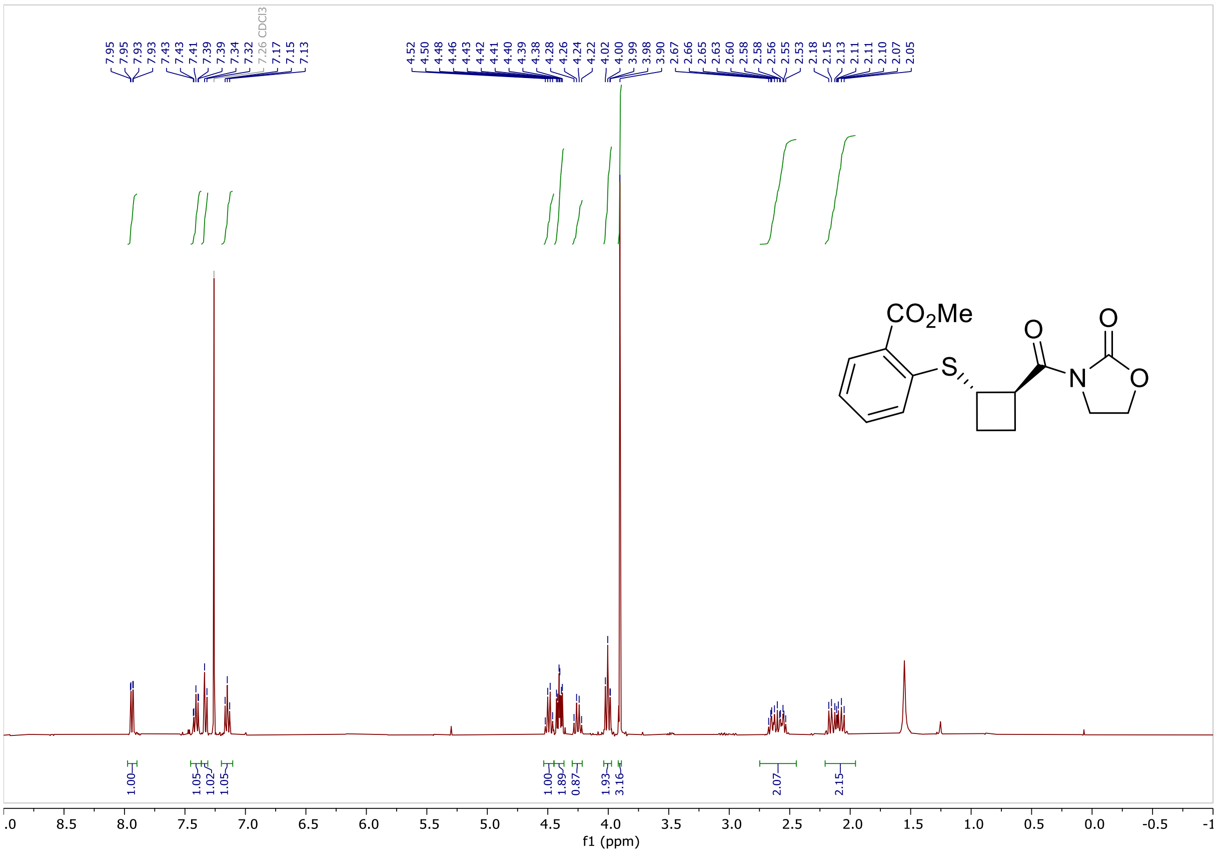

^{13}C-NMR (101 MHz, chloroform-*d*) (4b)

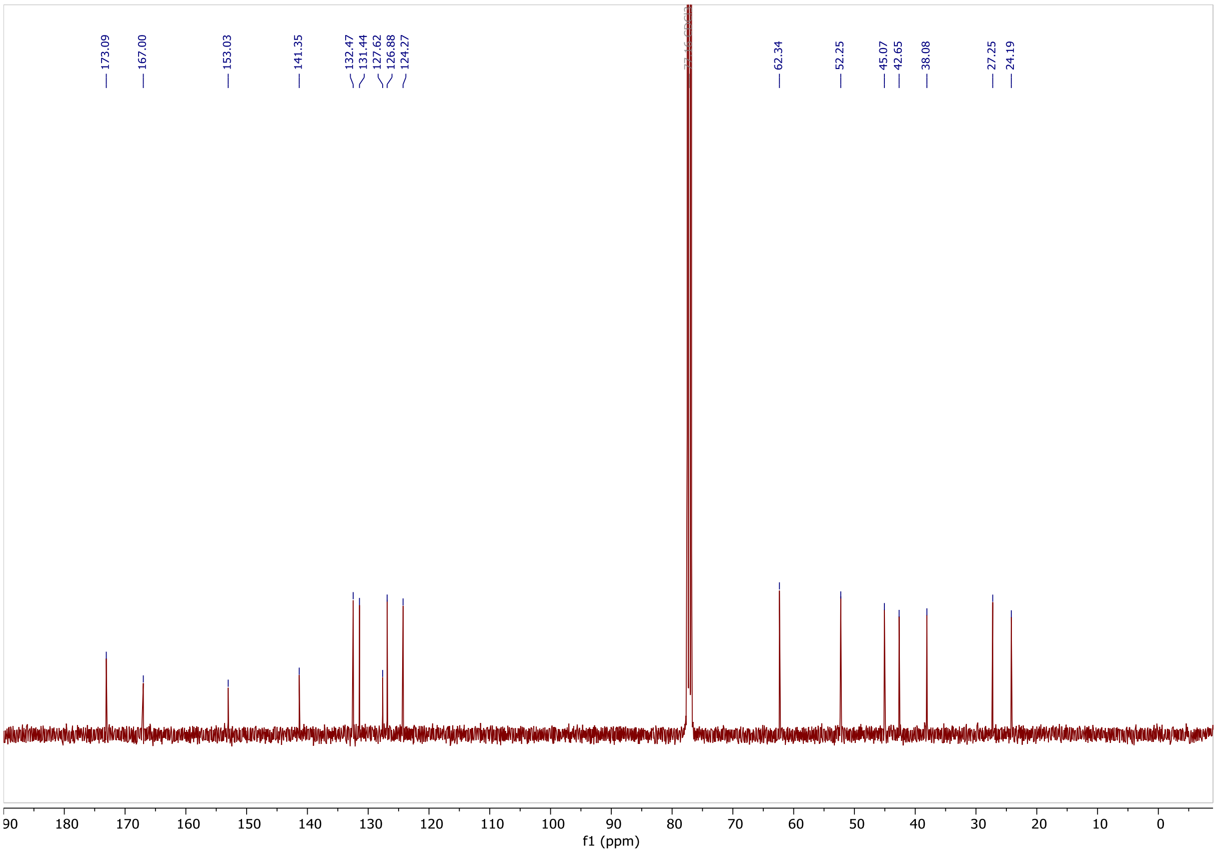

^{1}H-NMR (400 MHz, chloroform-*d*) (4c)

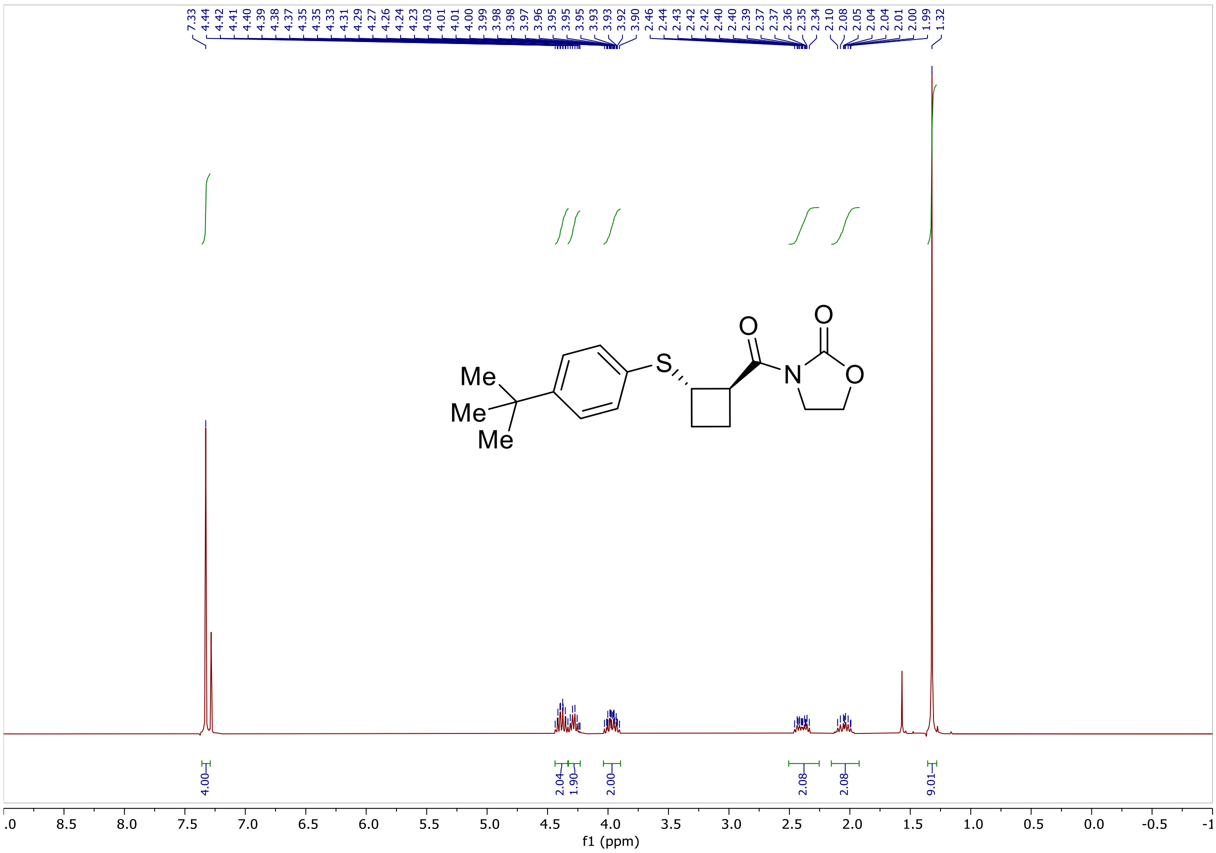

^{13}C-NMR (101 MHz, chloroform-*d*) (4c)

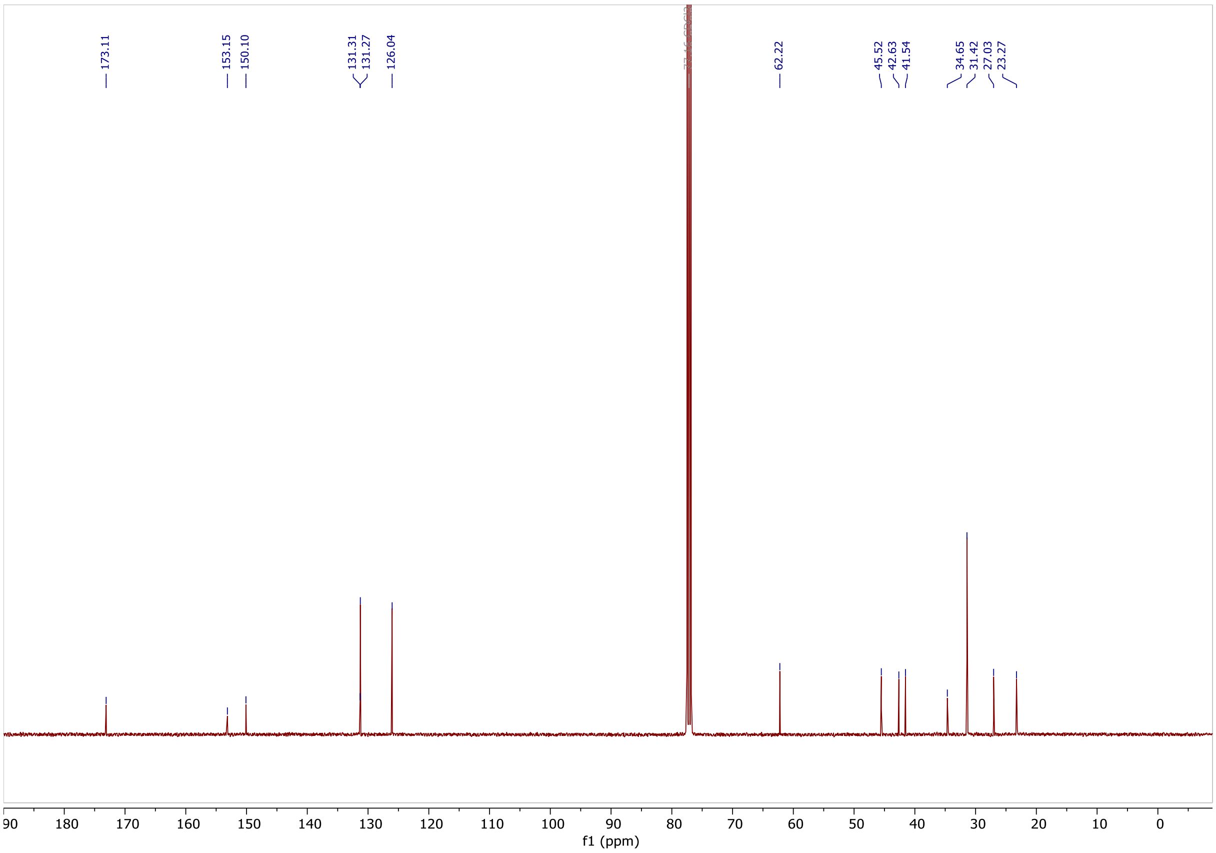

^{1}H-NMR (400 MHz, chloroform-*d*) (4d)

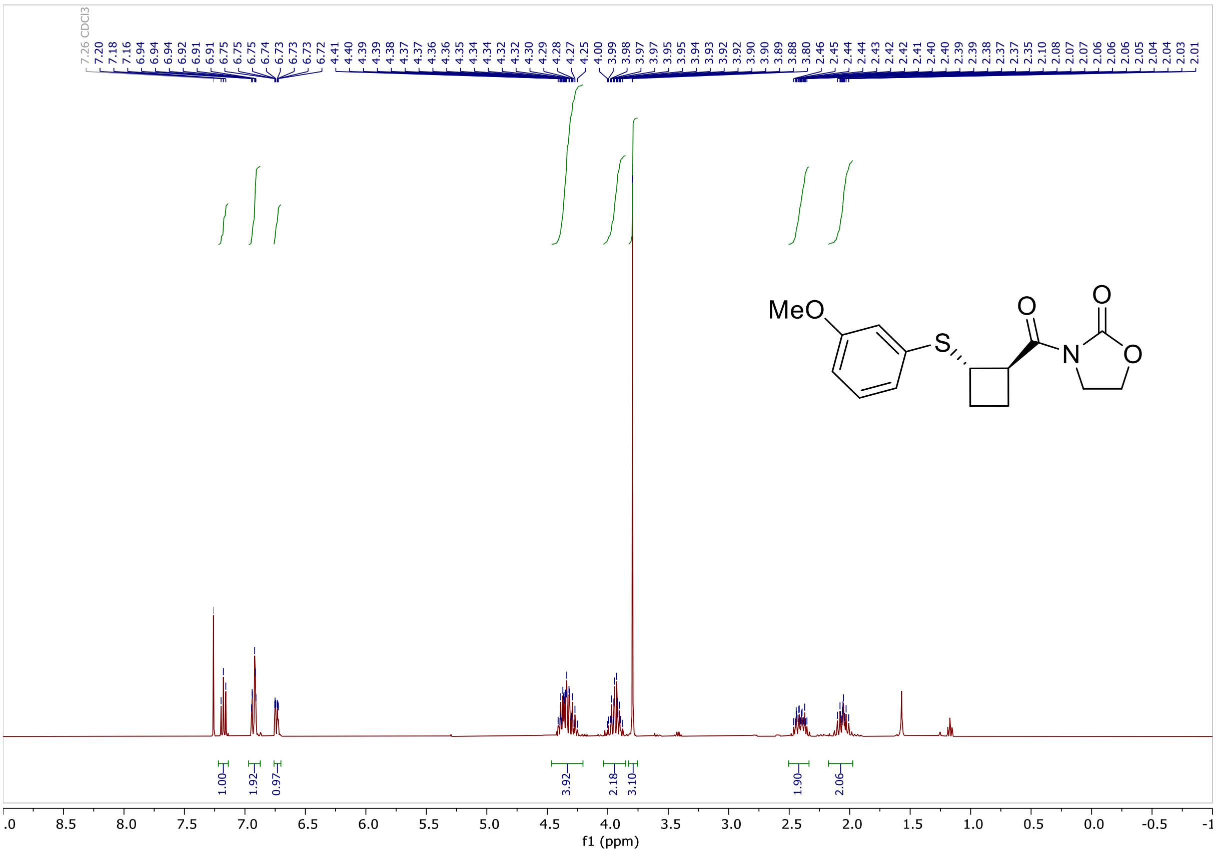

^{13}C-NMR (101 MHz, chloroform-*d*) (4d)

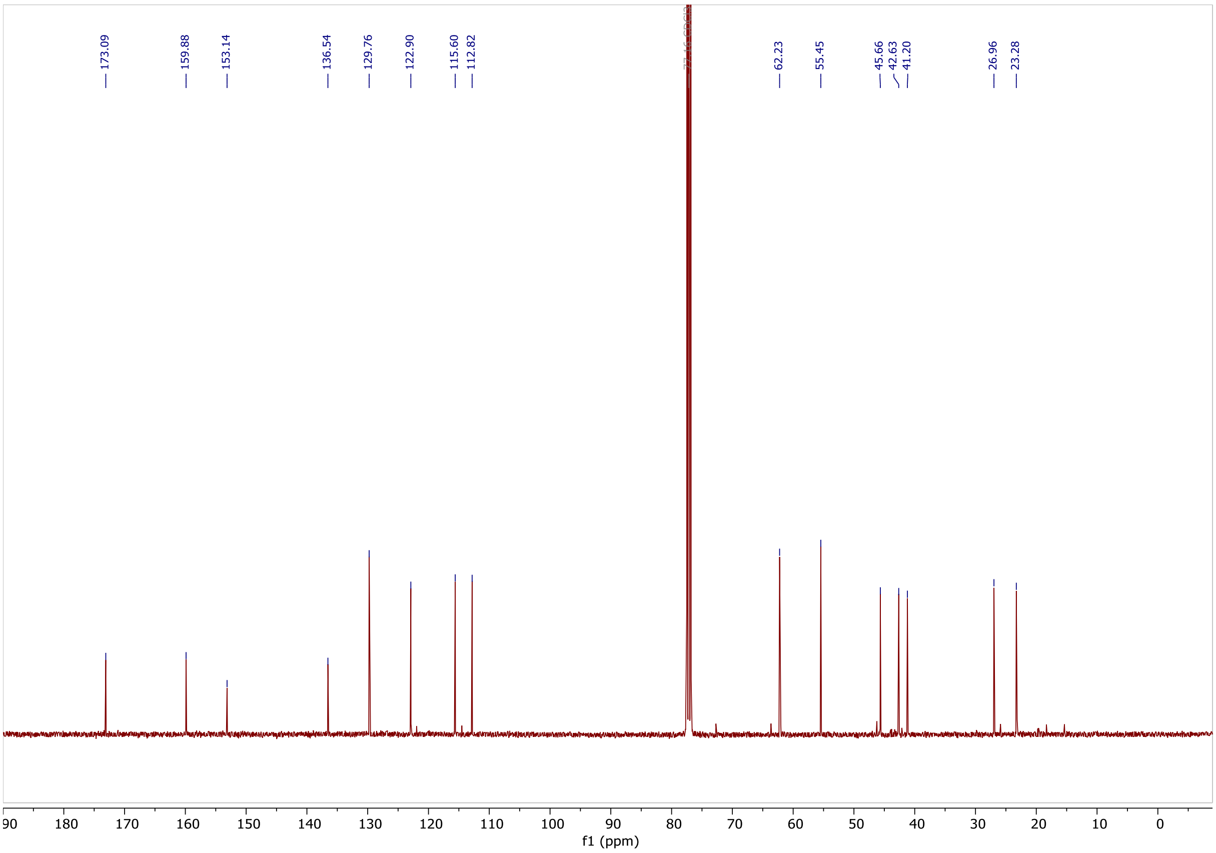

^{1}H-NMR (400 MHz, chloroform-*d*) (4e)

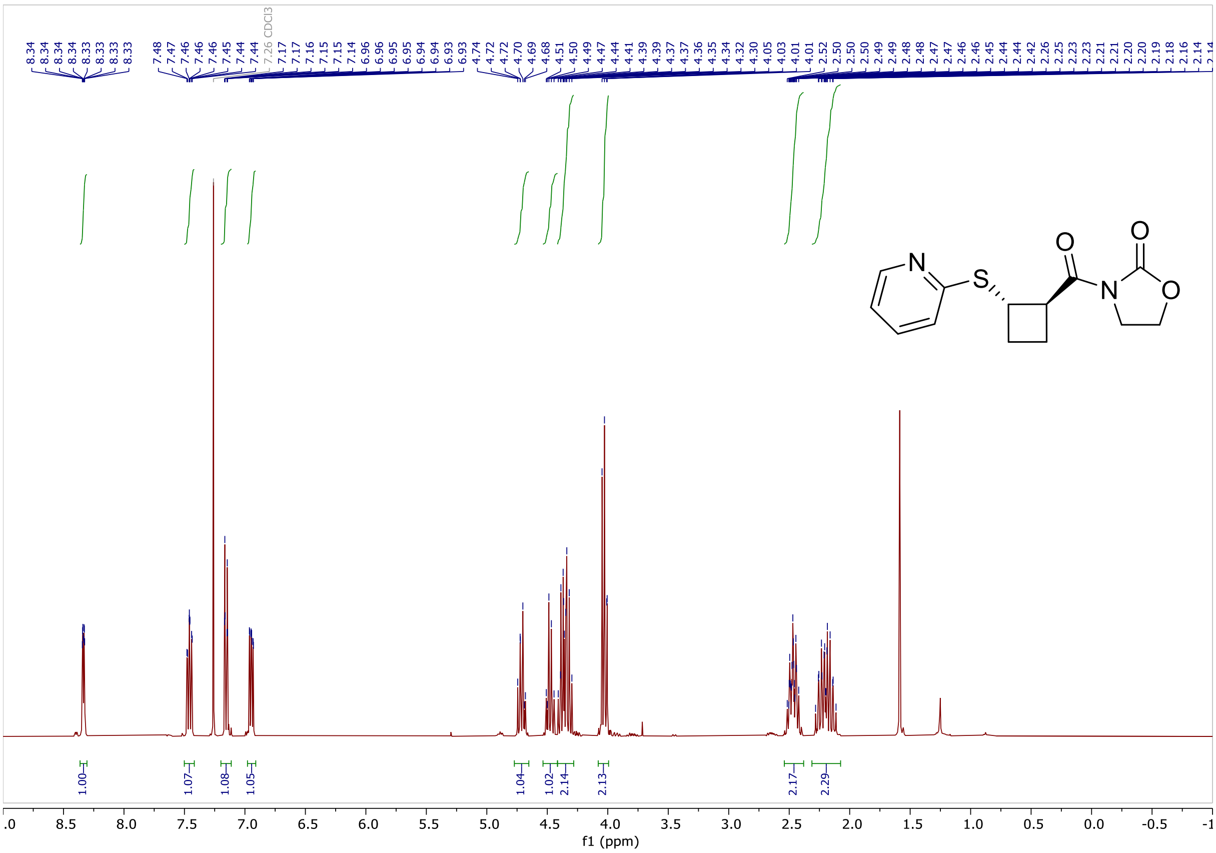

^{13}C-NMR (101 MHz, chloroform-*d*) (4e)

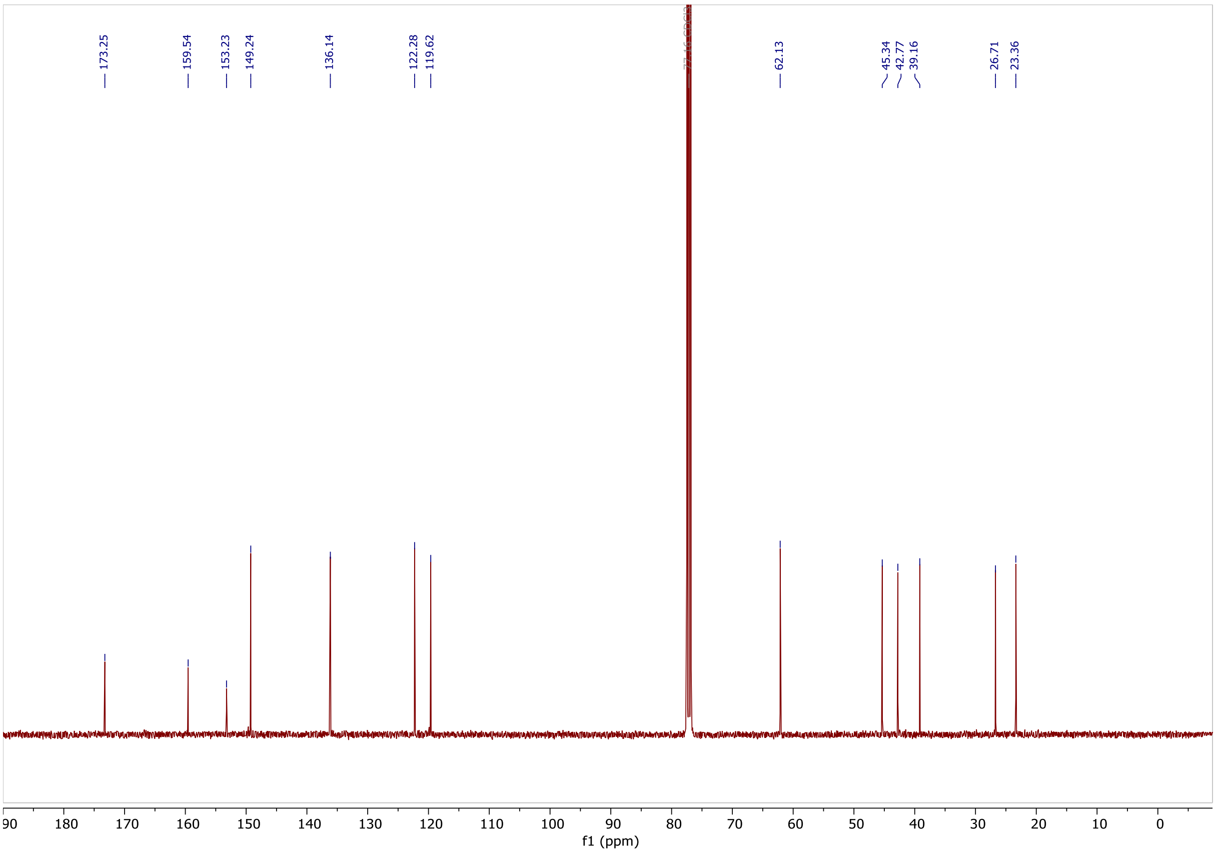

^{1}H-NMR (400 MHz, chloroform-*d*) (4f)

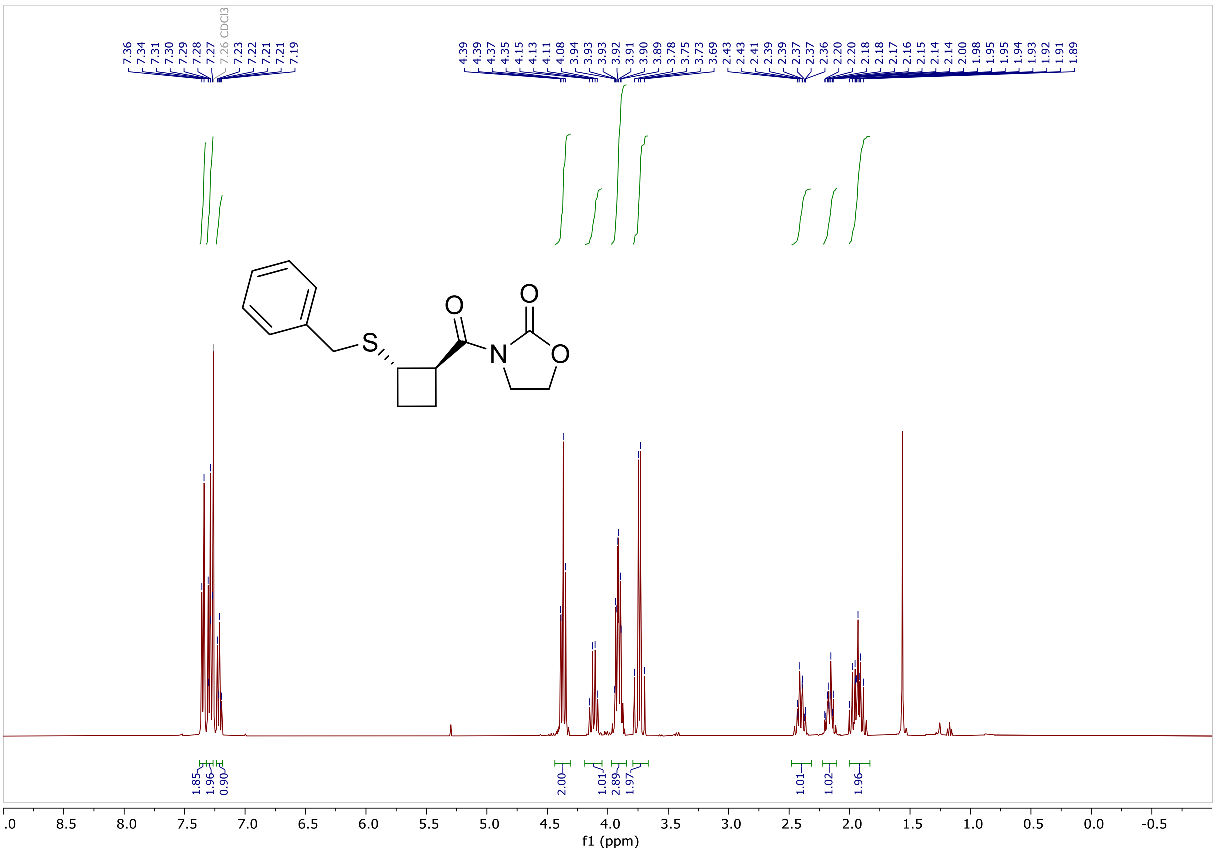

^{13}C-NMR (101 MHz, chloroform-*d*) (4f)

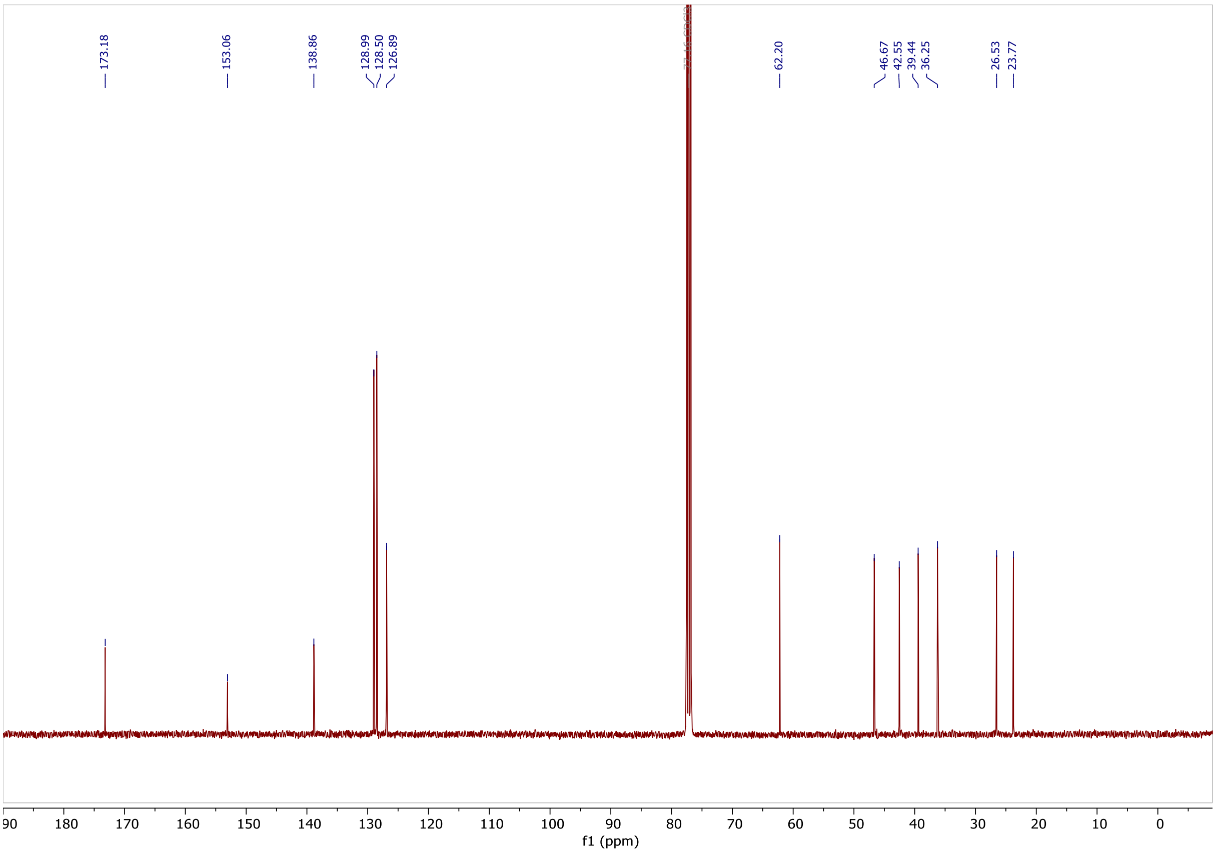

^{1}H-NMR (400 MHz, chloroform-*d*) (4g)

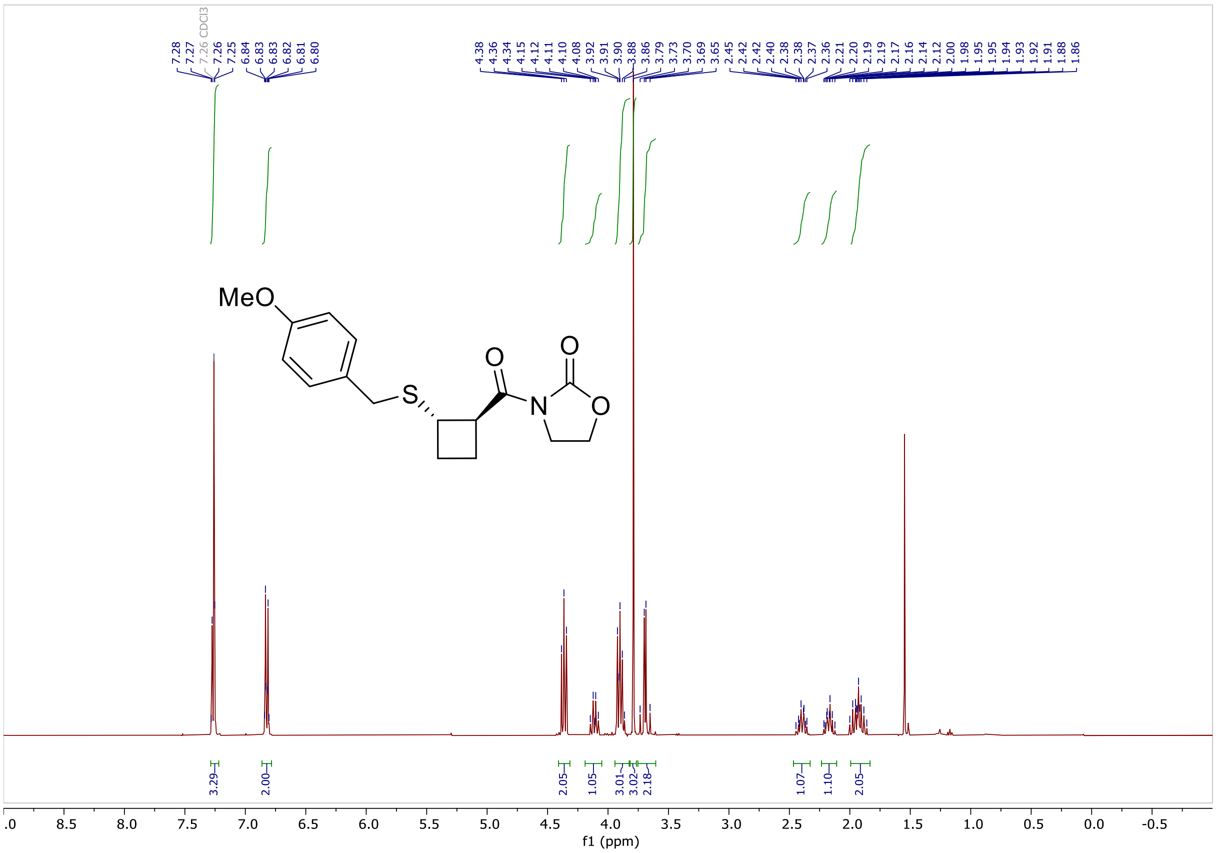

^{13}C-NMR (101 MHz, chloroform-*d*) (4g)

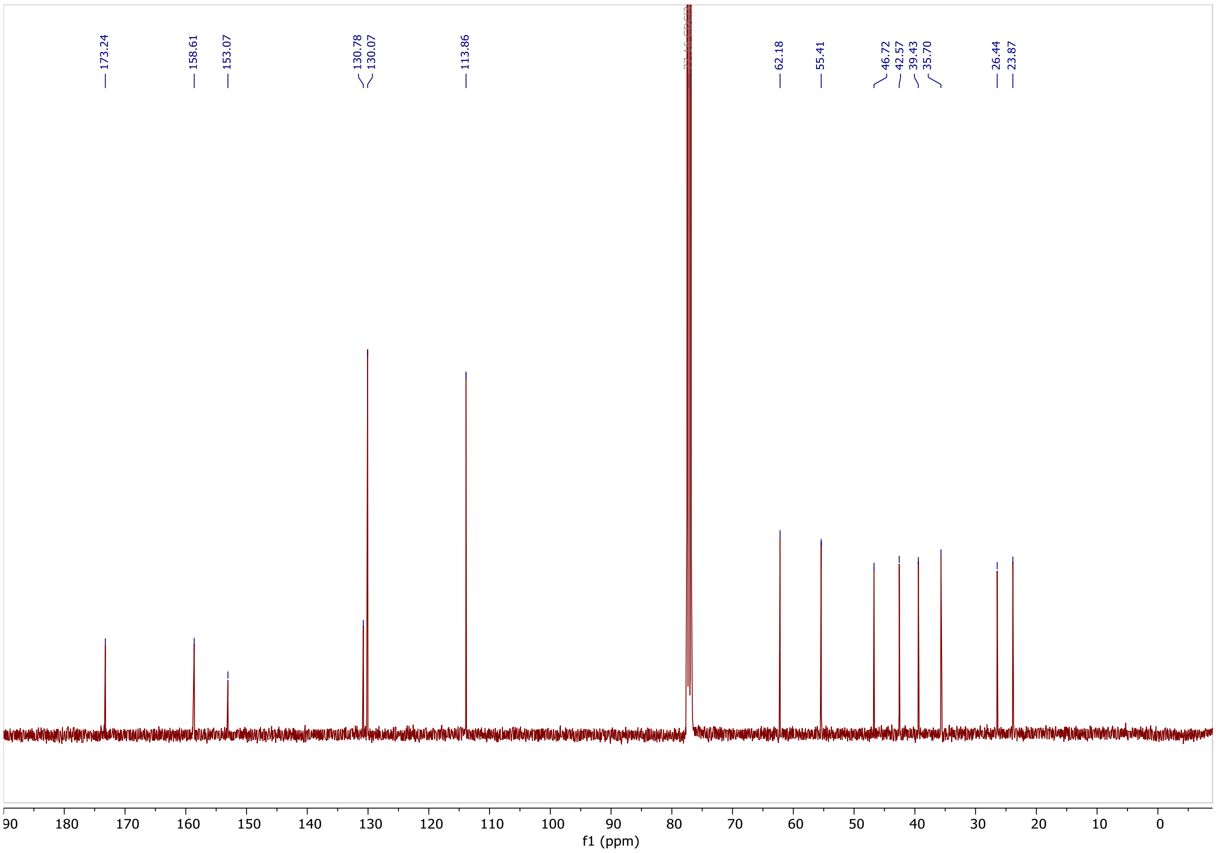

^{1}H-NMR (400 MHz, chloroform-*d*) (4h)

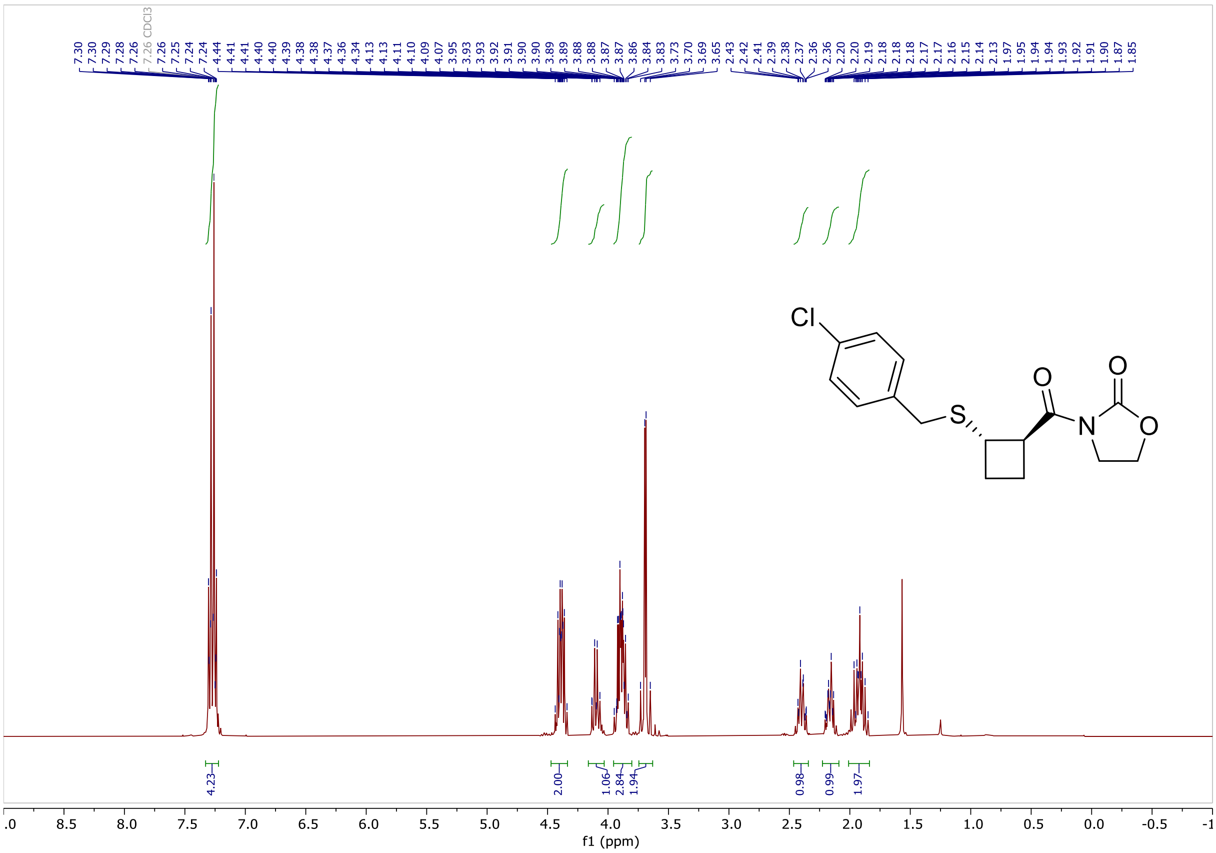

^{13}C-NMR (101 MHz, chloroform-*d*) (4h)

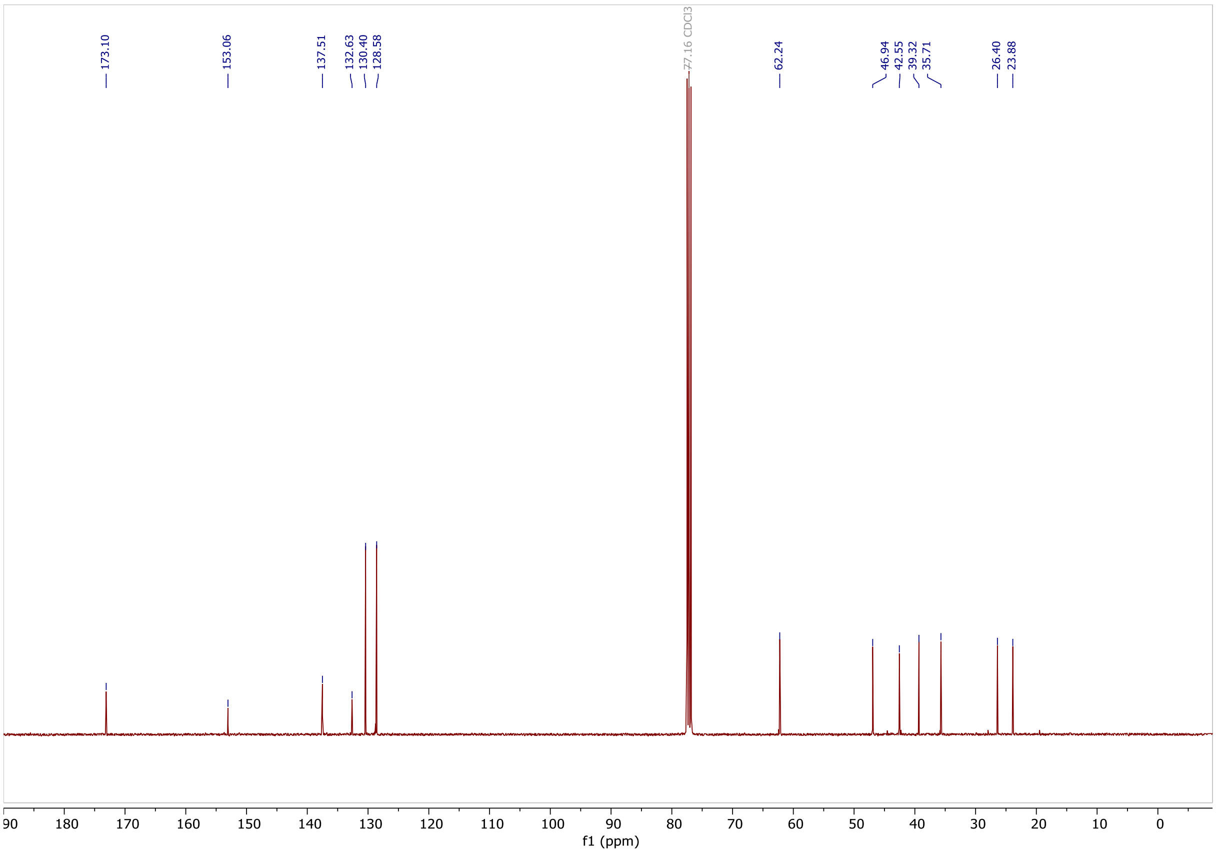

^{1}H-NMR (400 MHz, chloroform-*d*) (4i)

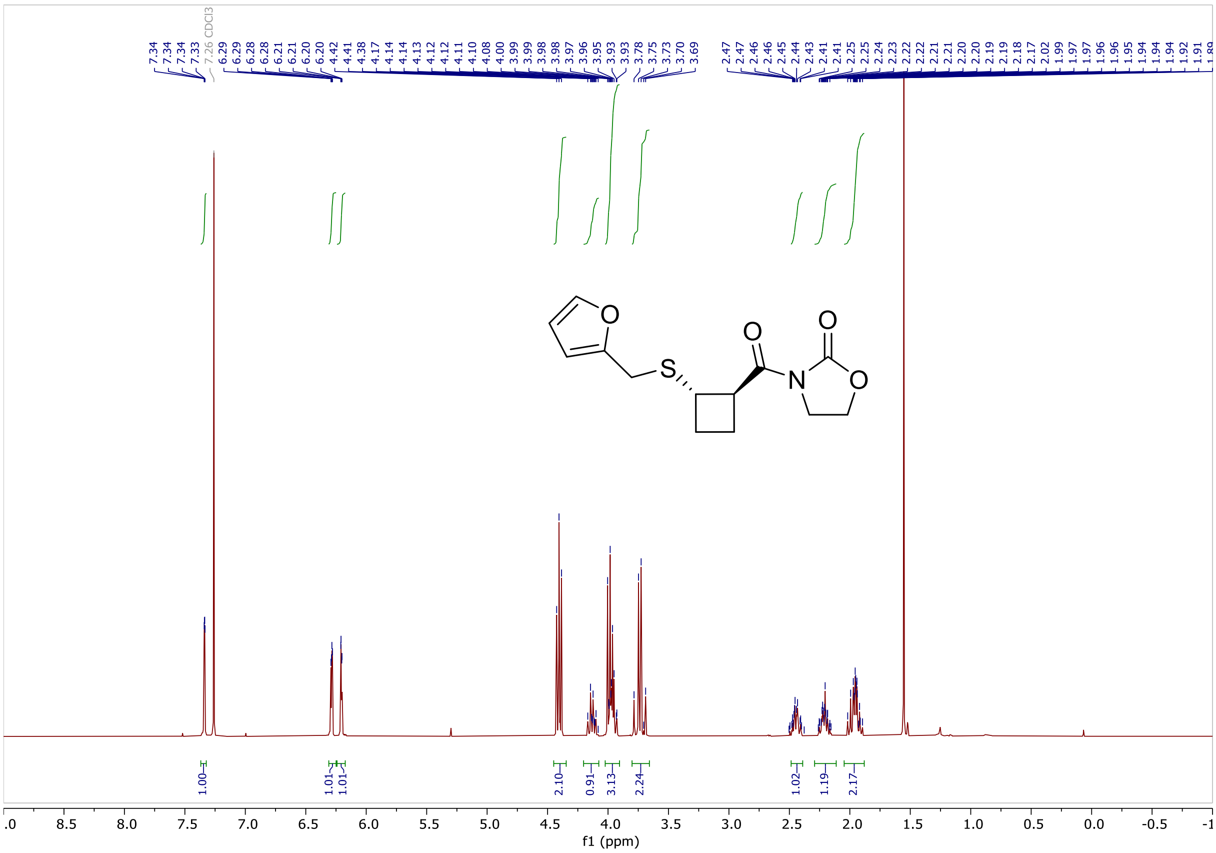

^{13}C-NMR (101 MHz, chloroform-*d*) (4i)

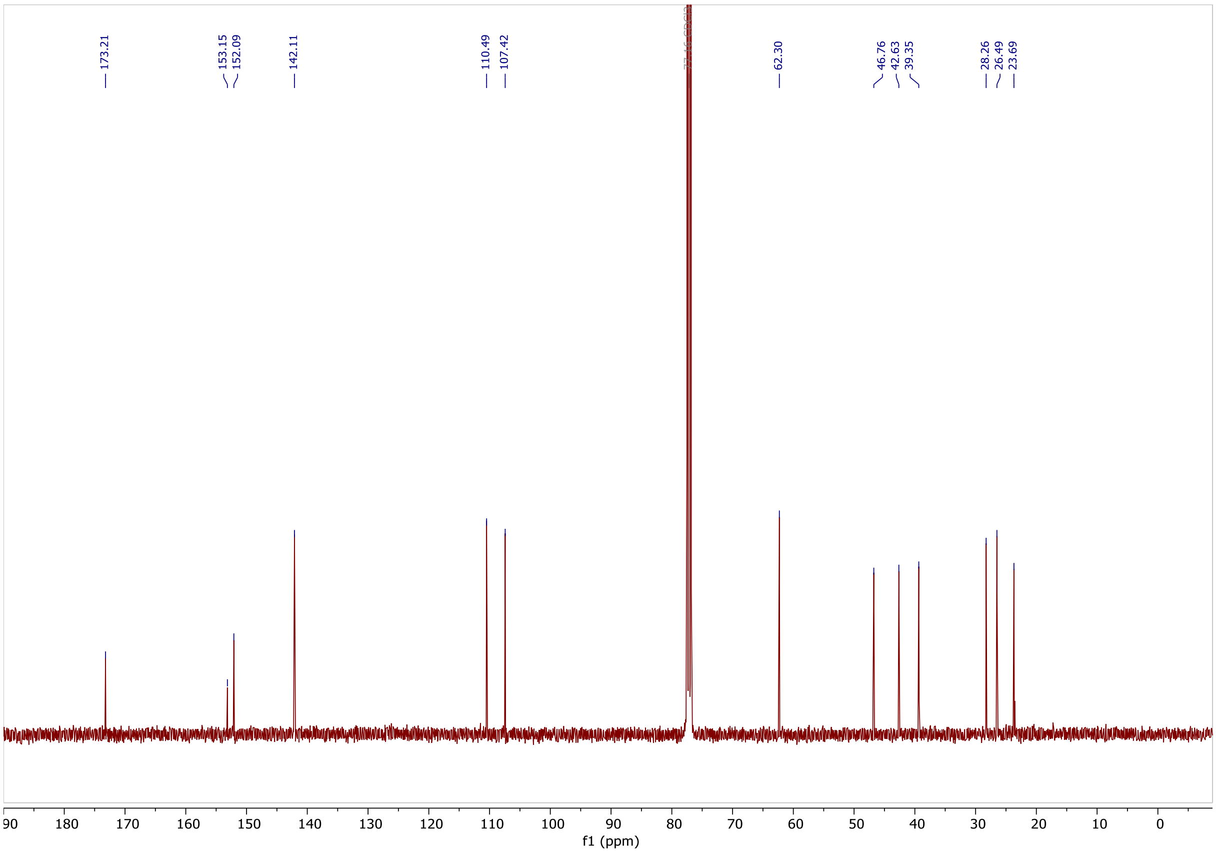

^{1}H-NMR (400 MHz, chloroform-*d*) (4j)

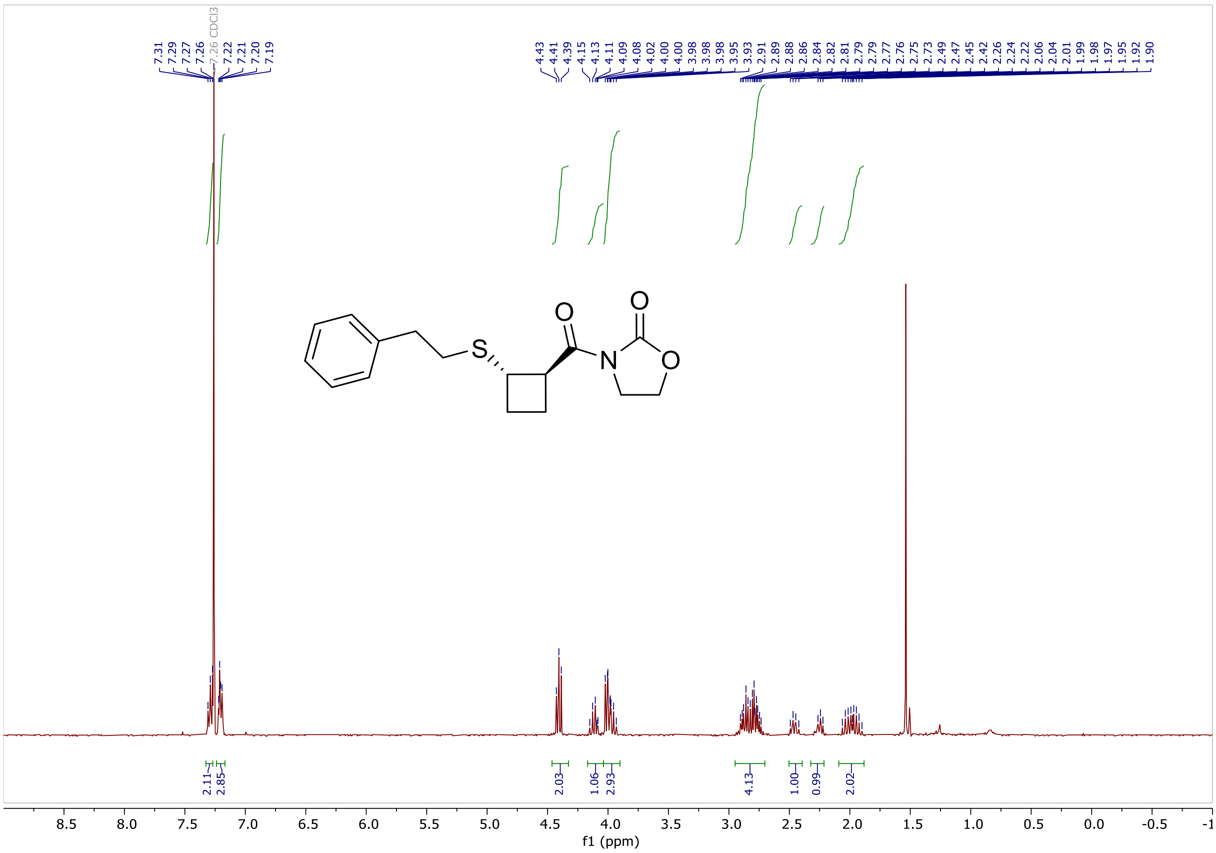

^{13}C-NMR (101 MHz, chloroform-*d*) (4j)

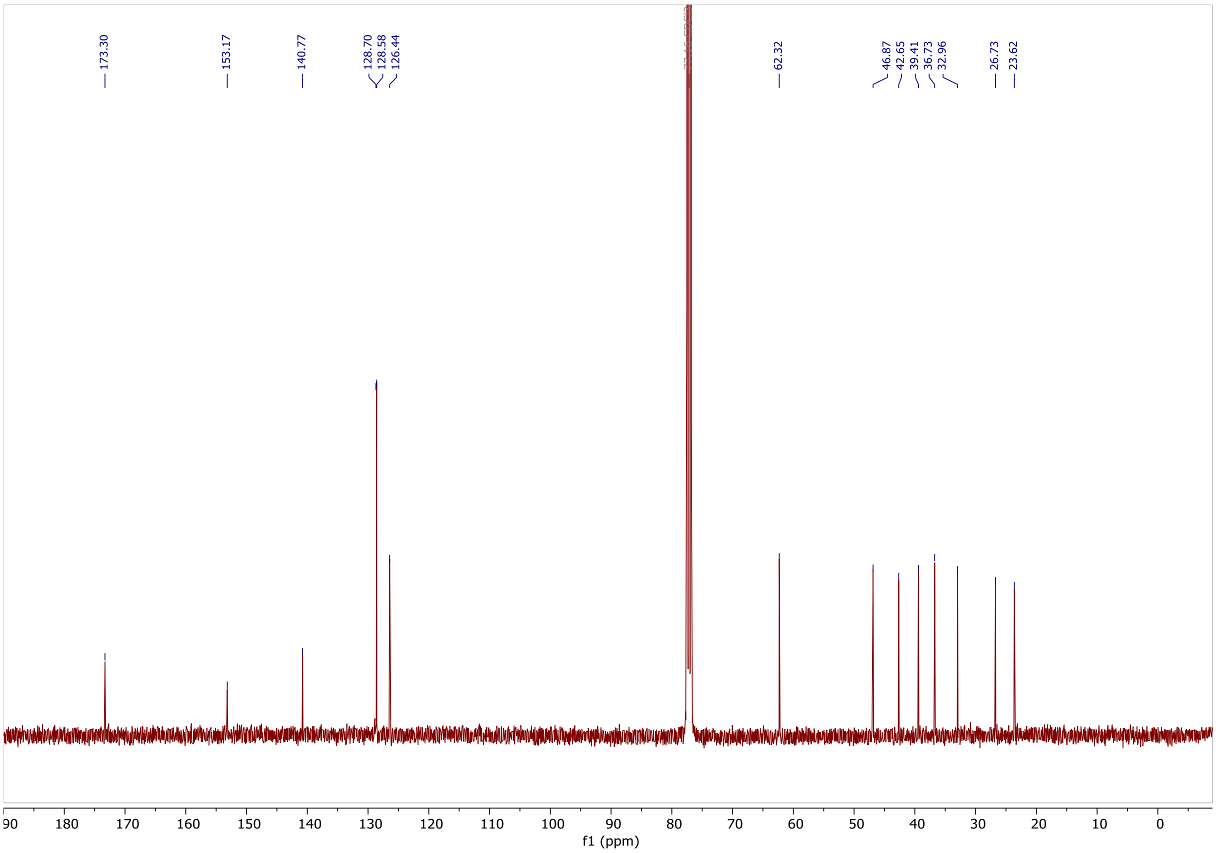

^{1}H-NMR (400 MHz, chloroform-*d*) (5)

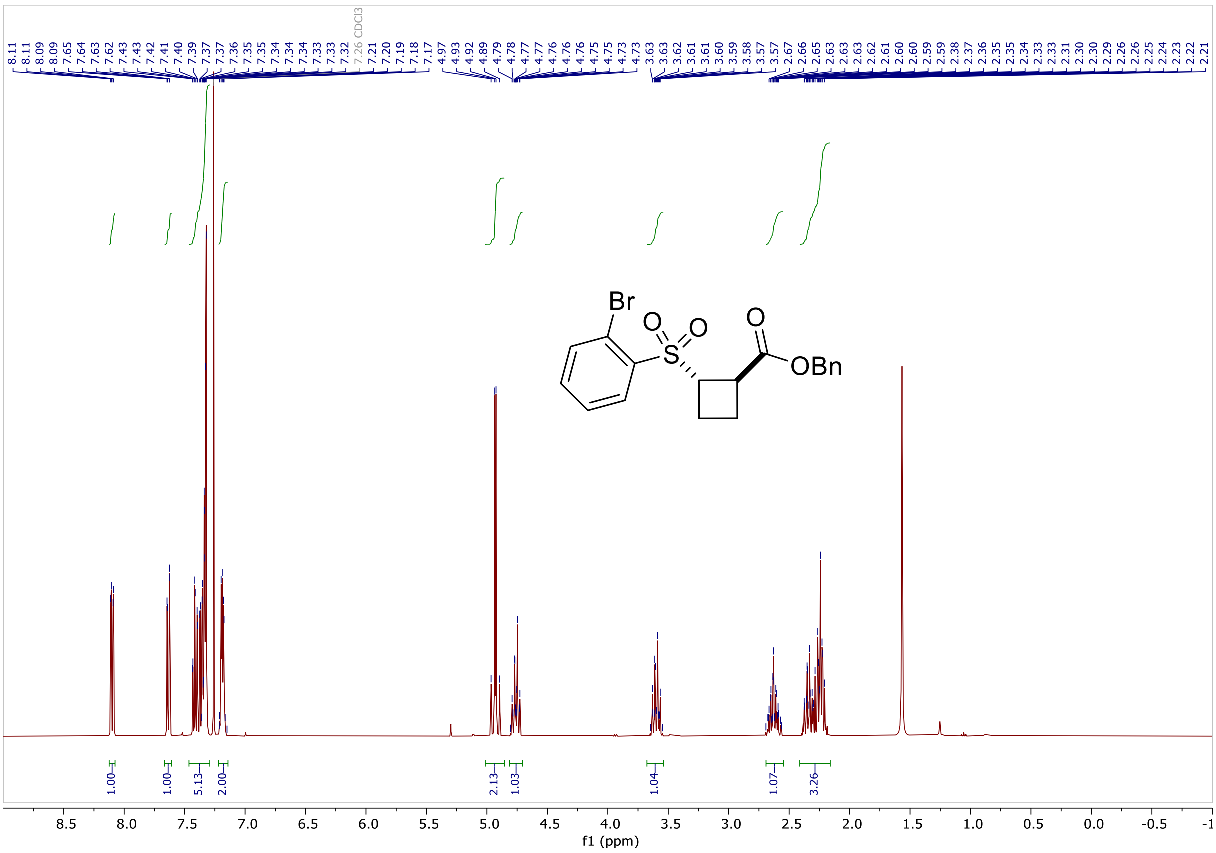

^{13}C-NMR (101 MHz, chloroform-*d*) (5)

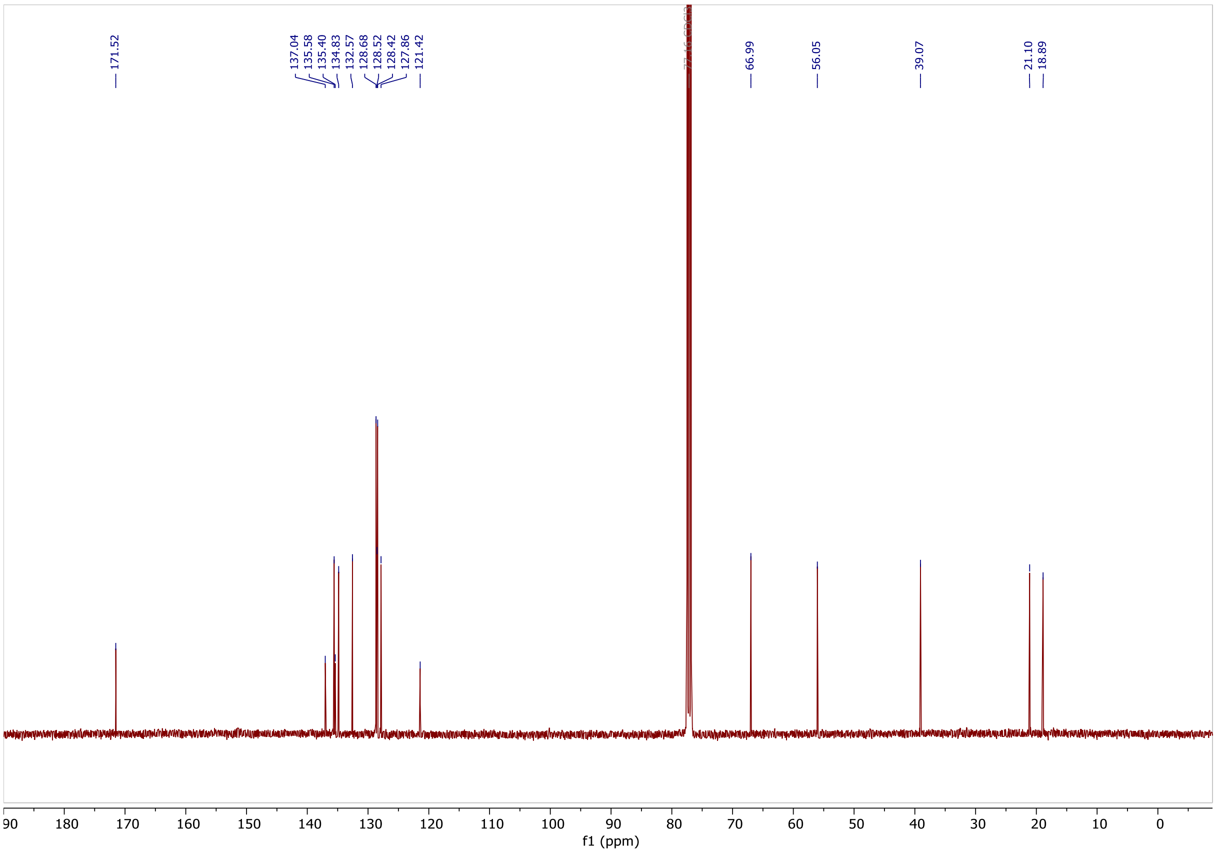

^{1}H-NMR (400 MHz, chloroform-*d*) (6)

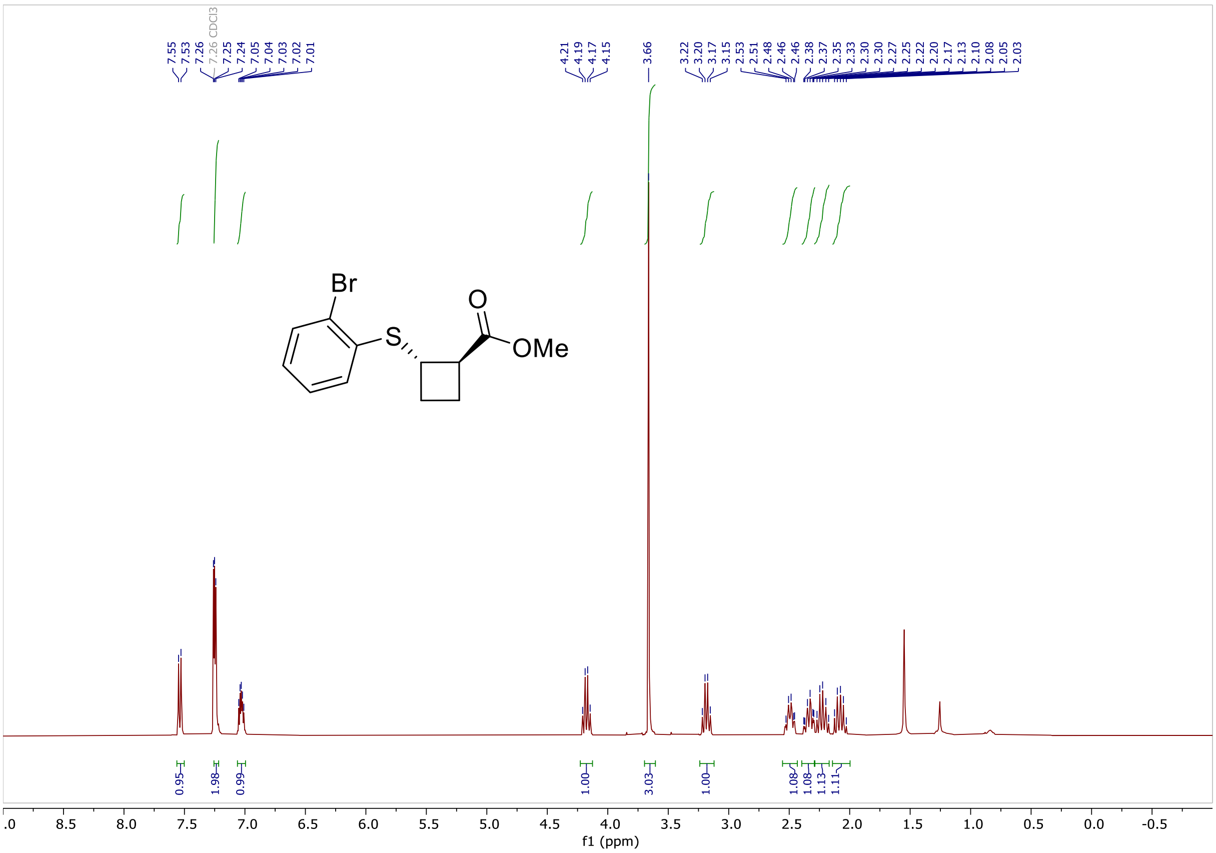

^{13}C-NMR (101 MHz, chloroform-*d*) (6)

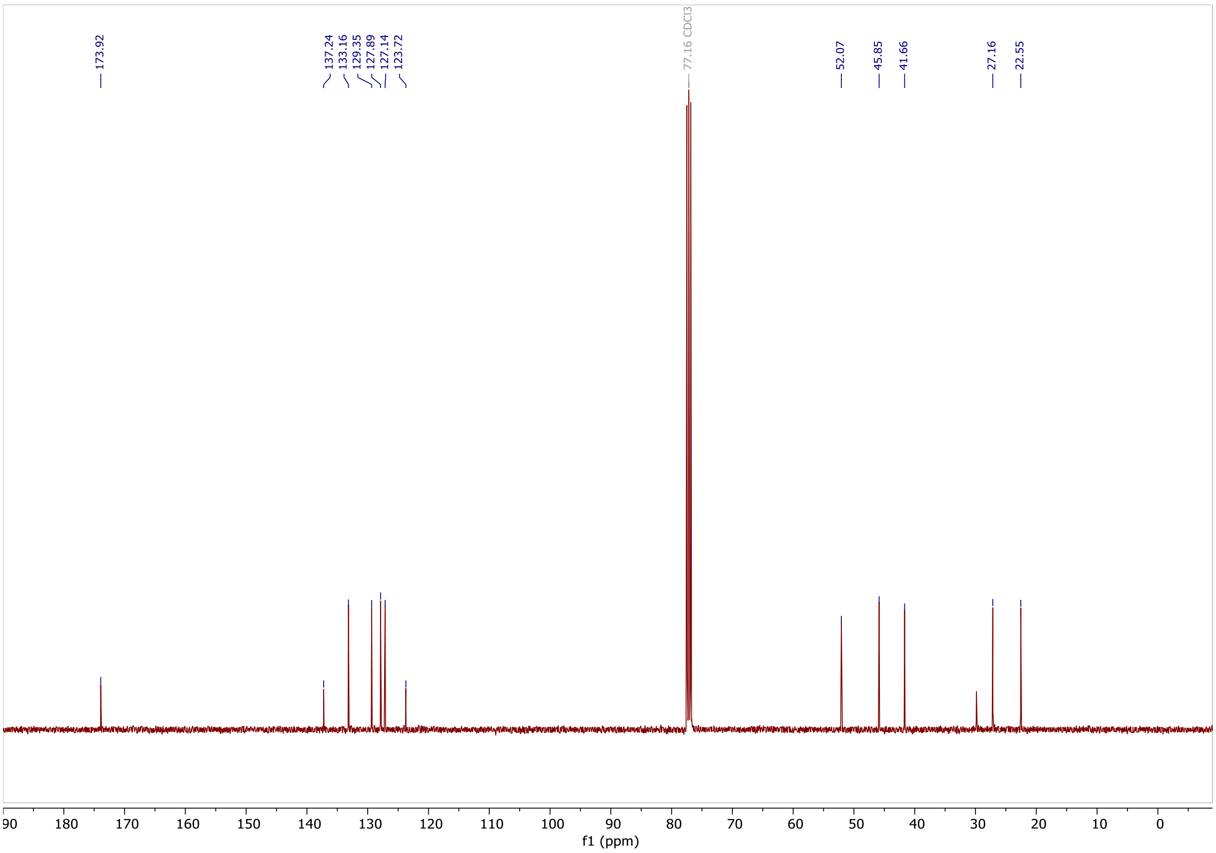

^{1}H-NMR (400 MHz, chloroform-*d*) (7)

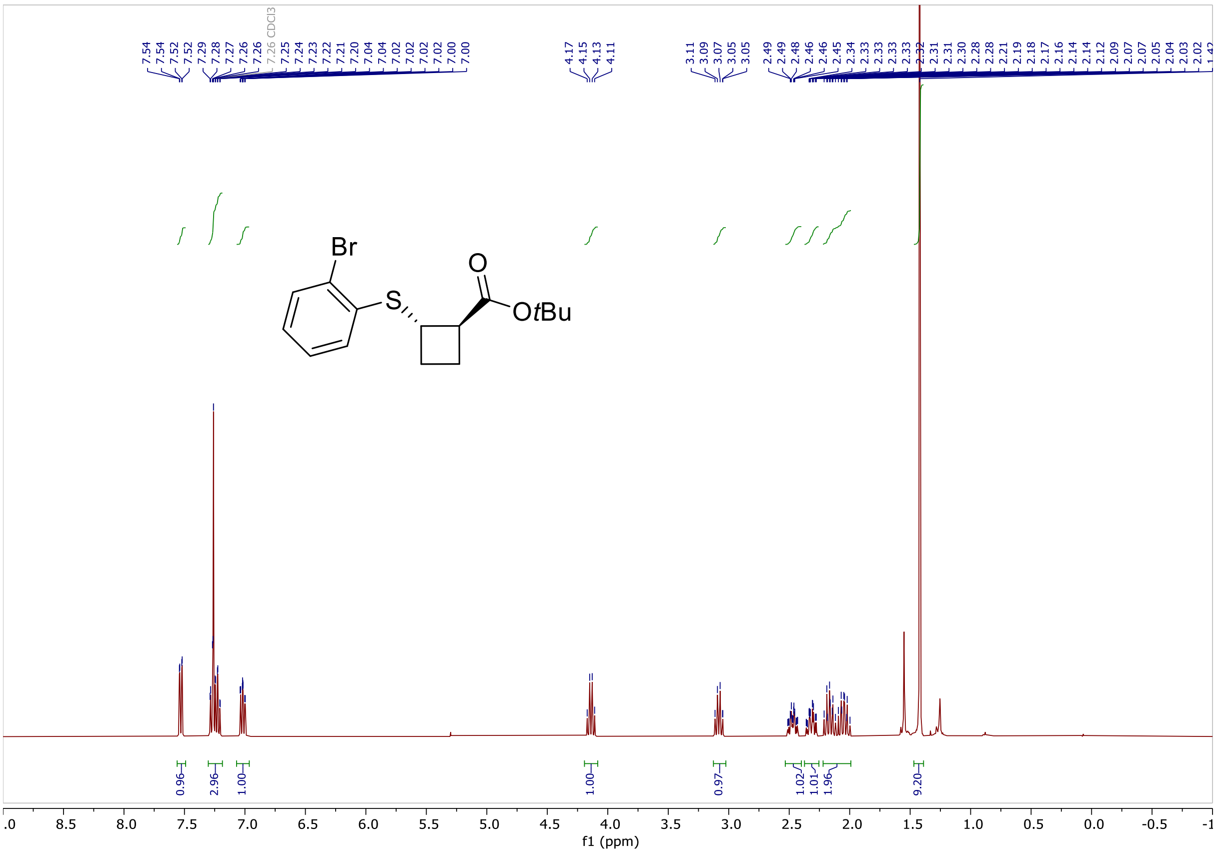

^{13}C-NMR (101 MHz, chloroform-*d*) (7)

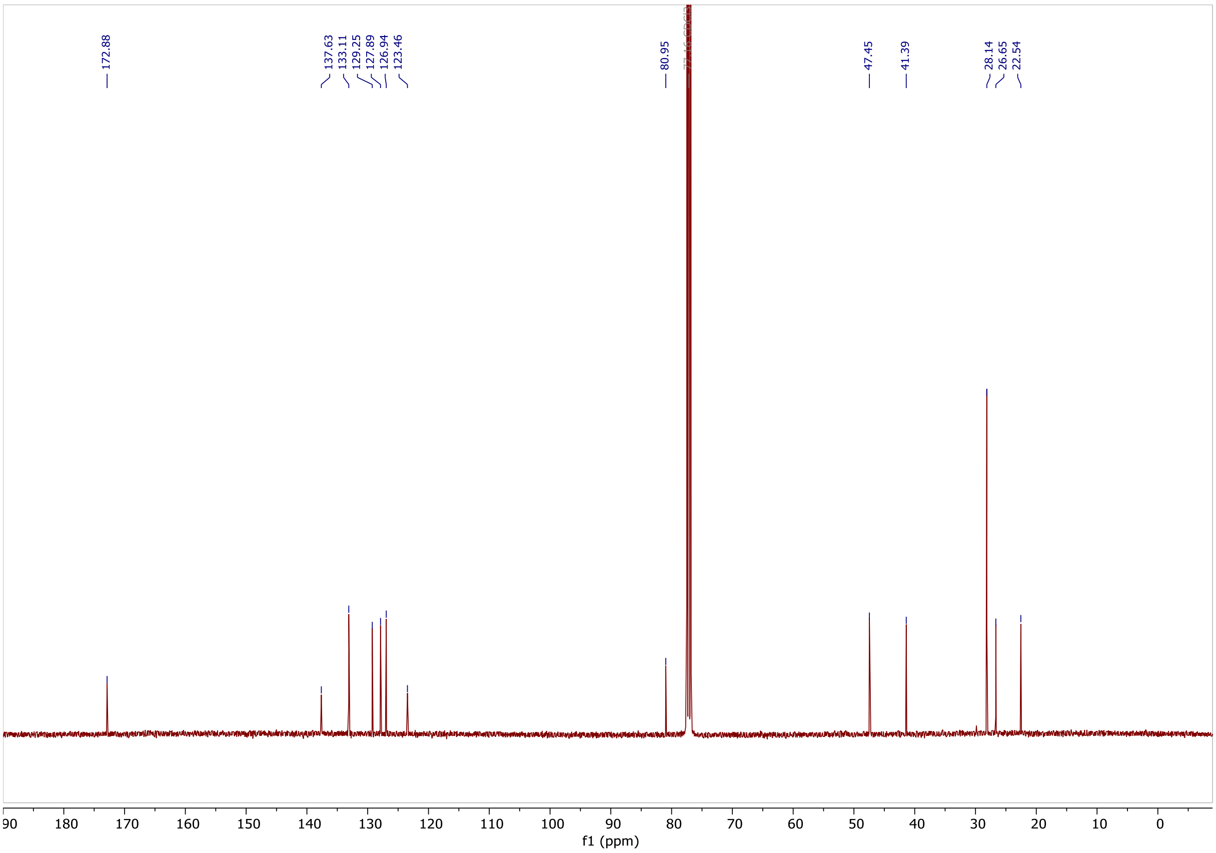

^{1}H-NMR (400 MHz, chloroform-*d*) (8)

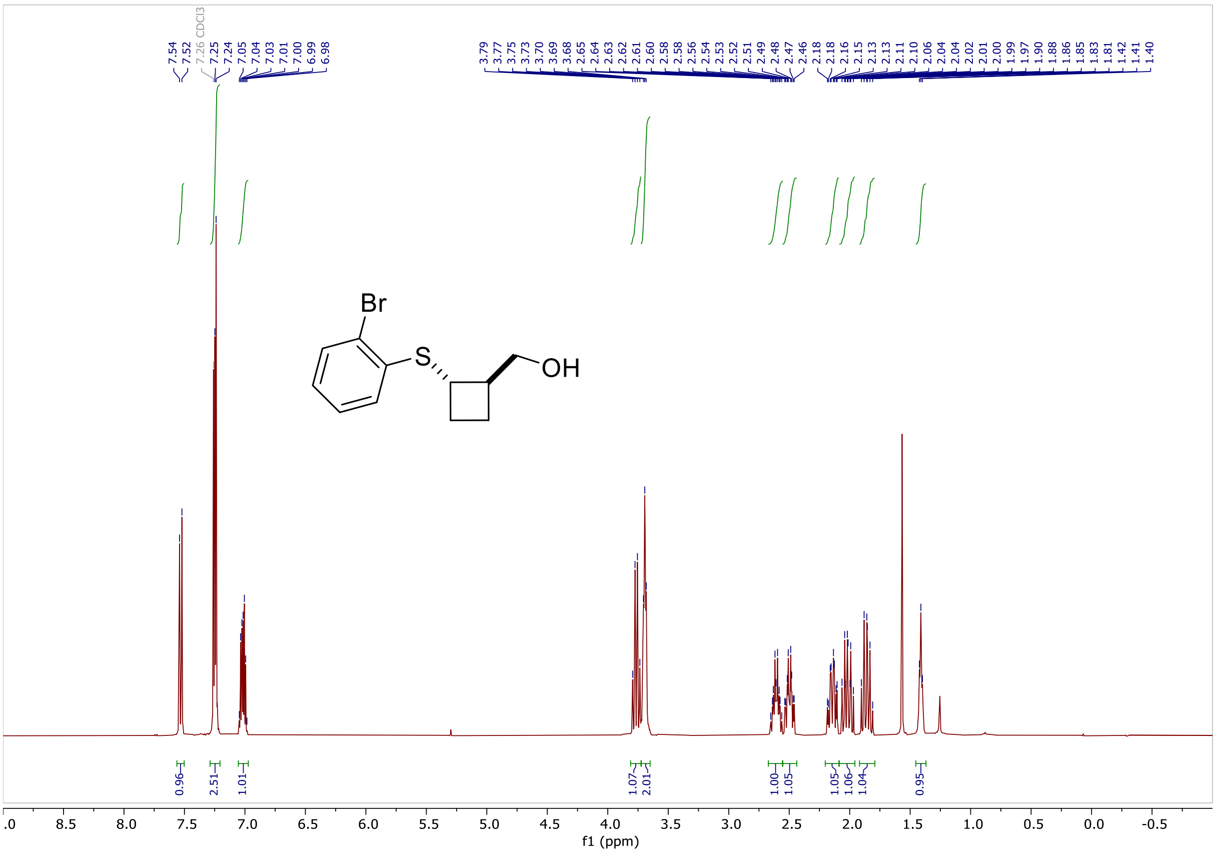

^{13}C-NMR (101 MHz, chloroform-*d*) (8)

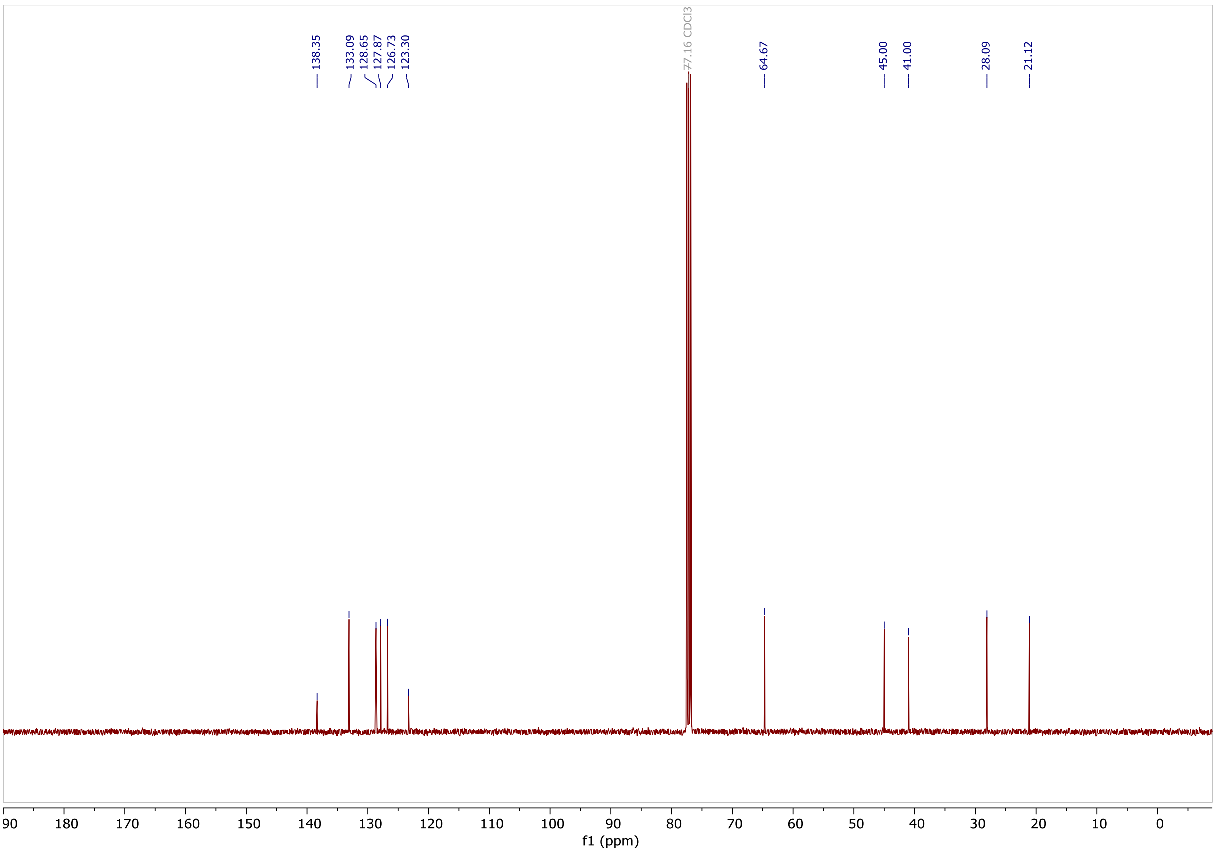

^{1}H-NMR (400 MHz, chloroform-*d*) (9)

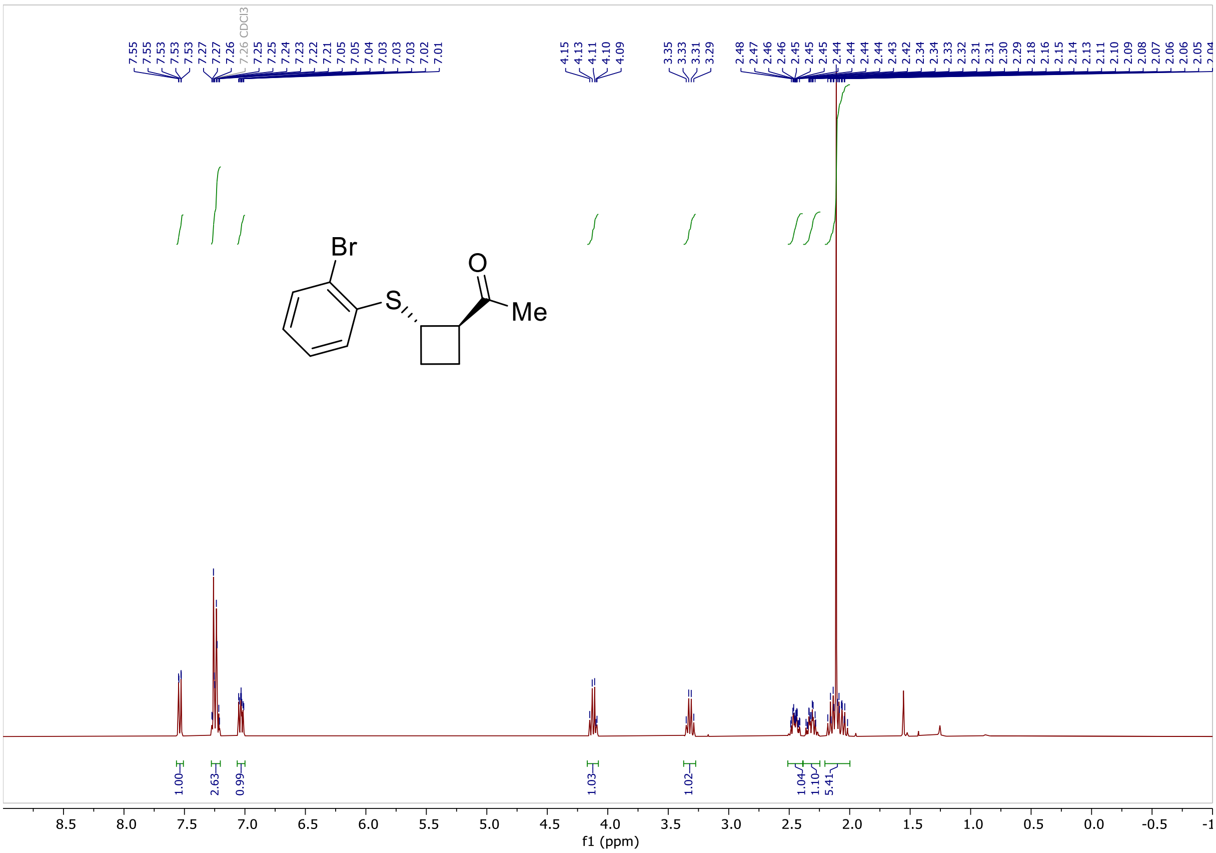

^{13}C-NMR (101 MHz, chloroform-*d*) (9)

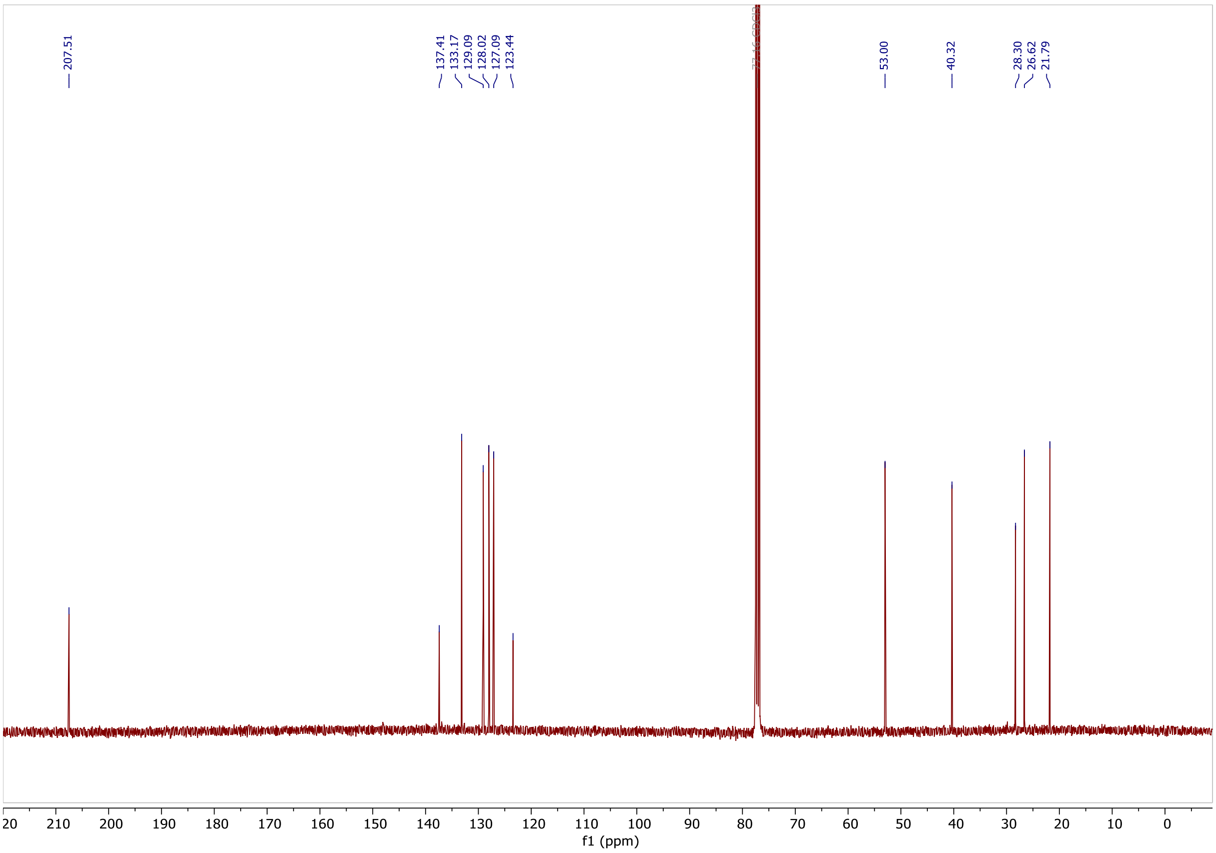

^{1}H-NMR (400 MHz, chloroform-*d*) (10)

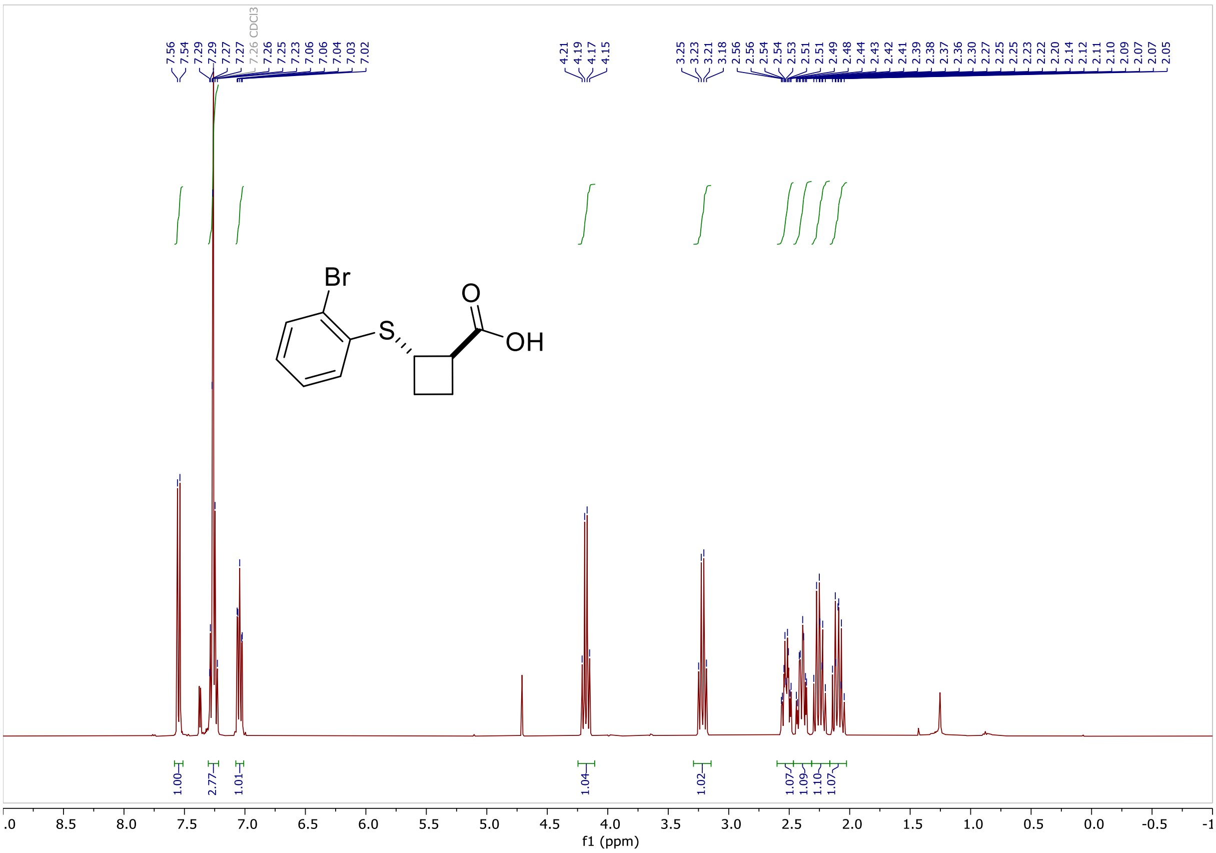

^{13}C-NMR (101 MHz, chloroform-*d*) (10)

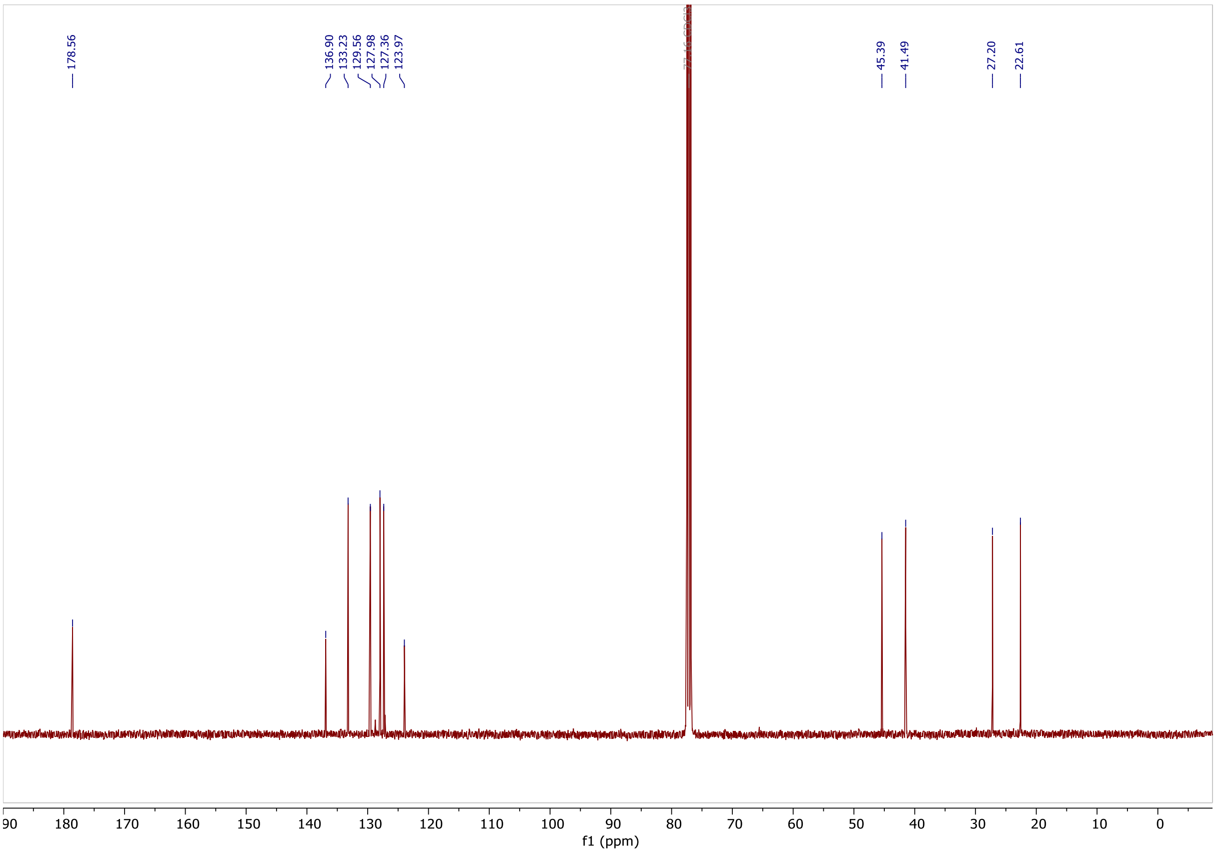

5. SFC traces

4a

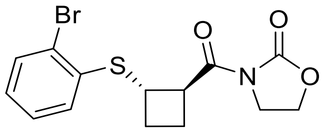

Racemic:

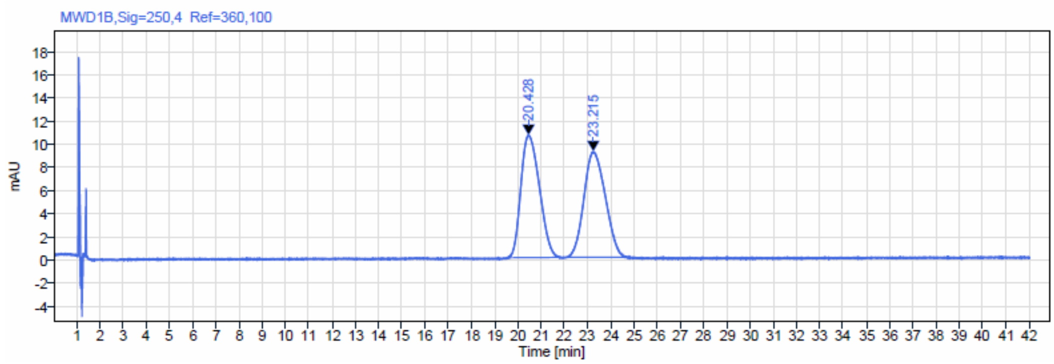

Signal:	MWD1B,Sig=250,4 Ref=360,100					
RT [min]	Type	Width [min]	Area	Height	Area%	Name
20.428	MM m	2.18	586.09	10.58	50.07	
23.215	MM m	2.81	584.40	9.15	49.93	
		**Sum**	**1170.50**			

Enantioenriched:

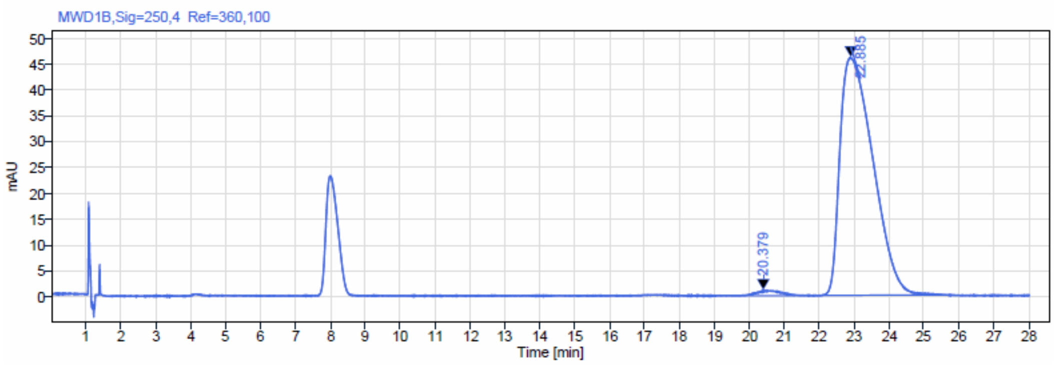

Signal:	MWD1B,Sig=250,4 Ref=360,100					
RT [min]	Type	Width [min]	Area	Height	Area%	Name
20.379	MM m	1.94	48.91	1.13	1.58	
22.885	MM m	3.59	3042.04	46.37	98.42	
		**Sum**	**3090.94**			

4b

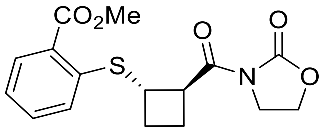

Racemic:

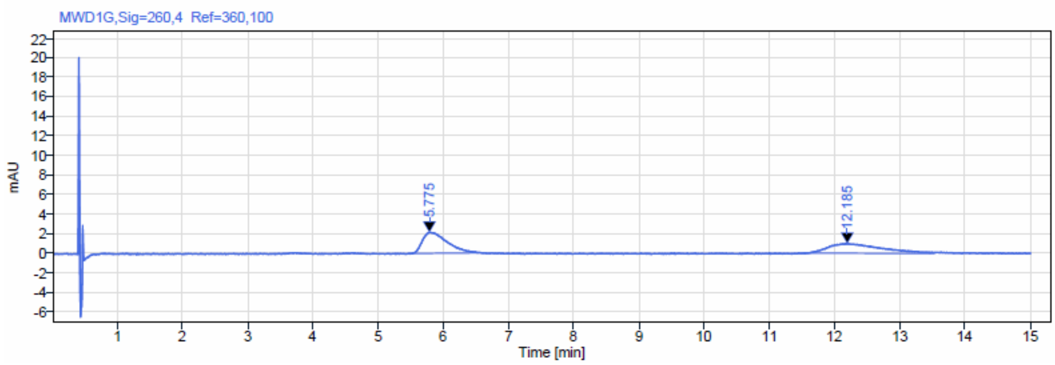

Signal: MWD1G,Sig=260,4 Ref=360,100

RT [min]	Type	Width [min]	Area	Height	Area%	Name
5.775	MM m	1.12	56.78	2.14	50.30	
12.185	MM m	2.01	56.10	1.00	49.70	
		**Sum**	**112.88**			

Enantioenriched:

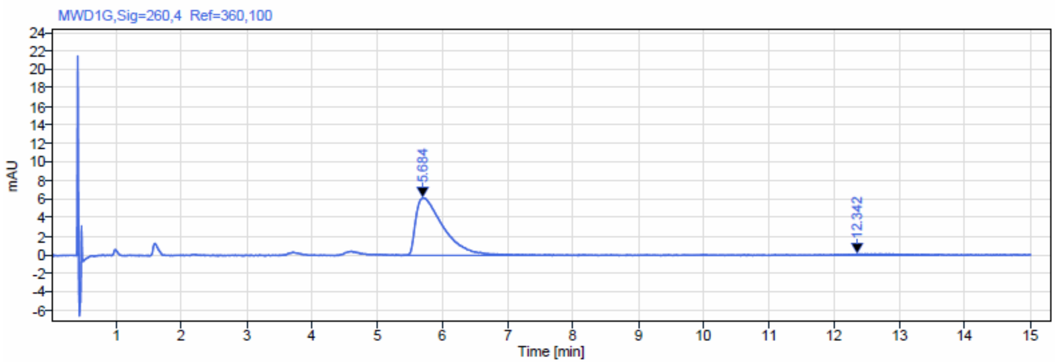

Signal: MWD1G,Sig=260,4 Ref=360,100

RT [min]	Type	Width [min]	Area	Height	Area%	Name
5.684	MM m	2.25	183.16	6.21	96.72	
12.342	MM m	1.59	6.20	0.14	3.28	
		**Sum**	**189.37**			

4c

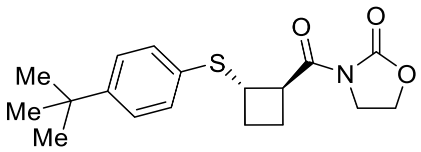

Racemic:

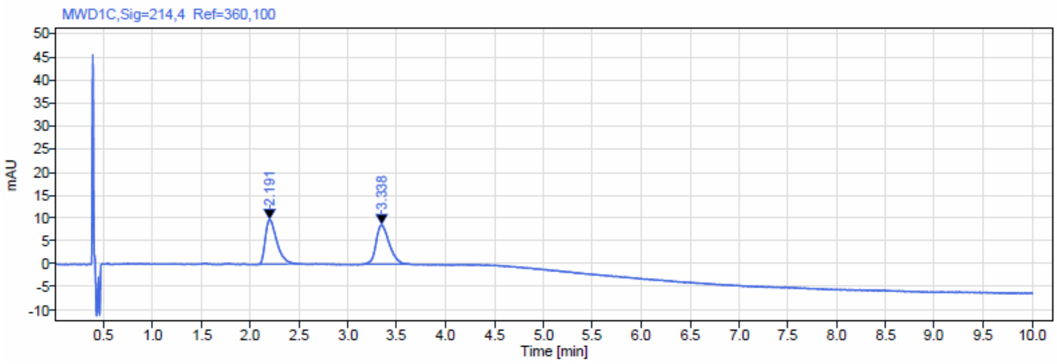

Signal: MWD1C,Sig=214,4 Ref=360,100

RT [min]	Type	Width [min]	Area	Height	Area%	Name
2.191	MM m	0.46	78.84	9.61	49.65	
3.338	MM m	0.48	79.95	8.53	50.35	
		**Sum**	**158.80**			

Enantioenriched:

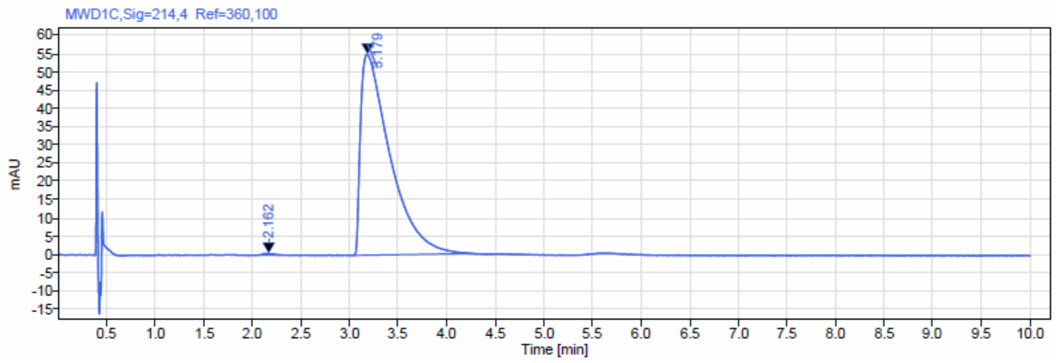

Signal: MWD1C,Sig=214,4 Ref=360,100

RT [min]	Type	Width [min]	Area	Height	Area%	Name
2.162	MM m	0.27	3.28	0.49	0.28	
3.179	MM m	1.21	1158.09	55.01	99.72	
		**Sum**	**1161.37**			

4d

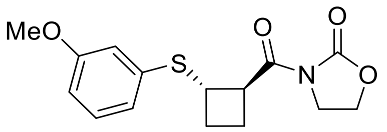

Racemic:

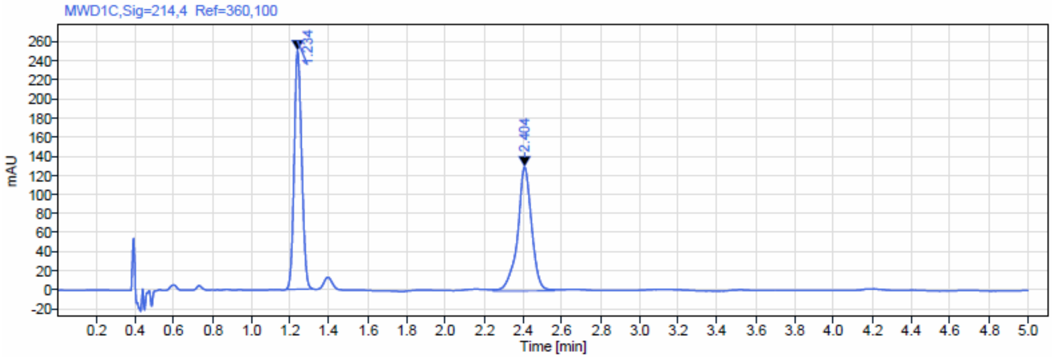

Signal: MWD1C,Sig=214,4 Ref=360,100

RT [min]	Type	Width [min]	Area	Height	Area%	Name
1.234	MM m	0.14	675.15	250.22	50.73	
2.404	MM m	0.31	655.65	130.07	49.27	
		**Sum**	**1330.80**			

Enantioenriched:

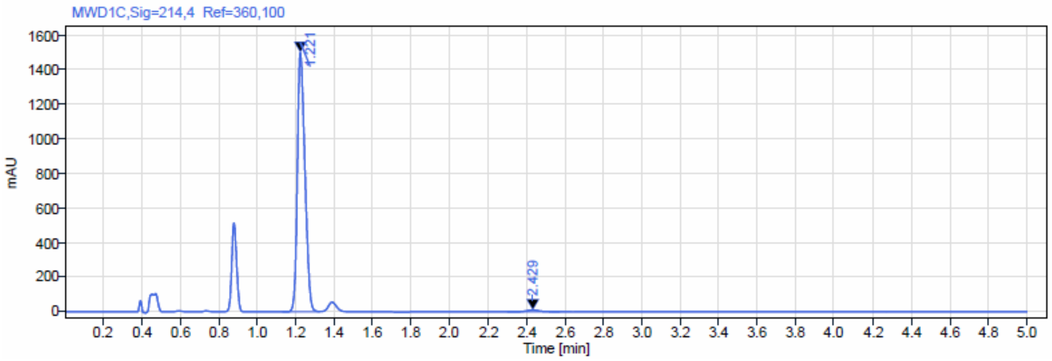

Signal: MWD1C,Sig=214,4 Ref=360,100

RT [min]	Type	Width [min]	Area	Height	Area%	Name
1.221	MM m	0.19	4115.04	1502.97	98.86	
2.429	MM m	0.27	47.62	9.91	1.14	
		**Sum**	**4162.66**			

4e

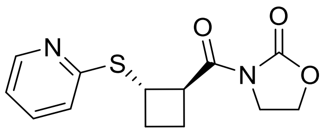

Racemic:

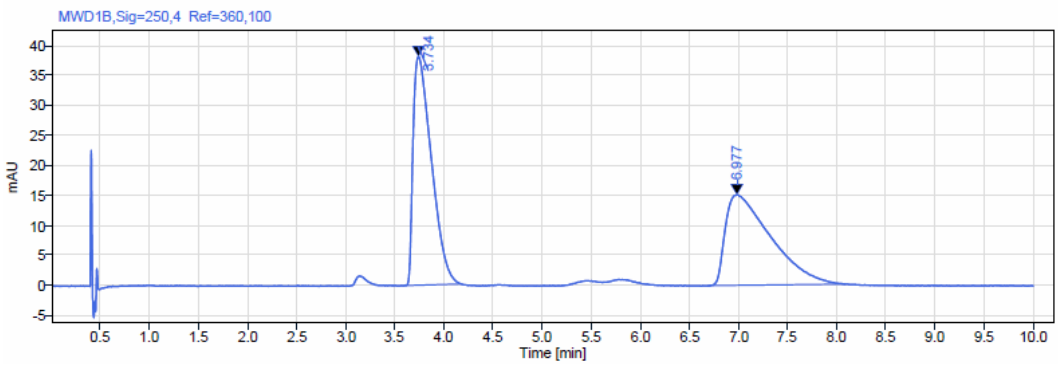

Signal: MWD1B,Sig=250,4 Ref=360,100

RT [min]	Type	Width [min]	Area	Height	Area%	Name
3.734	MM m	0.58	494.08	38.02	50.52	
6.977	MM m	1.40	483.91	15.03	49.48	
		**Sum**	**977.99**			

Enantioenriched:

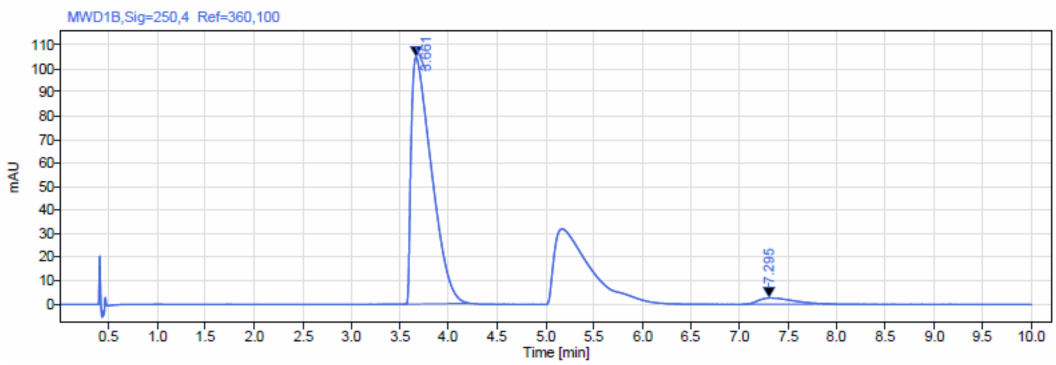

Signal: MWD1B,Sig=250,4 Ref=360,100

RT [min]	Type	Width [min]	Area	Height	Area%	Name
3.661	MM m	0.69	1527.96	105.02	95.94	
7.295	MM m	0.89	64.66	2.65	4.06	
		**Sum**	**1592.62**			

4f

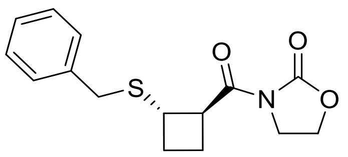

Racemic:

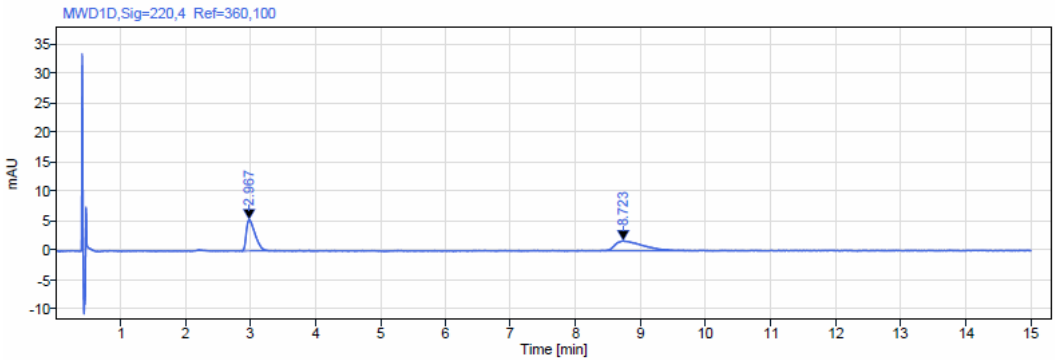

Signal:	MWD1D,Sig=220,4 Ref=360,100					
**RT [min]**	**Type**	**Width [min]**	**Area**	**Height**	**Area%**	**Name**
2.967	MM m	0.44	49.36	5.26	50.29	
8.723	MM m	1.19	48.78	1.65	49.71	
		**Sum**	**98.14**			

Enantioenriched:

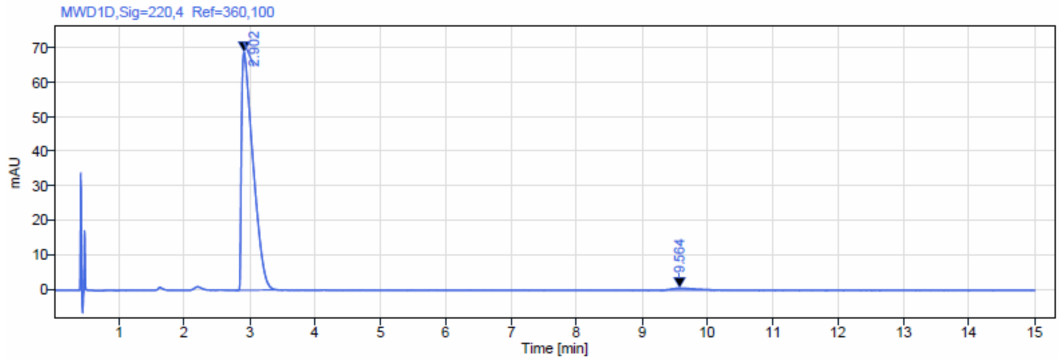

Signal:	MWD1D,Sig=220,4 Ref=360,100					
**RT [min]**	**Type**	**Width [min]**	**Area**	**Height**	**Area%**	**Name**
2.902	MM m	0.67	842.60	68.81	98.13	
9.564	MM m	0.85	16.04	0.64	1.87	
		**Sum**	**858.64**			

4g

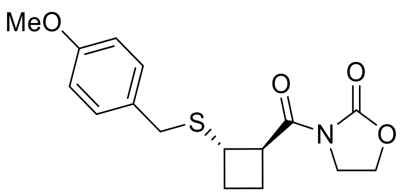

Racemic:

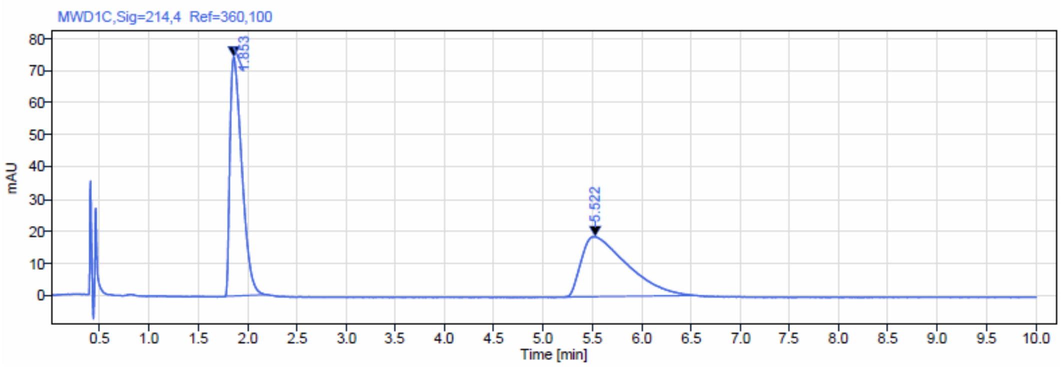

Signal: MWD1C,Sig=214,4 Ref=360,100

RT [min]	Type	Width [min]	Area	Height	Area%	Name
1.853	MM m	0.42	623.46	74.21	50.87	
5.522	MM m	1.33	602.09	18.63	49.13	
		**Sum**	**1225.54**			

Enantioenriched:

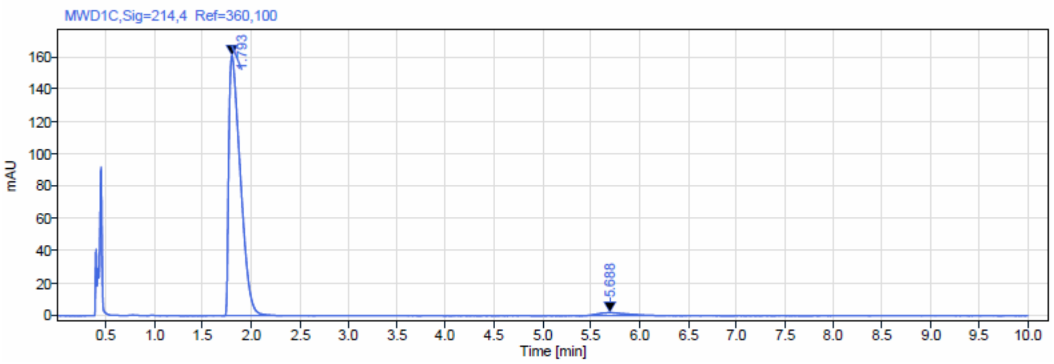

Signal: MWD1C,Sig=214,4 Ref=360,100

RT [min]	Type	Width [min]	Area	Height	Area%	Name
1.793	MM m	0.61	1333.80	161.03	96.94	
5.688	MM m	0.93	42.12	1.92	3.06	
		**Sum**	**1375.92**			

4h

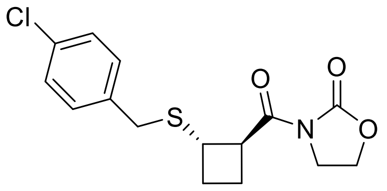

Racemic:

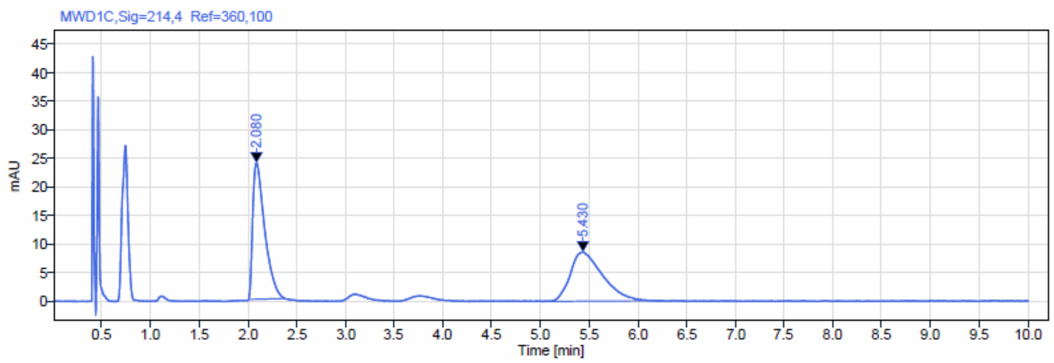

Signal: MWD1C,Sig=214,4 Ref=360,100

RT [min]	Type	Width [min]	Area	Height	Area%	Name
2.080	MM m	0.37	204.16	23.91	50.63	
5.430	MM m	1.22	199.10	8.63	49.37	
		**Sum**	**403.26**			

Enantioenriched:

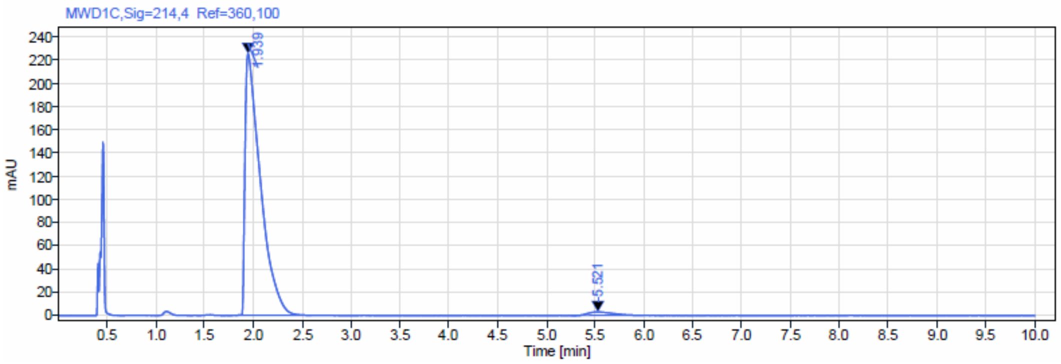

Signal: MWD1C,Sig=214,4 Ref=360,100

RT [min]	Type	Width [min]	Area	Height	Area%	Name
1.939	MM m	0.78	2478.73	225.92	97.91	
5.521	MM m	0.73	52.95	3.01	2.09	
		**Sum**	**2531.69**			

4i

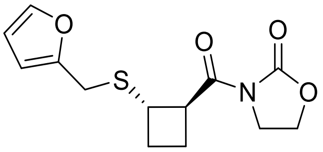

Racemic:

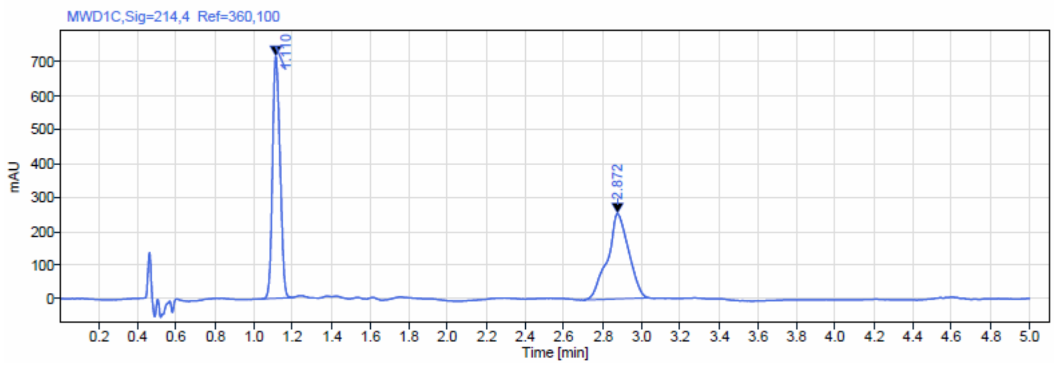

Signal: MWD1C,Sig=214,4 Ref=360,100

RT [min]	Type	Width [min]	Area	Height	Area%	Name
1.110	MM m	0.18	1930.71	713.17	50.31	
2.872	MM m	0.34	1906.66	251.18	49.69	
		**Sum**	**3837.37**			

Enantioenriched:

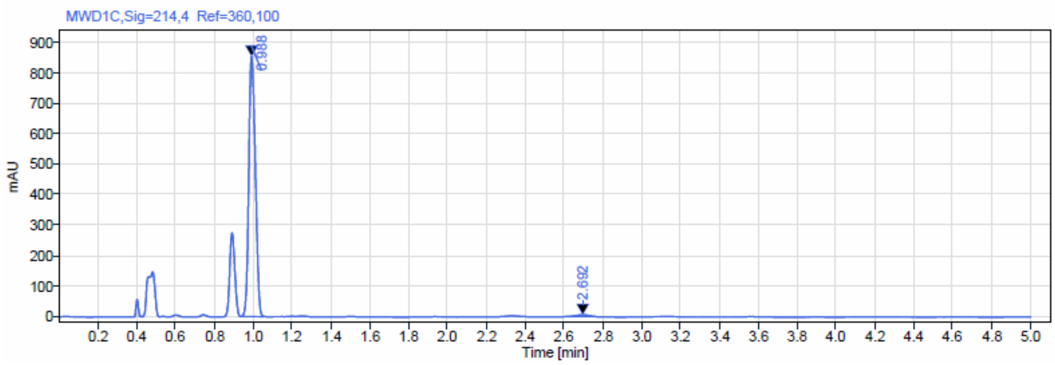

Signal: MWD1C,Sig=214,4 Ref=360,100

RT [min]	Type	Width [min]	Area	Height	Area%	Name
0.988	MM m	0.11	2004.09	855.40	98.70	
2.692	MM m	0.15	26.35	6.15	1.30	
		**Sum**	**2030.44**			

4j

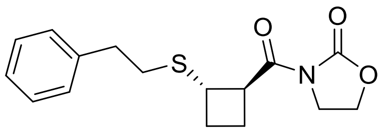

Racemic:

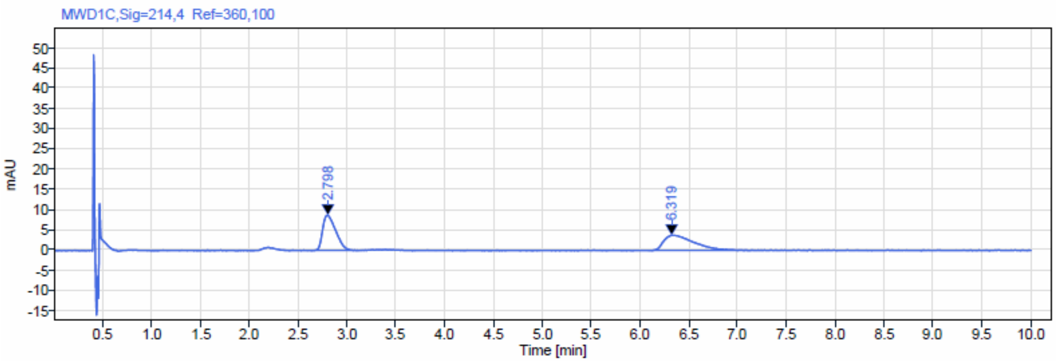

Signal: MWD1C,Sig=214,4 Ref=360,100

RT [min]	Type	Width [min]	Area	Height	Area%	Name
2.798	MM m	0.49	80.74	8.69	49.86	
6.319	MM m	1.05	81.21	3.73	50.14	
		**Sum**	**161.95**			

Enantioenriched:

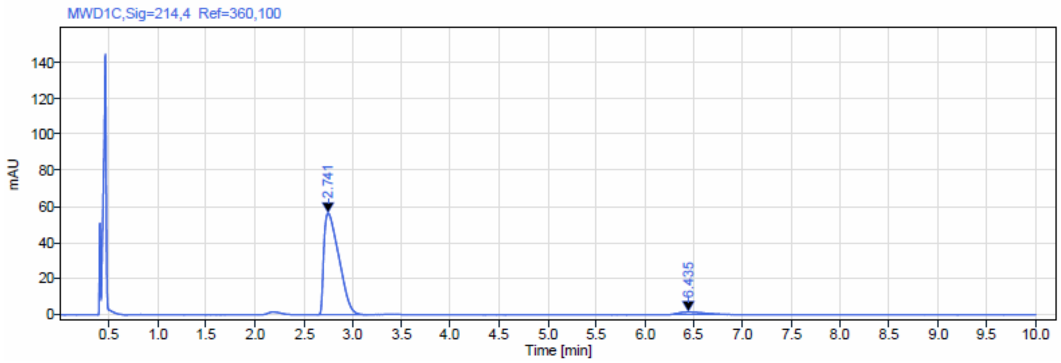

Signal: MWD1C,Sig=214,4 Ref=360,100

RT [min]	Type	Width [min]	Area	Height	Area%	Name
2.741	MM m	0.52	602.55	56.51	95.35	
6.435	MM m	0.86	29.37	1.50	4.65	
		**Sum**	**631.93**			

3a

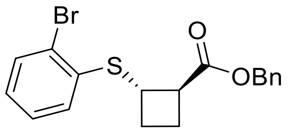

Racemic:

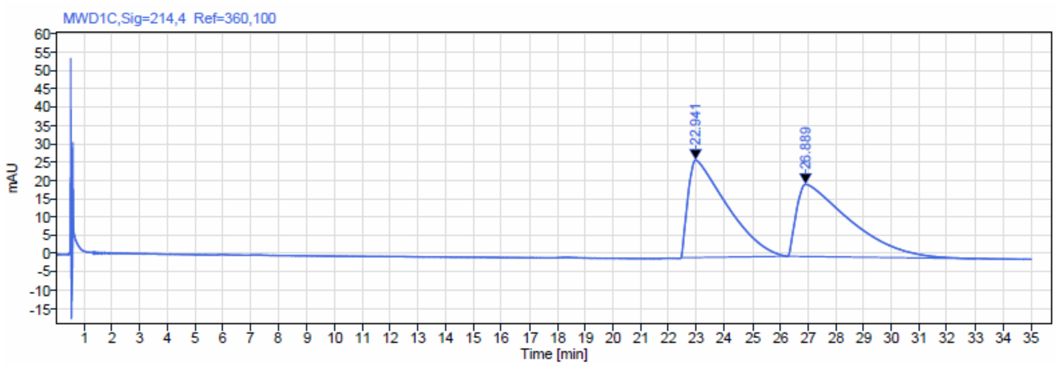

Signal: MWD1C,Sig=214,4 Ref=360,100

RT [min]	Type	Width [min]	Area	Height	Area%	Name
22.941	MM m	3.79	2575.75	26.64	49.75	
26.889	MM m	6.79	2601.47	19.75	50.25	
		**Sum**	**5177.22**			

Enantioenriched:

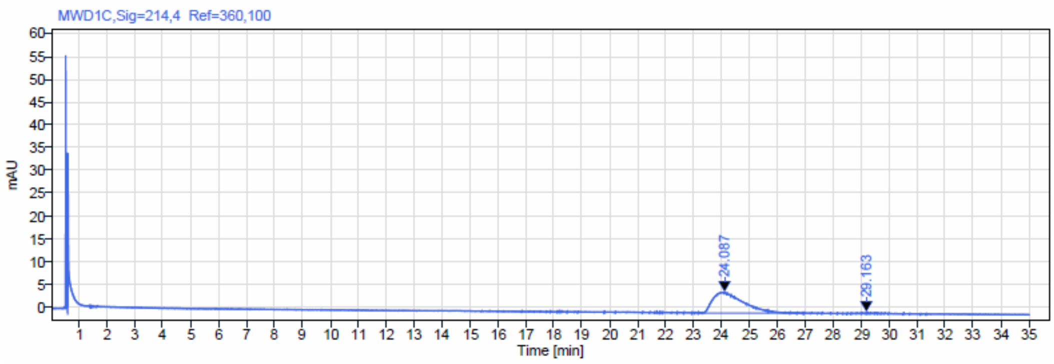

Signal: MWD1C,Sig=214,4 Ref=360,100

RT [min]	Type	Width [min]	Area	Height	Area%	Name
24.087	MM m	3.87	346.47	4.61	97.11	
29.163	MM m	2.10	10.31	0.38	2.89	
		**Sum**	**356.78**			

5

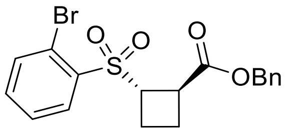

Racemic:

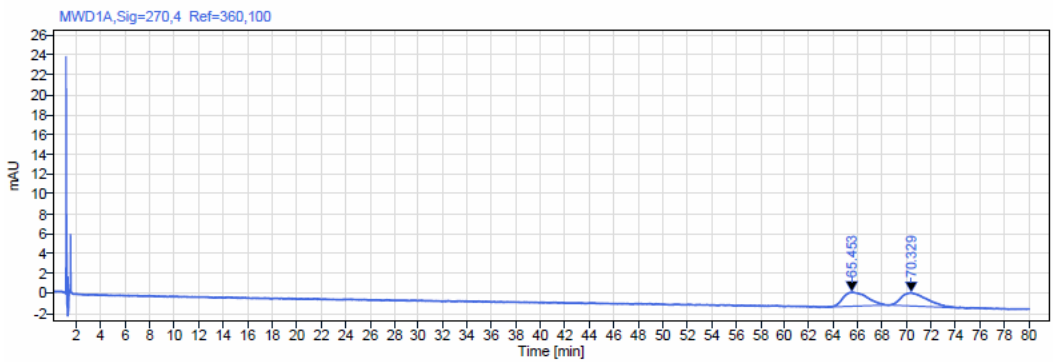

Signal: MWD1A,Sig=270,4 Ref=360,100

RT [min]	Type	Width [min]	Area	Height	Area%	Name
65.453	MM m	4.96	192.54	1.43	49.66	
70.329	MM m	5.85	195.19	1.35	50.34	
		**Sum**	**387.73**			

Enantioenriched:

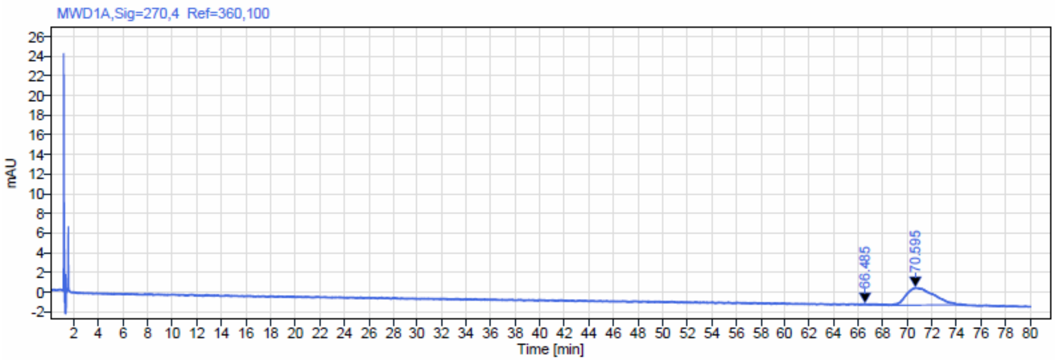

Signal: MWD1A,Sig=270,4 Ref=360,100

RT [min]	Type	Width [min]	Area	Height	Area%	Name
66.485	MM m	2.35	8.21	0.15	2.89	
70.595	MM m	5.87	276.20	1.79	97.11	
		**Sum**	**284.41**			

6

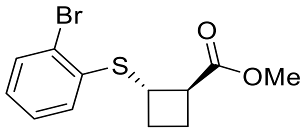

Racemic:

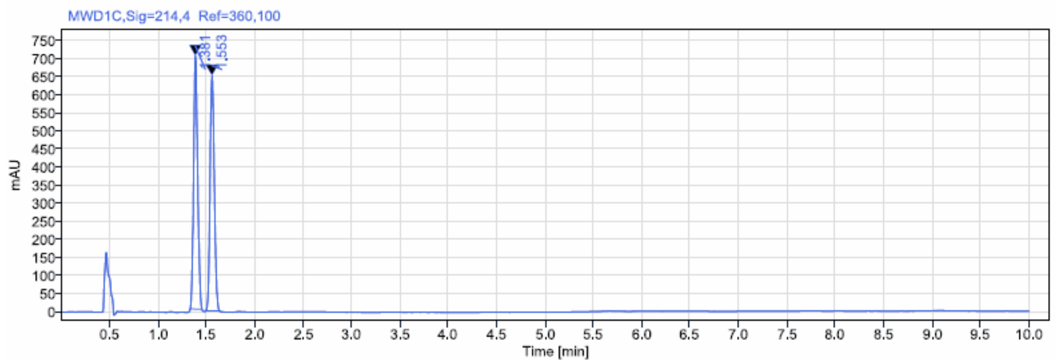

Signal: MWD1C,Sig=214,4 Ref=360,100

RT [min]	Type	Width [min]	Area	Height	Area%	Name
1,381	MM m	0,12	2048,17	701,02	50,80	
1,553	MM m	0,15	1983,75	652,35	49,20	
		**Sum**	**4031,92**			

Enantioenriched:

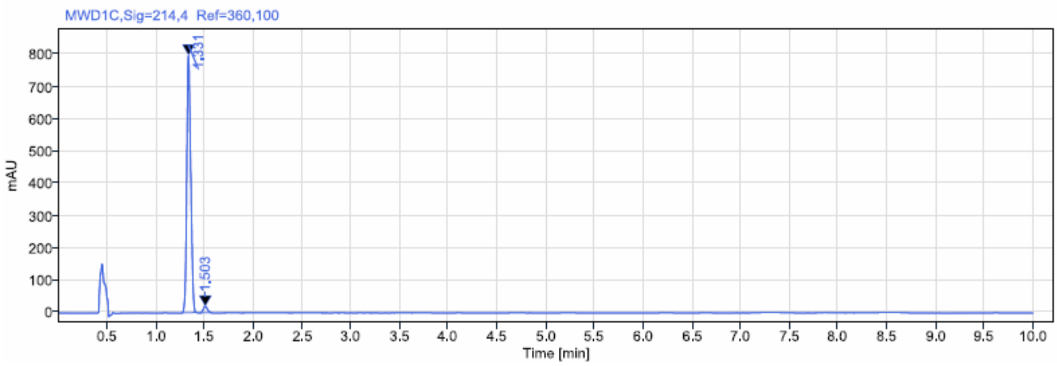

Signal: MWD1C,Sig=214,4 Ref=360,100

RT [min]	Type	Width [min]	Area	Height	Area%	Name
1,331	MM m	0,14	2253,58	796,81	97,41	
1,503	MM m	0,12	59,87	19,92	2,59	
		**Sum**	**2313,45**			

7

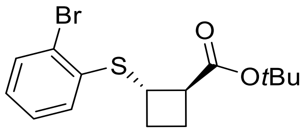

Racemic:

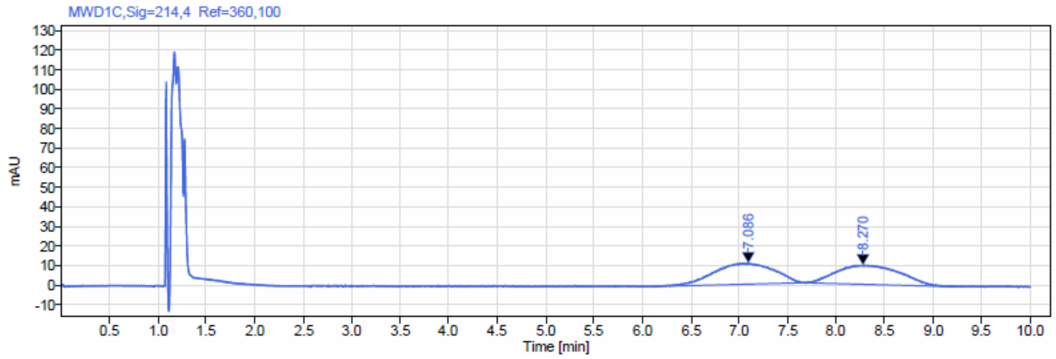

Signal:	MWD1C,Sig=214,4 Ref=360,100					
**RT [min]**	**Type**	**Width [min]**	**Area**	**Height**	**Area%**	**Name**
7.086	MM m	1.39	459.75	10.77	50.76	
8.270	MM m	1.55	446.04	9.77	49.24	
		**Sum**	**905.79**			

Enantioenriched:

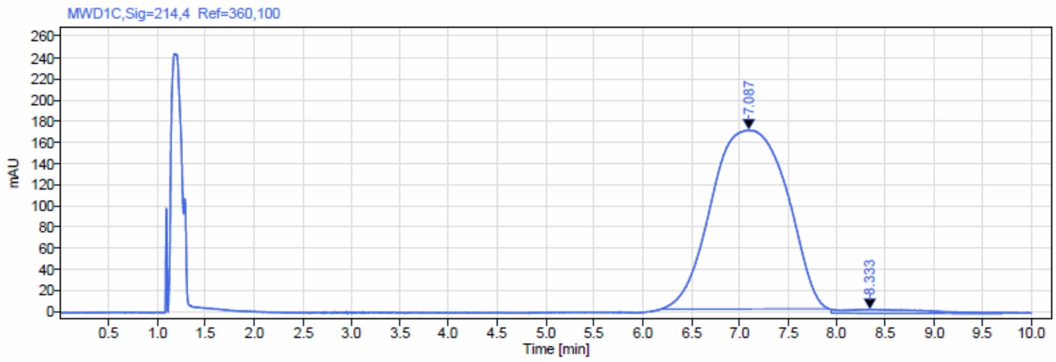

Signal:	MWD1C,Sig=214,4 Ref=360,100					
**RT [min]**	**Type**	**Width [min]**	**Area**	**Height**	**Area%**	**Name**
7.087	MM m	1.72	9077.36	168.66	97.37	
8.333	MM m	1.76	245.18	3.66	2.63	
		**Sum**	**9322.54**			

8

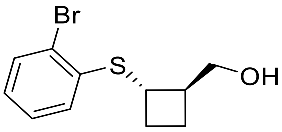

Racemic:

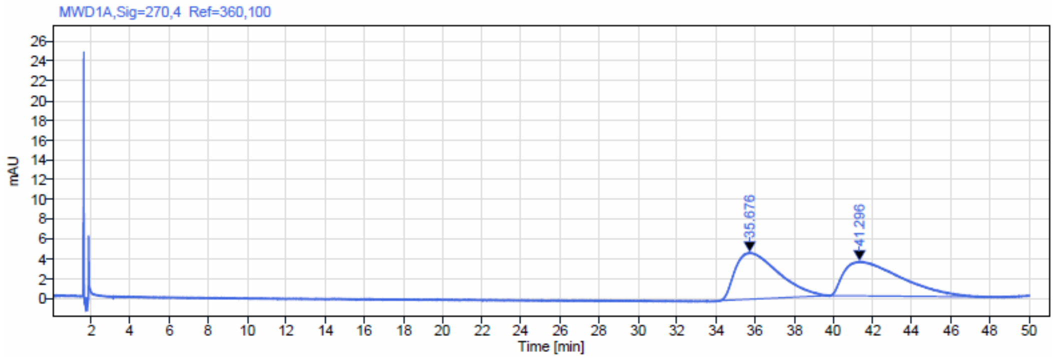

Signal: MWD1A,Sig=270,4 Ref=360,100

RT [min]	Type	Width [min]	Area	Height	Area%	Name
35.676	MM m	5.55	719.05	4.76	50.44	
41.296	MM m	8.55	706.47	3.52	49.56	
		**Sum**	**1425.52**			

Enantioenriched:

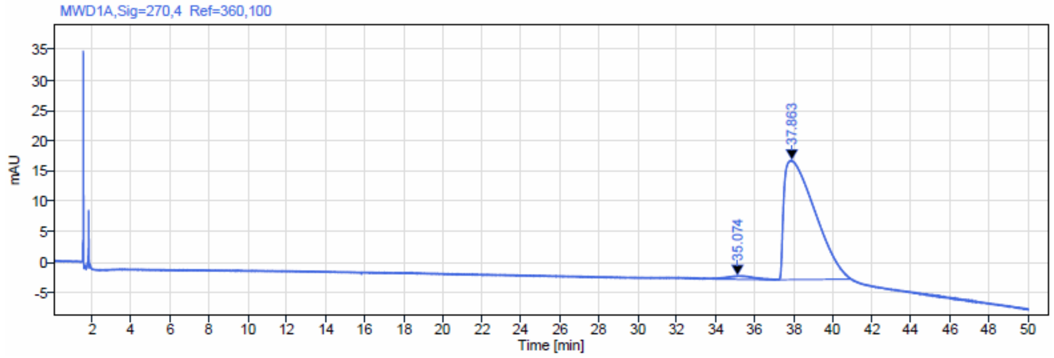

Signal: MWD1A,Sig=270,4 Ref=360,100

RT [min]	Type	Width [min]	Area	Height	Area%	Name
35.074	MM m	3.50	55.69	0.63	2.51	
37.863	MM m	3.69	2159.88	19.69	97.49	
		**Sum**	**2215.57**			

3z

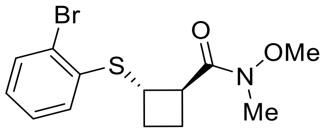

Racemic:

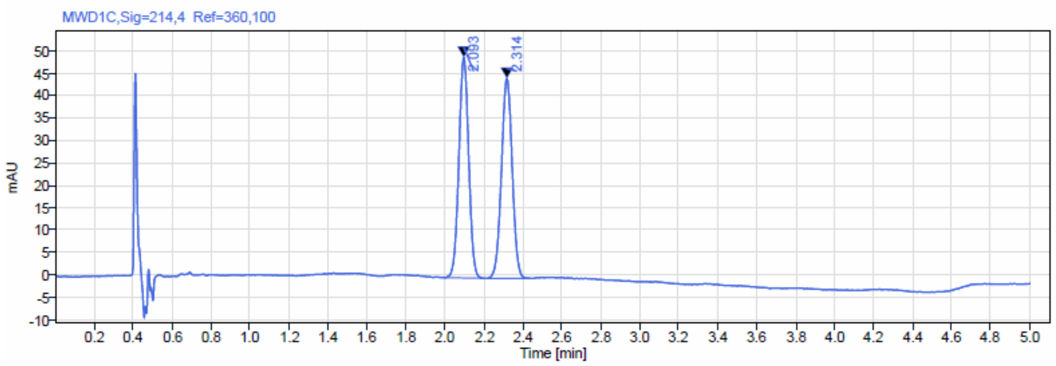

Signal:	MWD1C,Sig=214,4 Ref=360,100					
**RT [min]**	**Type**	**Width [min]**	**Area**	**Height**	**Area%**	**Name**
2.093	MM m	0.23	171.67	49.15	50.08	
2.314	MM m	0.23	171.11	44.56	49.92	
		**Sum**	**342.78**			

Enantioenriched:

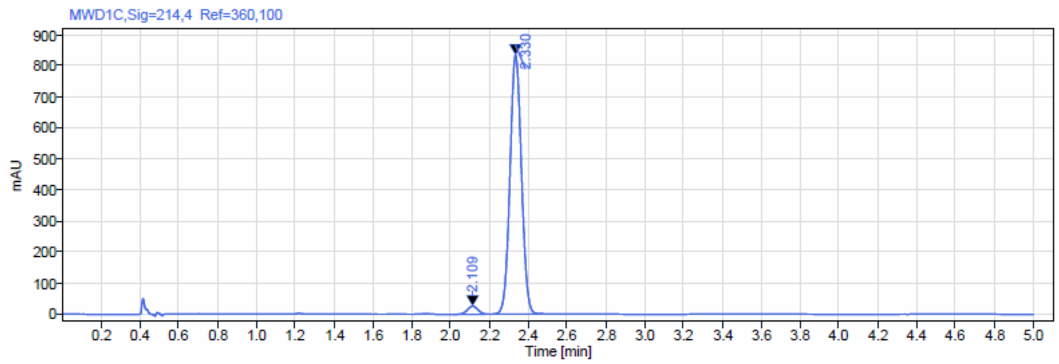

Signal:	MWD1C,Sig=214,4 Ref=360,100					
**RT [min]**	**Type**	**Width [min]**	**Area**	**Height**	**Area%**	**Name**
2.109	MM m	0.20	97.87	26.17	2.78	
2.330	MM m	0.26	3424.70	836.26	97.22	
		**Sum**	**3522.57**			

9

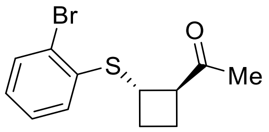

Racemic:

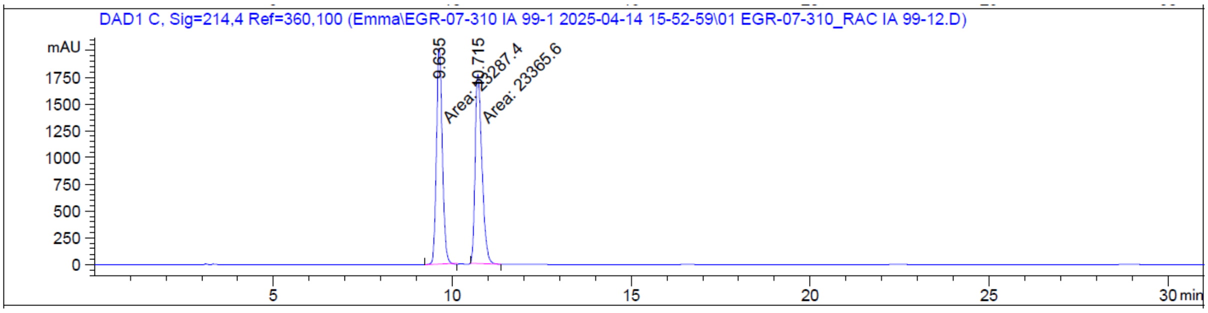

Signal 3: DAD1 C, Sig=214,4 Ref=360,100

Peak #	RetTime [min]	Type	Width [min]	Area [mAU*s]	Height [mAU]	Area %
1	9.635	MM	0.1926	2.32874e4	2015.40210	49.9162
2	10.715	MM	0.2206	2.33656e4	1764.92322	50.0838
Totals :				4.66530e4	3780.32532	

Enantioenriched:

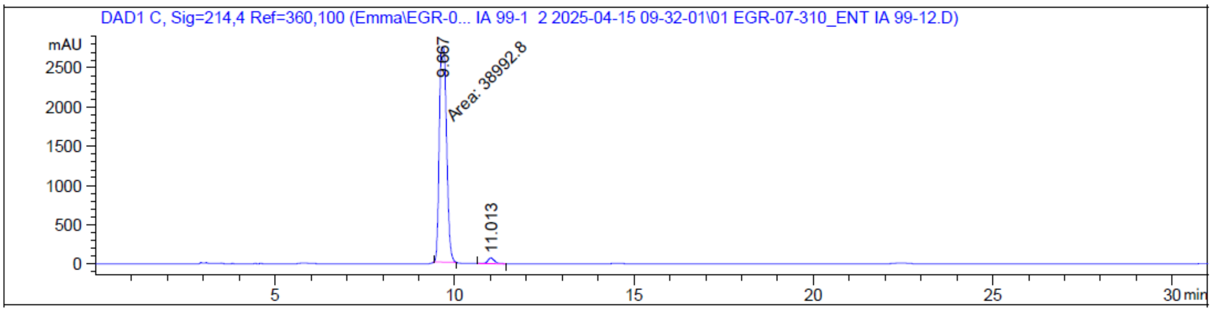

Signal 3: DAD1 C, Sig=214,4 Ref=360,100

Peak #	RetTime [min]	Type	Width [min]	Area [mAU*s]	Height [mAU]	Area %
1	9.667	MM	0.2365	3.89928e4	2748.00073	97.6764
2	11.013	BB	0.1973	927.59180	72.47859	2.3236
Totals :				3.99204e4	2820.47932	
